# Supplementary material for: Streamlined asymmetric α-difunctionalization of ynones
Source: Nat Commun. 2018 Jan 25;9:375. doi: 10.1038/s41467-017-02801-9 (PMC5785506; doi:10.1038/s41467-017-02801-9)
Supplement: Supplementary file 1 — Supplementary Information [file 41467_2017_2801_MOESM1_ESM.pdf]

Supplementary Figures

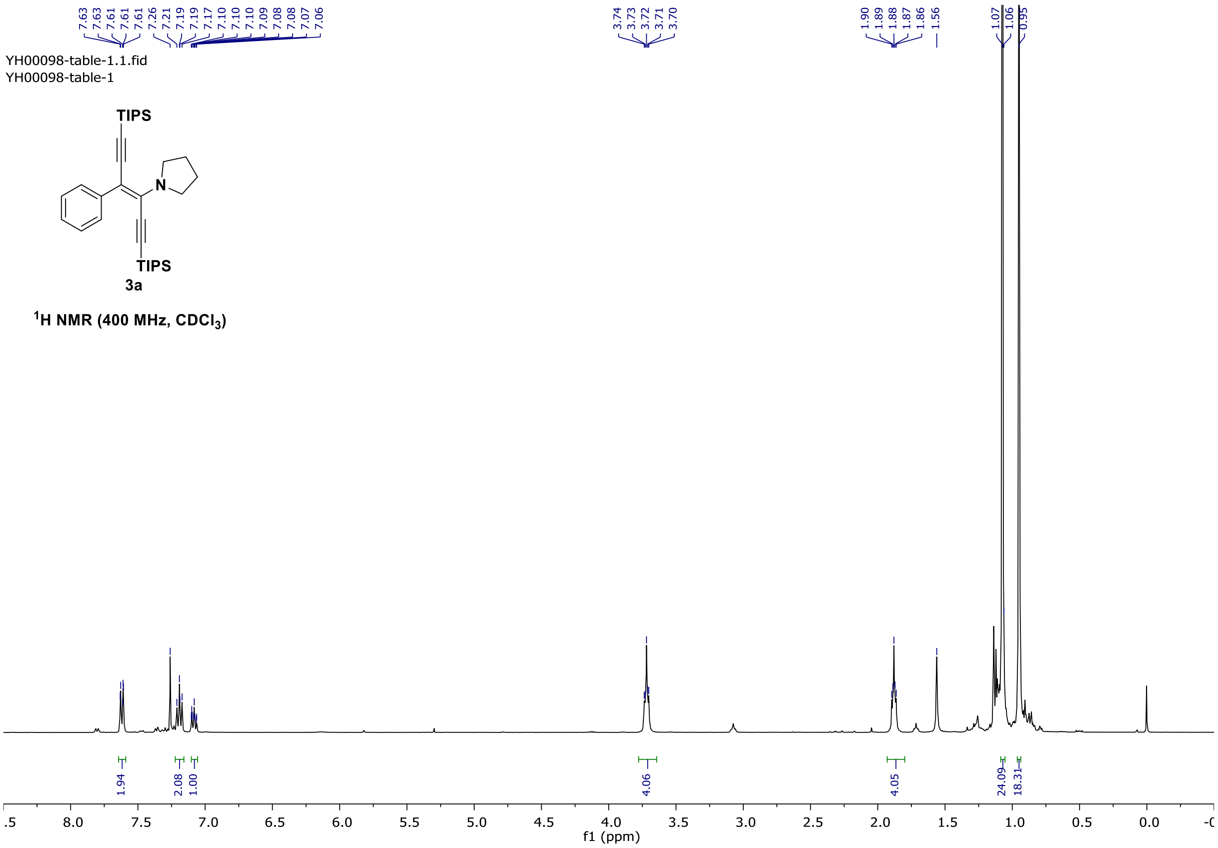

Supplementary Figure 1. ^{1}H NMR of the **3a** (400 MHz, $CDCl_3$)

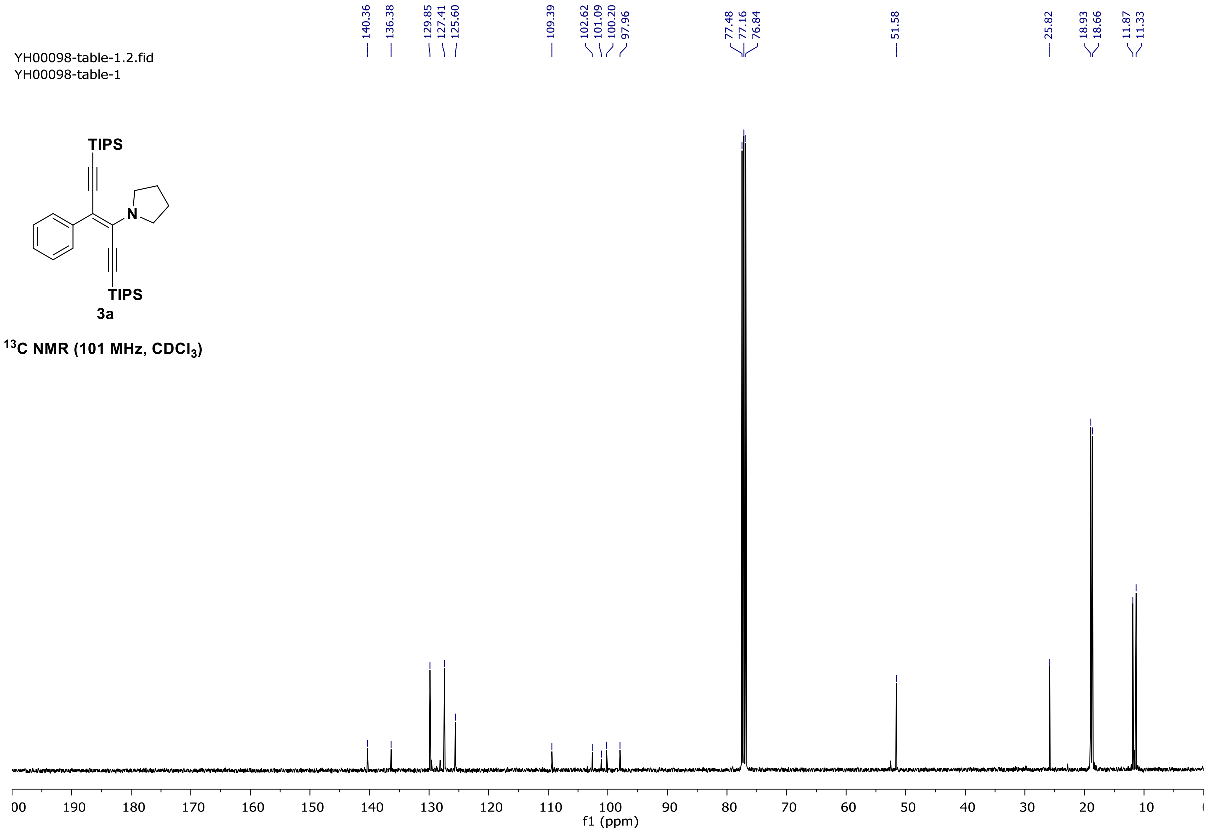

Supplementary Figure 2. ^{13}C NMR of the **3a** (101 MHz, $CDCl_3$)

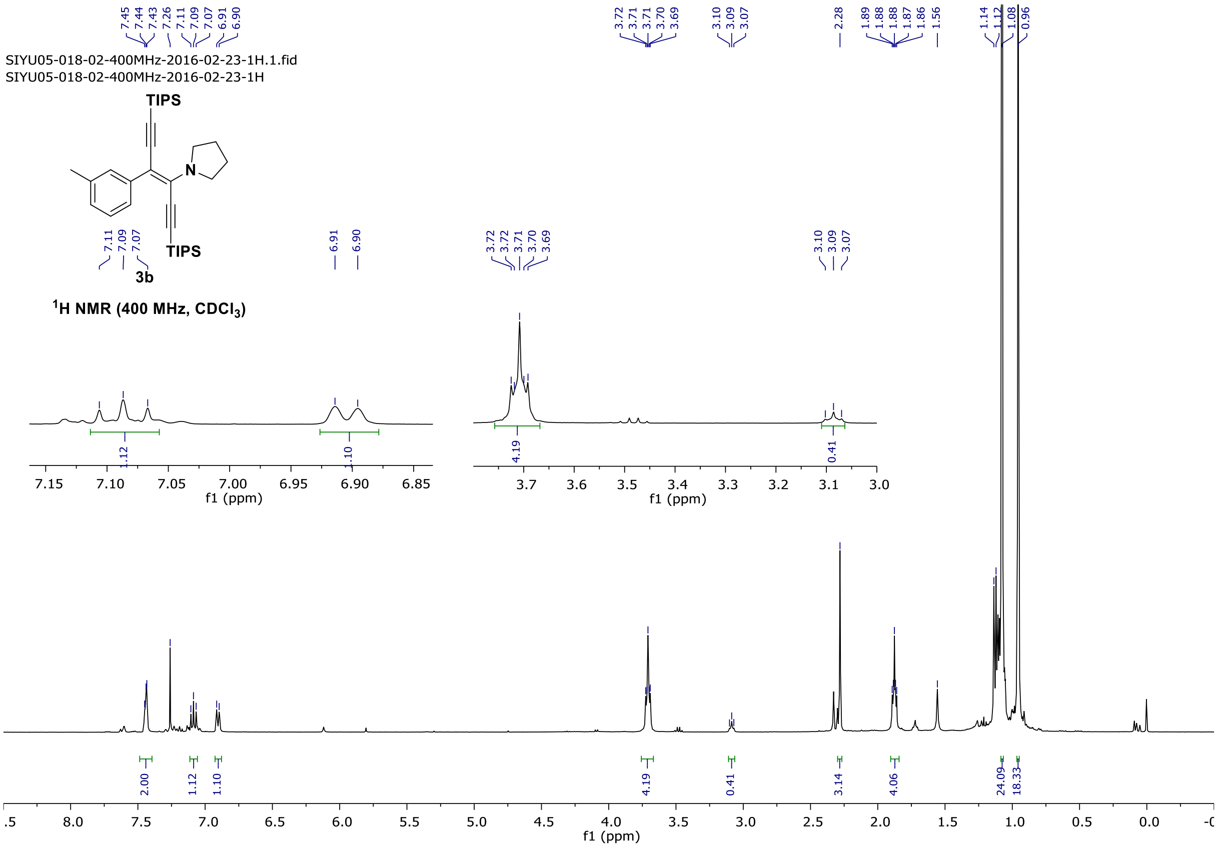

Supplementary Figure 3. ^{1}H NMR of the **3b** (400 MHz, $CDCl_3$)

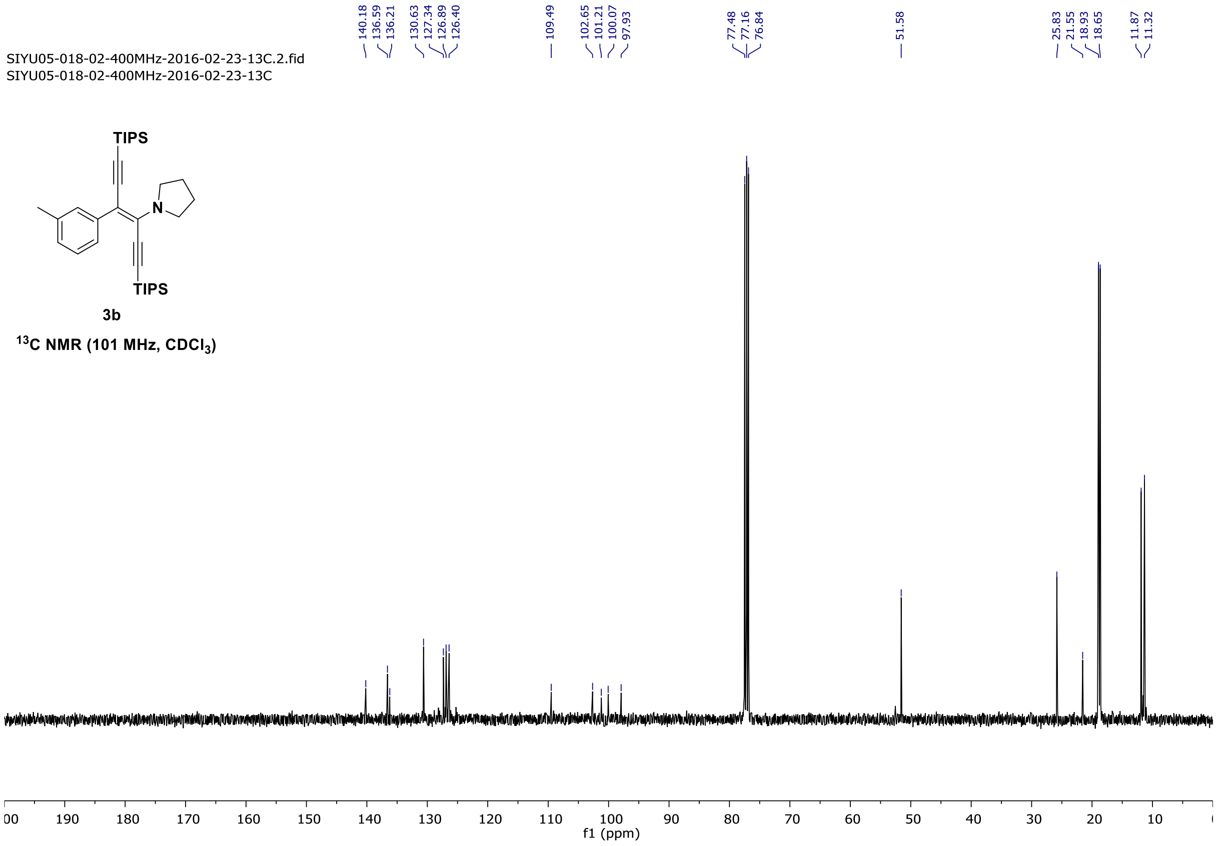

Supplementary Figure 4. ^{13}C NMR of the **3b** (101 MHz, $CDCl_3$)

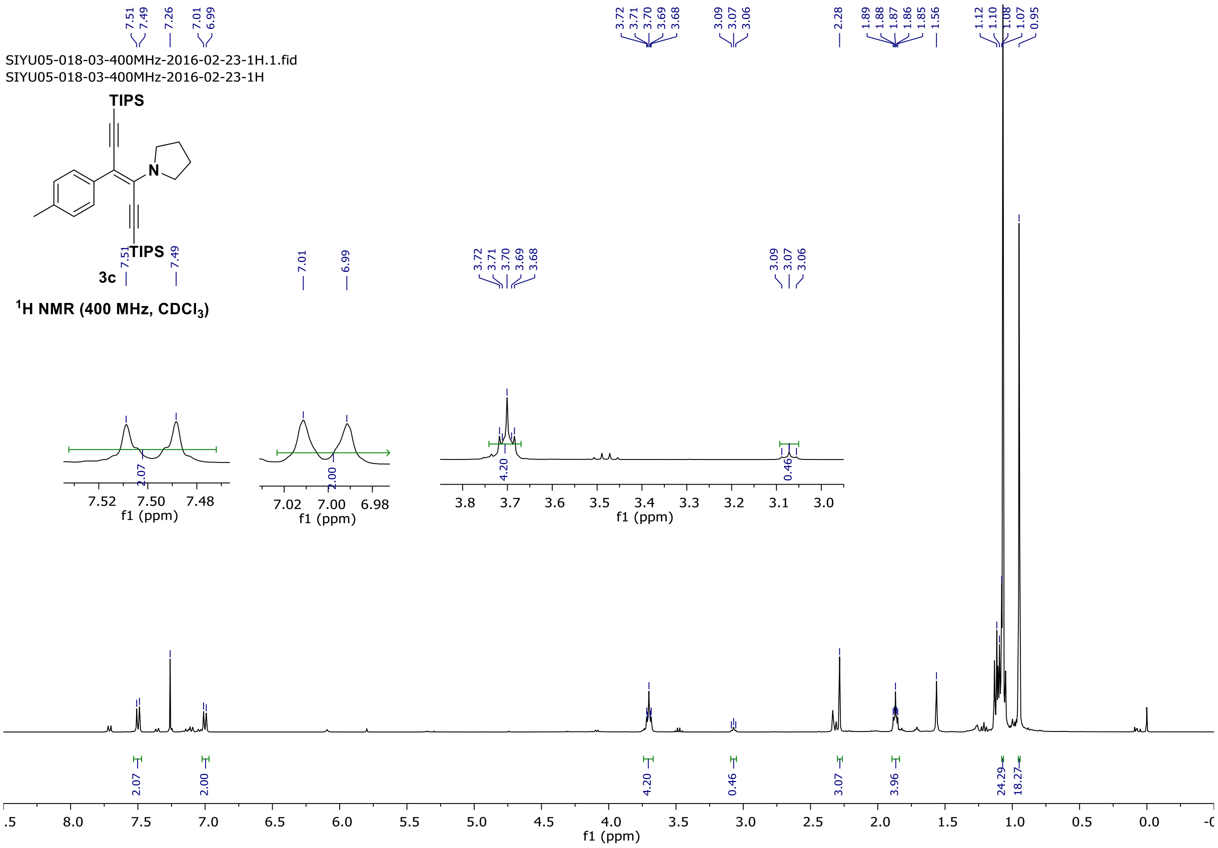

Supplementary Figure 5. ^{1}H NMR of the **3c** (400 MHz, $CDCl_3$)

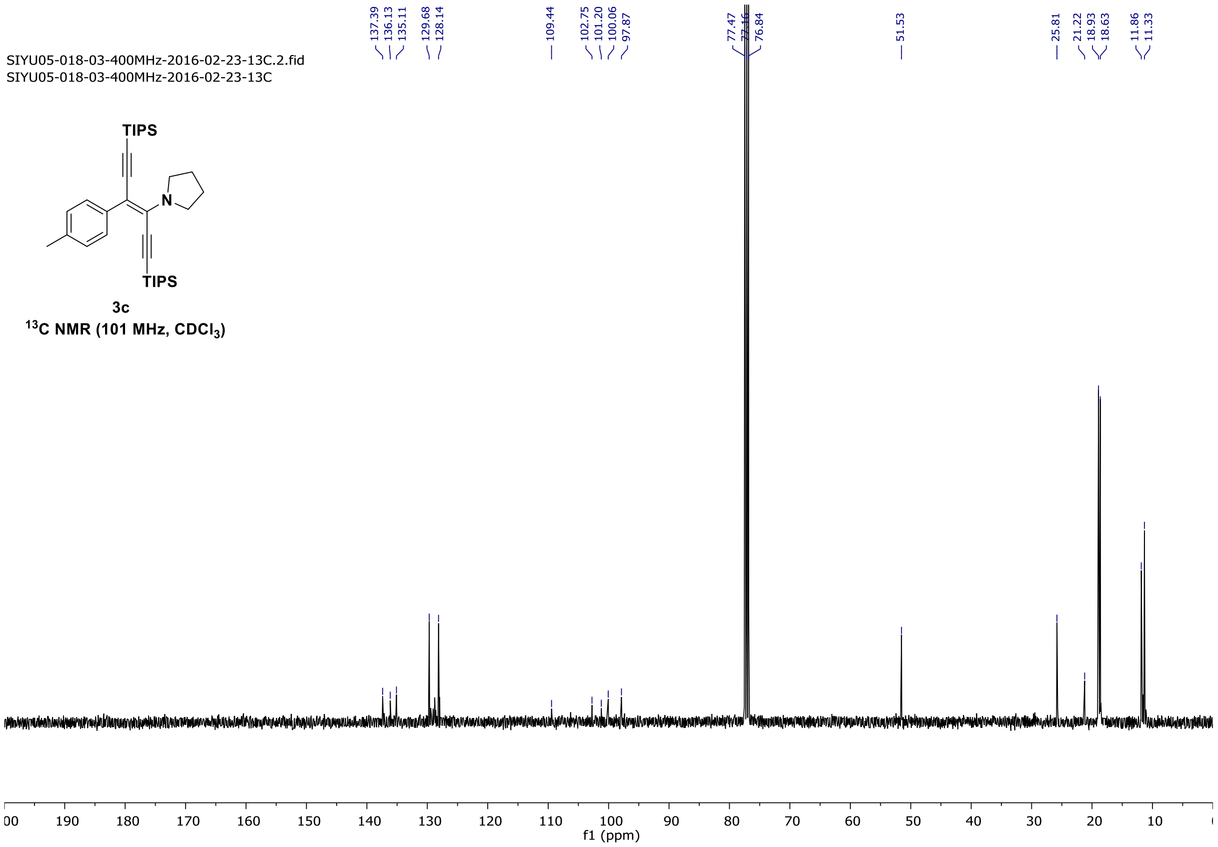

Supplementary Figure 6. ^{13}C NMR of the **3c** (101 MHz, $CDCl_3$)

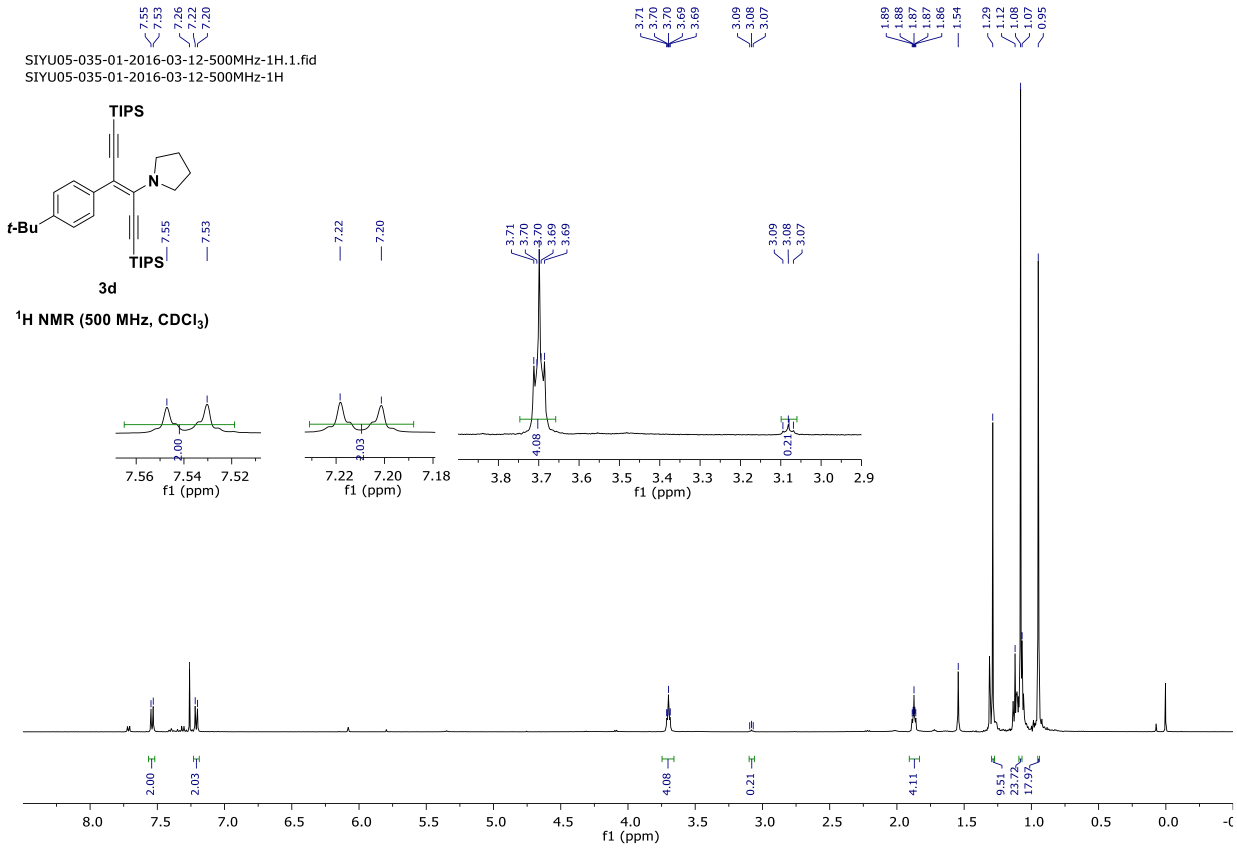

Supplementary Figure 7. ^{1}H NMR of the **3d** (500 MHz, $CDCl_3$)

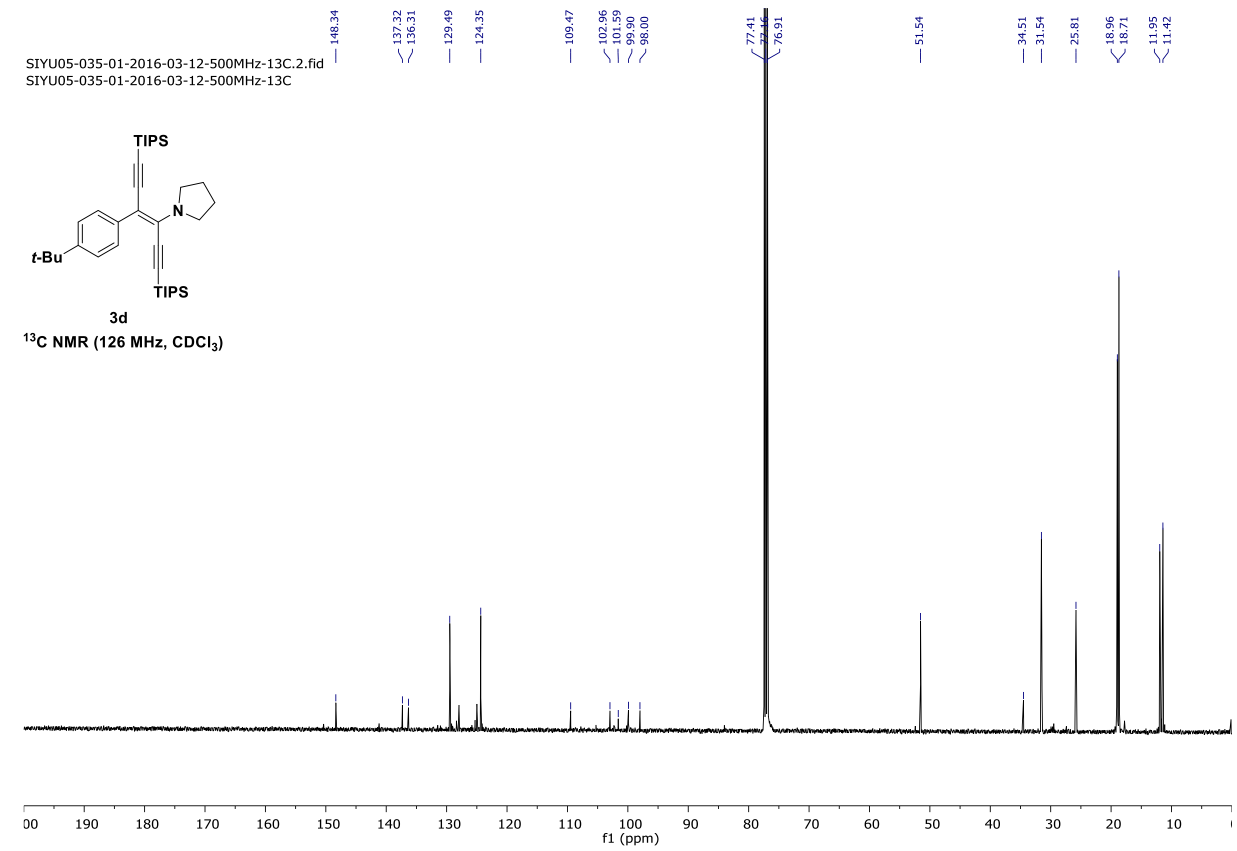

Supplementary Figure 8. ^{13}C NMR of the **3d** (126 MHz, $CDCl_3$)

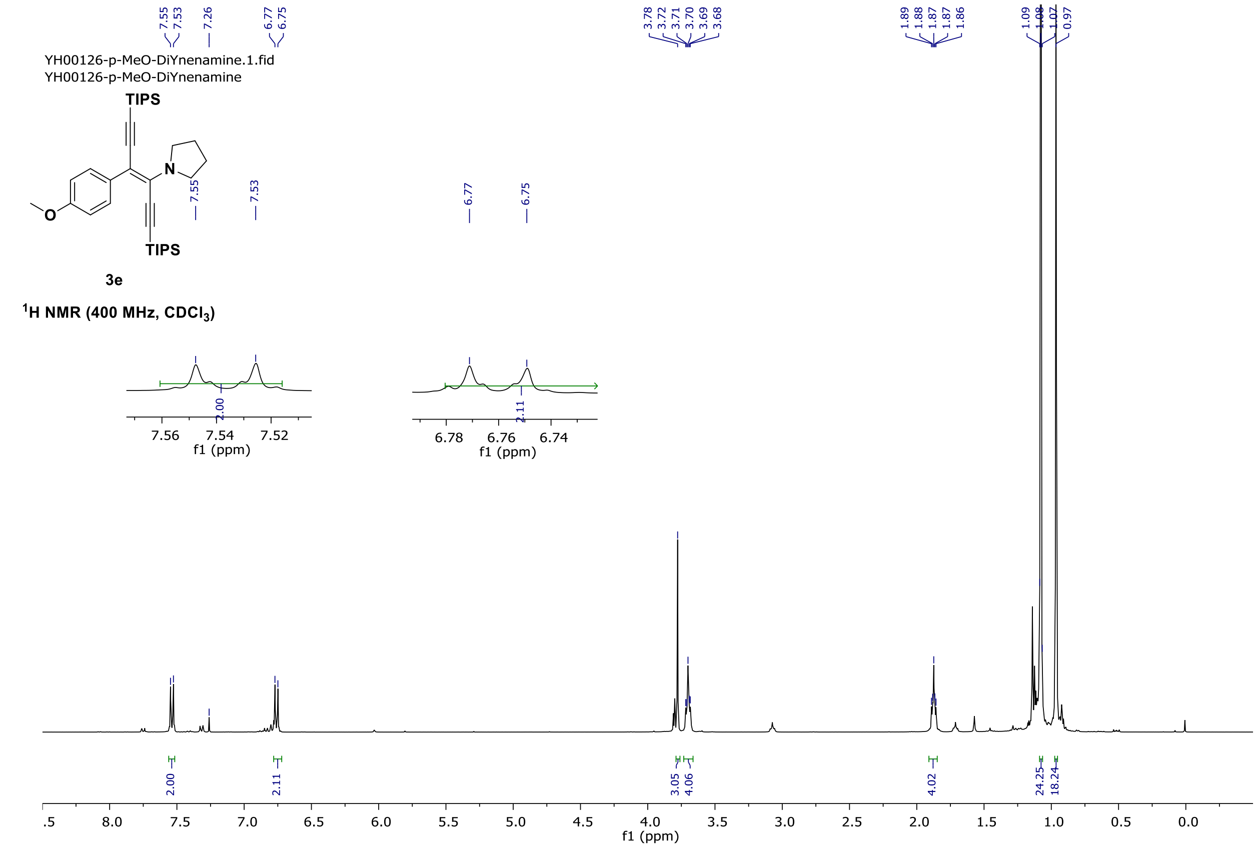

Supplementary Figure 9. ^{1}H NMR of the **3e** (400 MHz, $CDCl_3$)

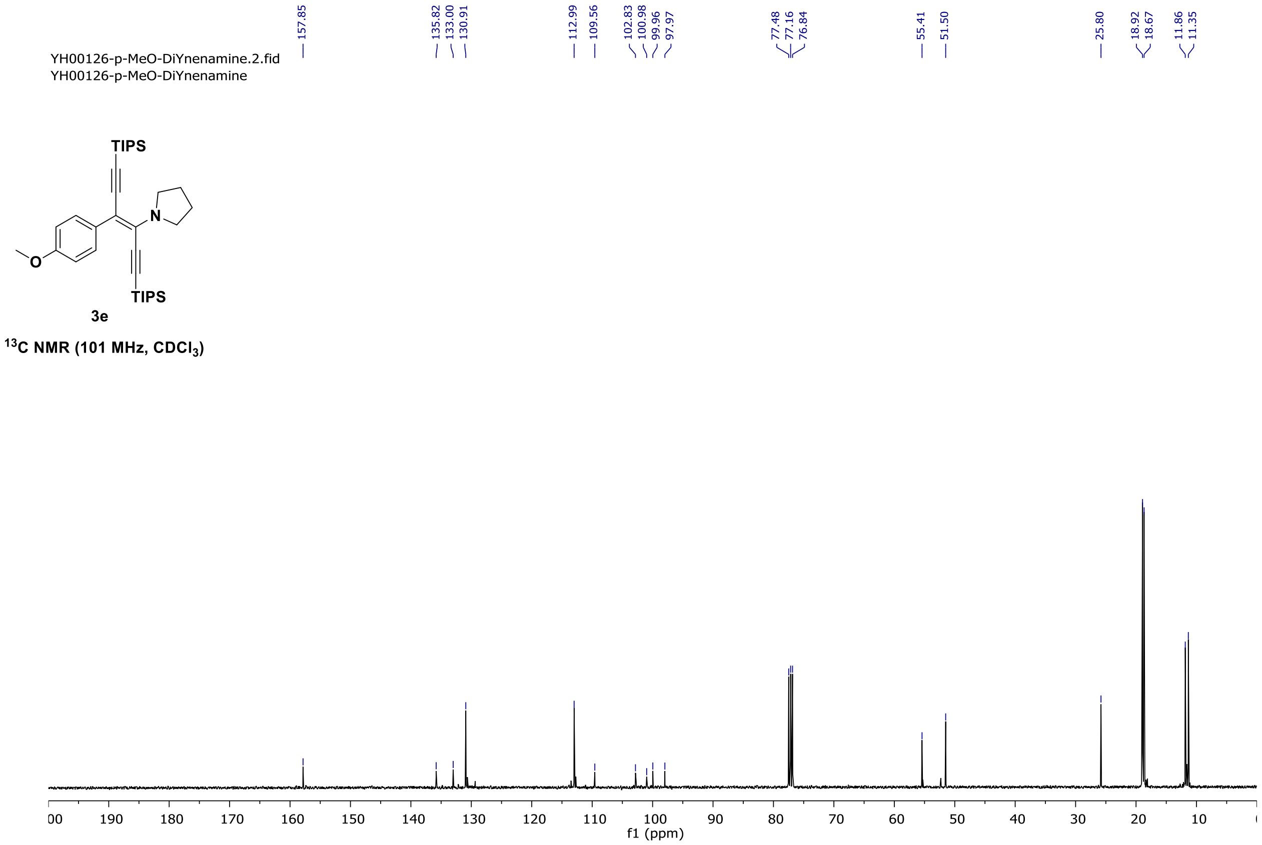

Supplementary Figure 10. ^{13}C NMR of the **3e** (101 MHz, $CDCl_3$)

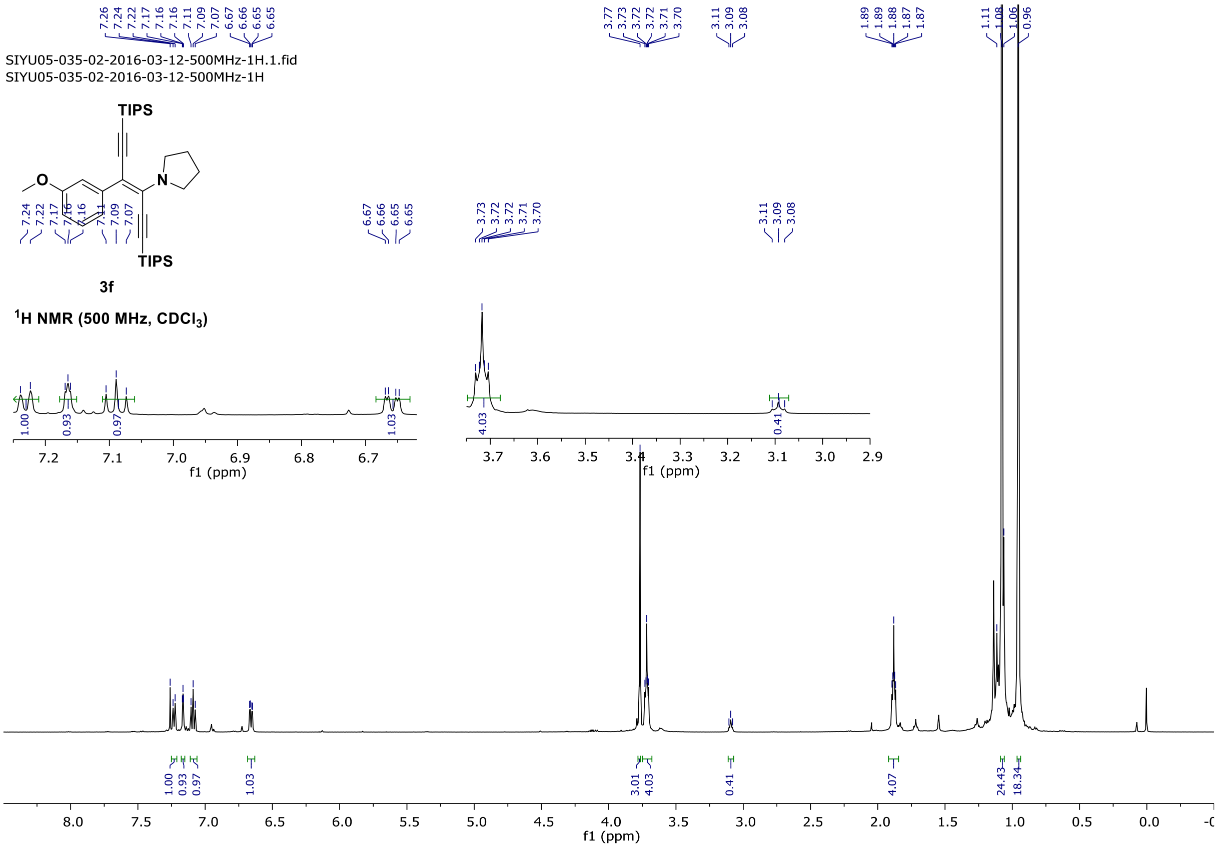

Supplementary Figure 11. ^{1}H NMR of the **3f** (500 MHz, $CDCl_3$)

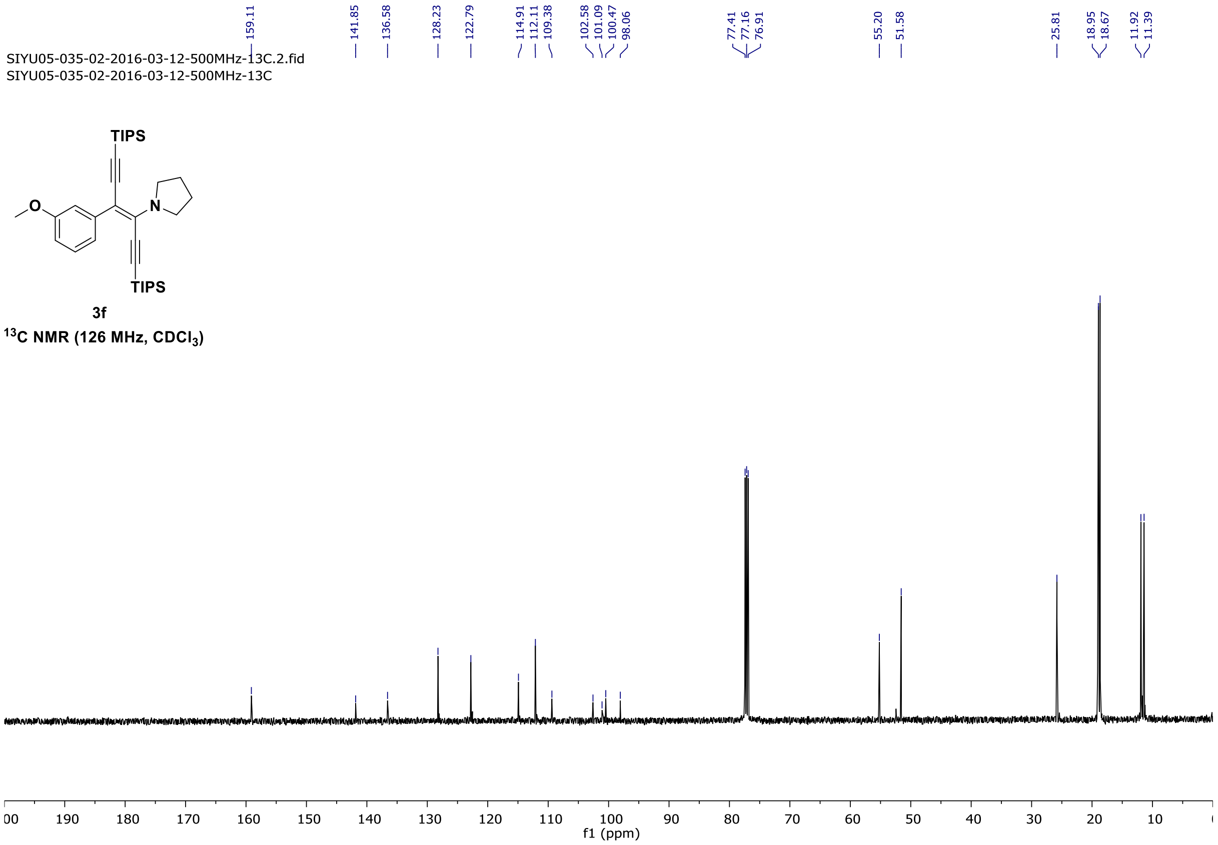

Supplementary Figure 12. ^{13}C NMR of the **3f** (126 MHz, $CDCl_3$)

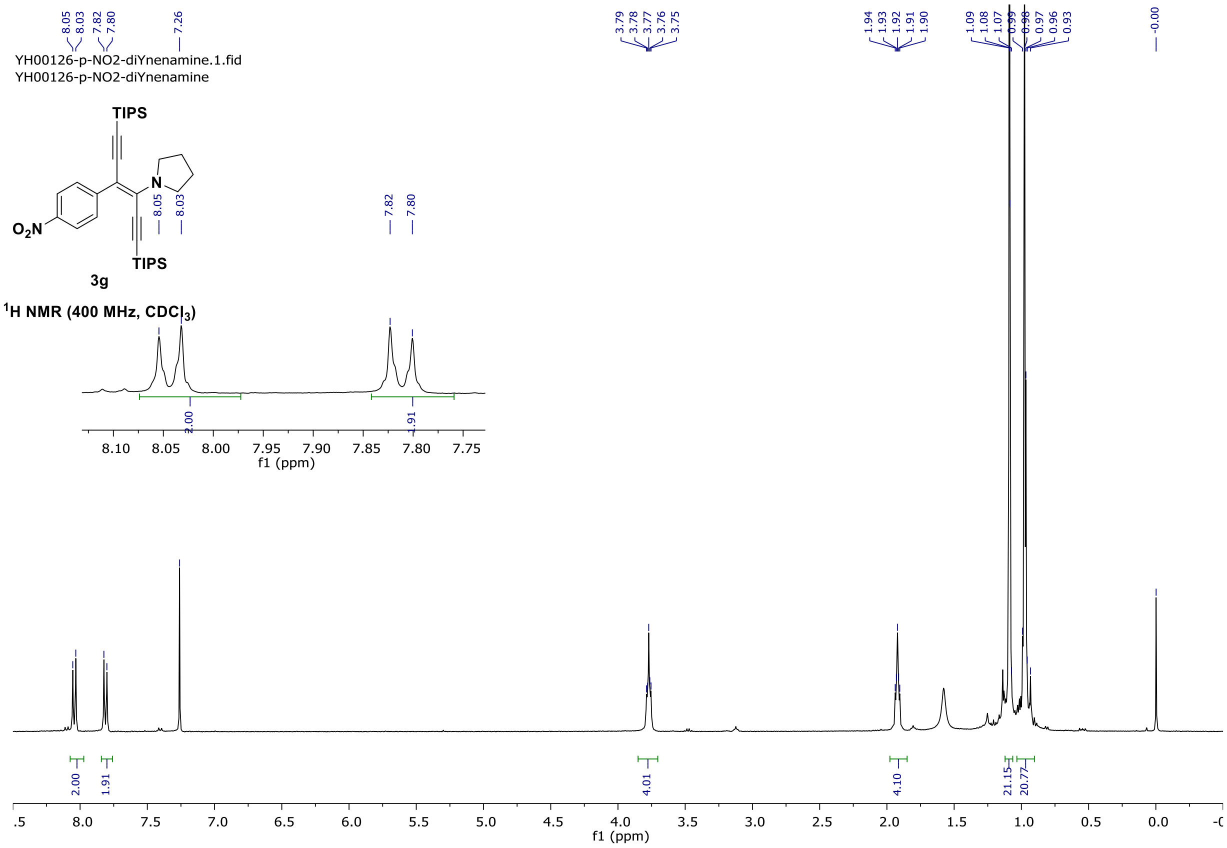

Supplementary Figure 13. ^{1}H NMR of the **3g** (400 MHz, $CDCl_3$)

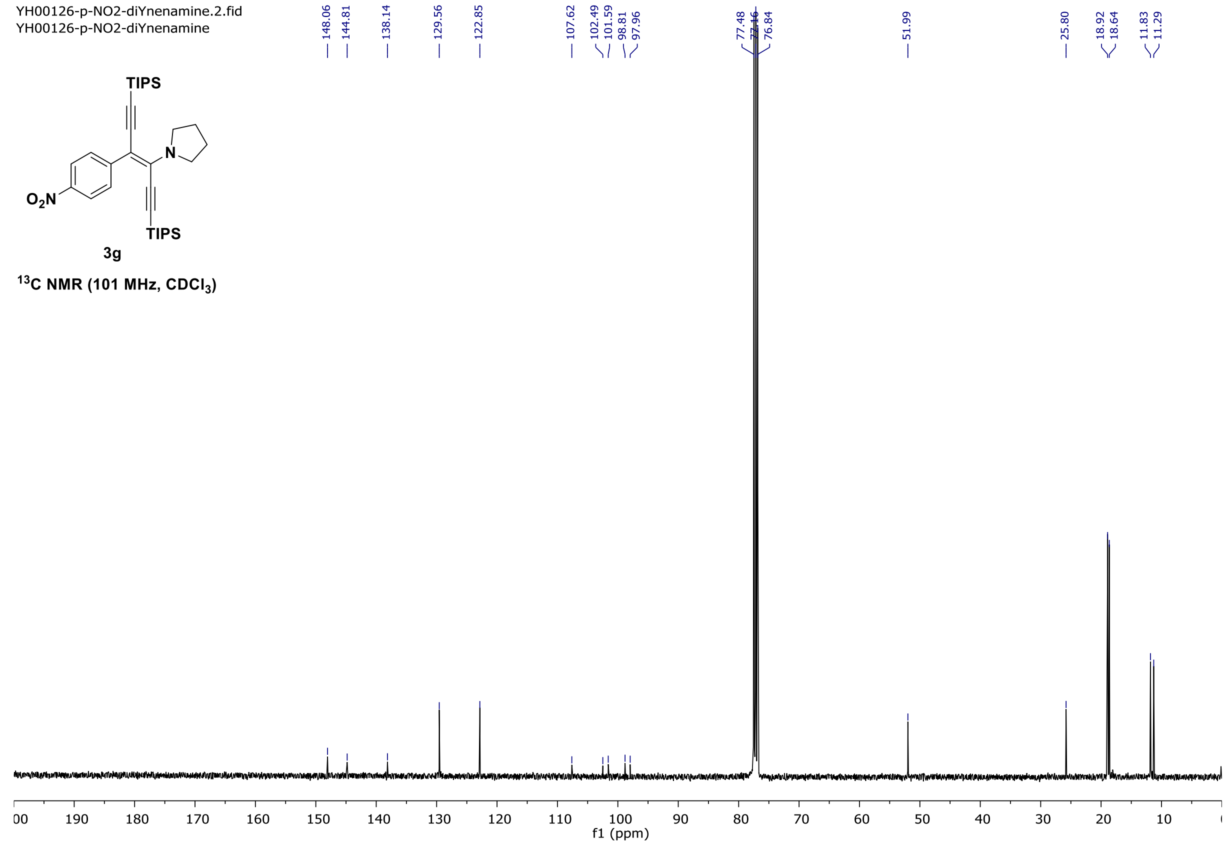

Supplementary Figure 14. ^{13}C NMR of the **3g** (101 MHz, $CDCl_3$)

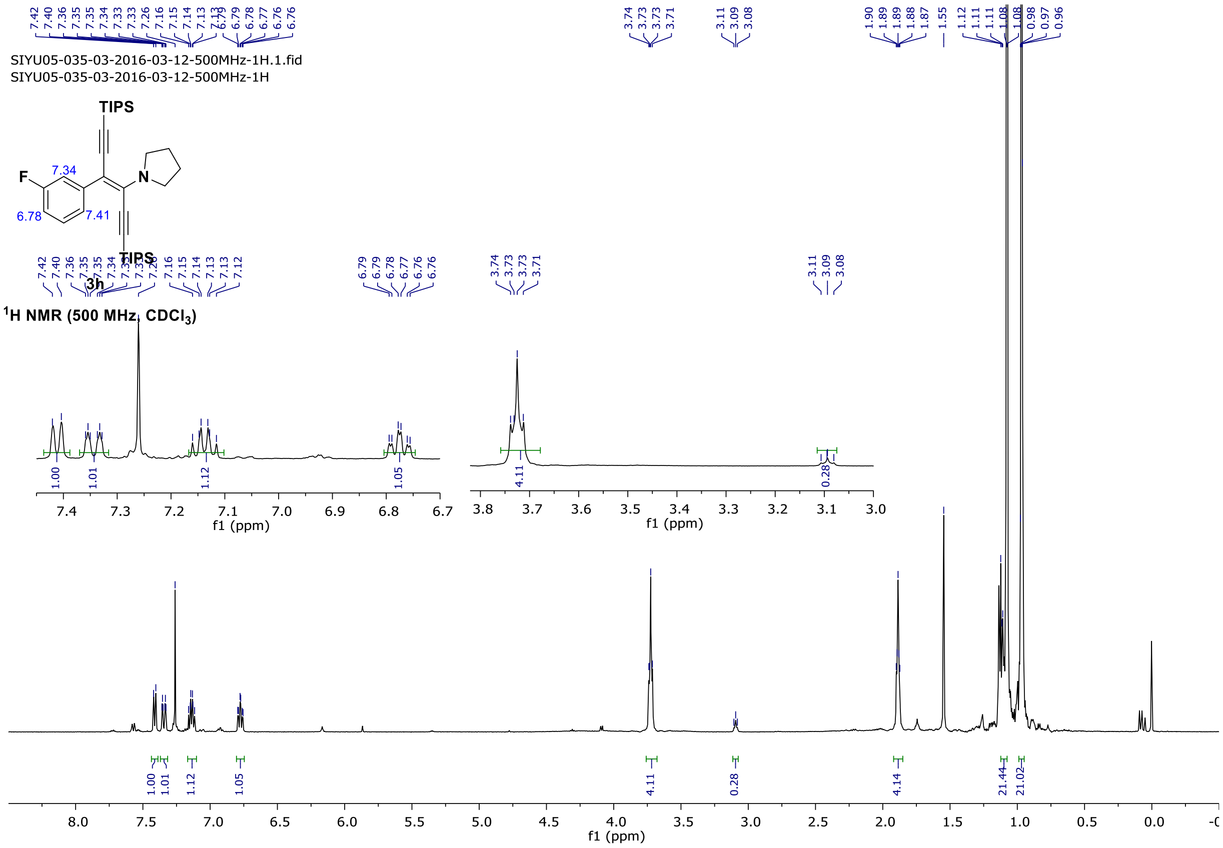

Supplementary Figure 15. ^{1}H NMR of the **3h** (500 MHz, $CDCl_3$)

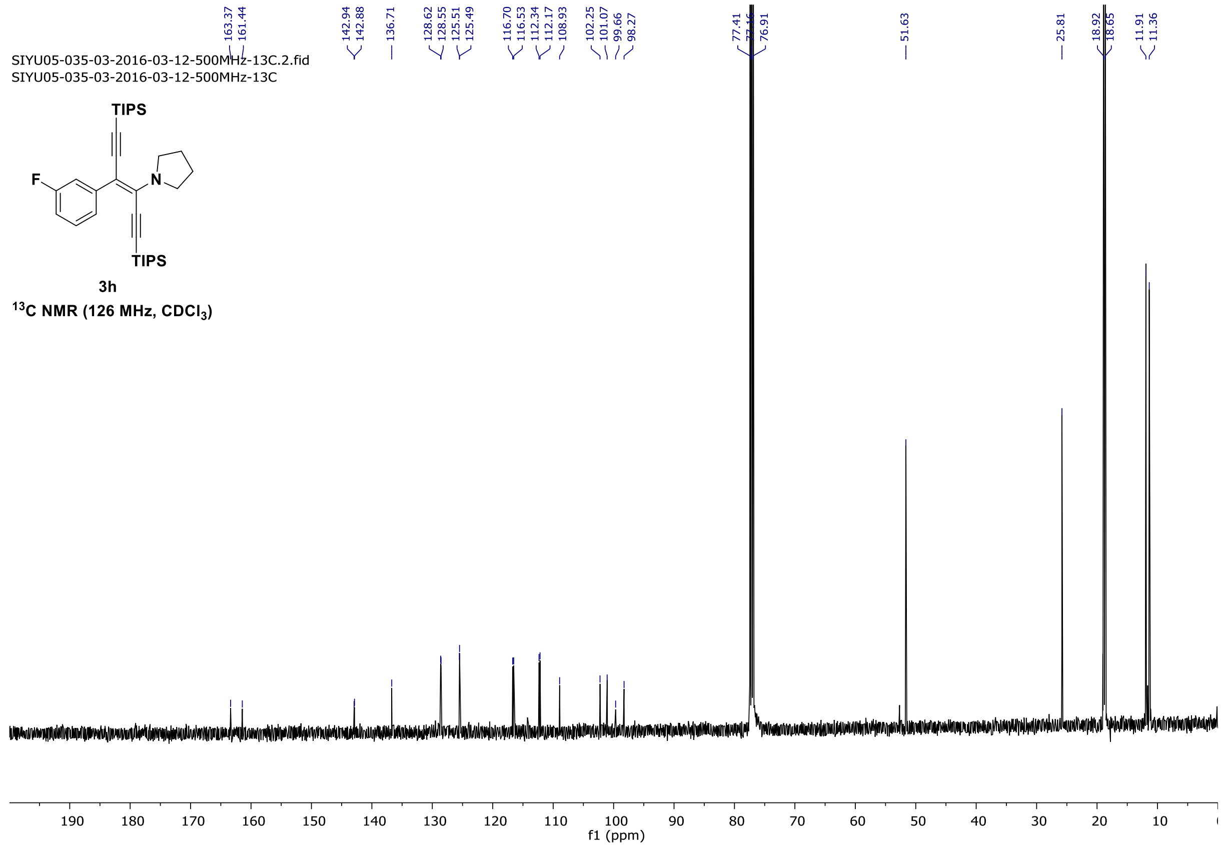

Supplementary Figure 16. ^{13}C NMR of the **3h** (126 MHz, $CDCl_3$)

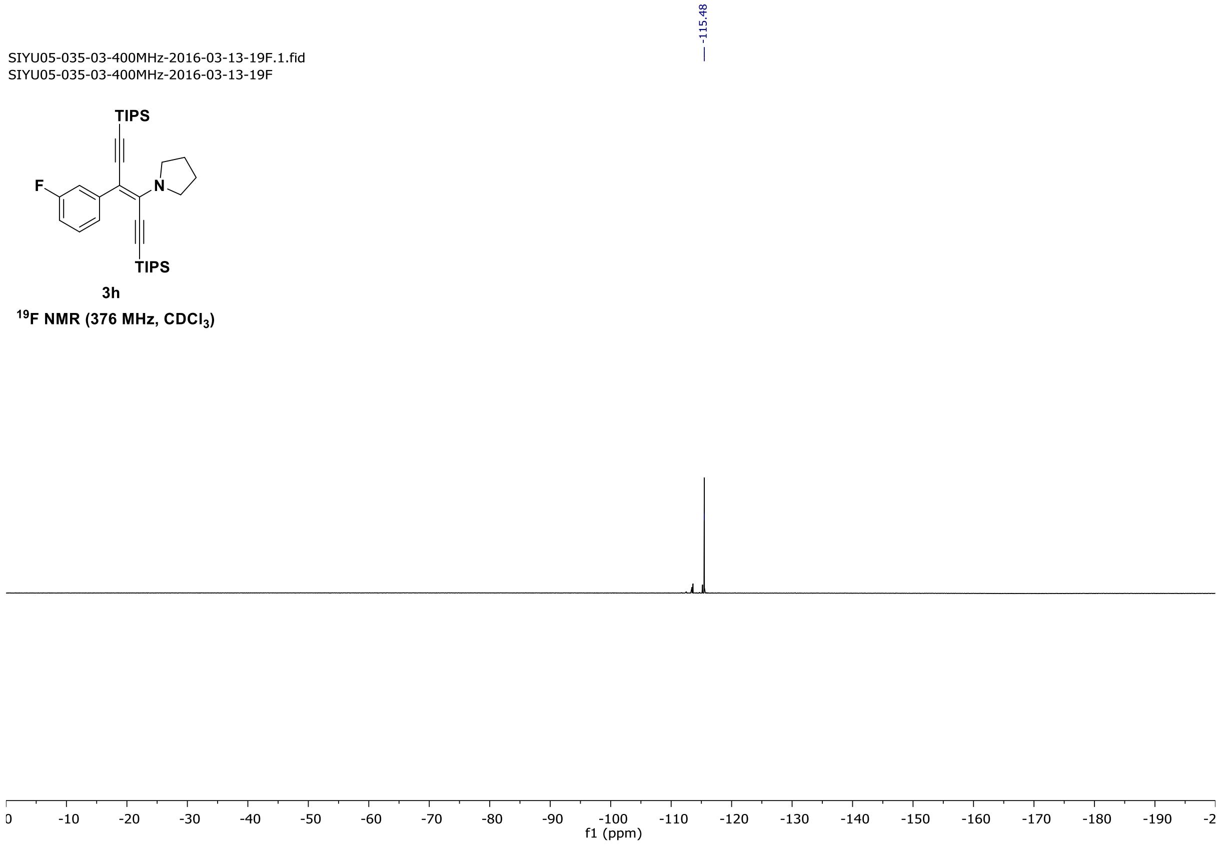

Supplementary Figure 17. ^{19}F NMR of the **3h** (376 MHz, $CDCl_3$)

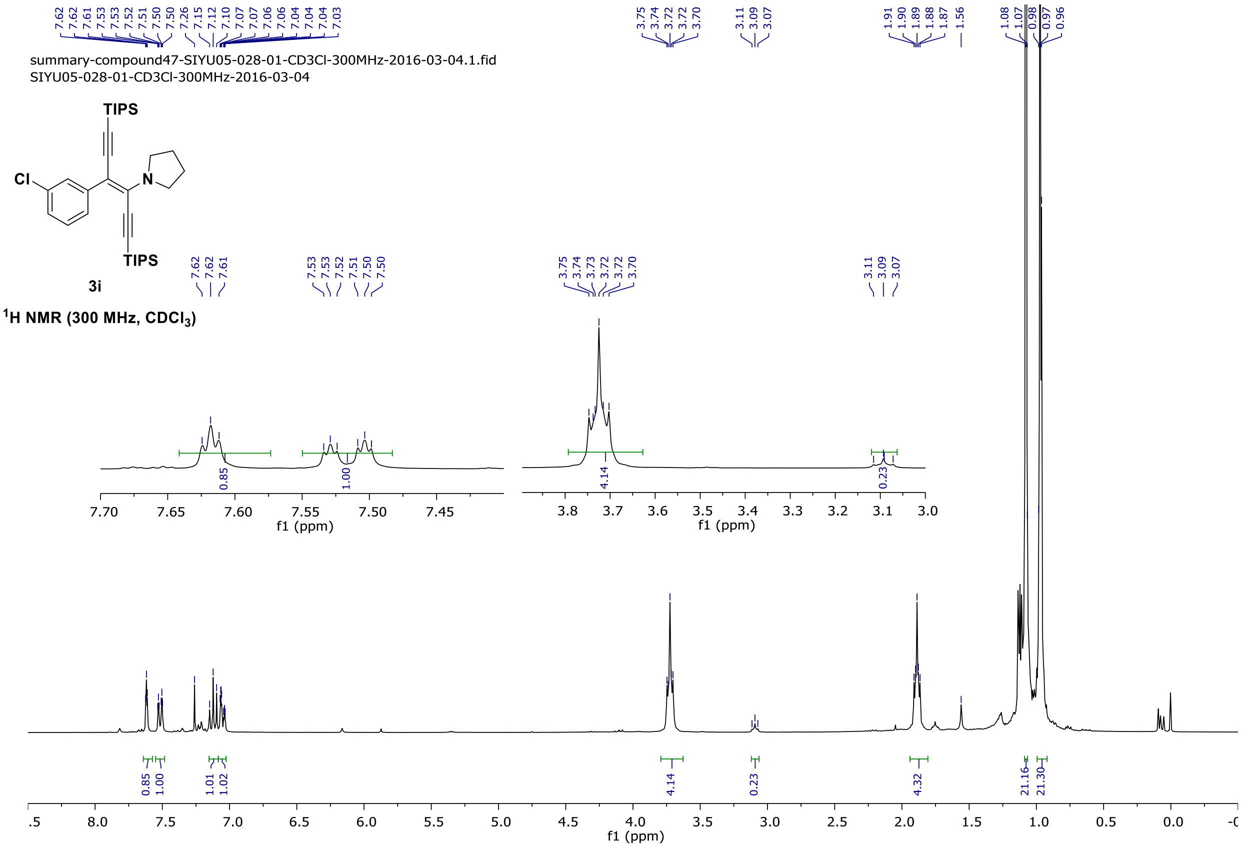

Supplementary Figure 18. ^{1}H NMR of the **3i** (300 MHz, $CDCl_3$)

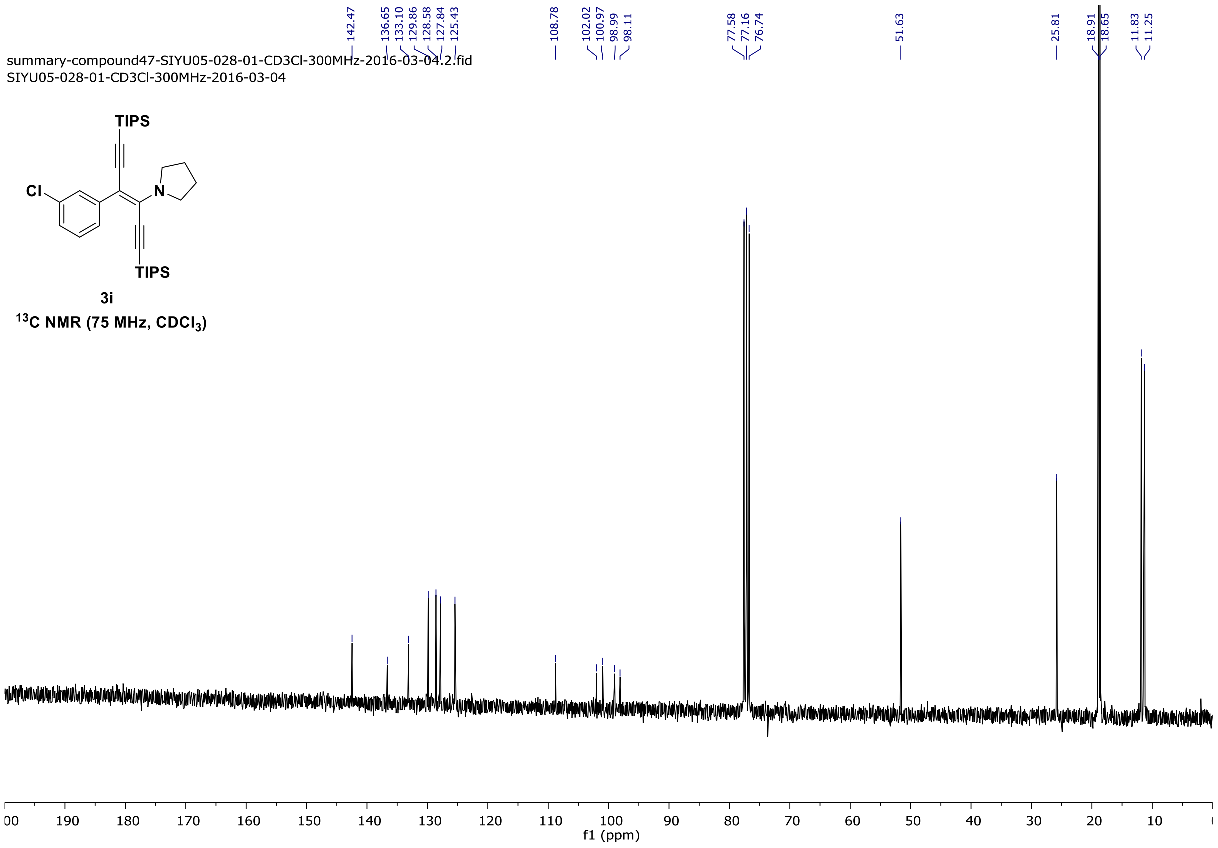

Supplementary Figure 19. ^{13}C NMR of the **3i** (75 MHz, $CDCl_3$)

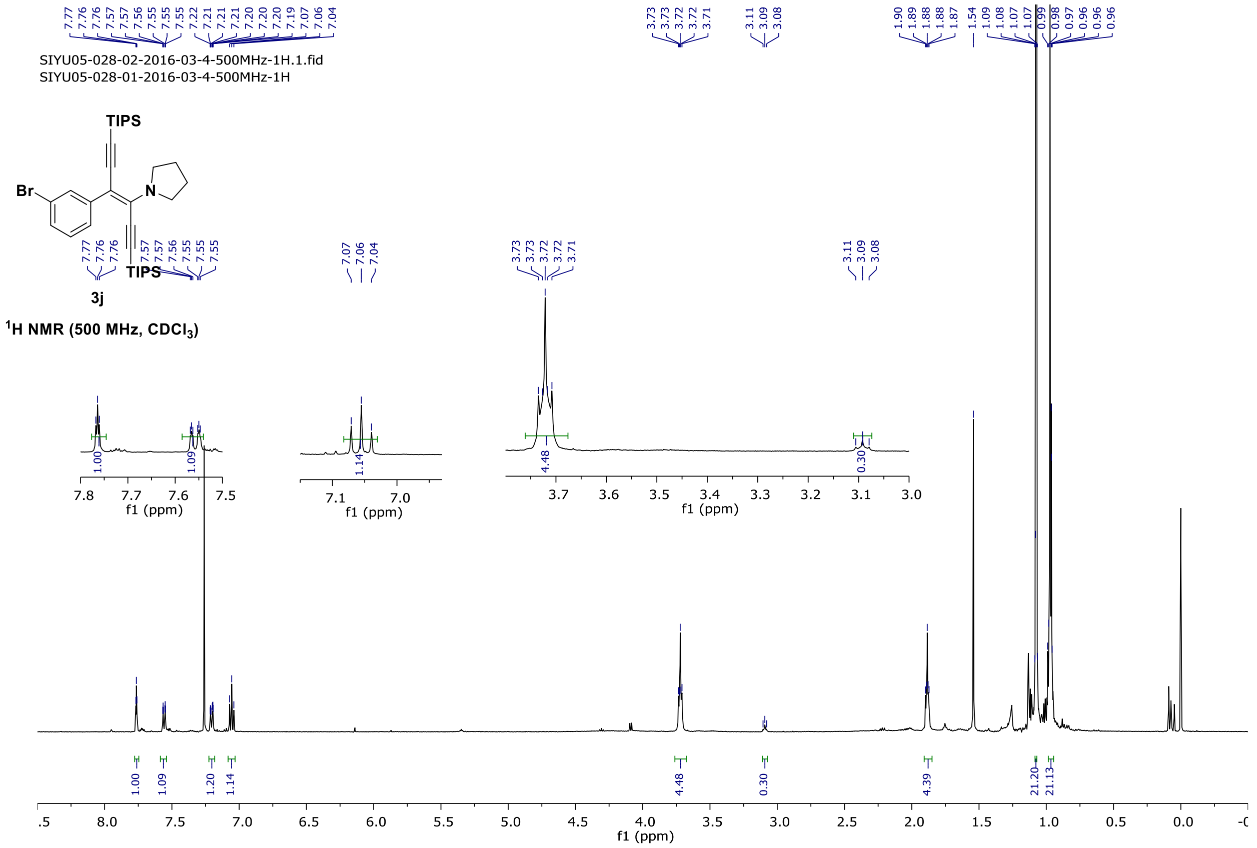

Supplementary Figure 20. ^{1}H NMR of the **3j** (500 MHz, $CDCl_3$)

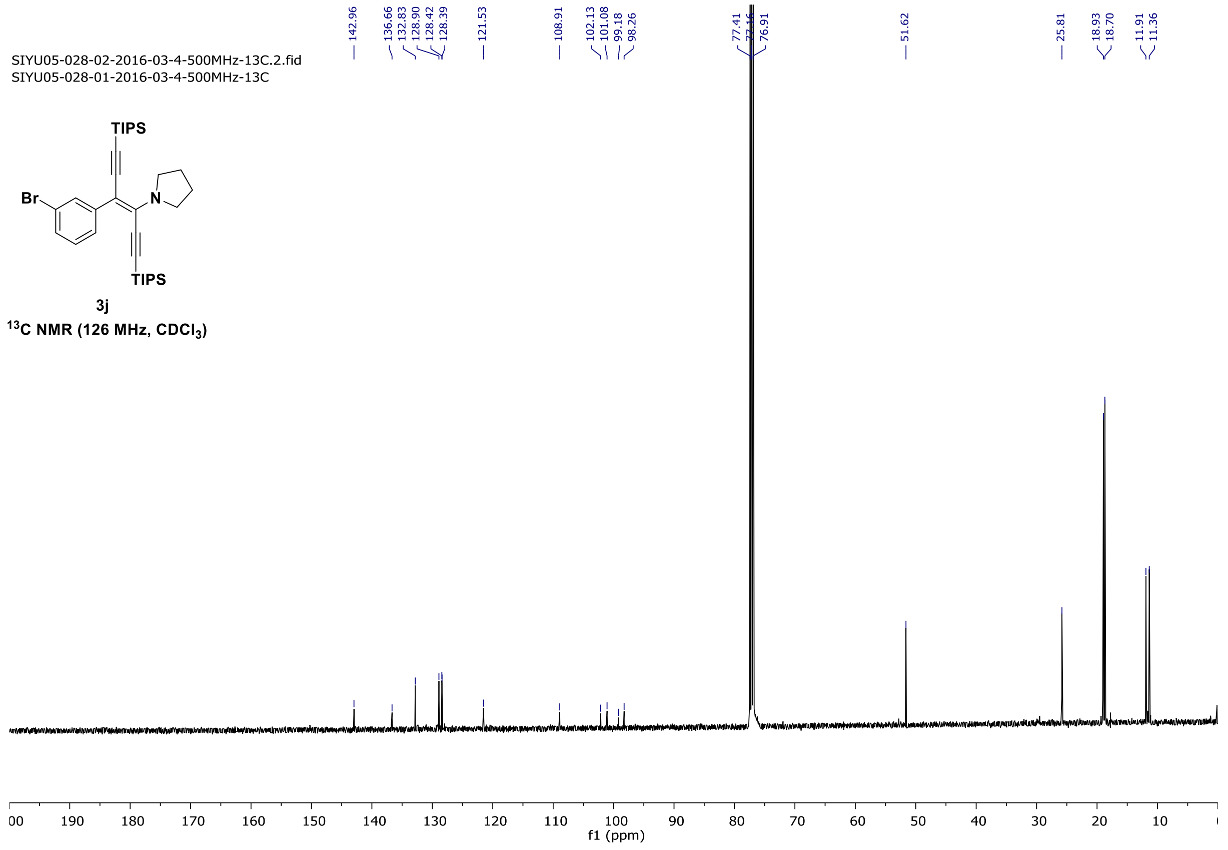

Supplementary Figure 21. ^{13}C NMR of the **3j** (126 MHz, $CDCl_3$)

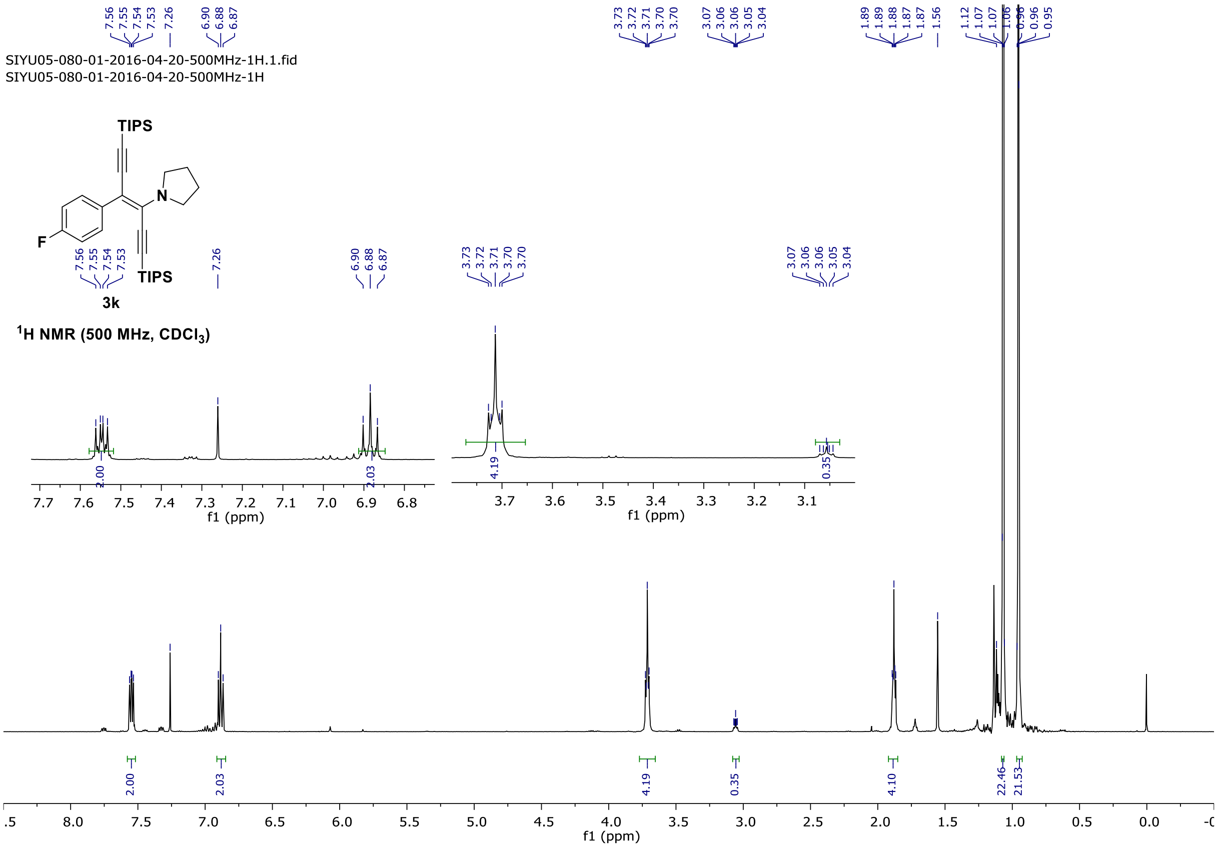

Supplementary Figure 22. ^{1}H NMR of the **3k** (500 MHz, $CDCl_3$)

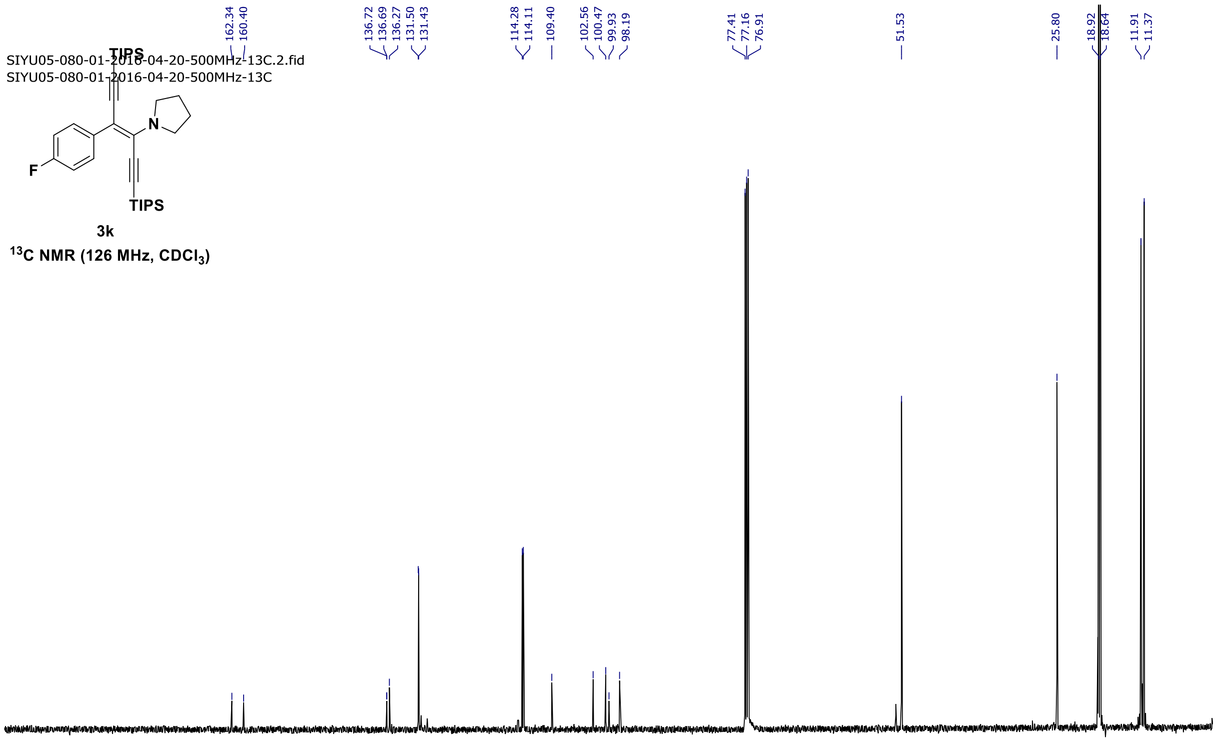

Supplementary Figure 23. ^{13}C NMR of the **3k** (126 MHz, $CDCl_3$)

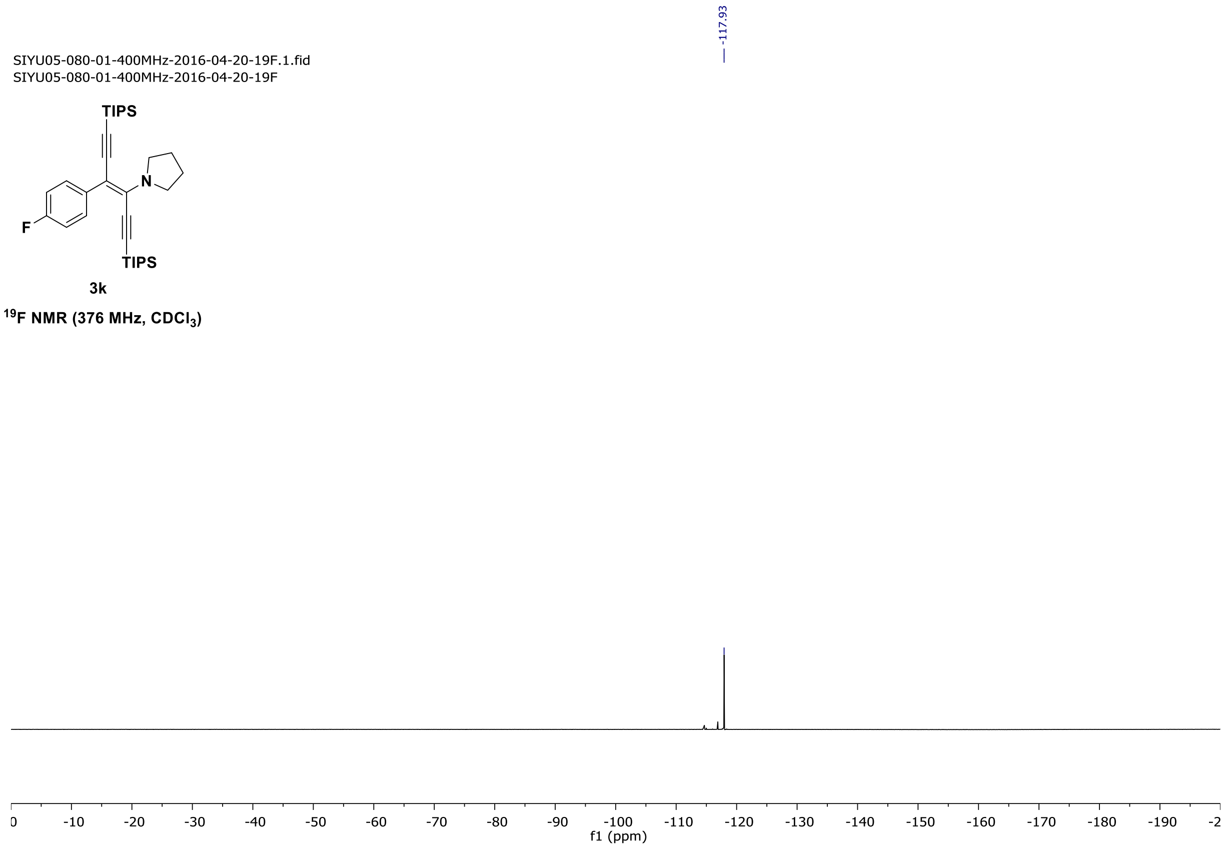

Supplementary Figure 24. ^{19}F NMR of the **3k** (376 MHz, $CDCl_3$)

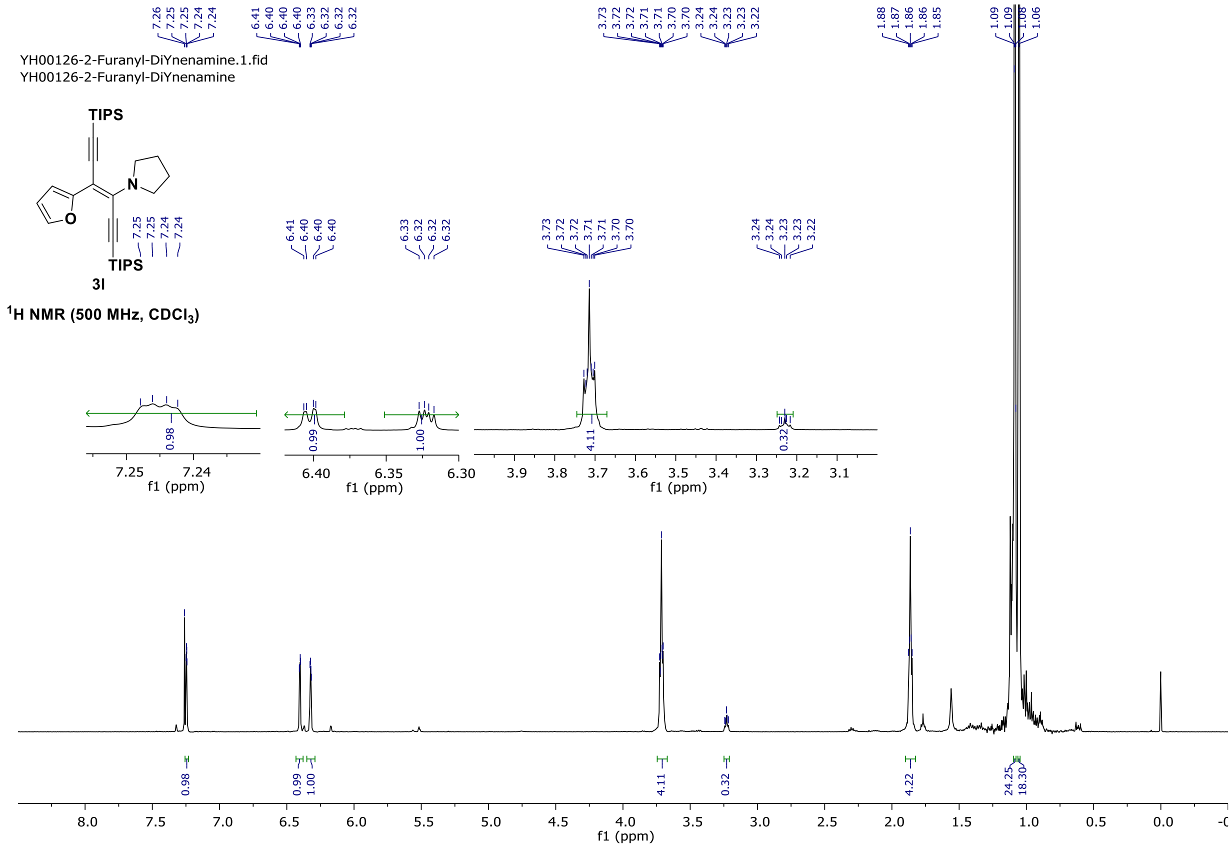

Supplementary Figure 25. ^{1}H NMR of the **3l** (500 MHz, $CDCl_3$)

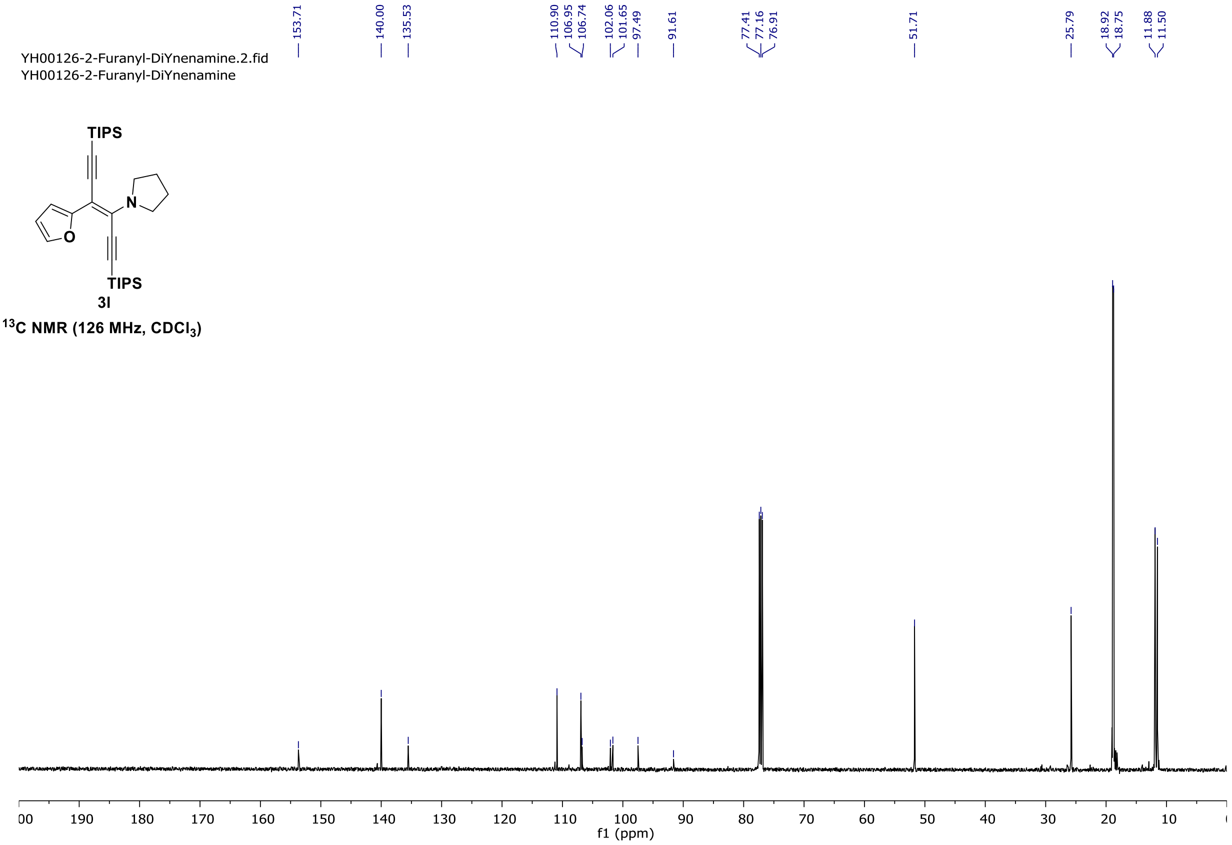

Supplementary Figure 26. ^{13}C NMR of the **3l** (126 MHz, $CDCl_3$)

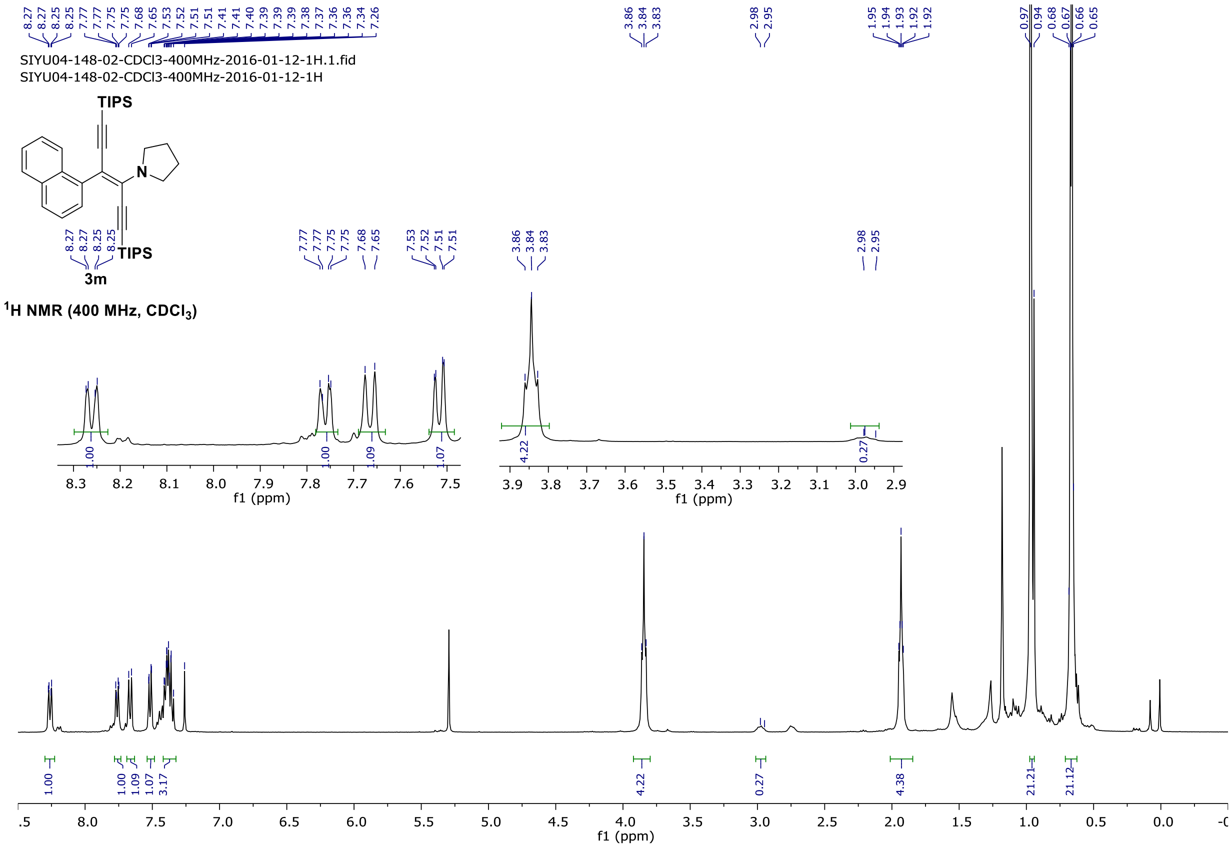

Supplementary Figure 27. ^{1}H NMR of the **3m** (400 MHz, $CDCl_3$)

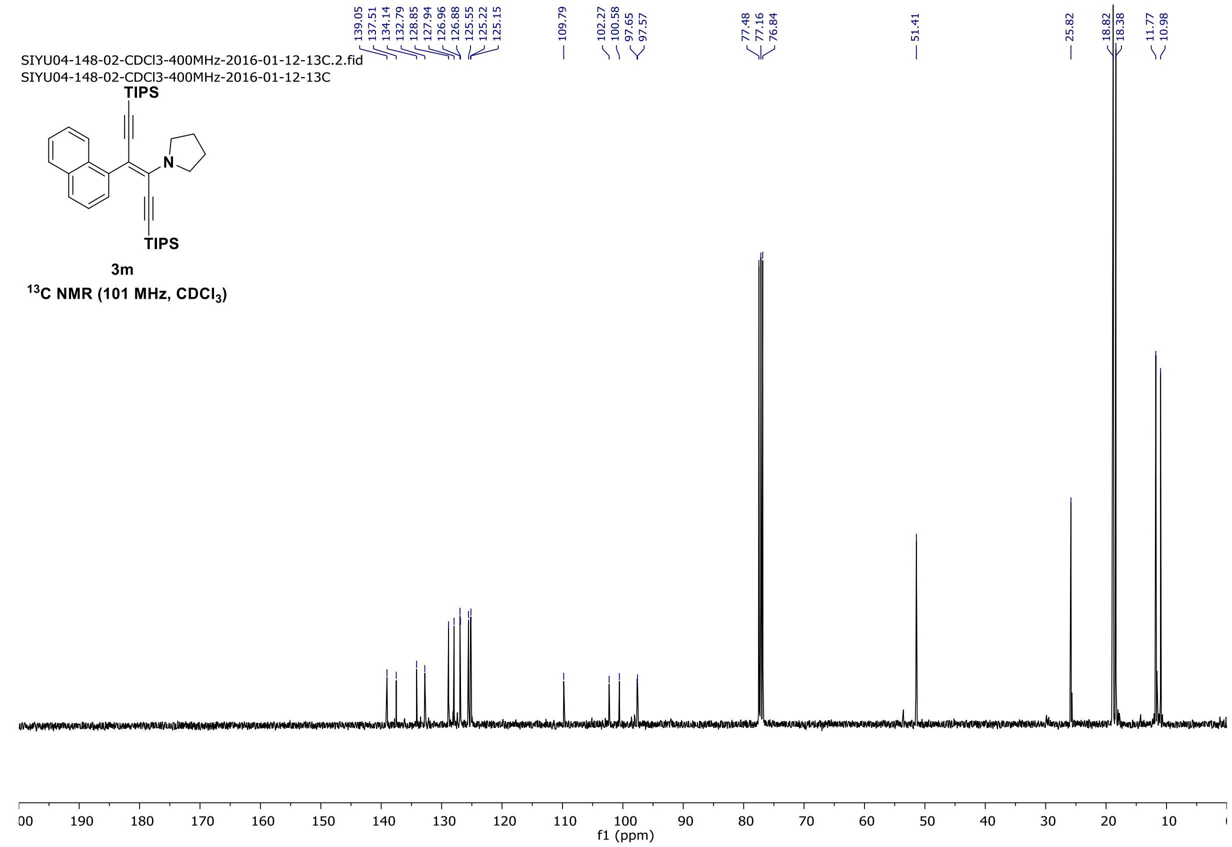

Supplementary Figure 28. ^{13}C NMR of the **3m** (101 MHz, $CDCl_3$)

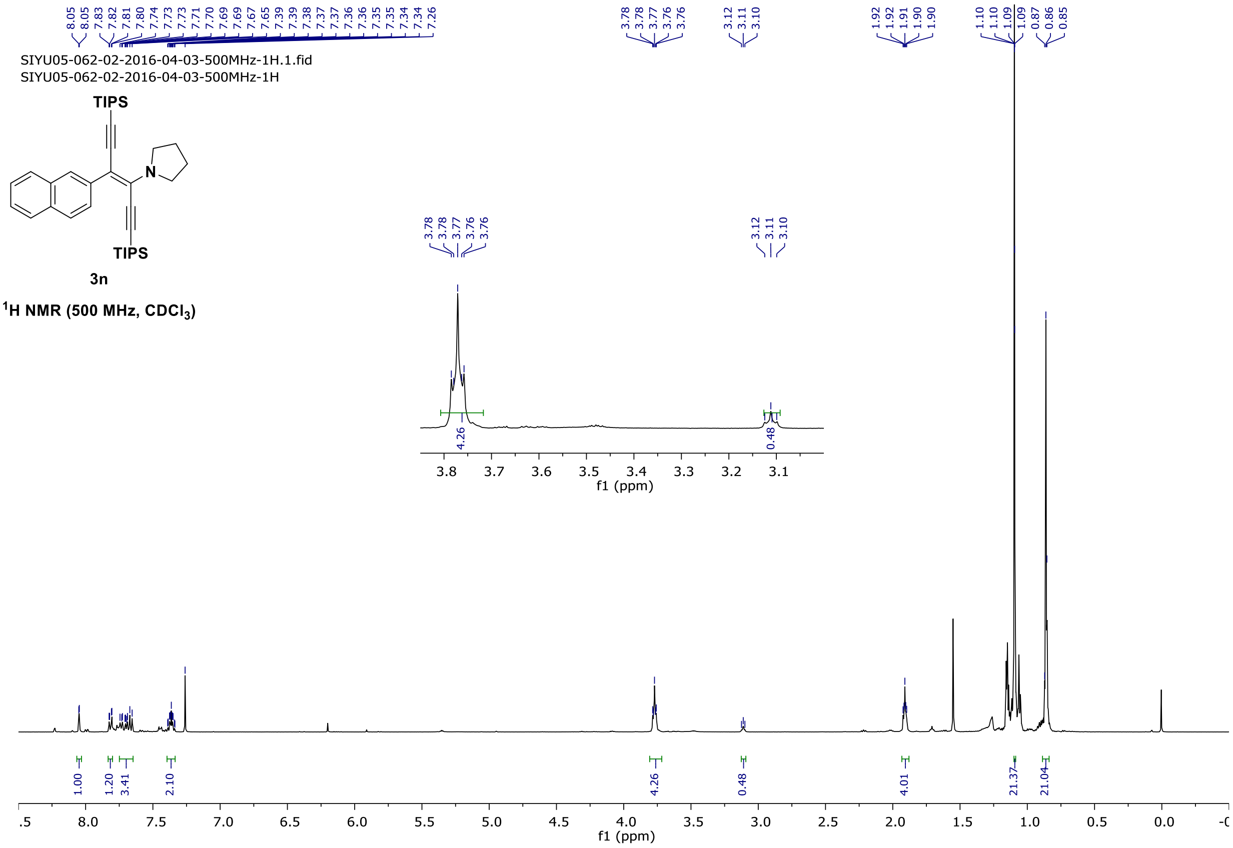

Supplementary Figure 29. ^{1}H NMR of the **3n** (500 MHz, $CDCl_3$)

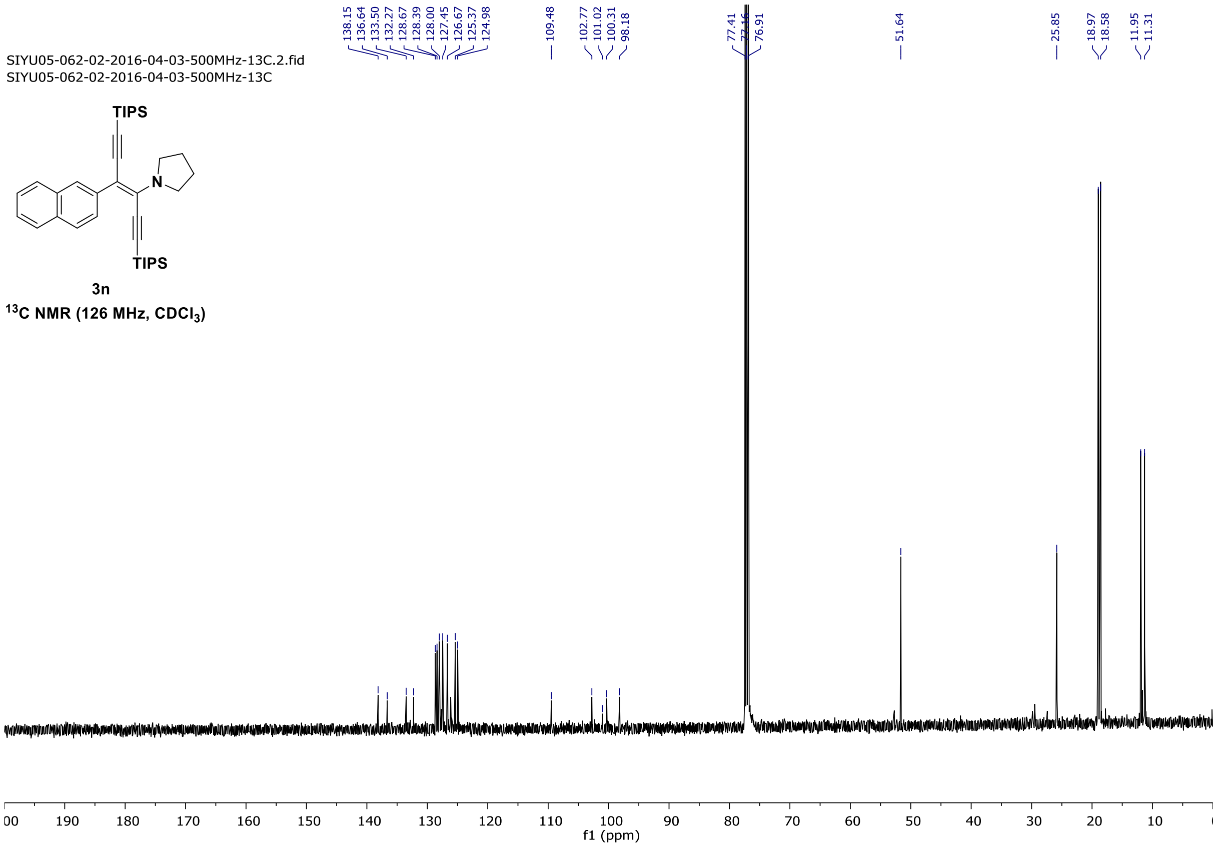

Supplementary Figure 30. ^{13}C NMR of the **3n** (126 MHz, $CDCl_3$)

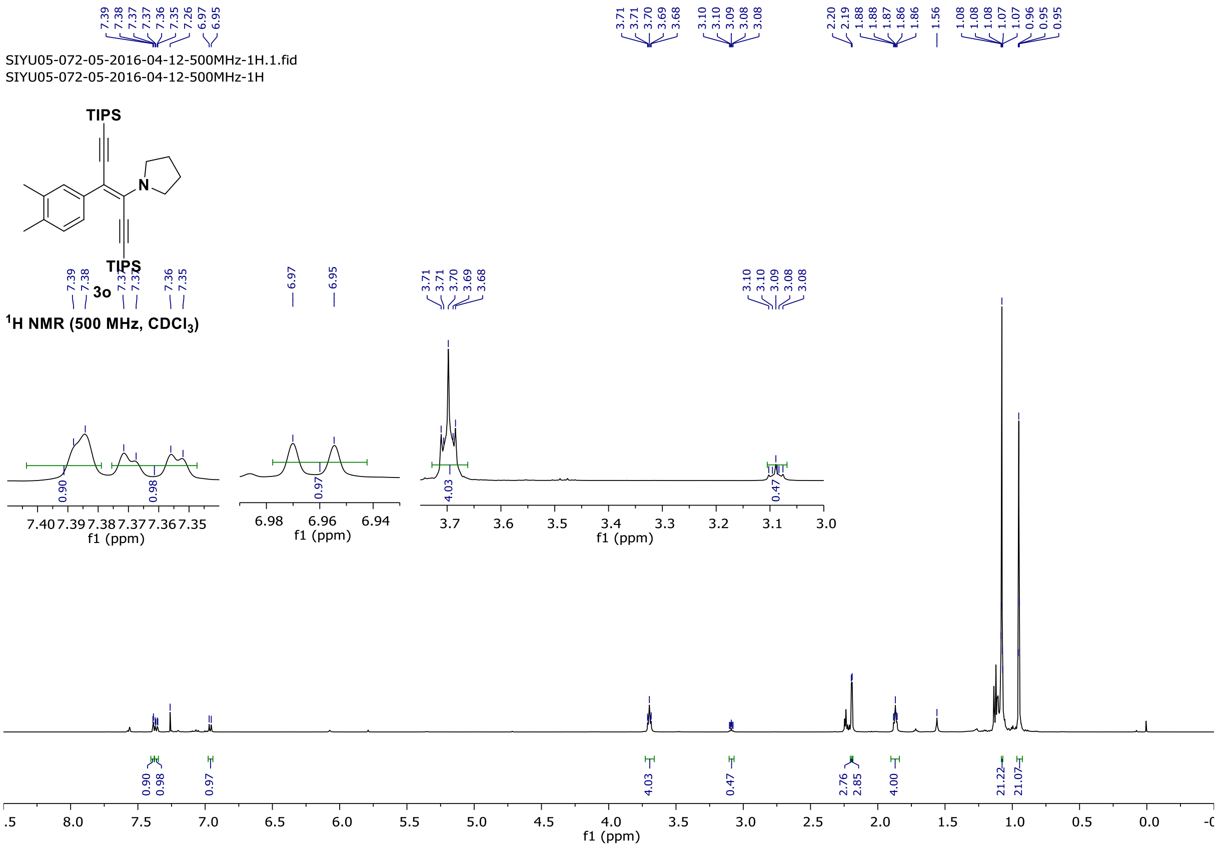

Supplementary Figure 31. ^{1}H NMR of the **3o** (500 MHz, $CDCl_3$)

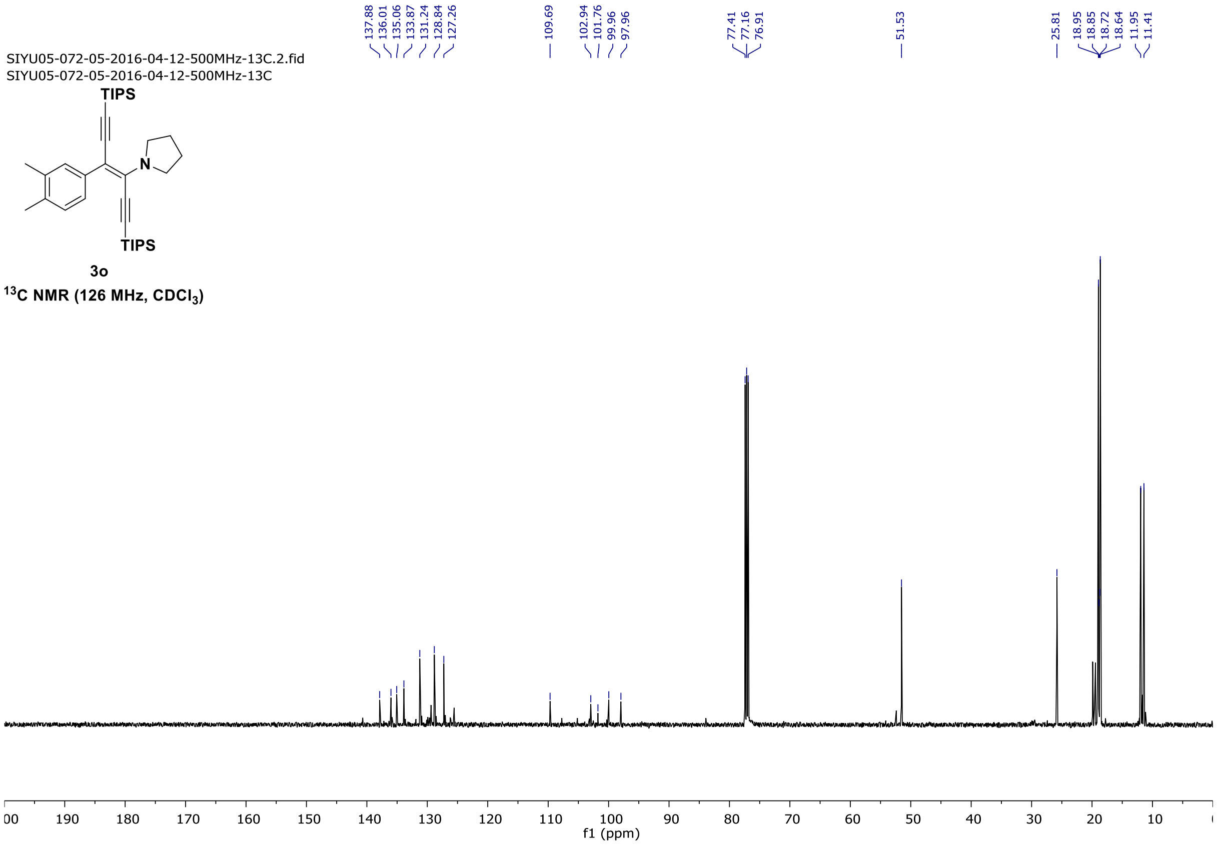

Supplementary Figure 32. ^{13}C NMR of the **3o** (126 MHz, $CDCl_3$)

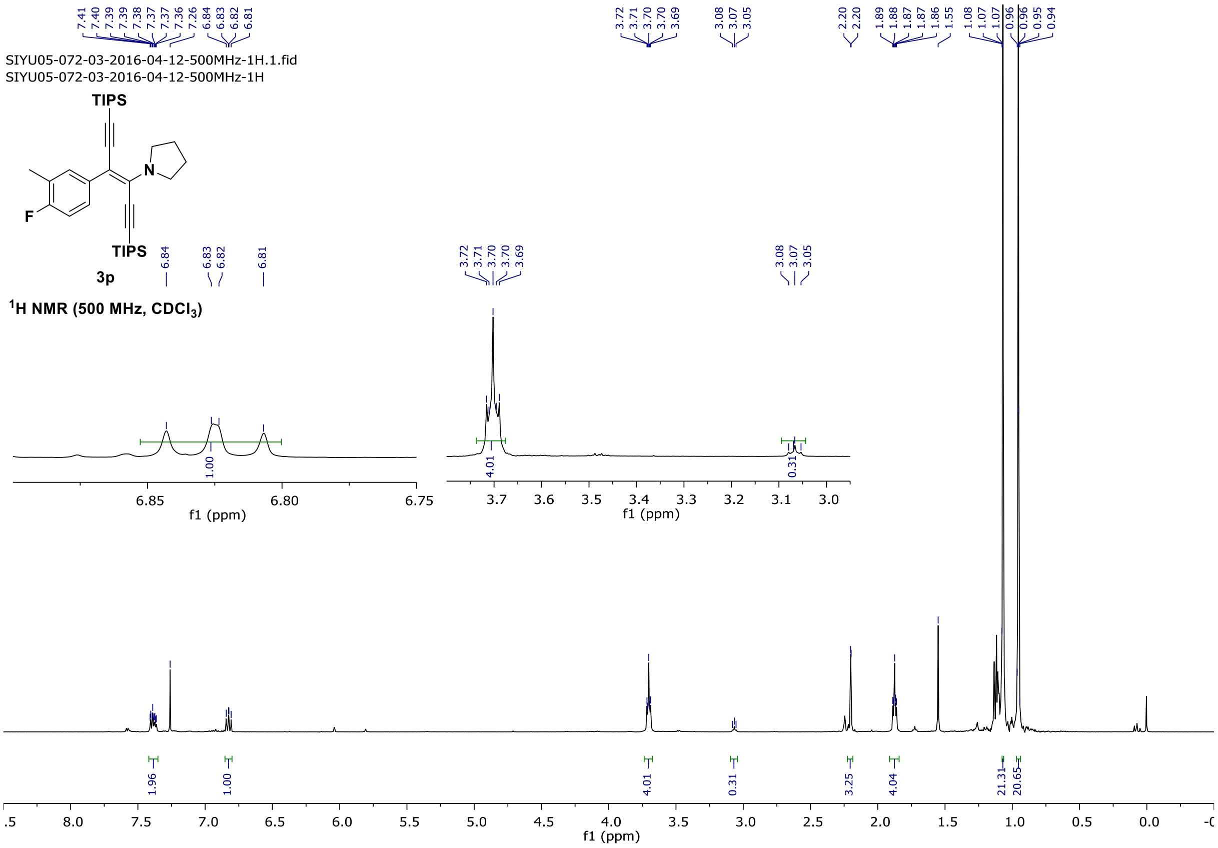

Supplementary Figure 33. ^{1}H NMR of the **3p** (500 MHz, $CDCl_3$)

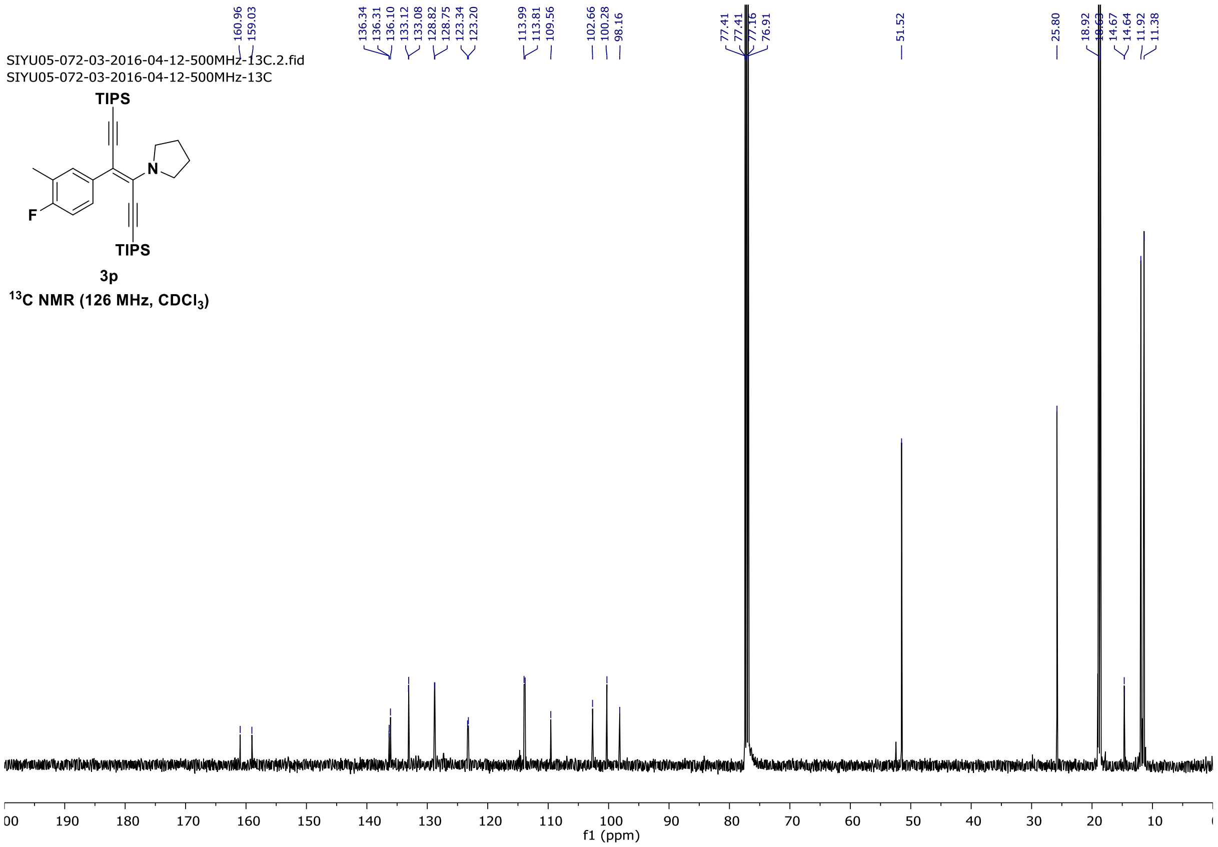

Supplementary Figure 34. ^{13}C NMR of the **3p** (126 MHz, $CDCl_3$)

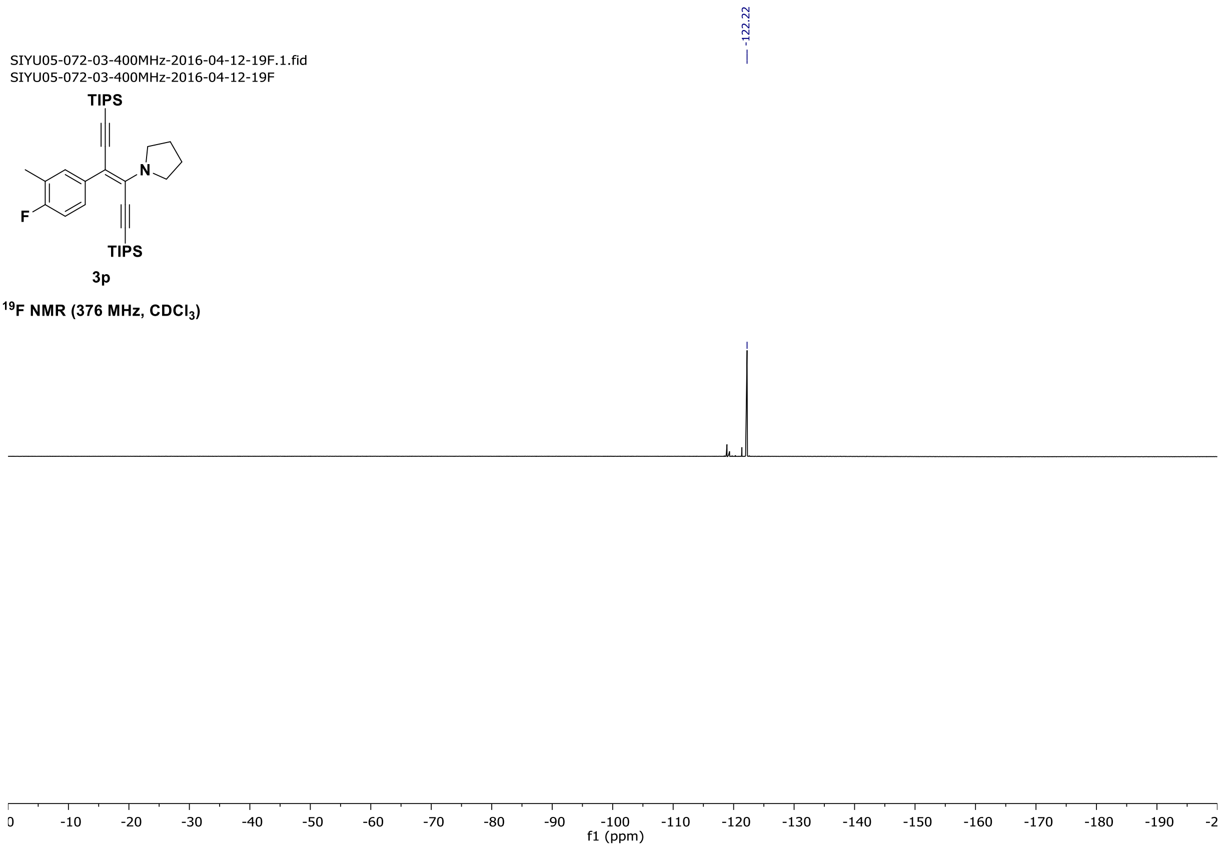

Supplementary Figure 35. ^{19}F NMR of the **3p** (376 MHz, $CDCl_3$)

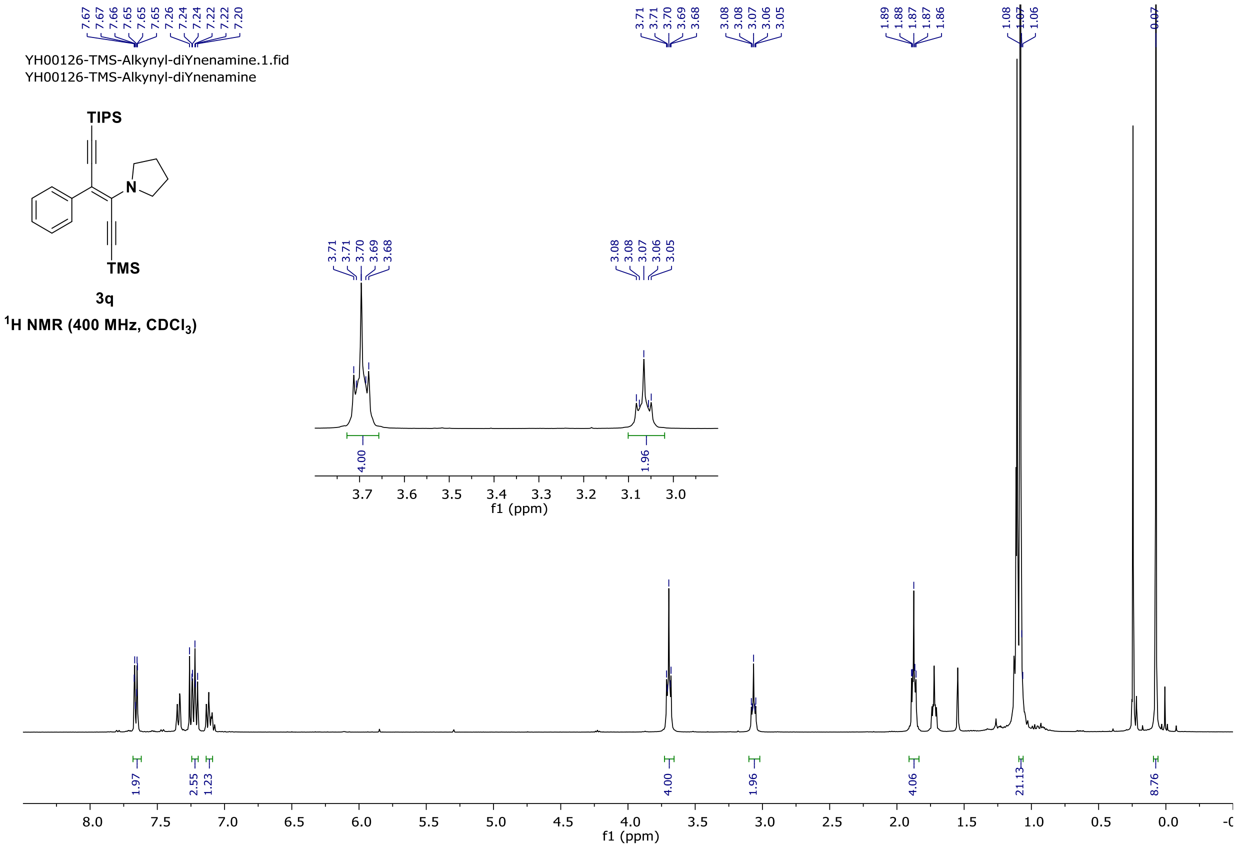

Supplementary Figure 36. ^{1}H NMR of the **3q** (400 MHz, $CDCl_3$)

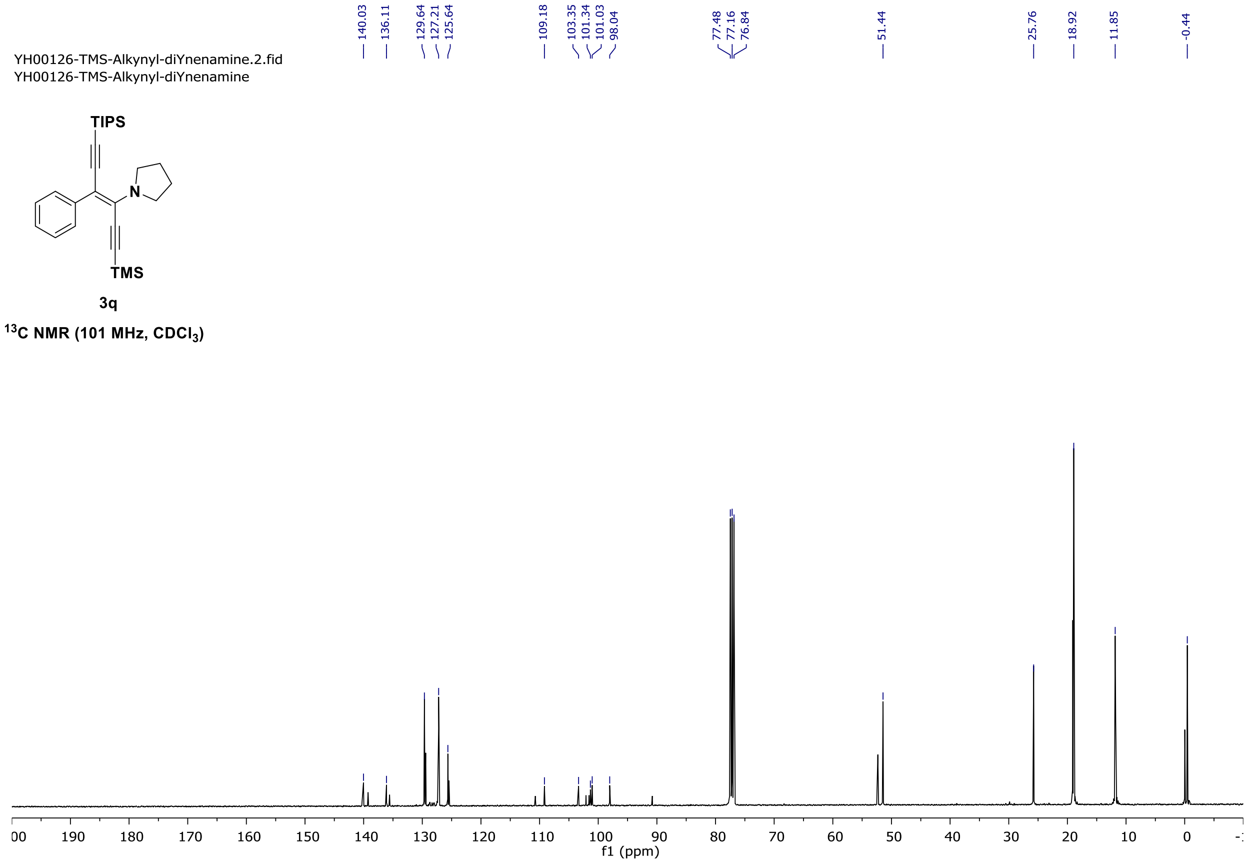

Supplementary Figure 37. ^{13}C NMR of the **3q** (101 MHz, $CDCl_3$)

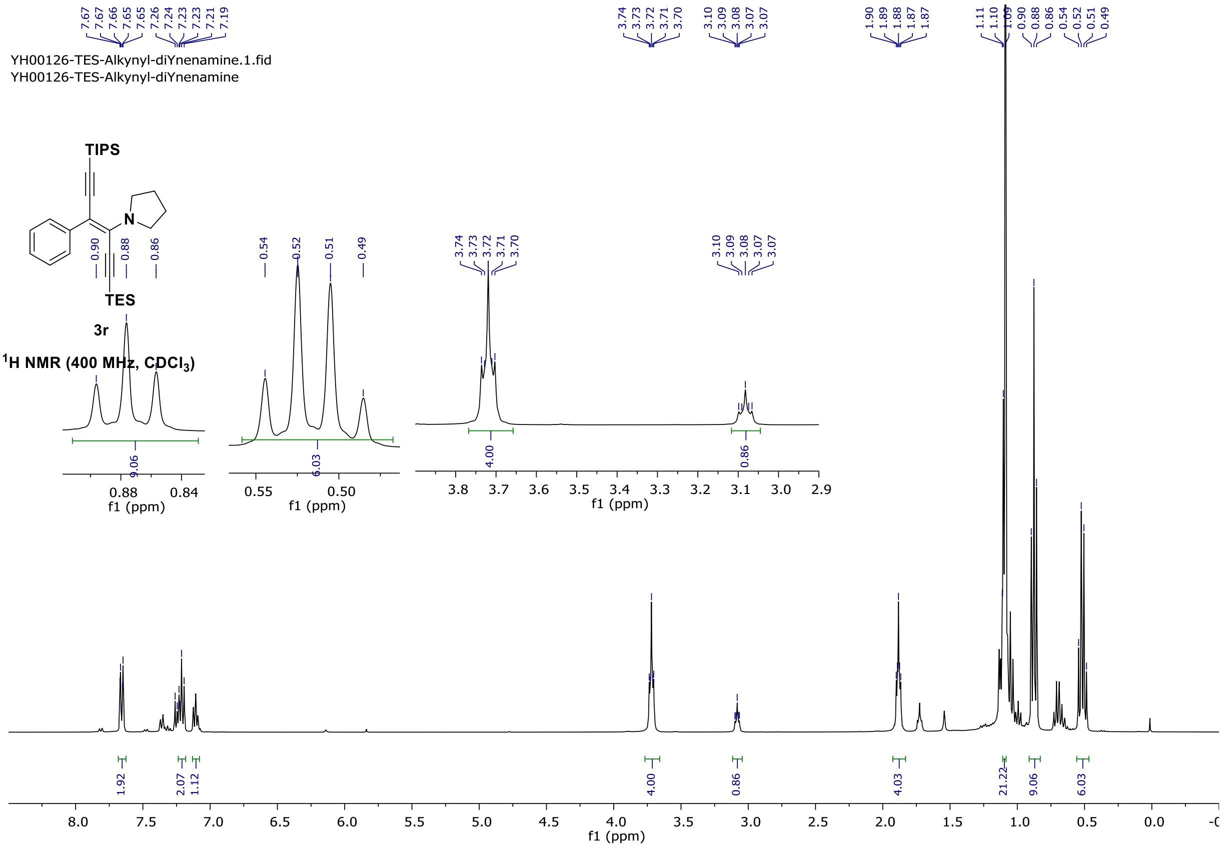

Supplementary Figure 38. ^{1}H NMR of the **3r** (400 MHz, $CDCl_3$)

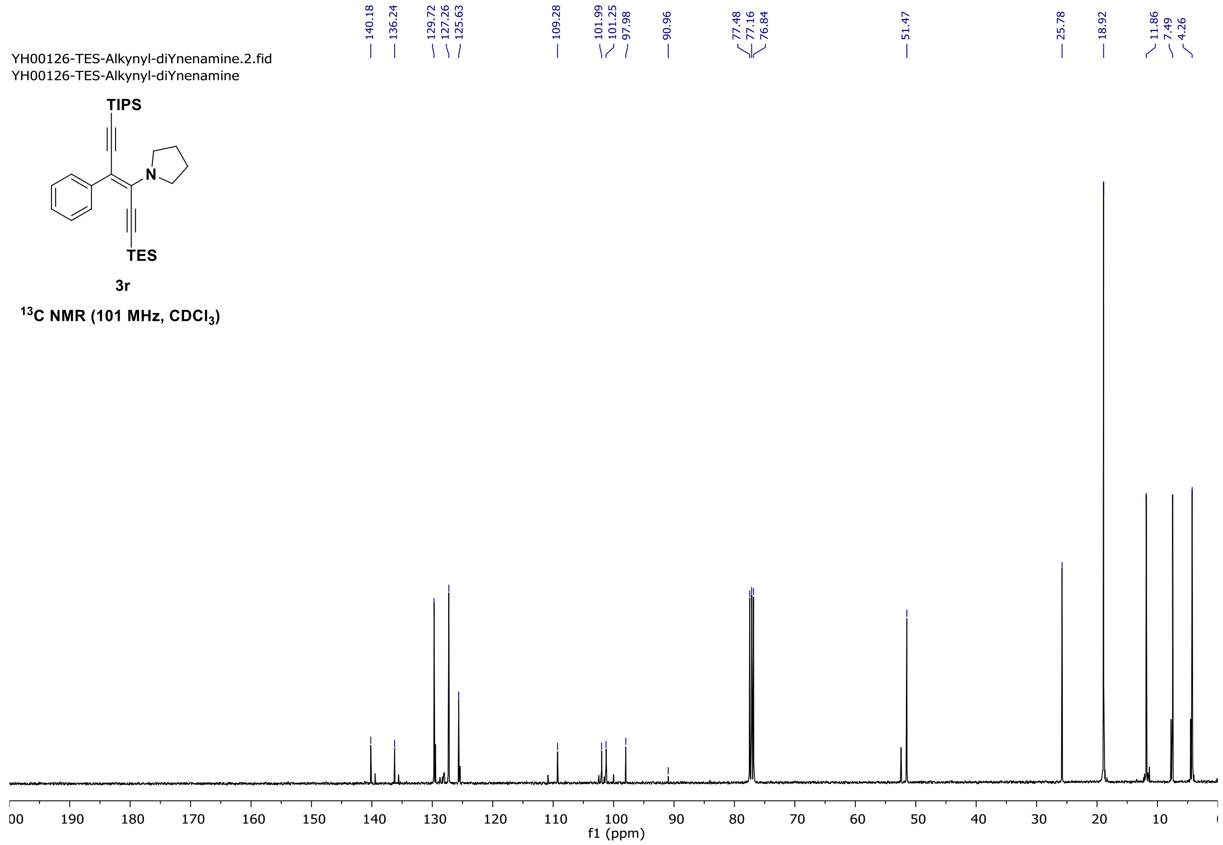

Supplementary Figure 39. ^{13}C NMR of the **3r** (101 MHz, $CDCl_3$)

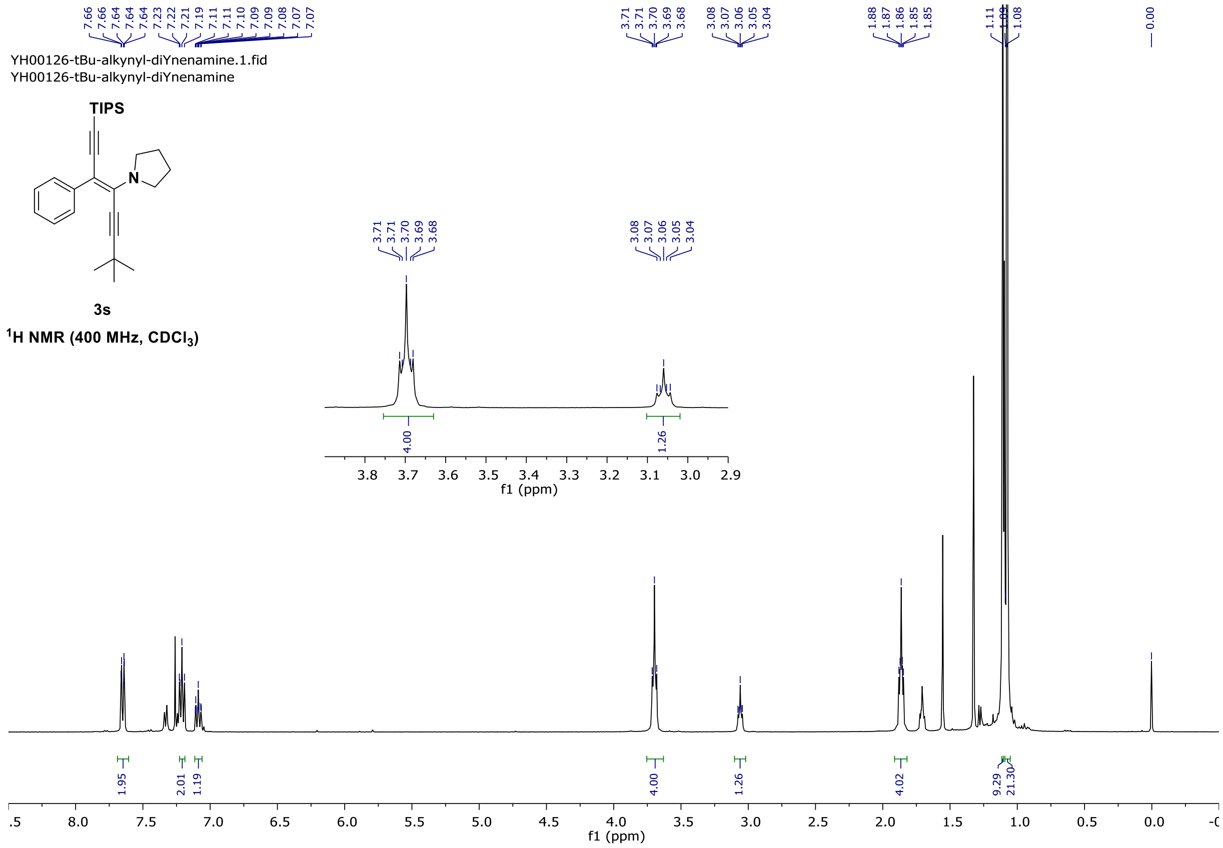

Supplementary Figure 40. ^{1}H NMR of the **3s** (400 MHz, $CDCl_3$)

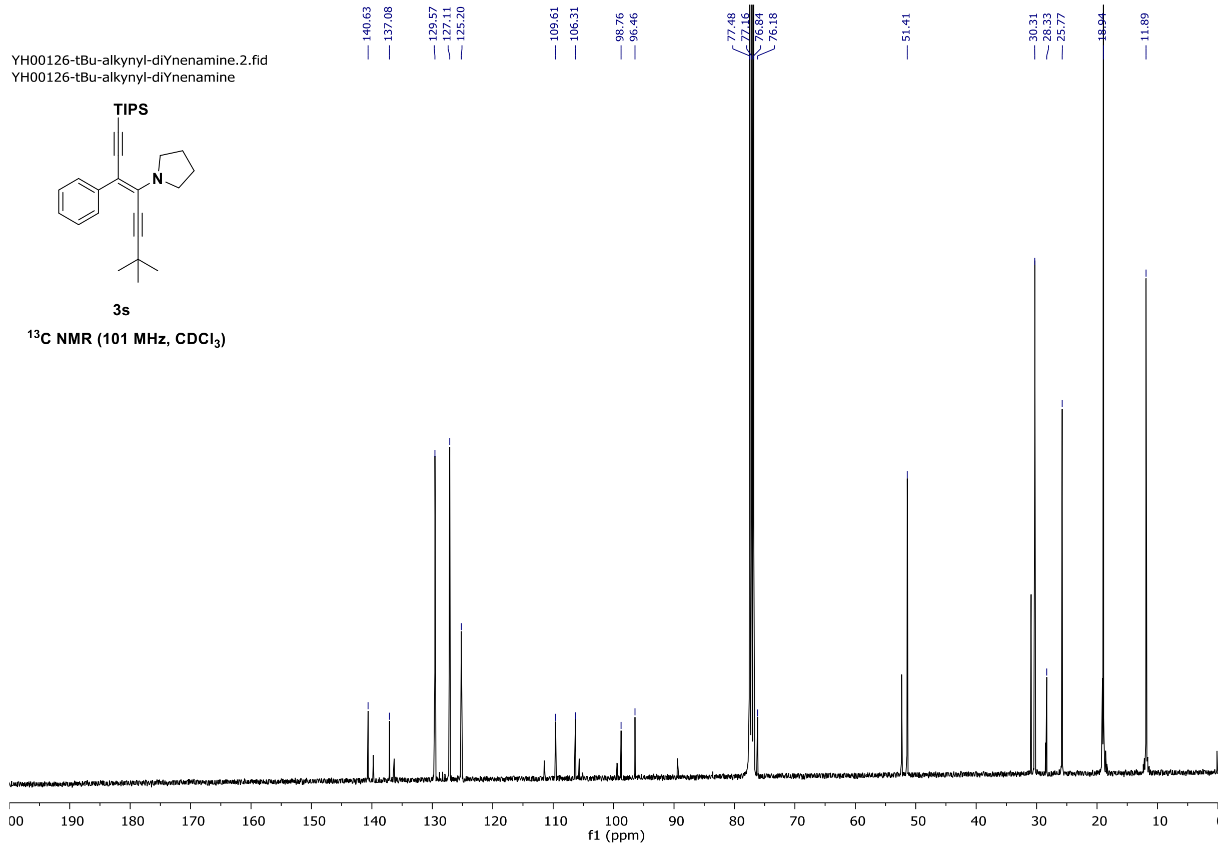

Supplementary Figure 41. ^{13}C NMR of the **3s** (101 MHz, $CDCl_3$)

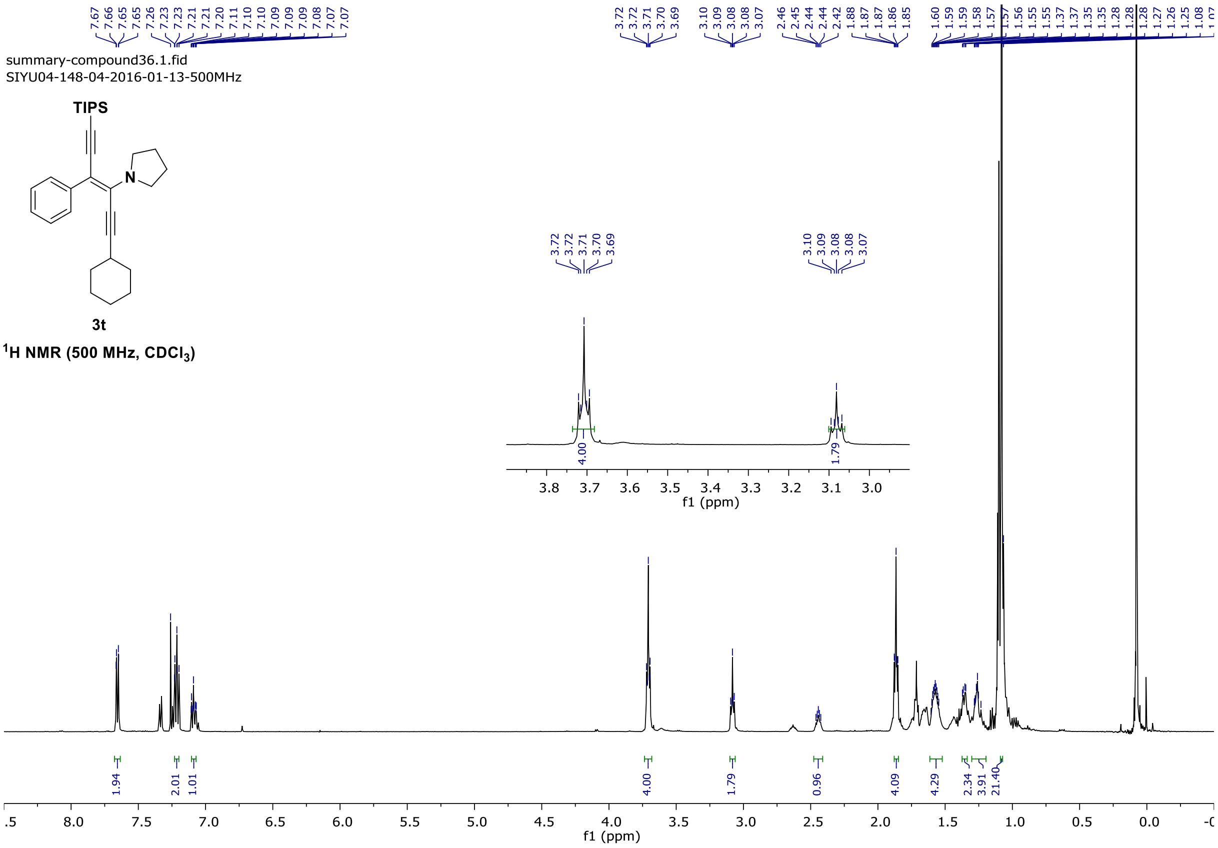

Supplementary Figure 42. ^{1}H NMR of the **3t** (500 MHz, $CDCl_3$)

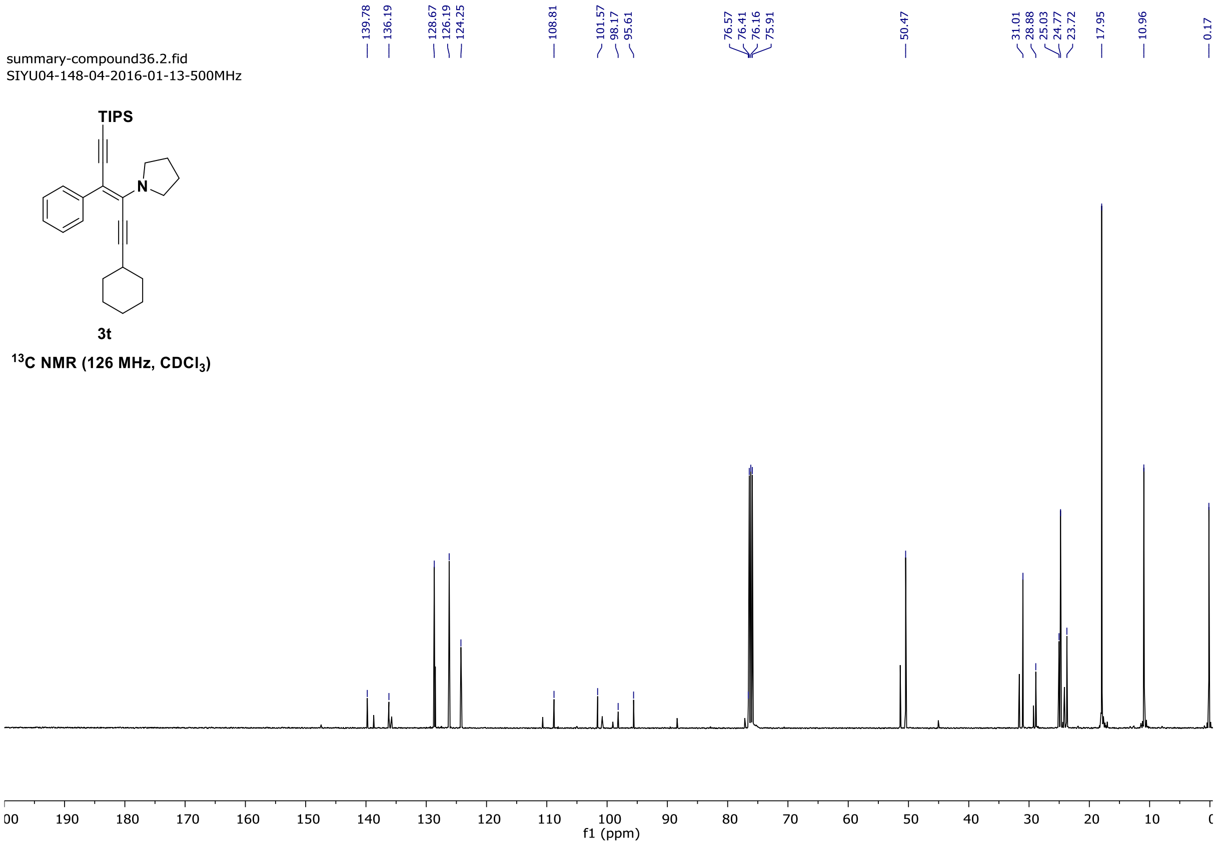

Supplementary Figure 43. ^{13}C NMR of the **3t** (126 MHz, $CDCl_3$)

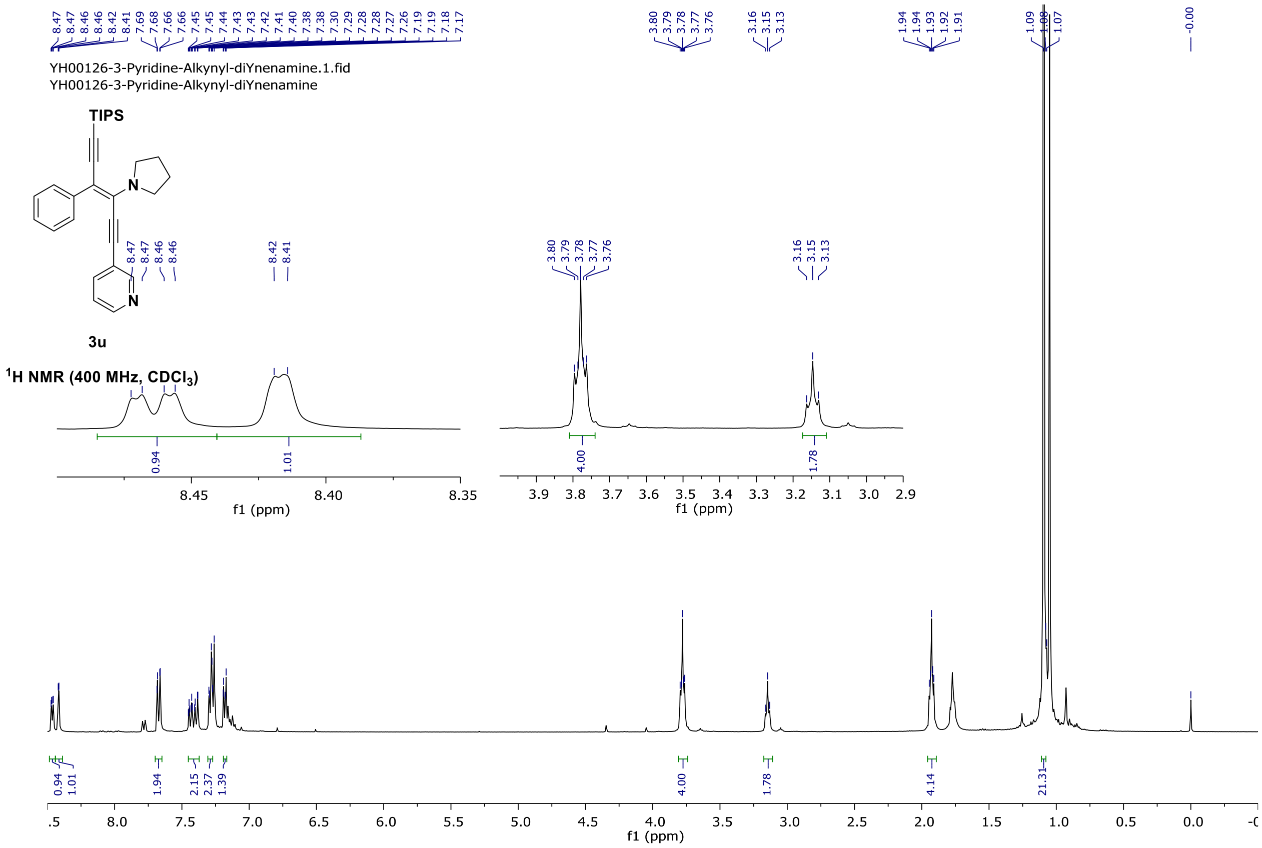

Supplementary Figure 44. ^{1}H NMR of the **3u** (400 MHz, $CDCl_3$)

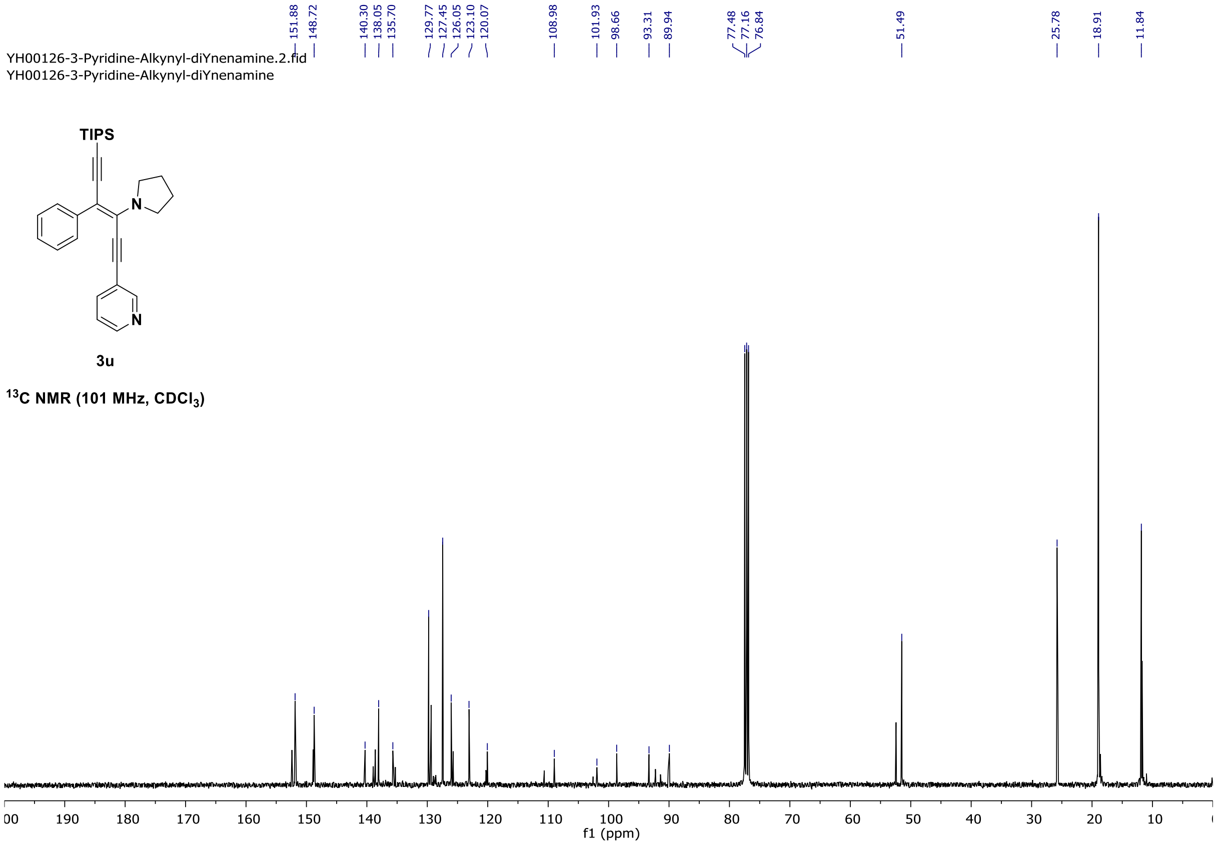

Supplementary Figure 45. ^{13}C NMR of the **3u** (101 MHz, $CDCl_3$)

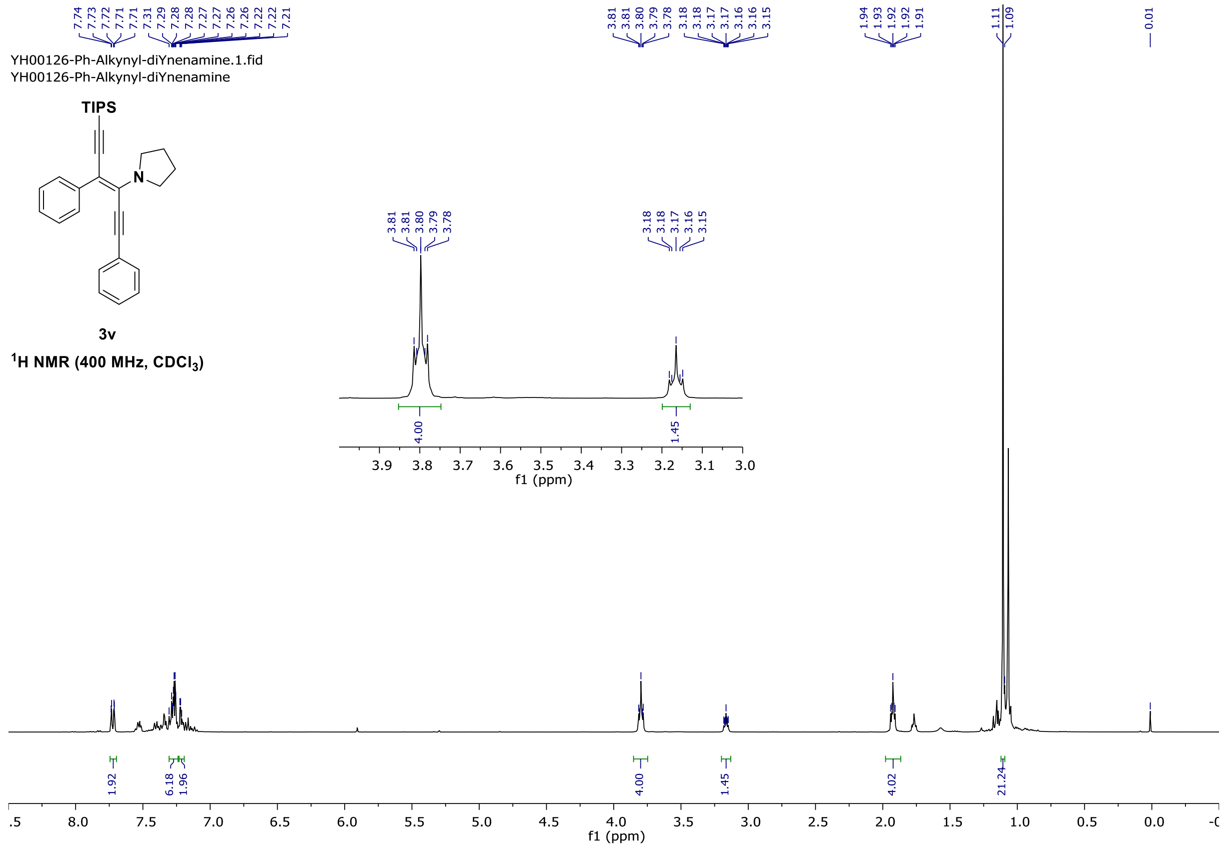

Supplementary Figure 46. ^{1}H NMR of the **3v** (400 MHz, $CDCl_3$)

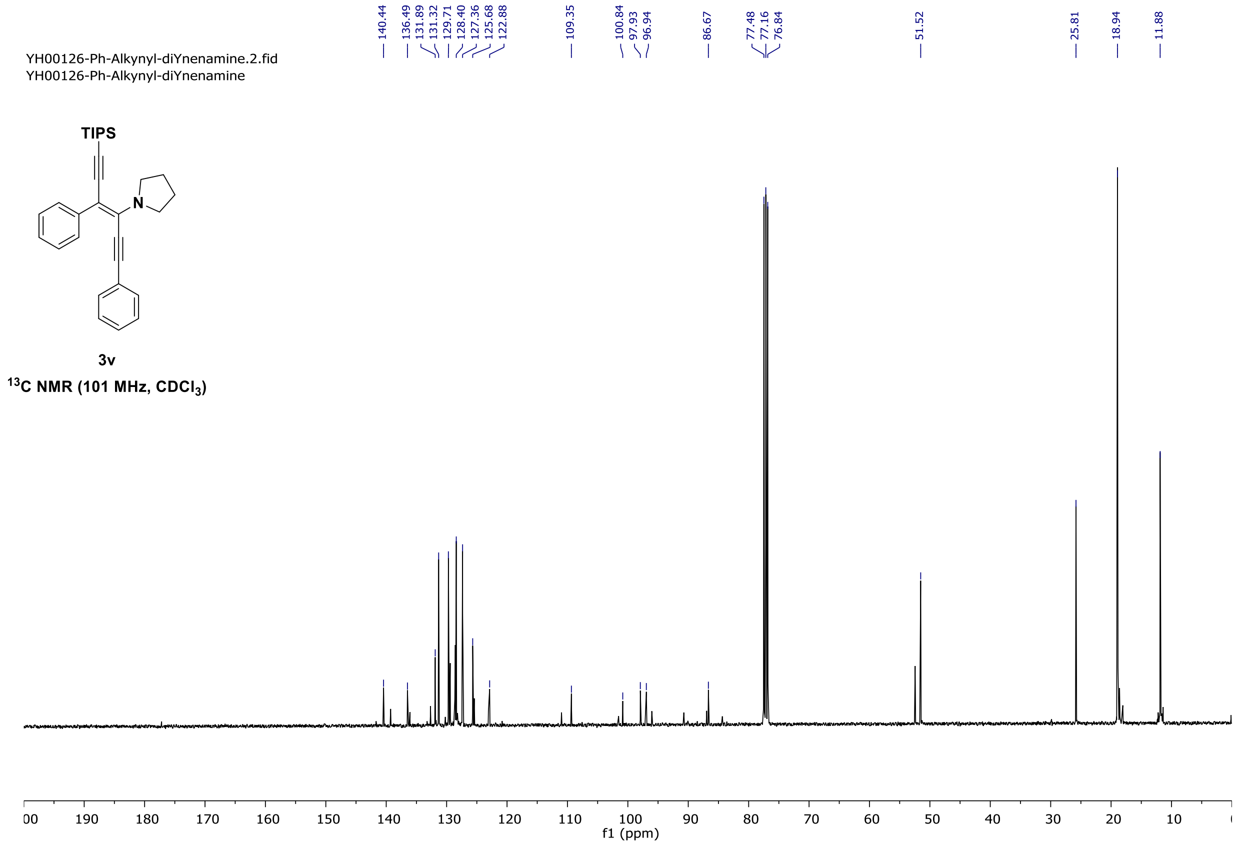

Supplementary Figure 47. ^{13}C NMR of the **3v** (101 MHz, $CDCl_3$)

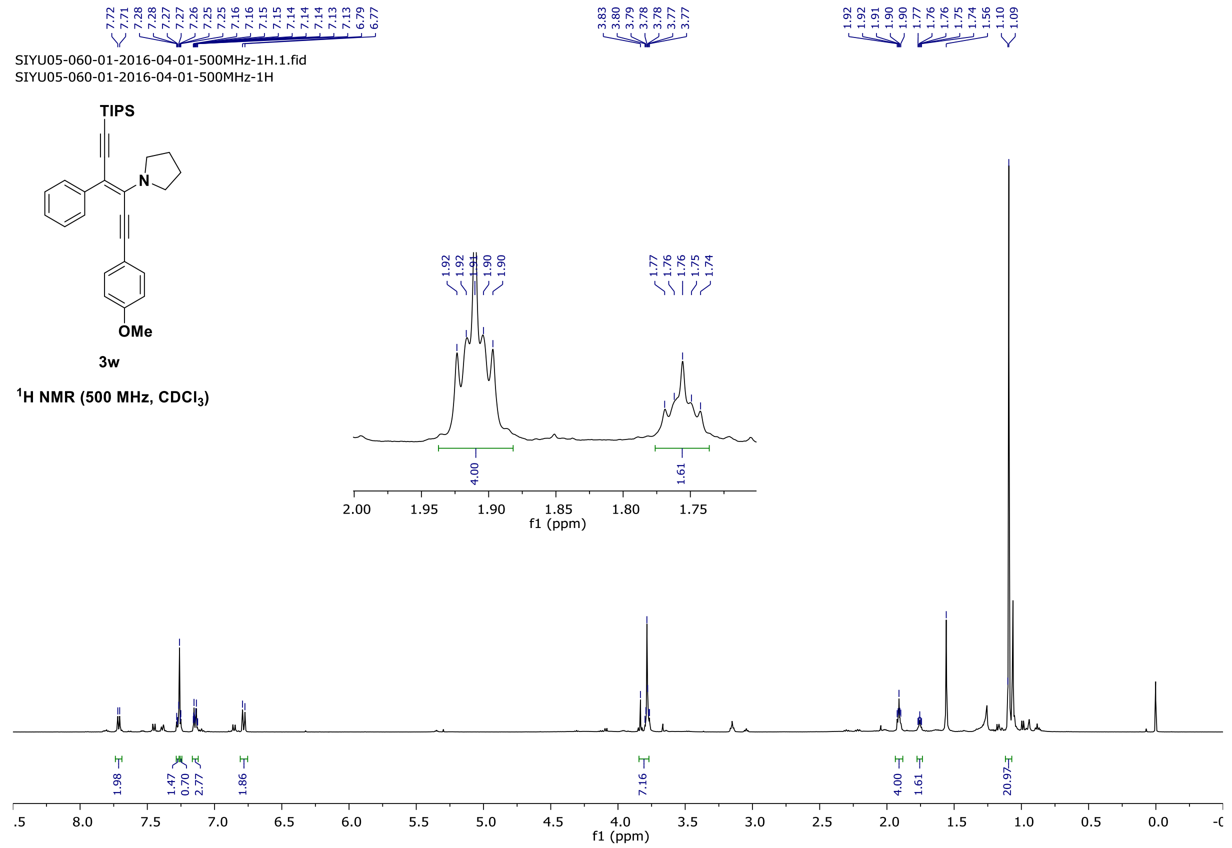

Supplementary Figure 48. ^{1}H NMR of the **3w** (500 MHz, $CDCl_3$)

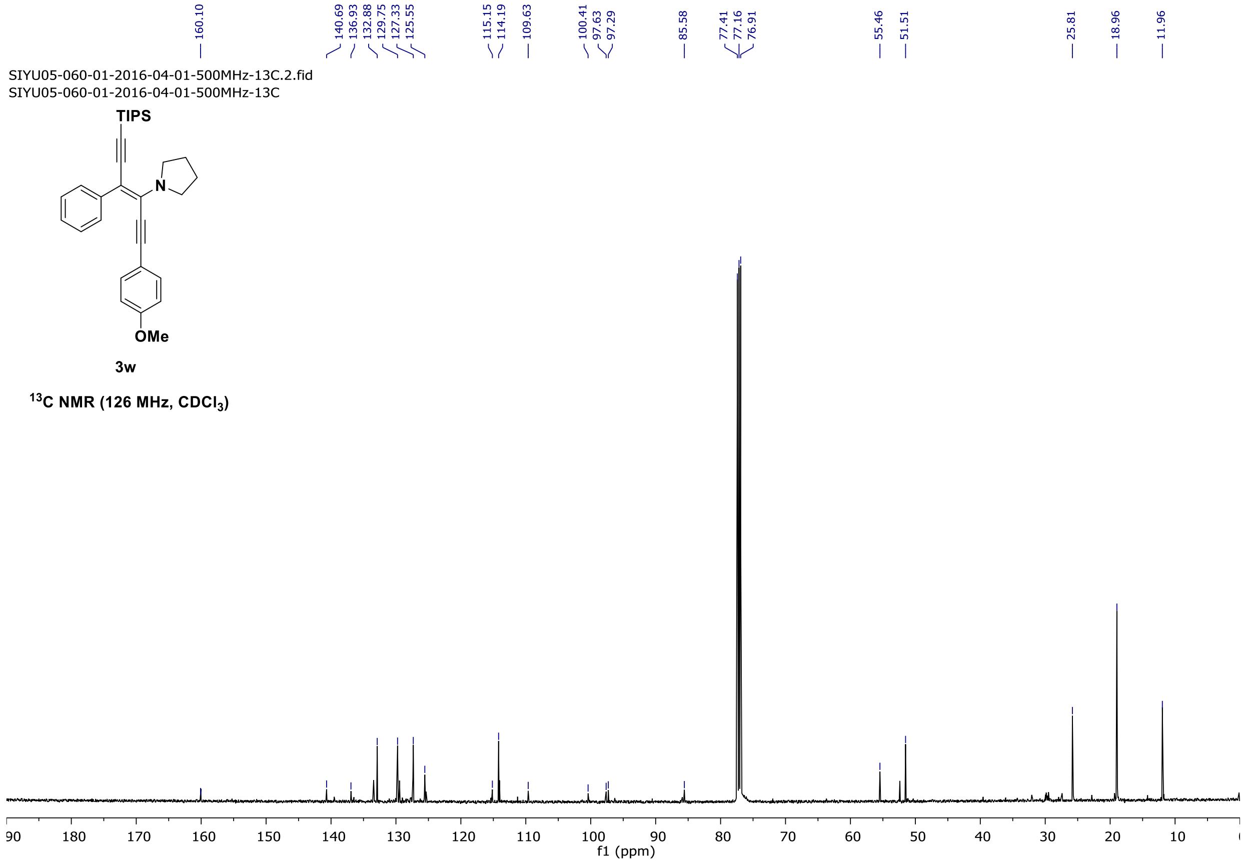

Supplementary Figure 49. ^{13}C NMR of the **3w** (126 MHz, $CDCl_3$)

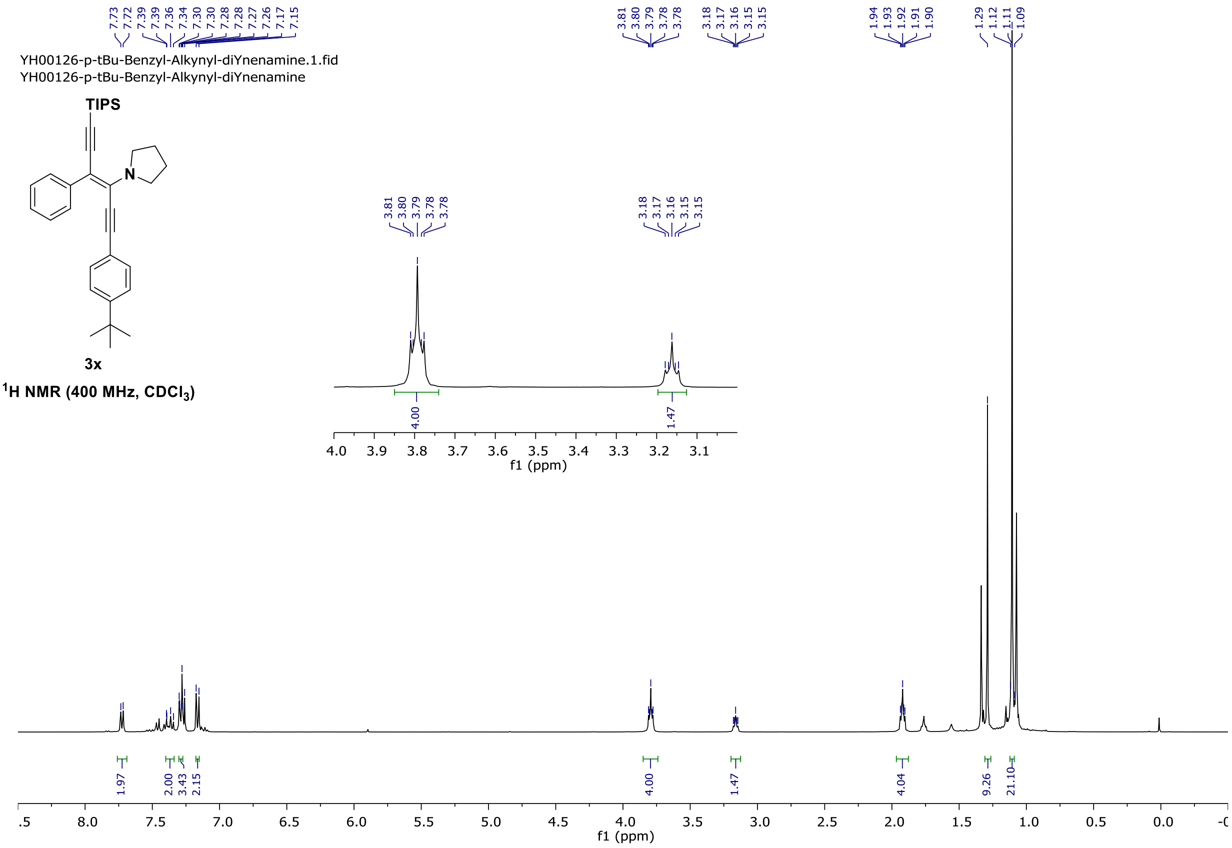

Supplementary Figure 50. ^{1}H NMR of the **3x** (400 MHz, $CDCl_3$)

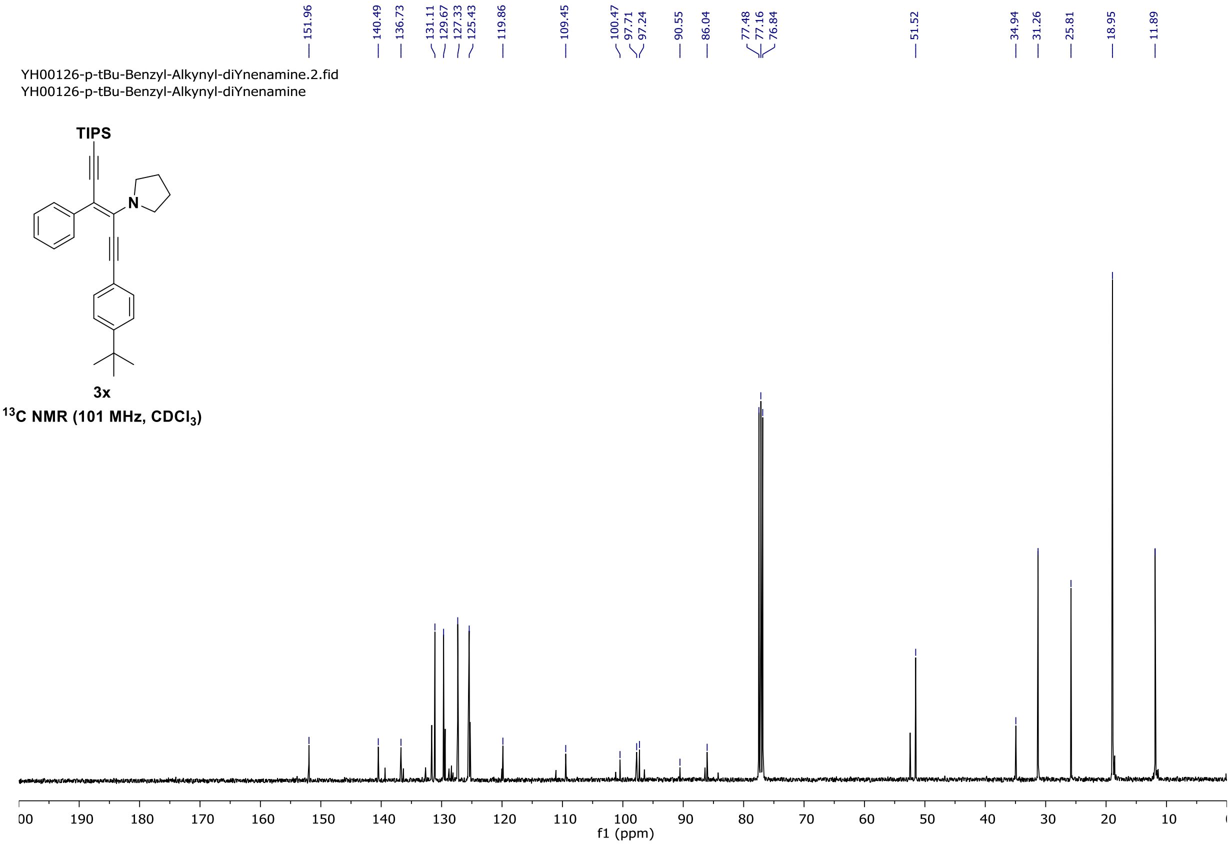

Supplementary Figure 51. ^{13}C NMR of the **3x** (101 MHz, $CDCl_3$)

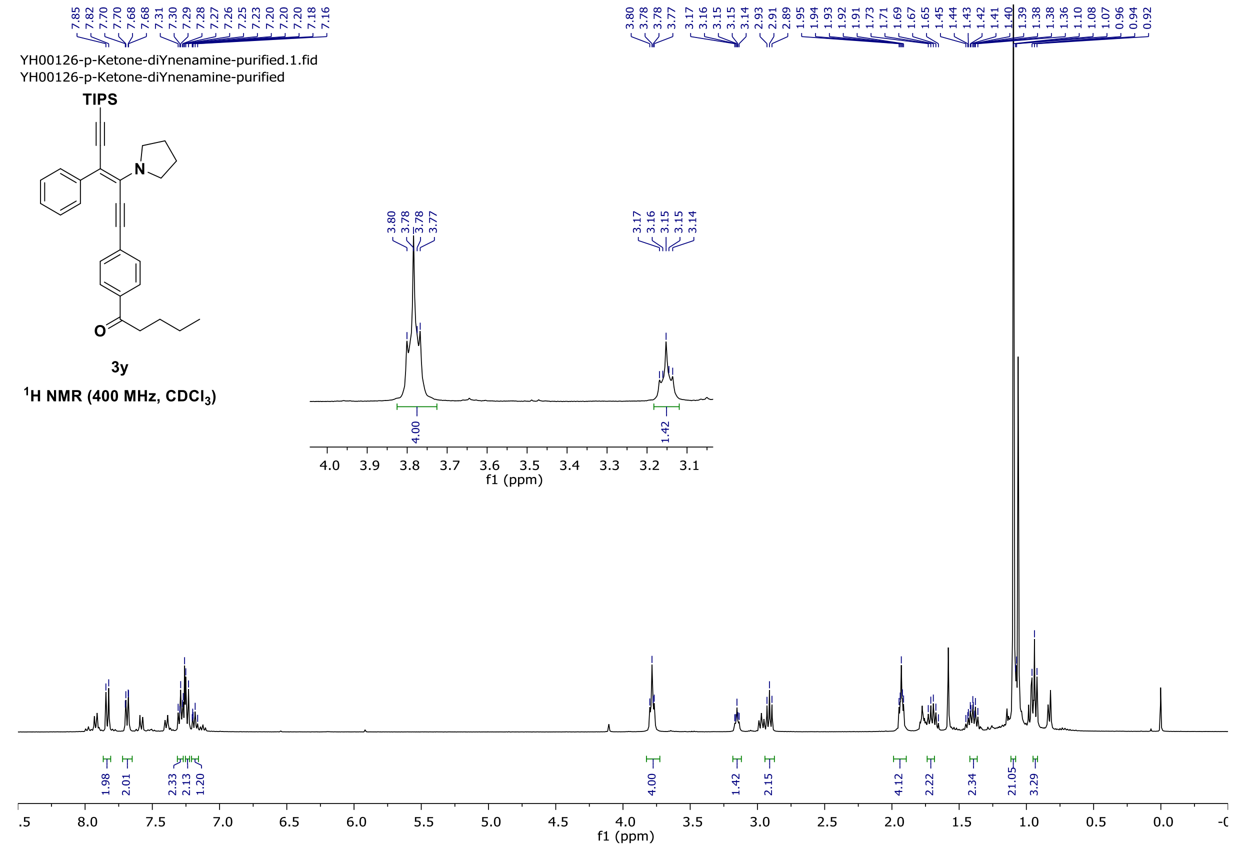

Supplementary Figure 52. ^{1}H NMR of the **3y** (400 MHz, $CDCl_3$)

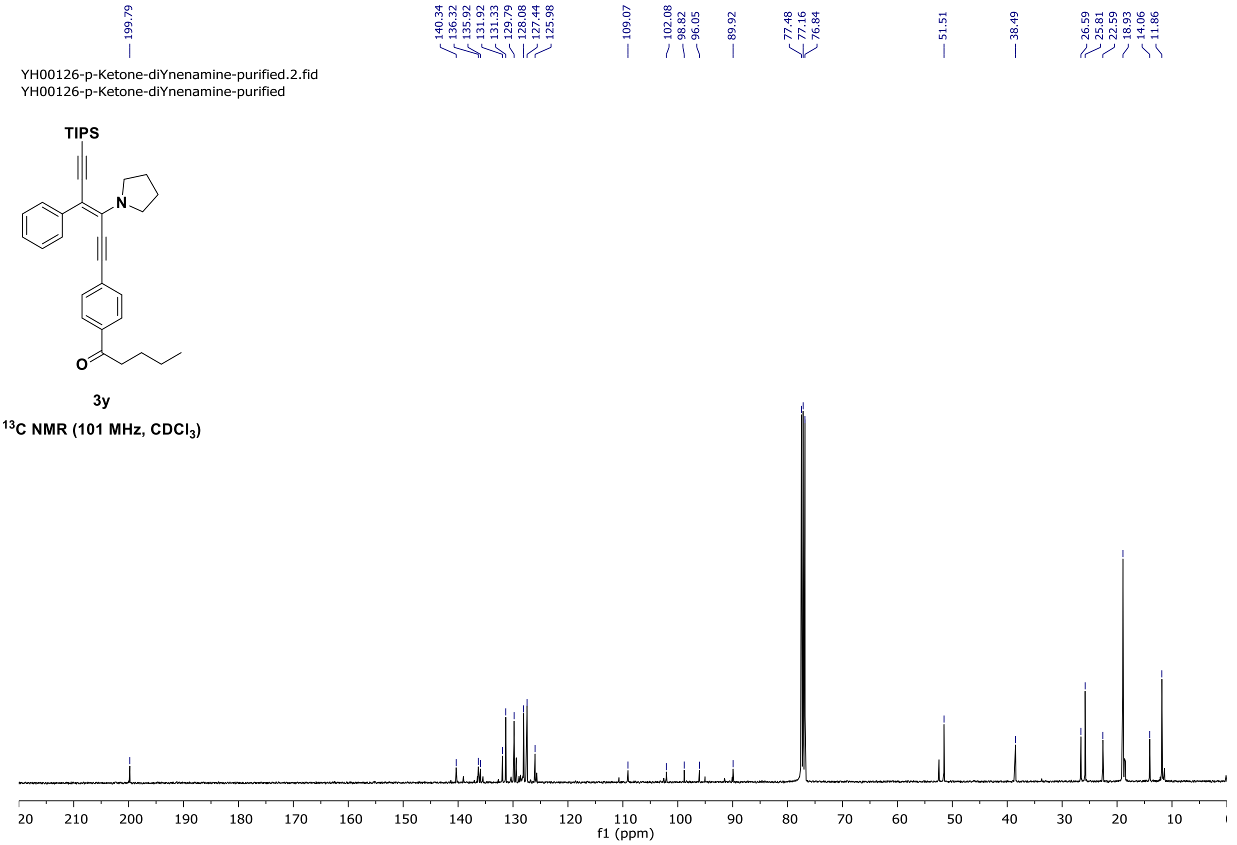

Supplementary Figure 53. ^{13}C NMR of the **3y** (101 MHz, $CDCl_3$)

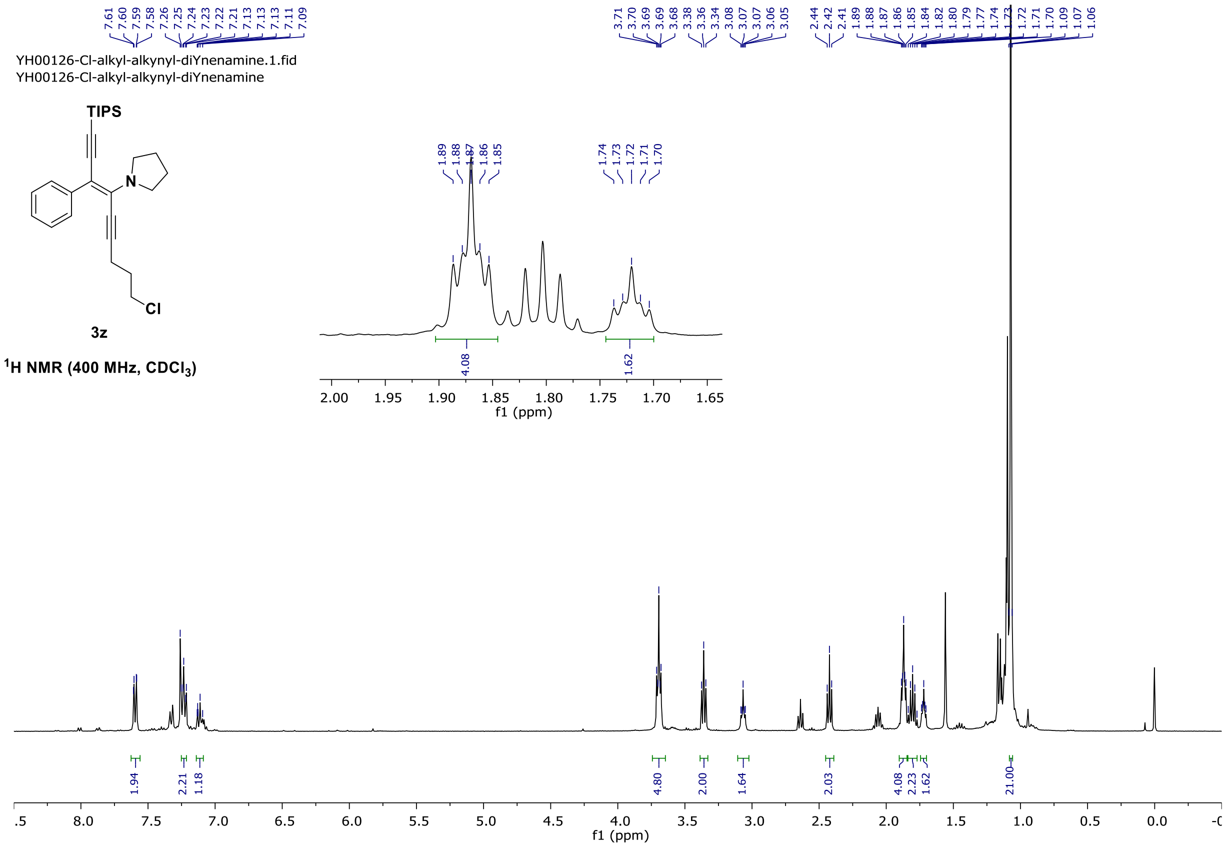

Supplementary Figure 54. ^{1}H NMR of the **3z** (400 MHz, $CDCl_3$)

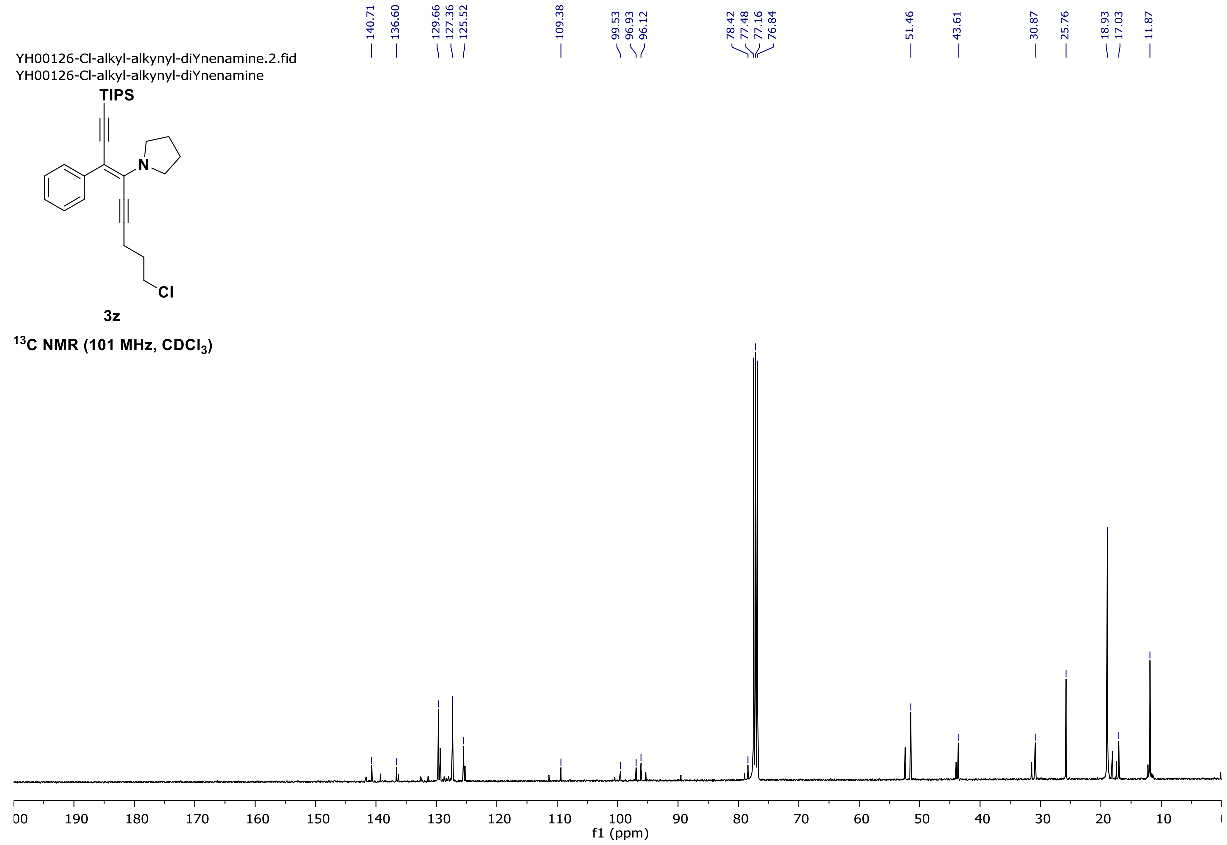

Supplementary Figure 55. ^{13}C NMR of the **3z** (101 MHz, $CDCl_3$)

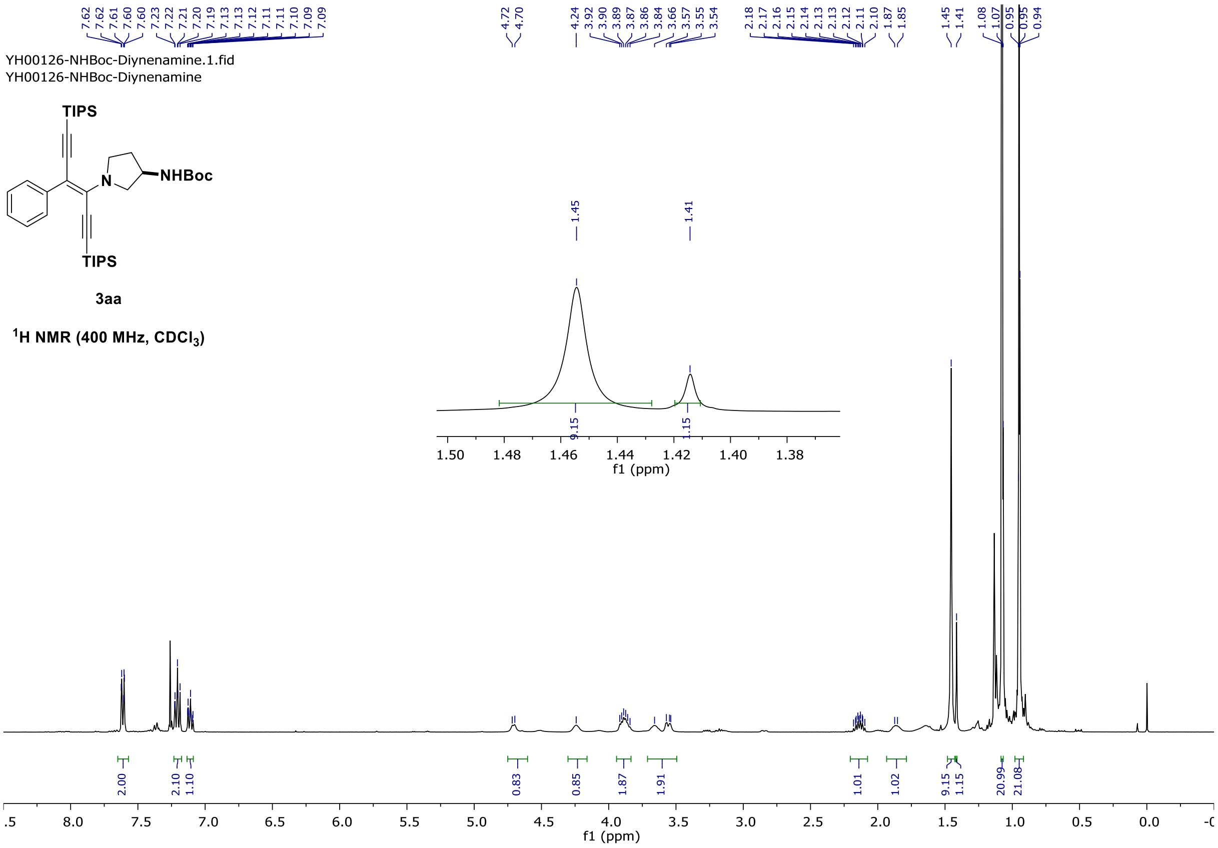

Supplementary Figure 56. ^{1}H NMR of the **3aa** (400 MHz, $CDCl_3$)

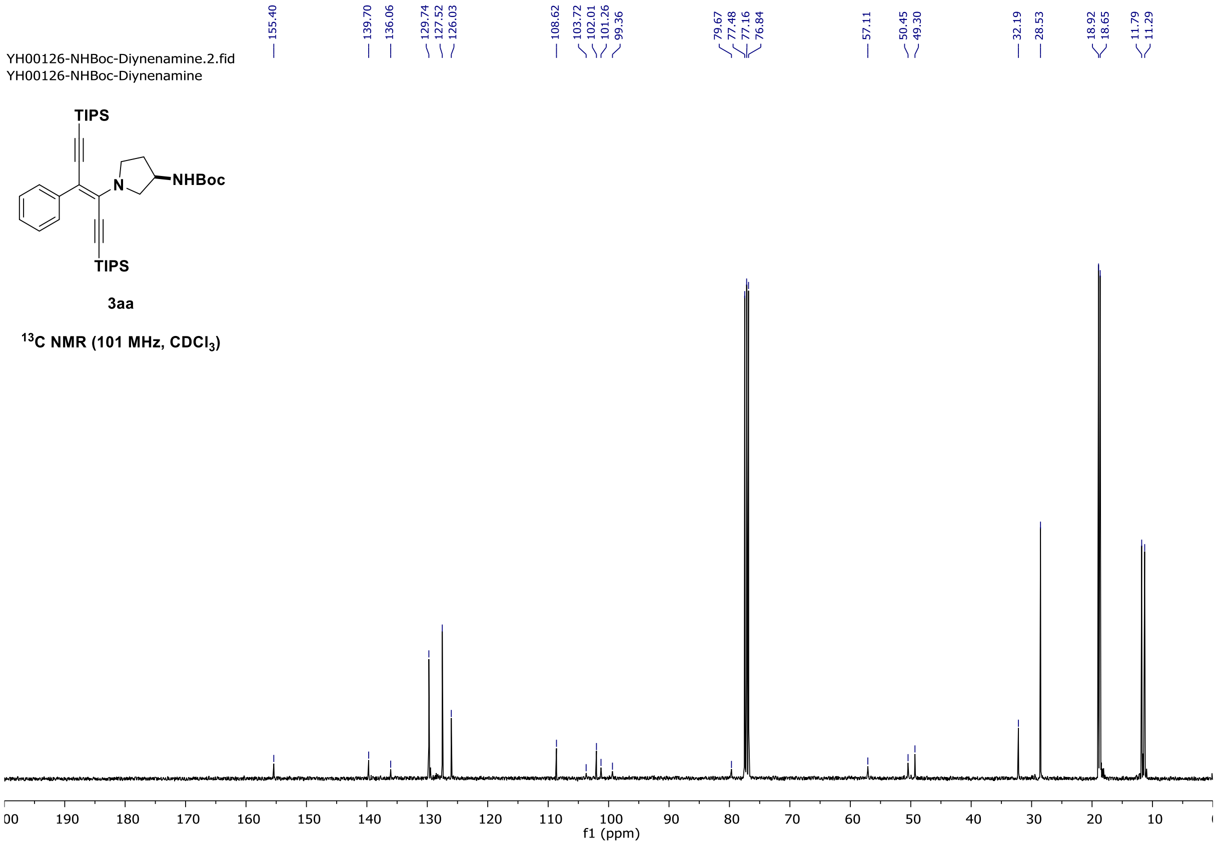

Supplementary Figure 57. ^{13}C NMR of the **3aa** (101 MHz, $CDCl_3$)

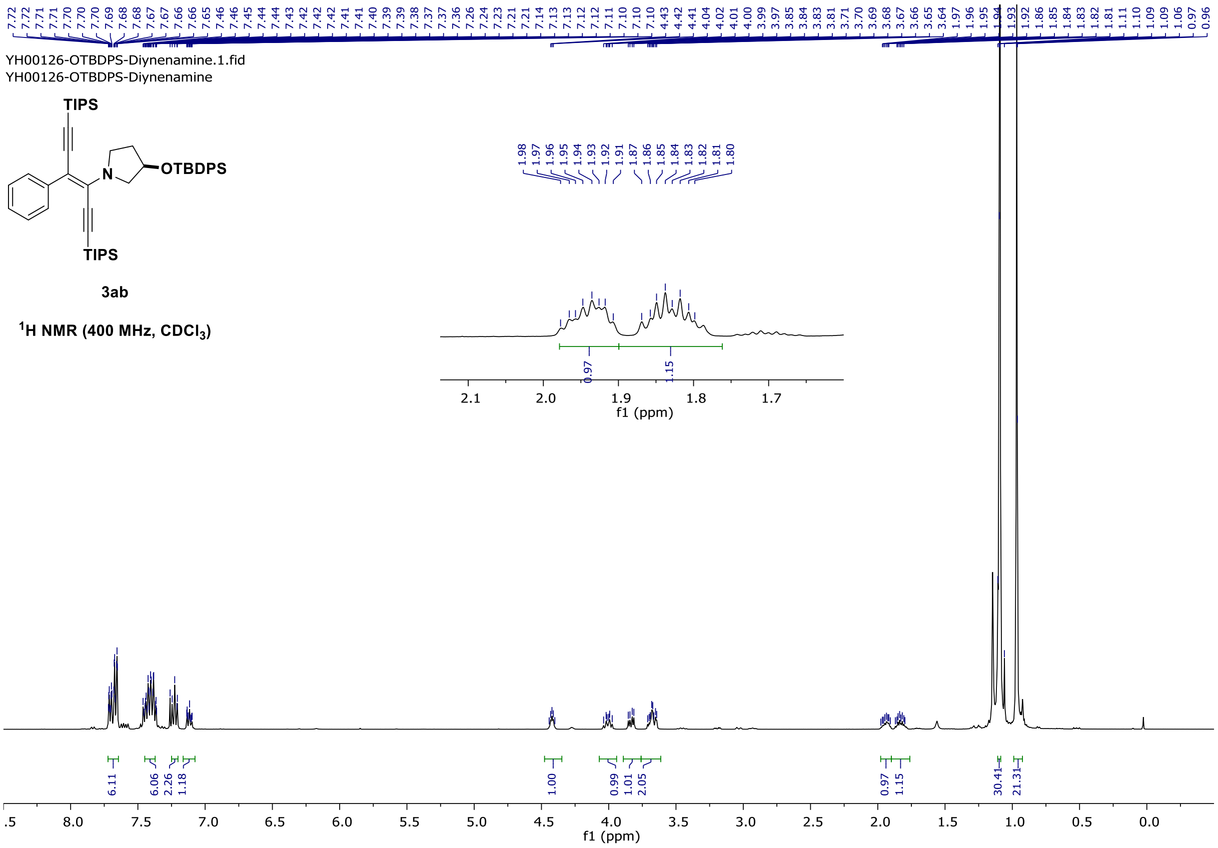

Supplementary Figure 58. ^{1}H NMR of the **3ab** (400 MHz, $CDCl_3$)

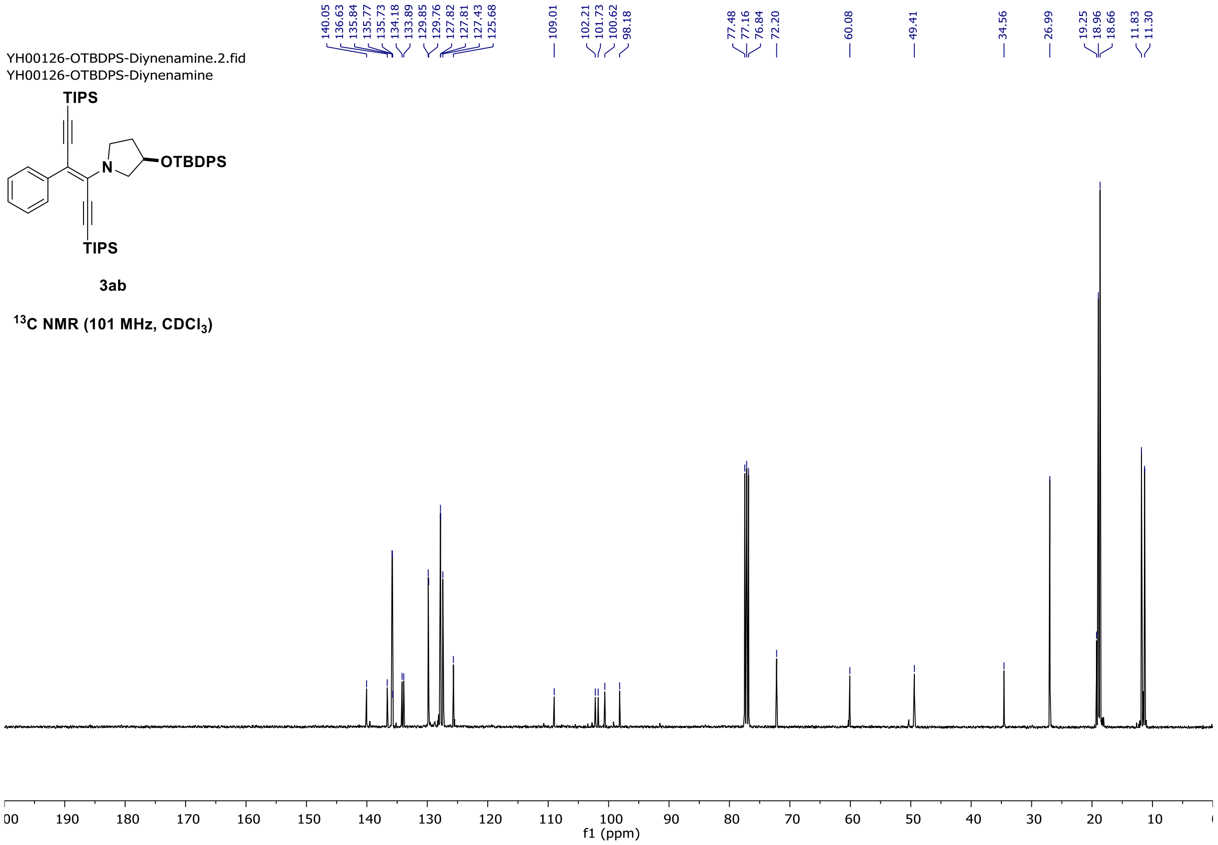

Supplementary Figure 59. ^{13}C NMR of the **3ab** (101 MHz, $CDCl_3$)

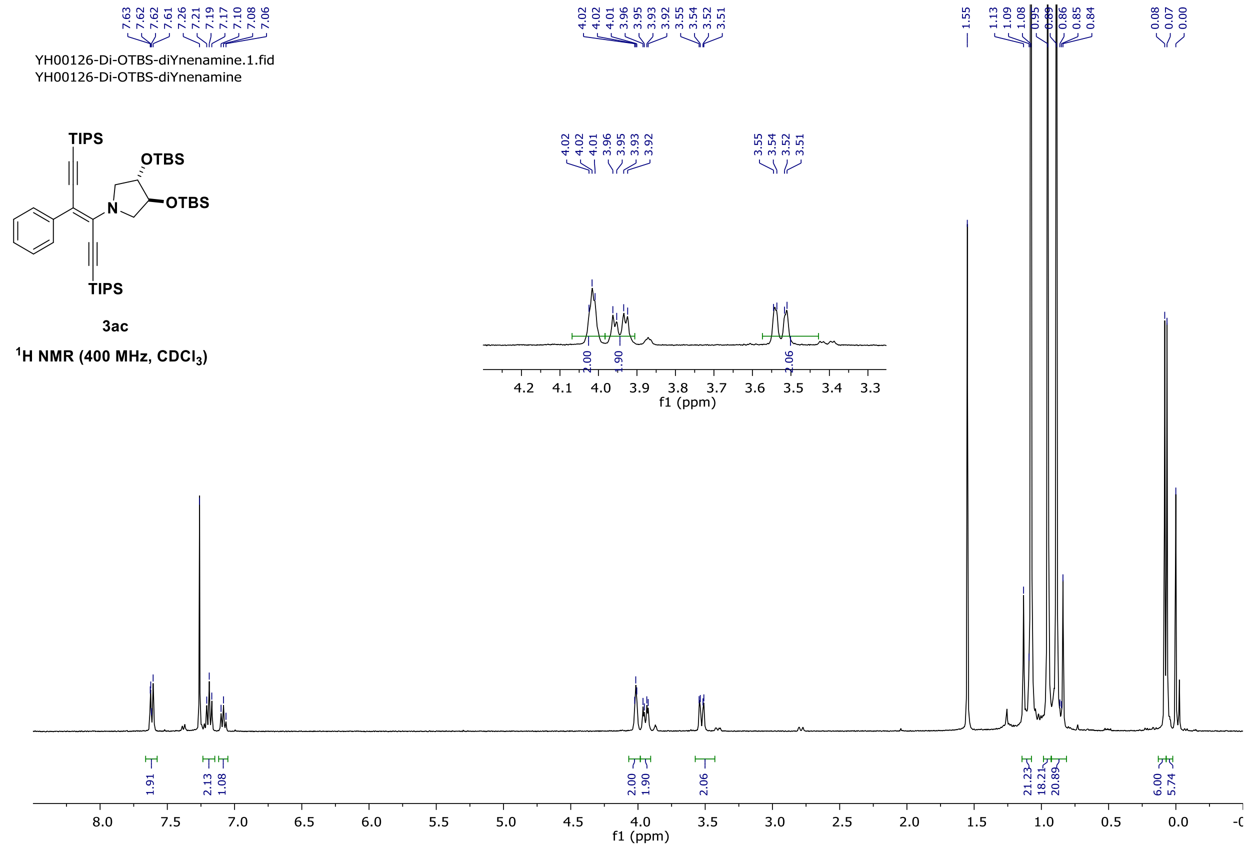

Supplementary Figure 60. ^{1}H NMR of the **3ac** (400 MHz, $CDCl_3$)

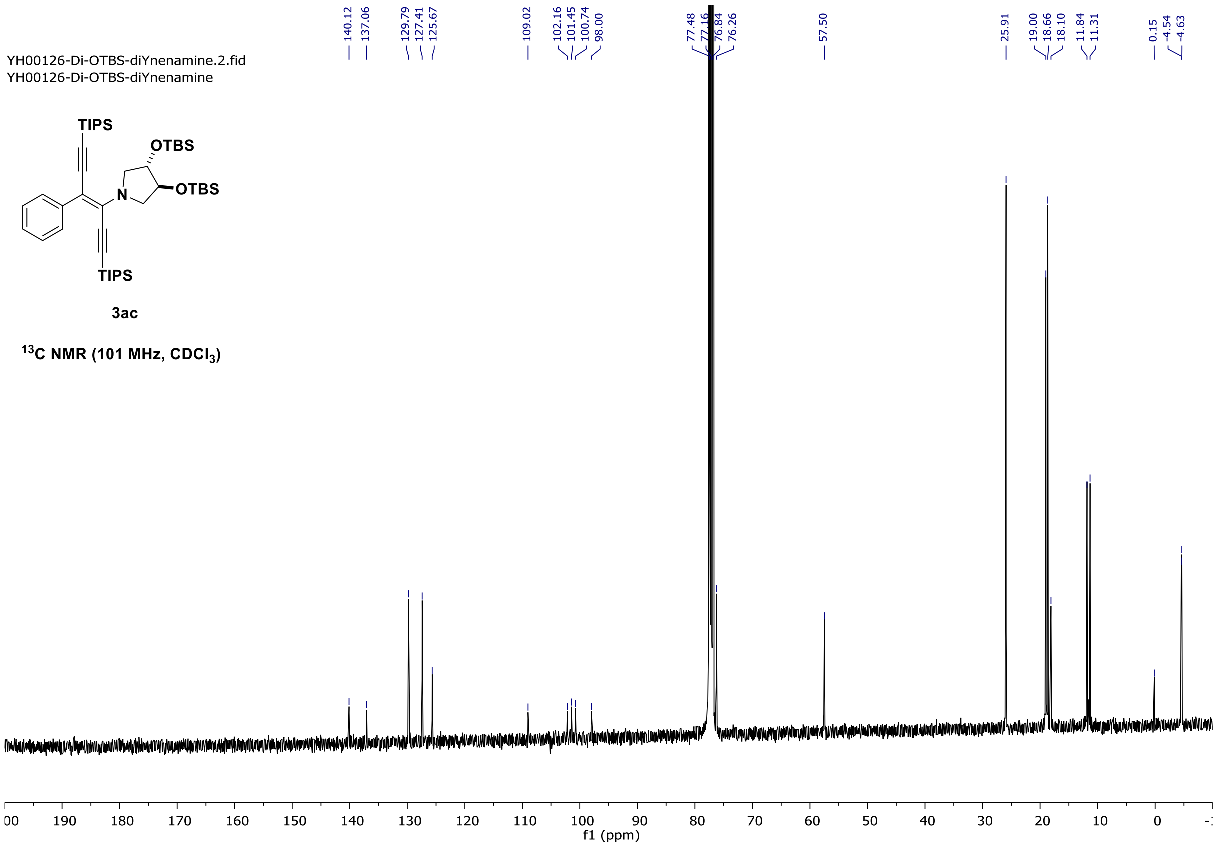

Supplementary Figure 61. ^{13}C NMR of the **3ac** (101 MHz, $CDCl_3$)

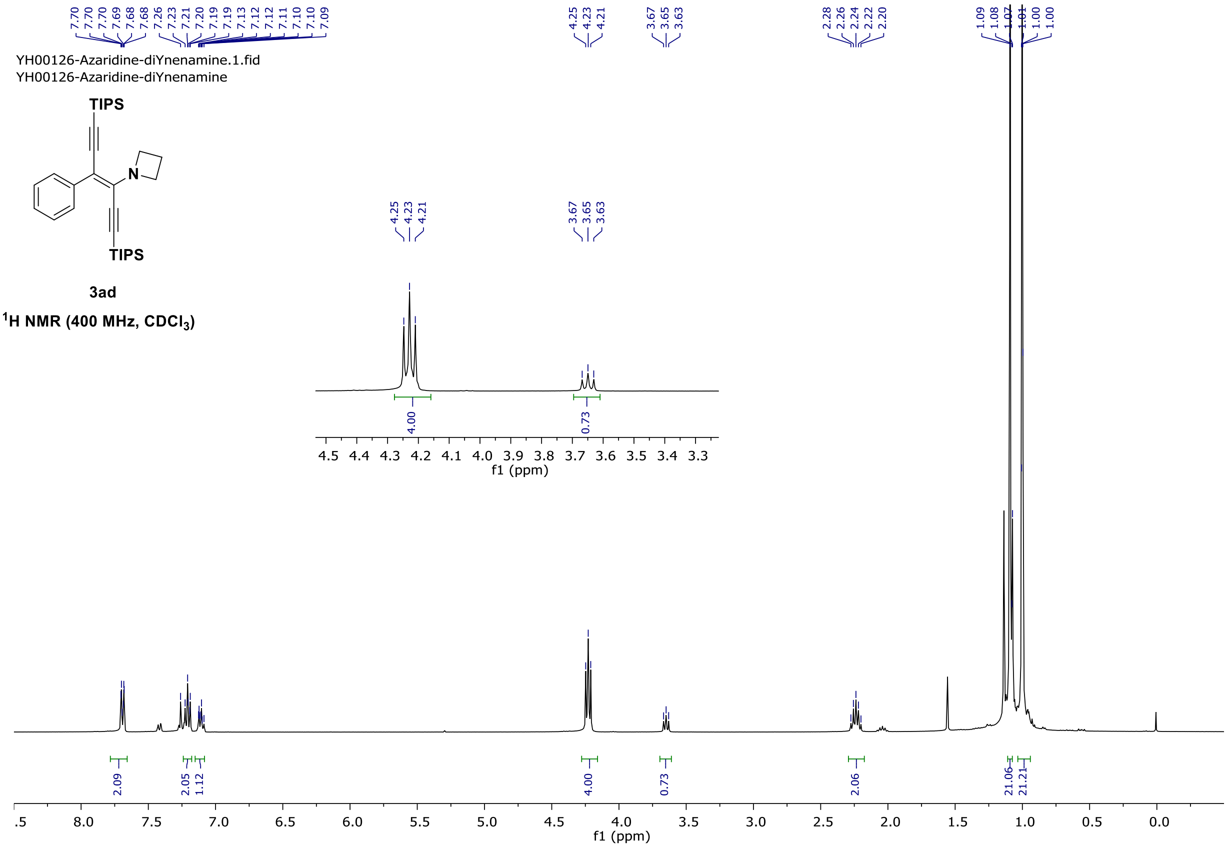

Supplementary Figure 62. ^{1}H NMR of the **3ad** (400 MHz, $CDCl_3$)

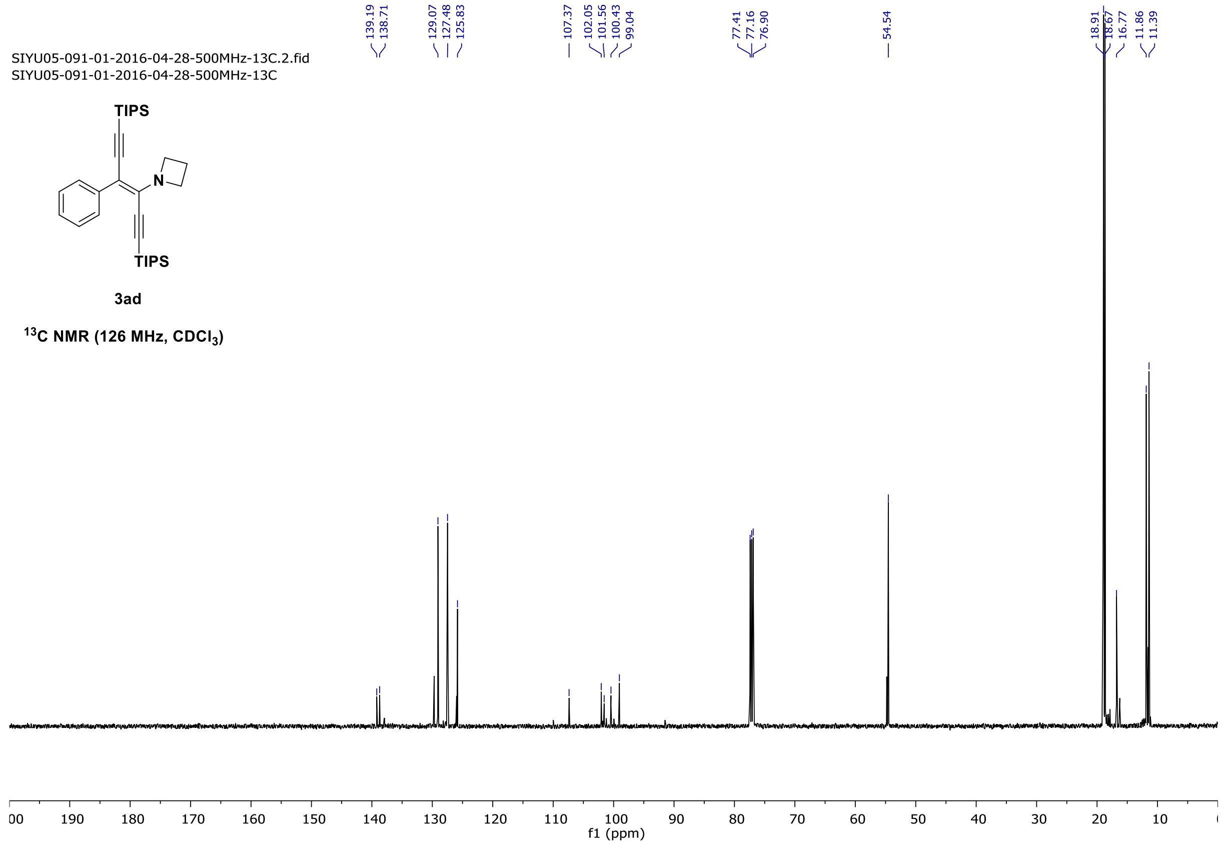

Supplementary Figure 63. ^{13}C NMR of the **3ad** (126 MHz, $CDCl_3$)

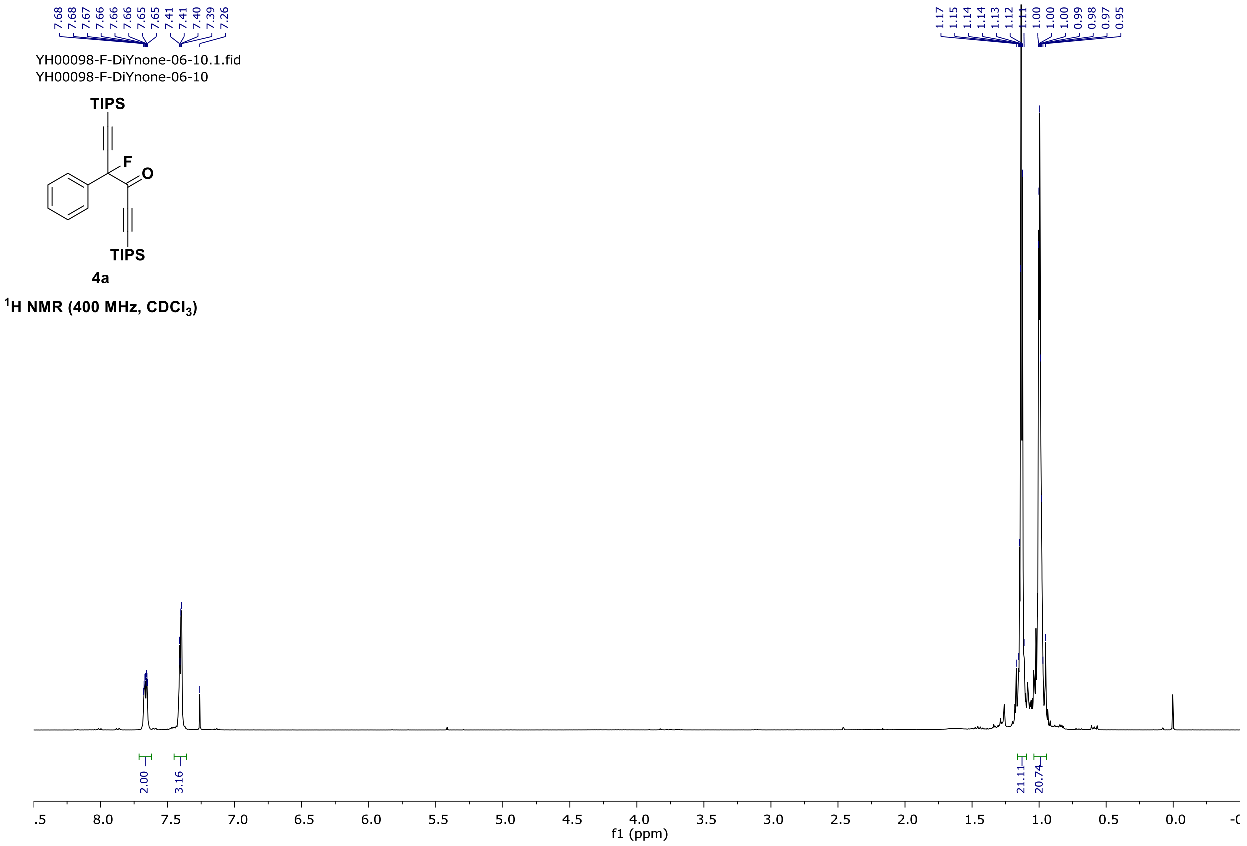

Supplementary Figure 64. ^{1}H NMR of the **4a** (400 MHz, $CDCl_3$)

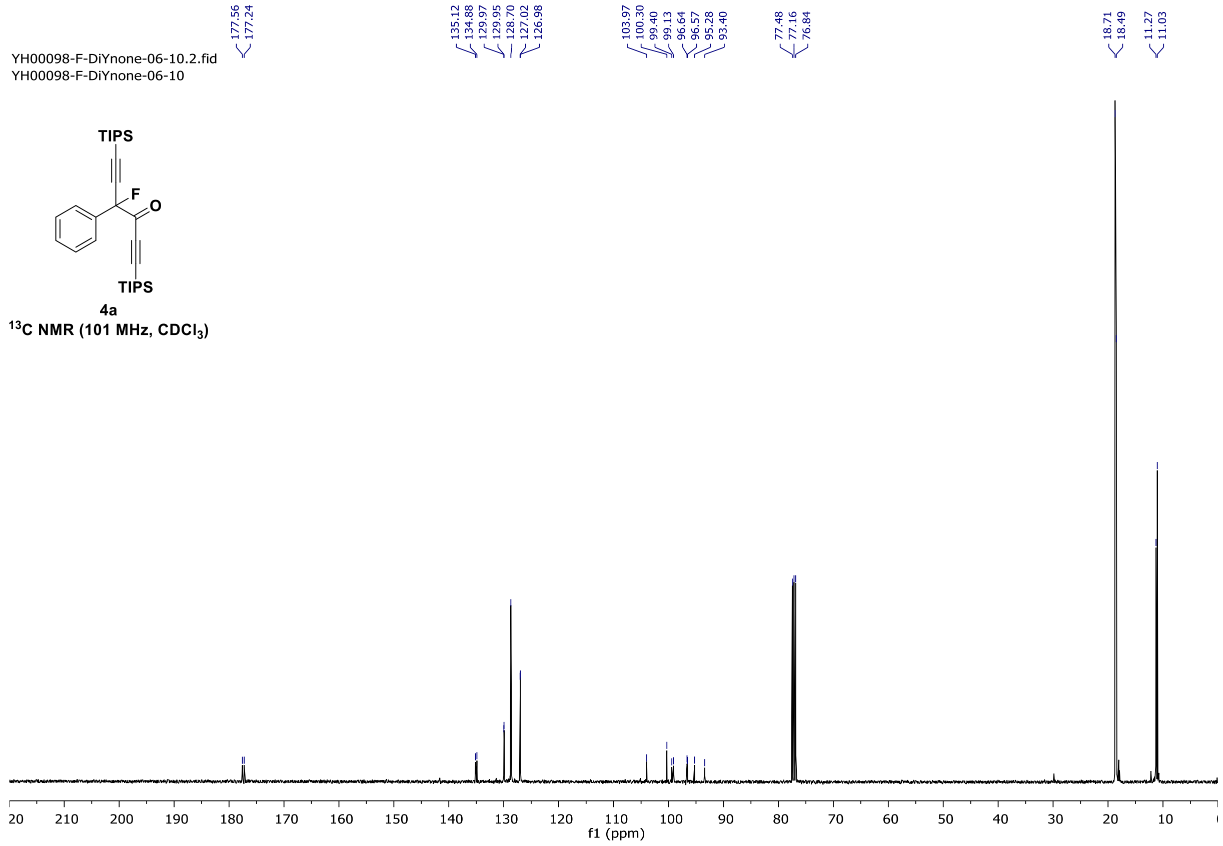

Supplementary Figure 65. ^{13}C NMR of the **4a** (101 MHz, $CDCl_3$)

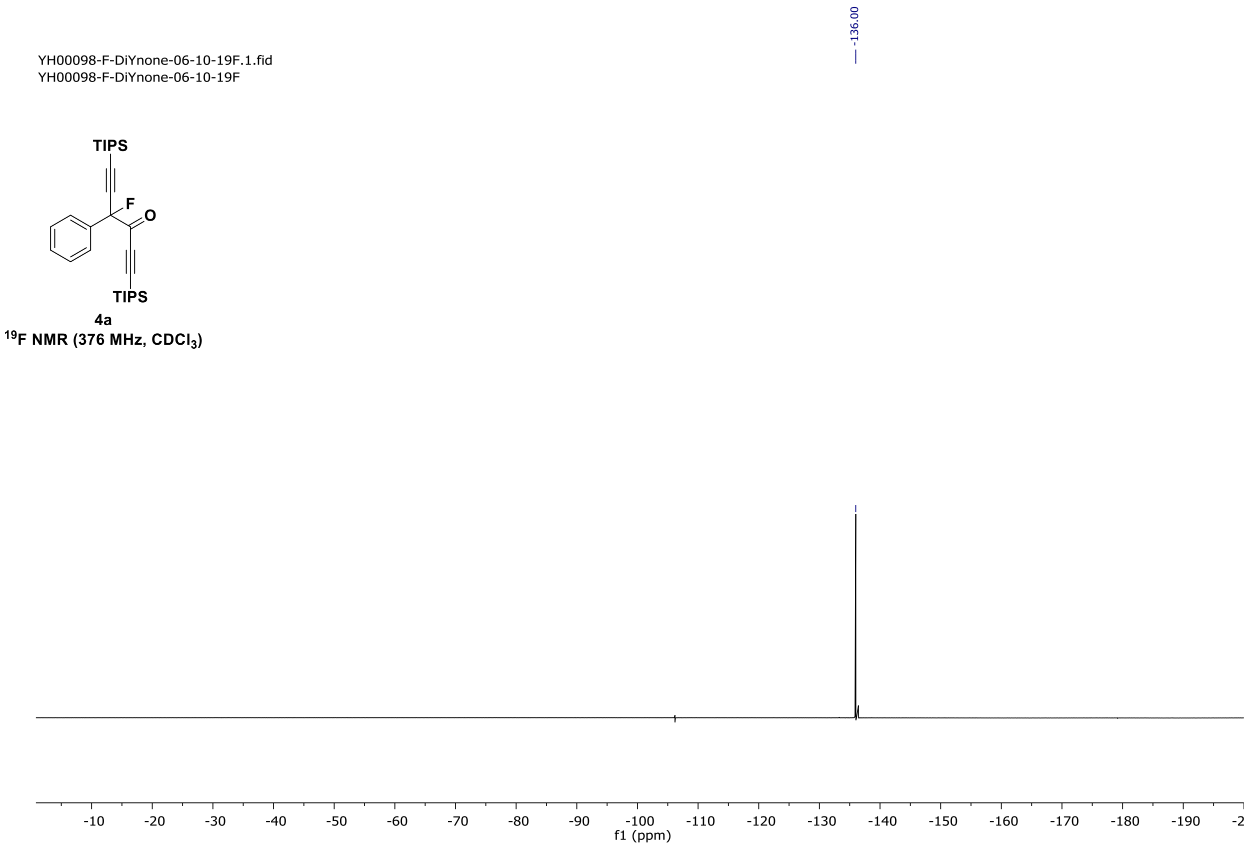

Supplementary Figure 66. ^{19}F NMR of the **4a** (376 MHz, $CDCl_3$)

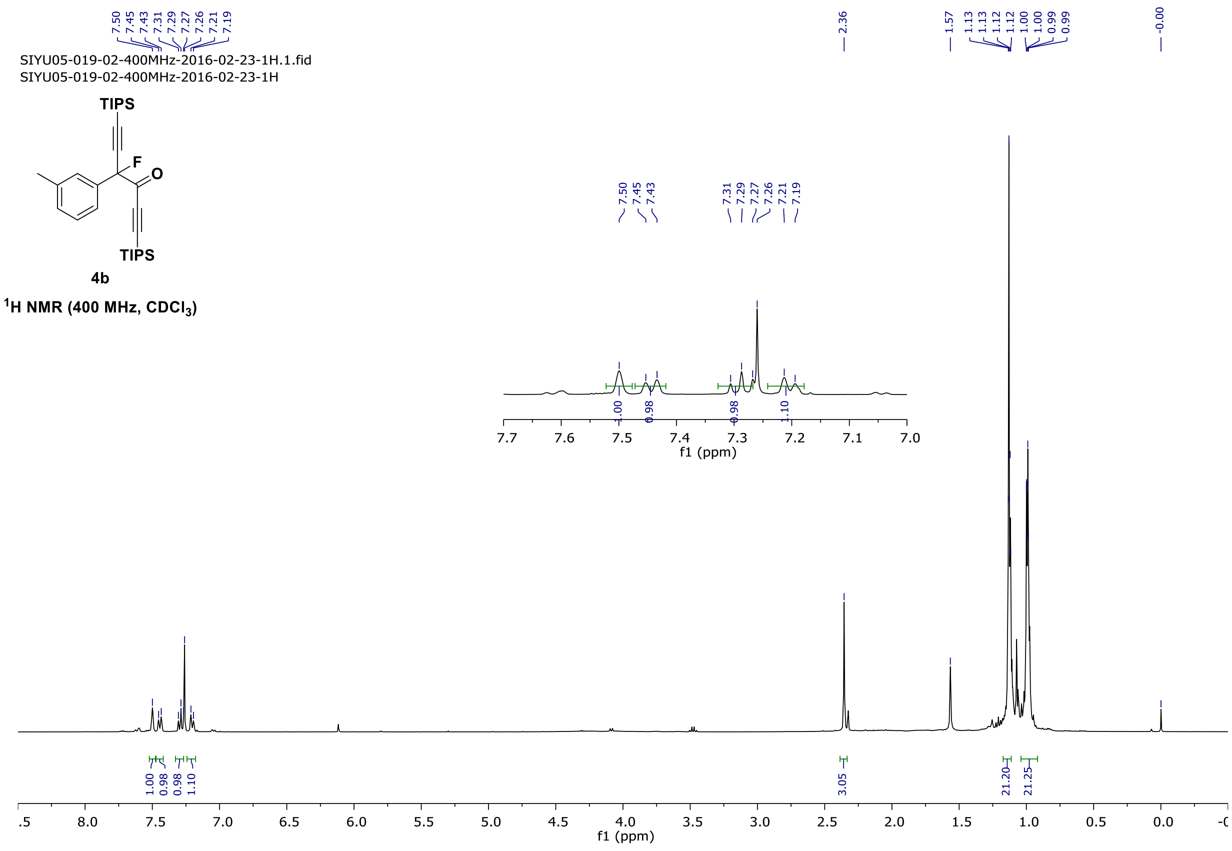

Supplementary Figure 67. ^{1}H NMR of the **4b** (400 MHz, $CDCl_3$)

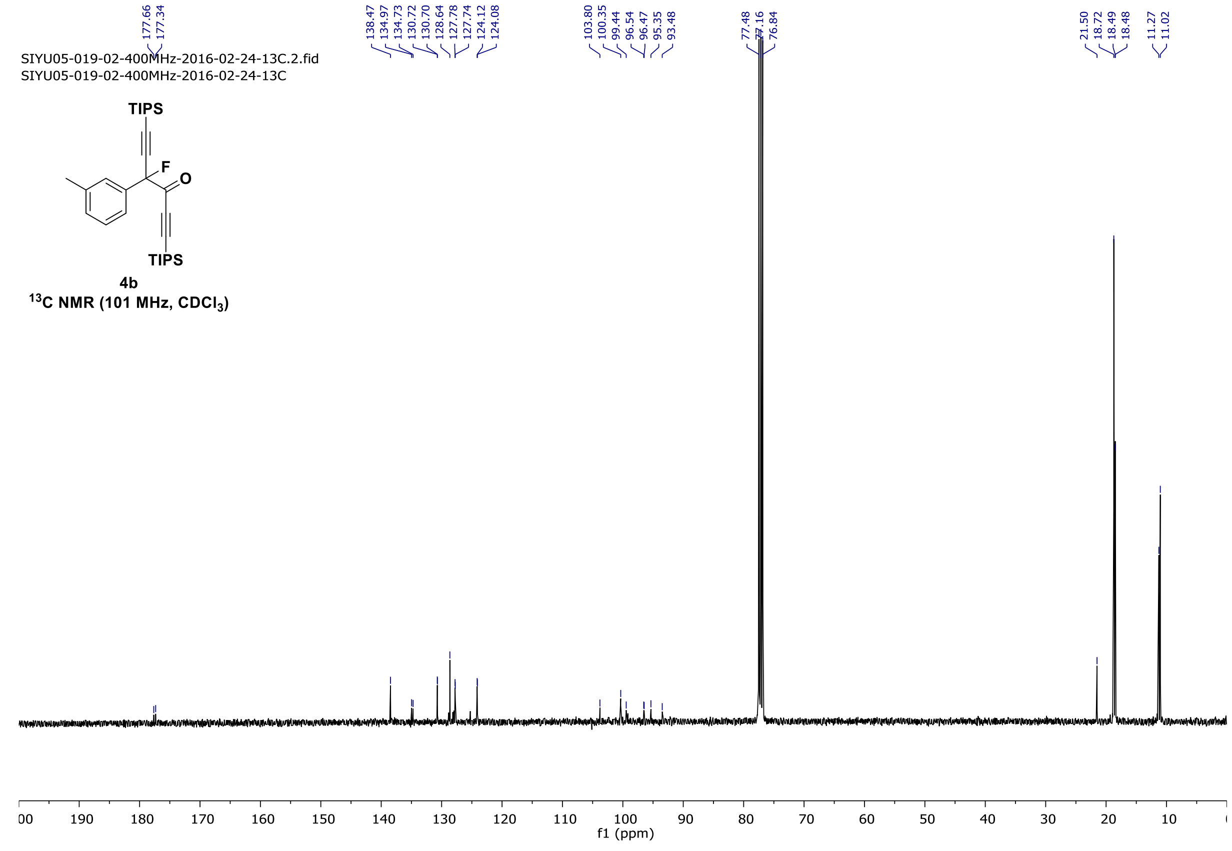

Supplementary Figure 68. ^{13}C NMR of the **4b** (101 MHz, $CDCl_3$)

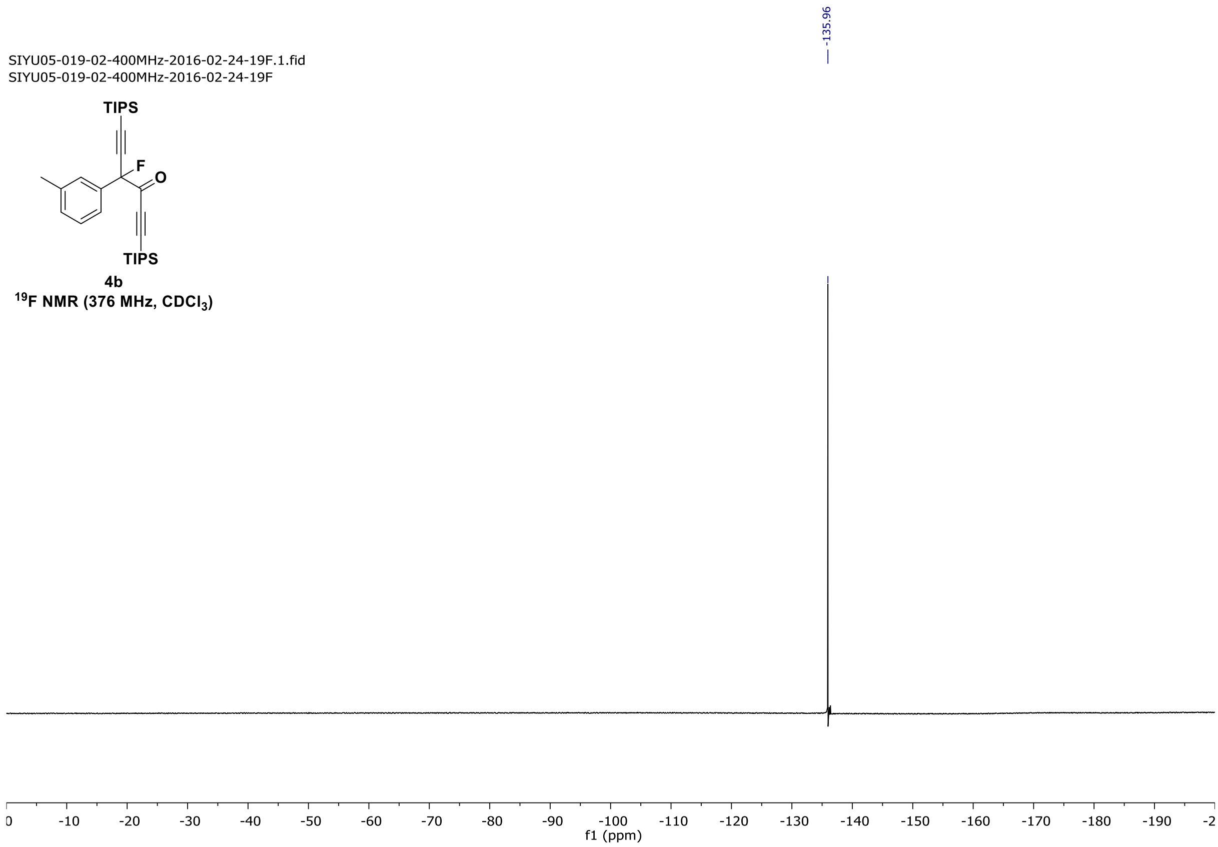

Supplementary Figure 69. ^{19}F NMR of the **4b** (376 MHz, $CDCl_3$)

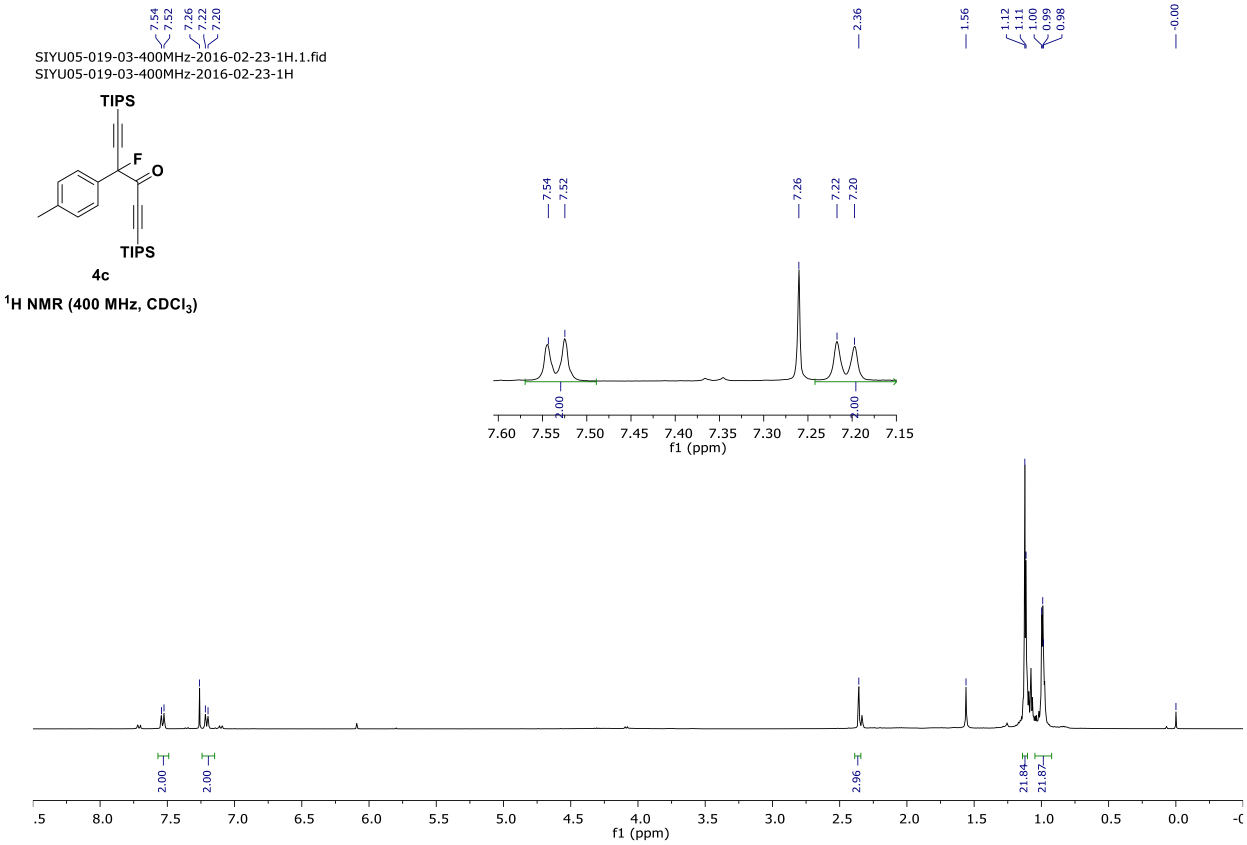

Supplementary Figure 70. ^{1}H NMR of the **4c** (400 MHz, $CDCl_3$)

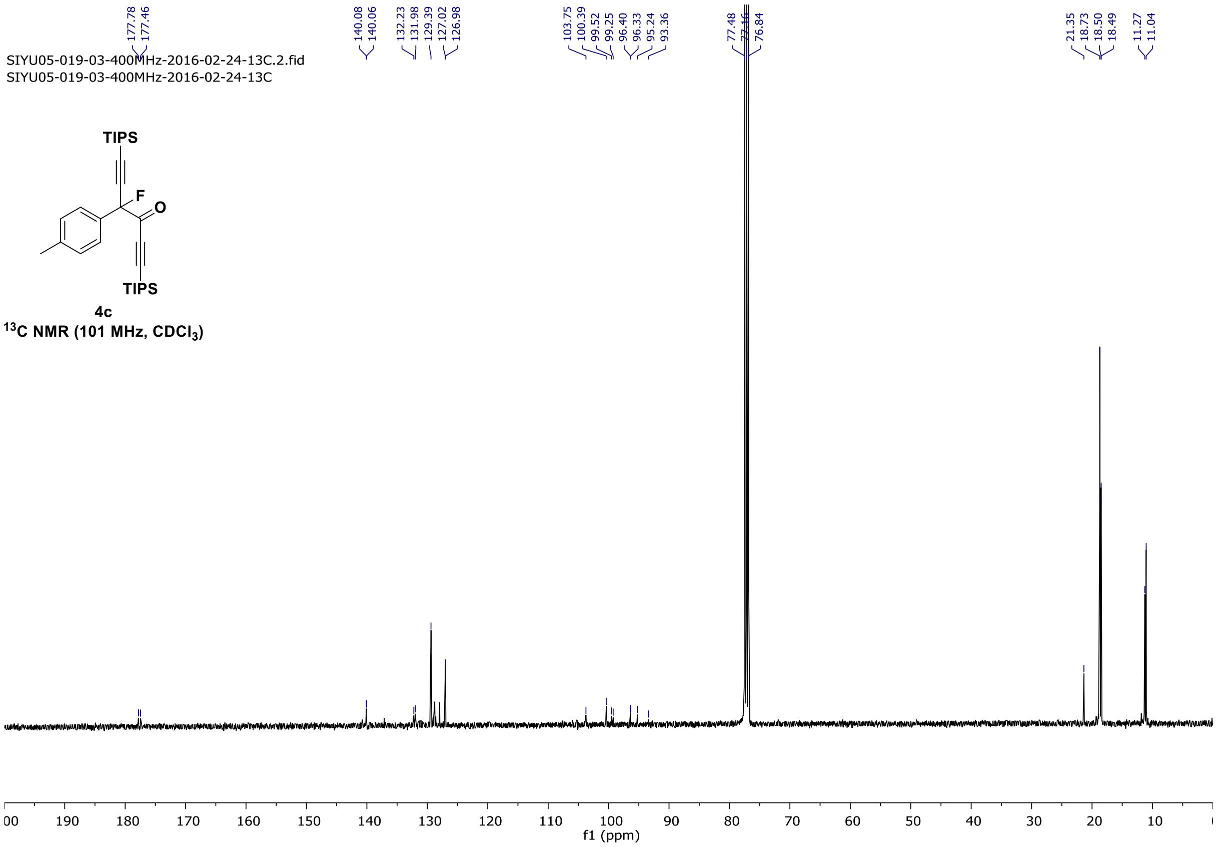

Supplementary Figure 71. ^{13}C NMR of the **4c** (101 MHz, $CDCl_3$)

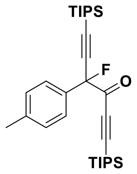

4c

^{19}F NMR (376 MHz, $CDCl_3$)

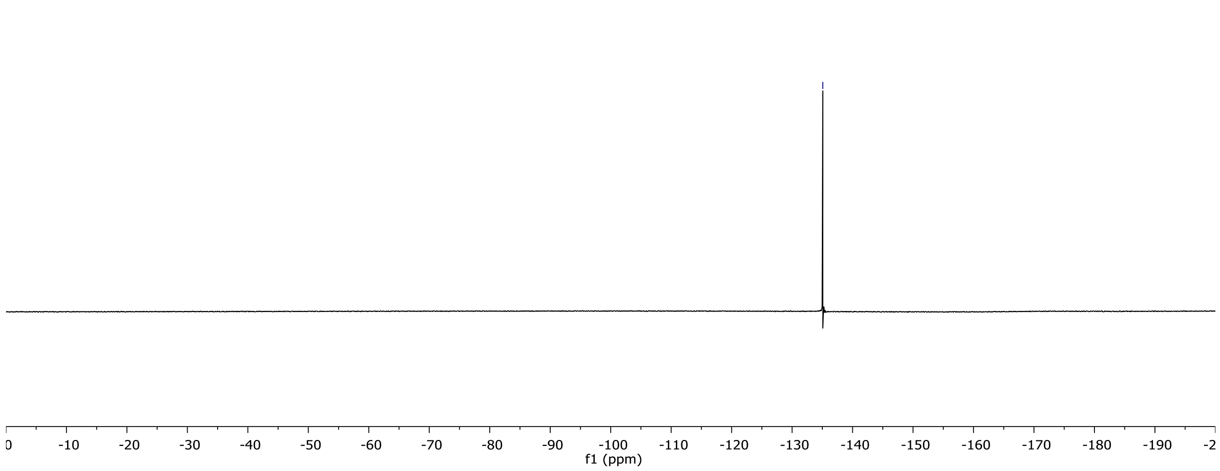

Supplementary Figure 72. ^{19}F NMR of the **4c** (376 MHz, $CDCl_3$)

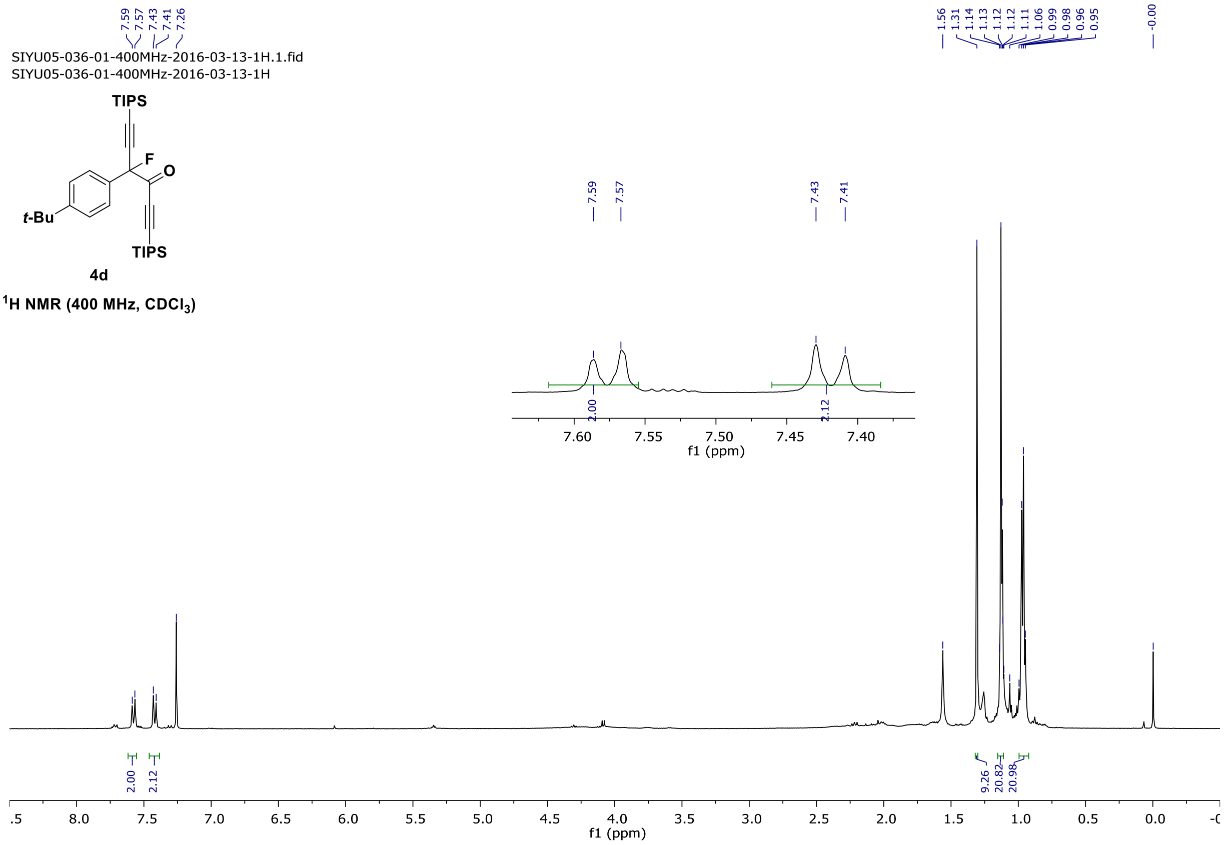

Supplementary Figure 73. ^{1}H NMR of the **4d** (400 MHz, $CDCl_3$)

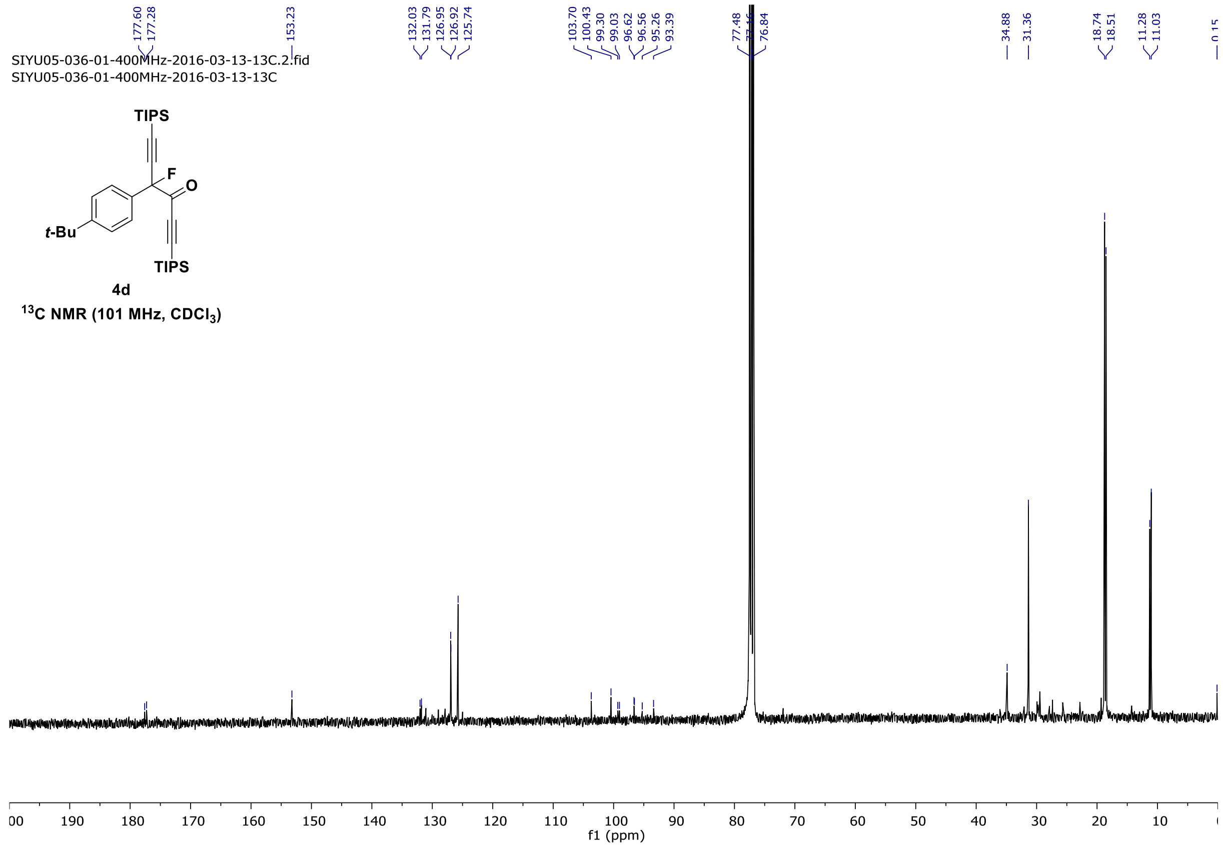

Supplementary Figure 74. ^{13}C NMR of the **4d** (101 MHz, $CDCl_3$)

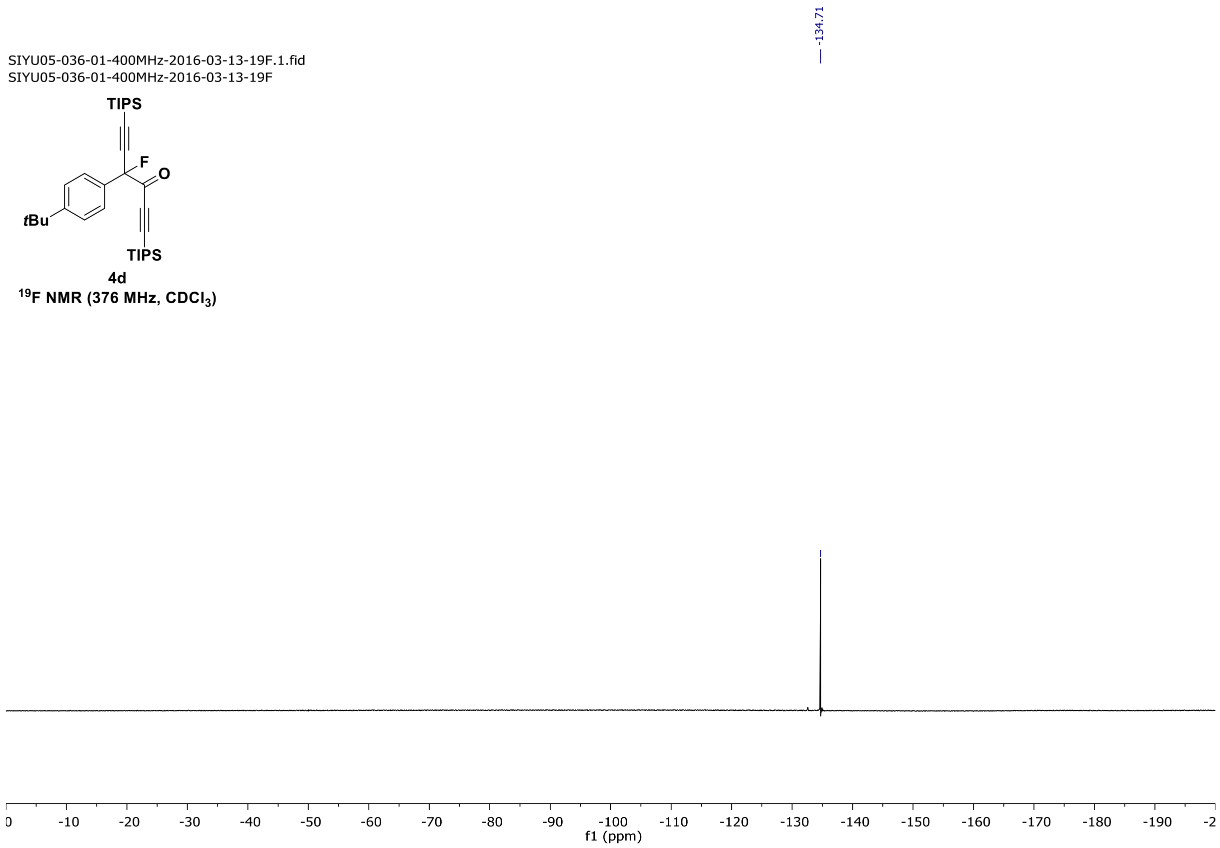

Supplementary Figure 75. ^{19}F NMR of the **4d** (376 MHz, $CDCl_3$)

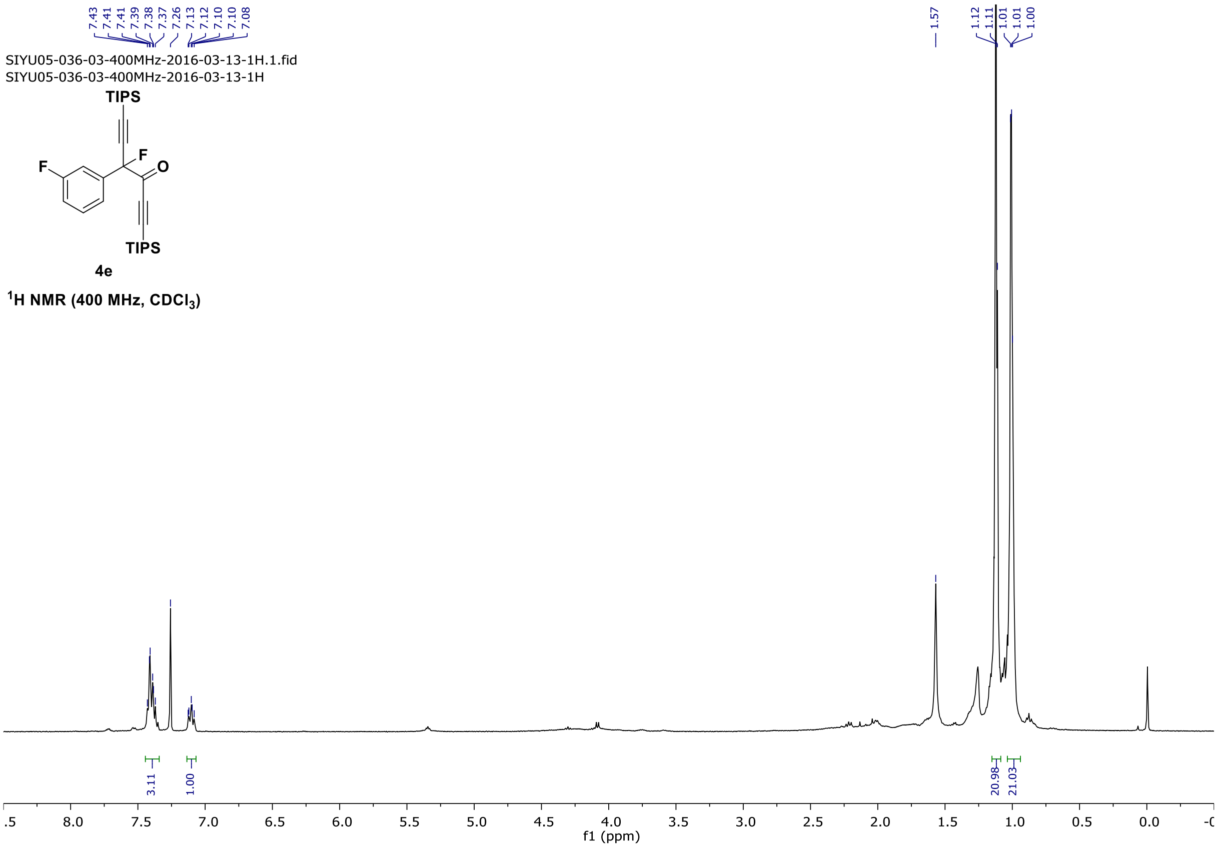

Supplementary Figure 76. ^{1}H NMR of the **4e** (400 MHz, $CDCl_3$)

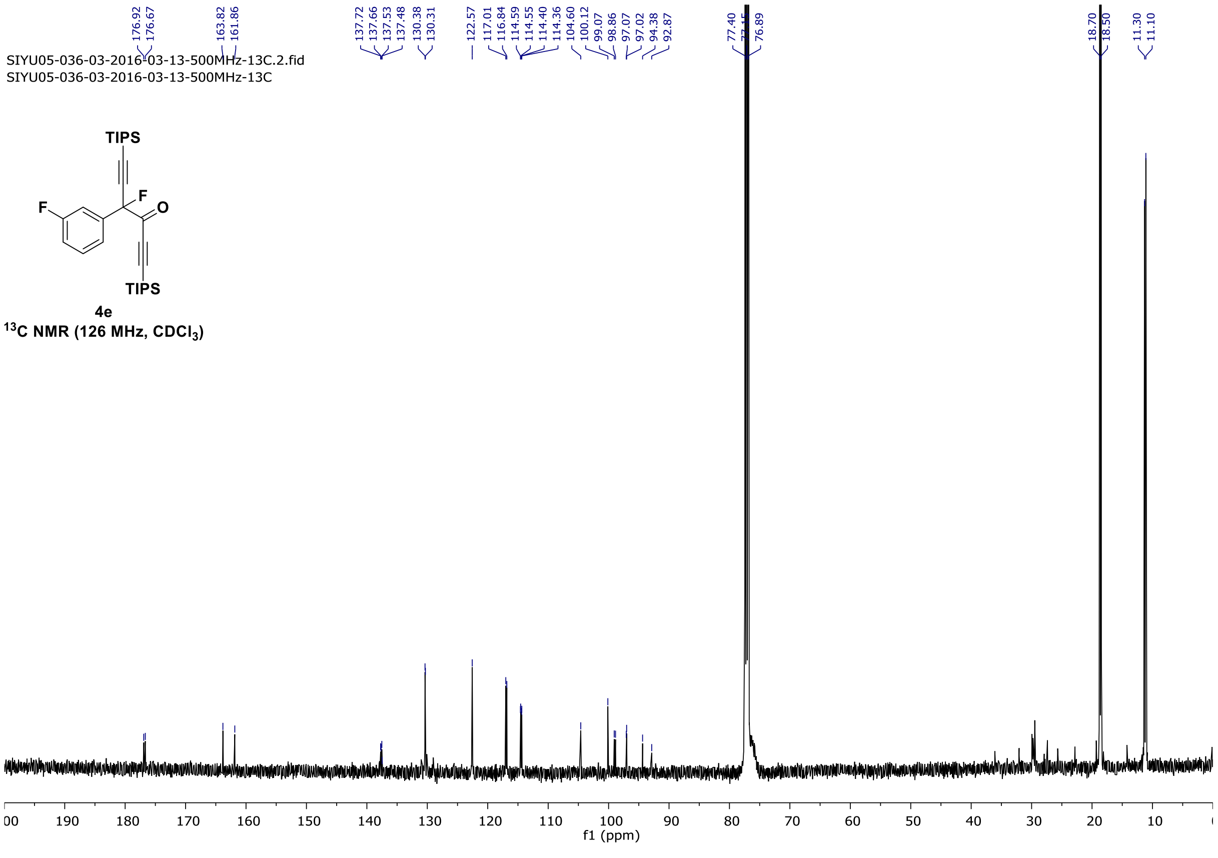

Supplementary Figure 77. ^{13}C NMR of the **4e** (126 MHz, $CDCl_3$)

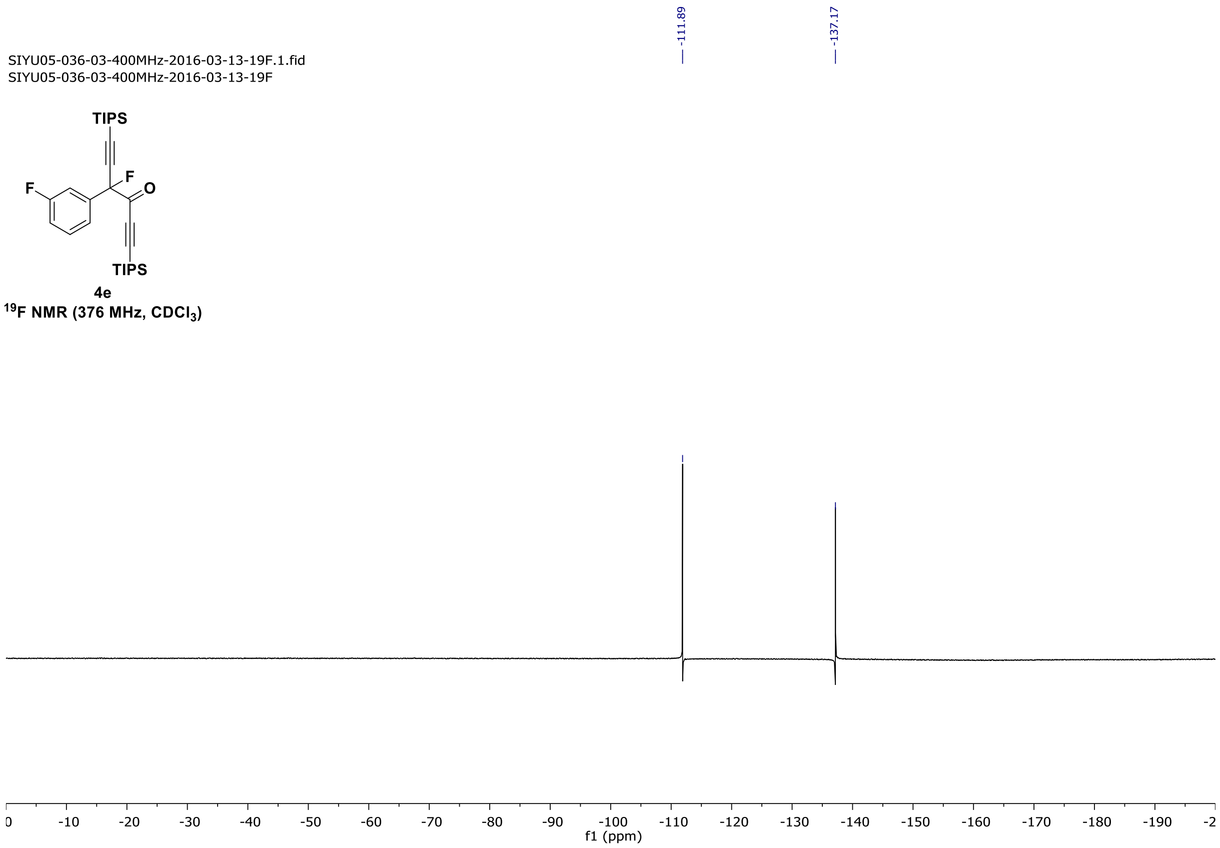

Supplementary Figure 78. ^{19}F NMR of the **4e** (376 MHz, $CDCl_3$)

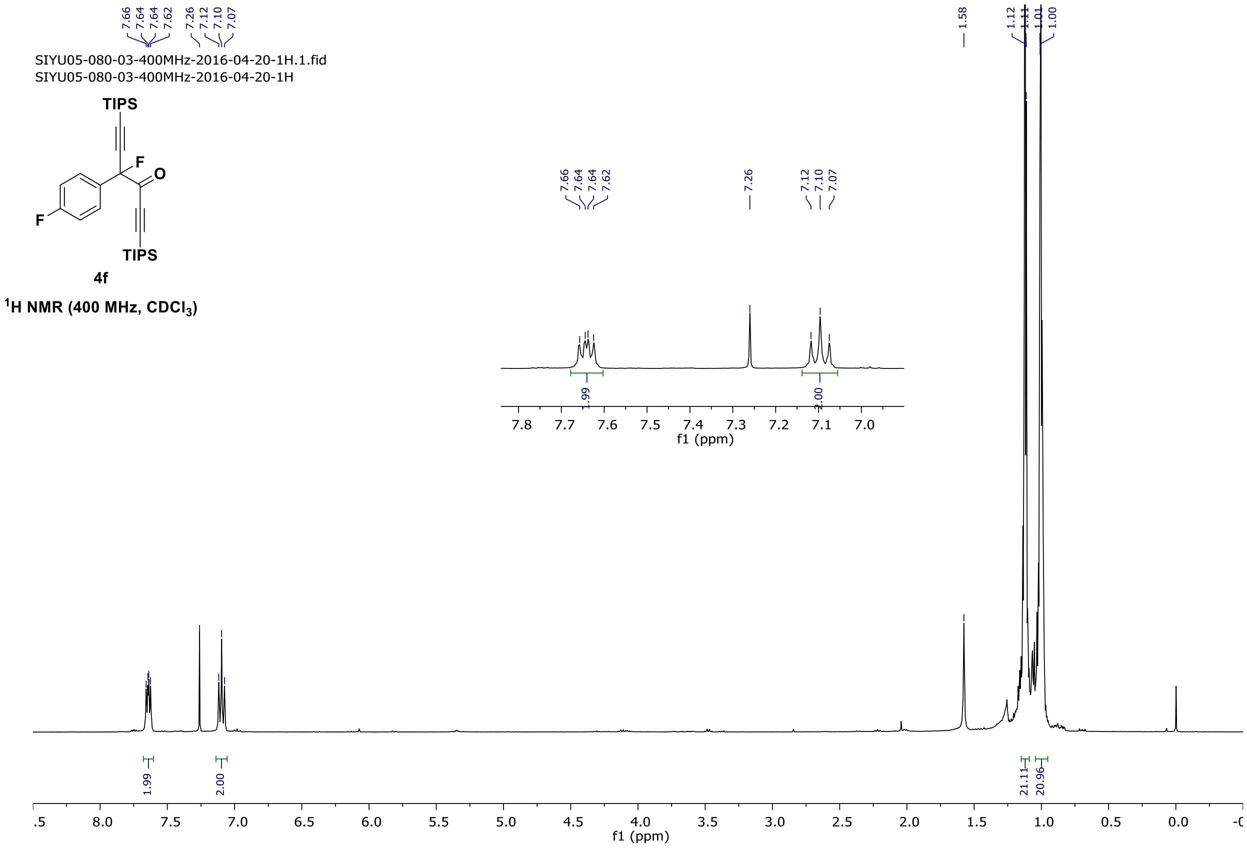

Supplementary Figure 79. ^{1}H NMR of the **4f** (400 MHz, $CDCl_3$)

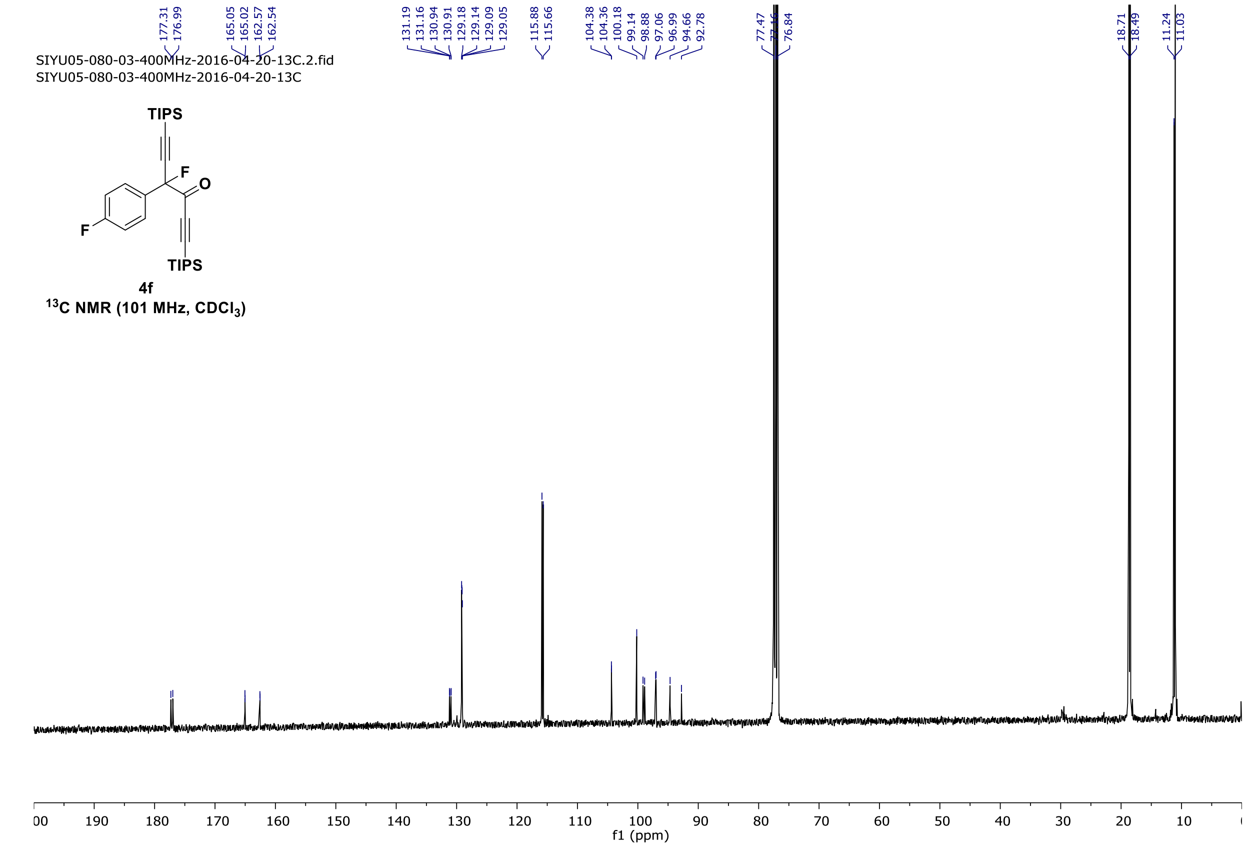

Supplementary Figure 80. ^{13}C NMR of the **4f** (101 MHz, $CDCl_3$)

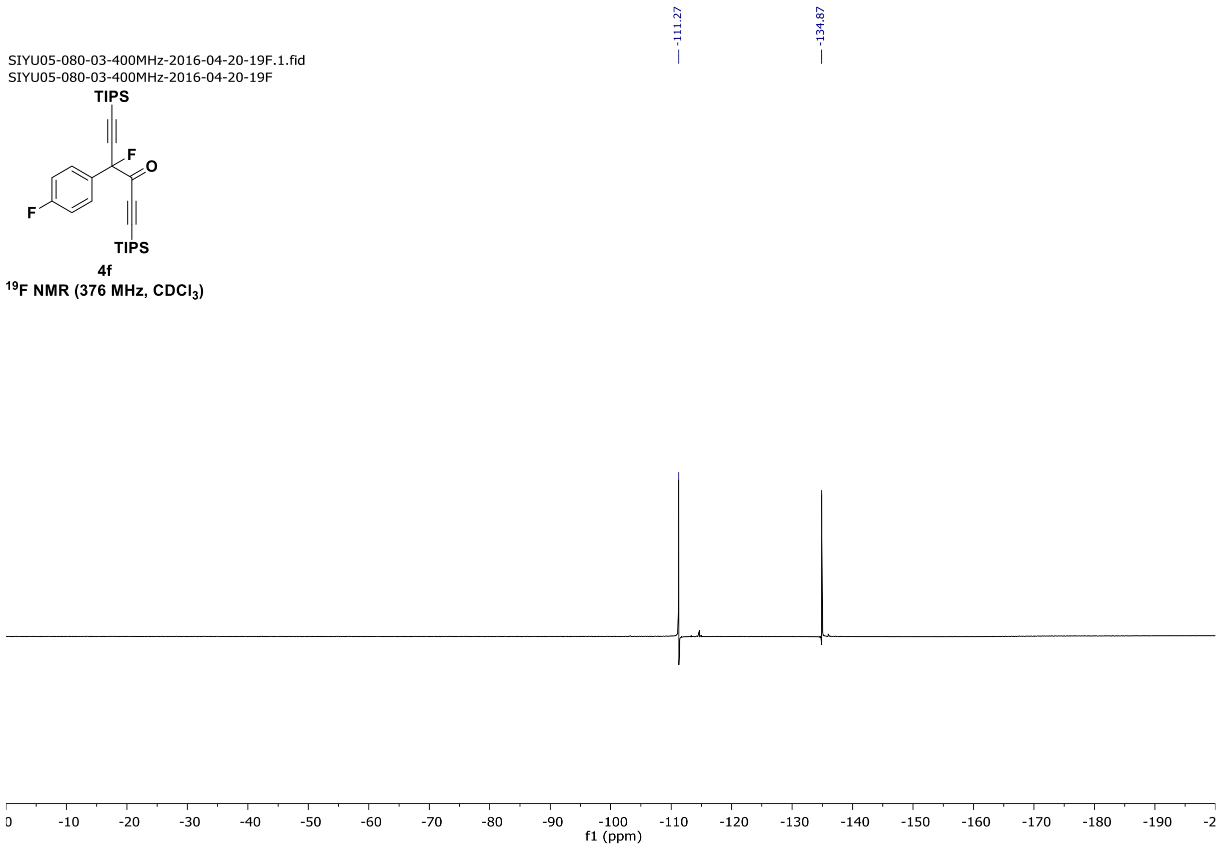

Supplementary Figure 81. ^{19}F NMR of the **4f** (376 MHz, $CDCl_3$)

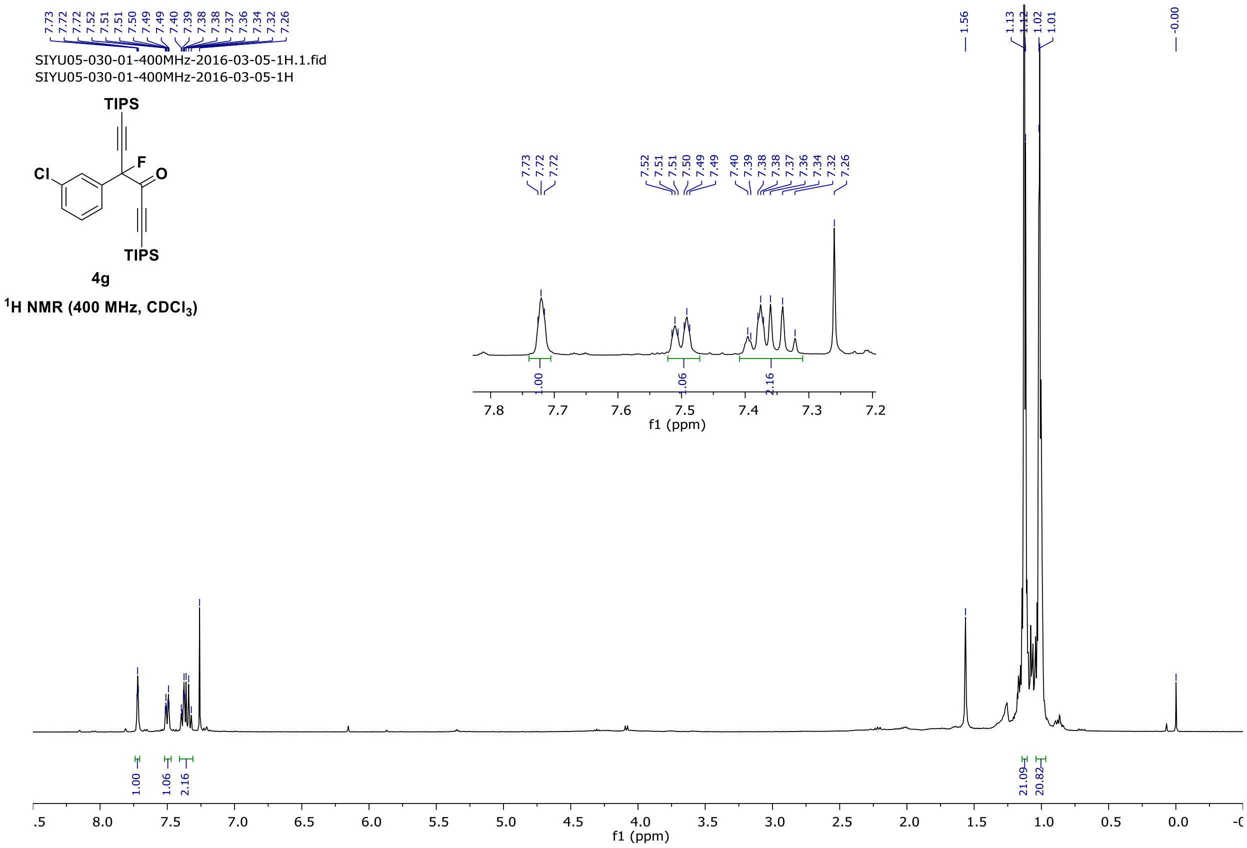

Supplementary Figure 82. ^{1}H NMR of the **4g** (400 MHz, $CDCl_3$)

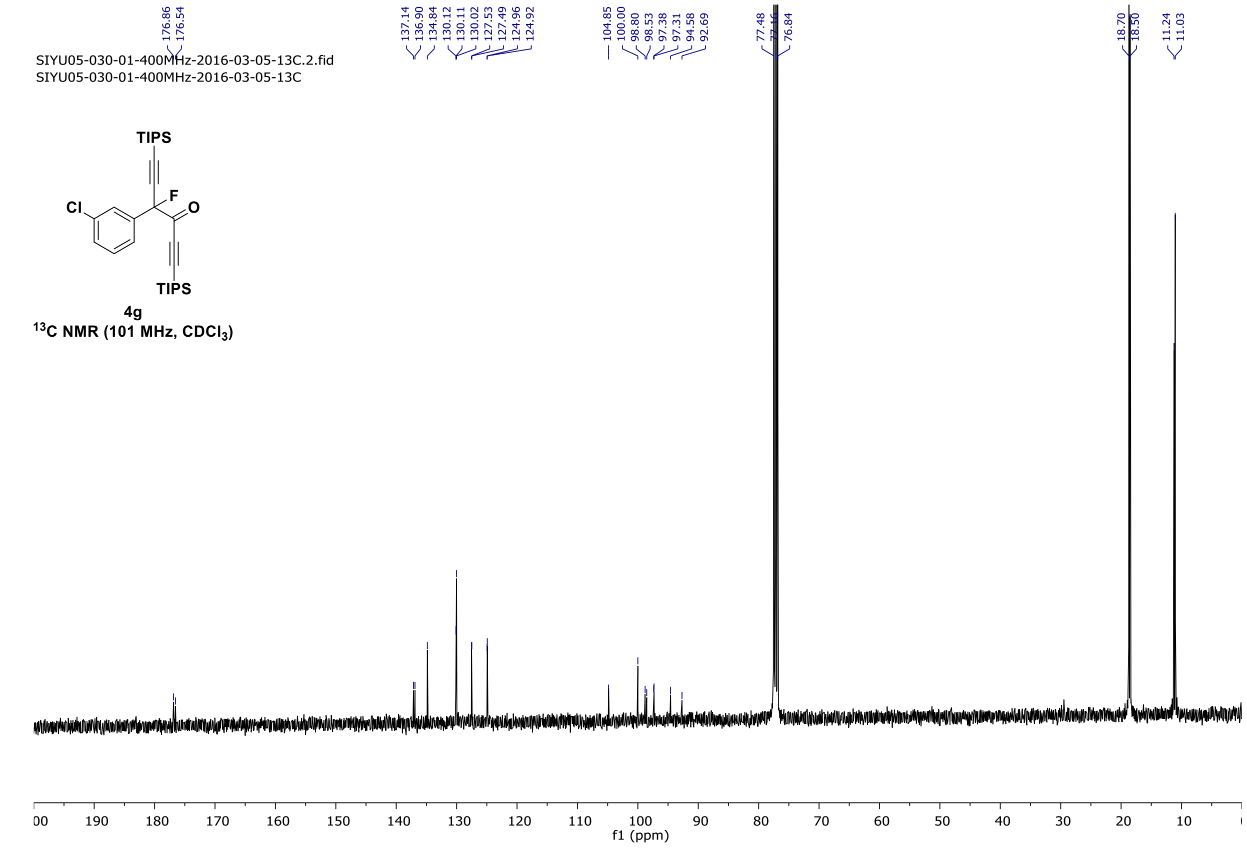

Supplementary Figure 83. ^{13}C NMR of the **4g** (101 MHz, $CDCl_3$)

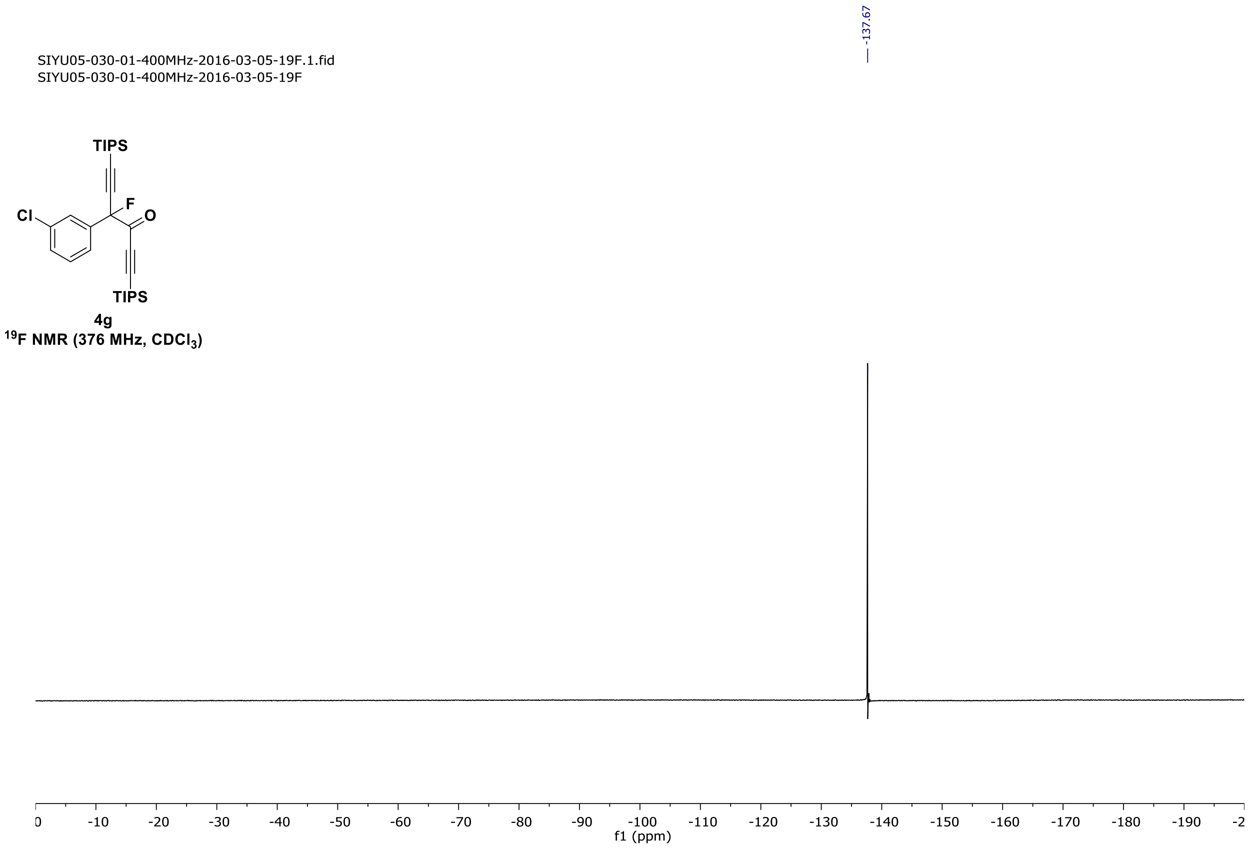

Supplementary Figure 84. ^{19}F NMR of the **4g** (376 MHz, $CDCl_3$)

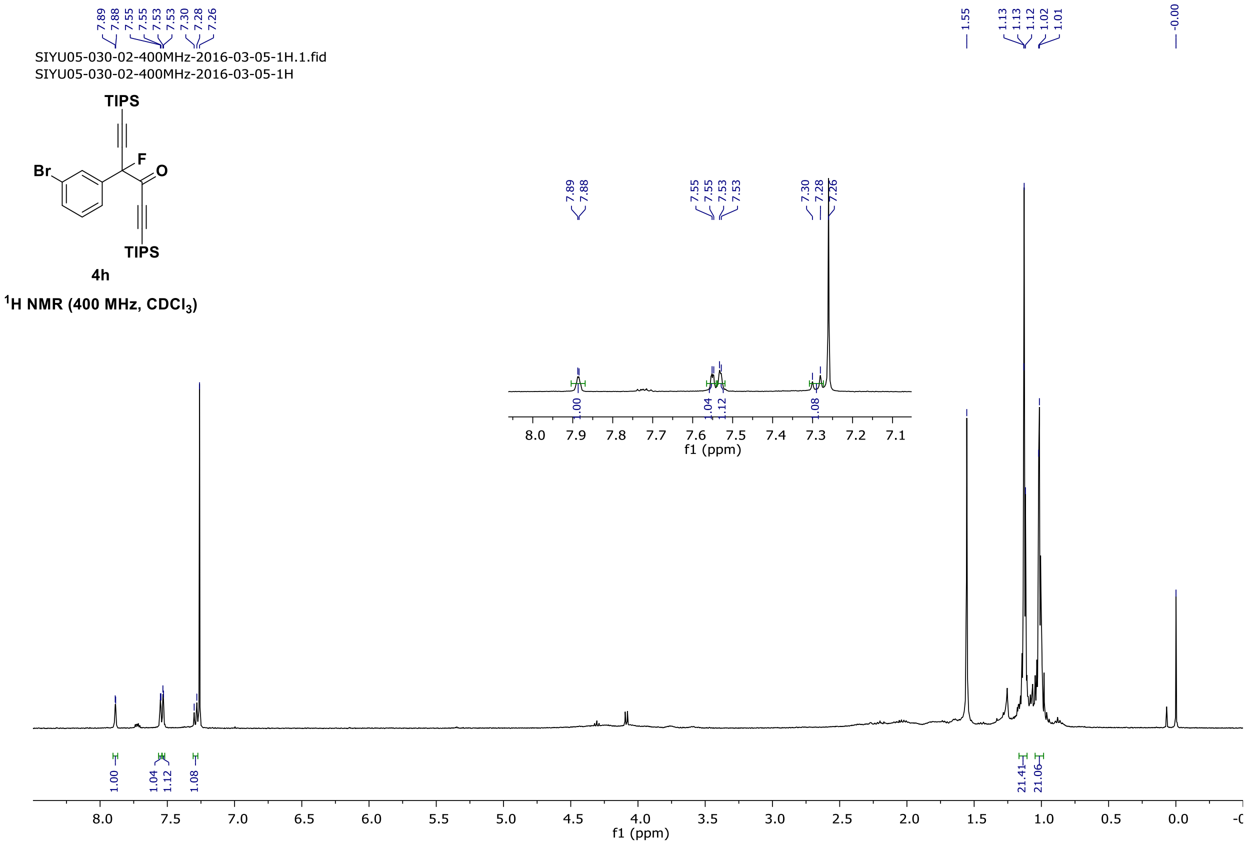

Supplementary Figure 85. ^{1}H NMR of the **4h** (400 MHz, $CDCl_3$)

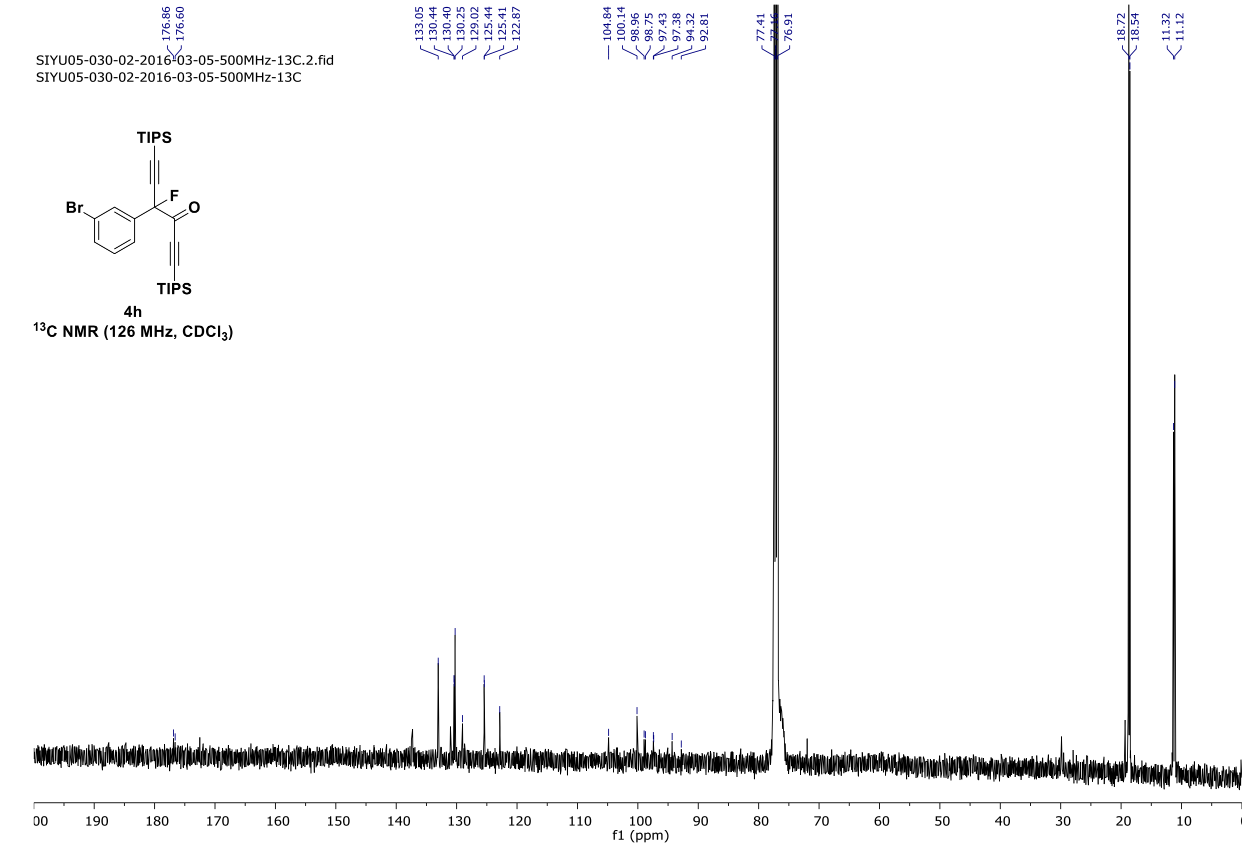

Supplementary Figure 85. ^{1}H NMR of the **4h** (400 MHz, $CDCl_3$)

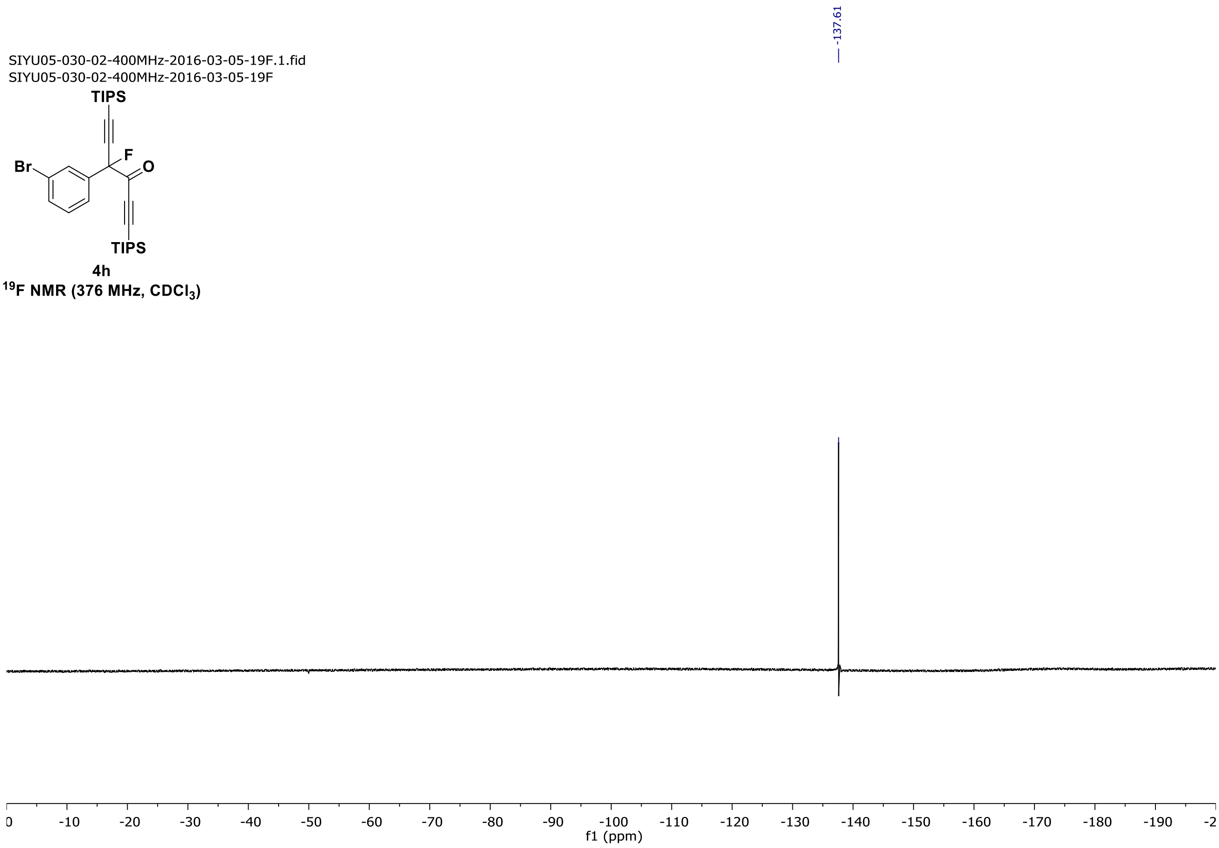

Supplementary Figure 87. ^{19}F NMR of the **4h** (376 MHz, $CDCl_3$)

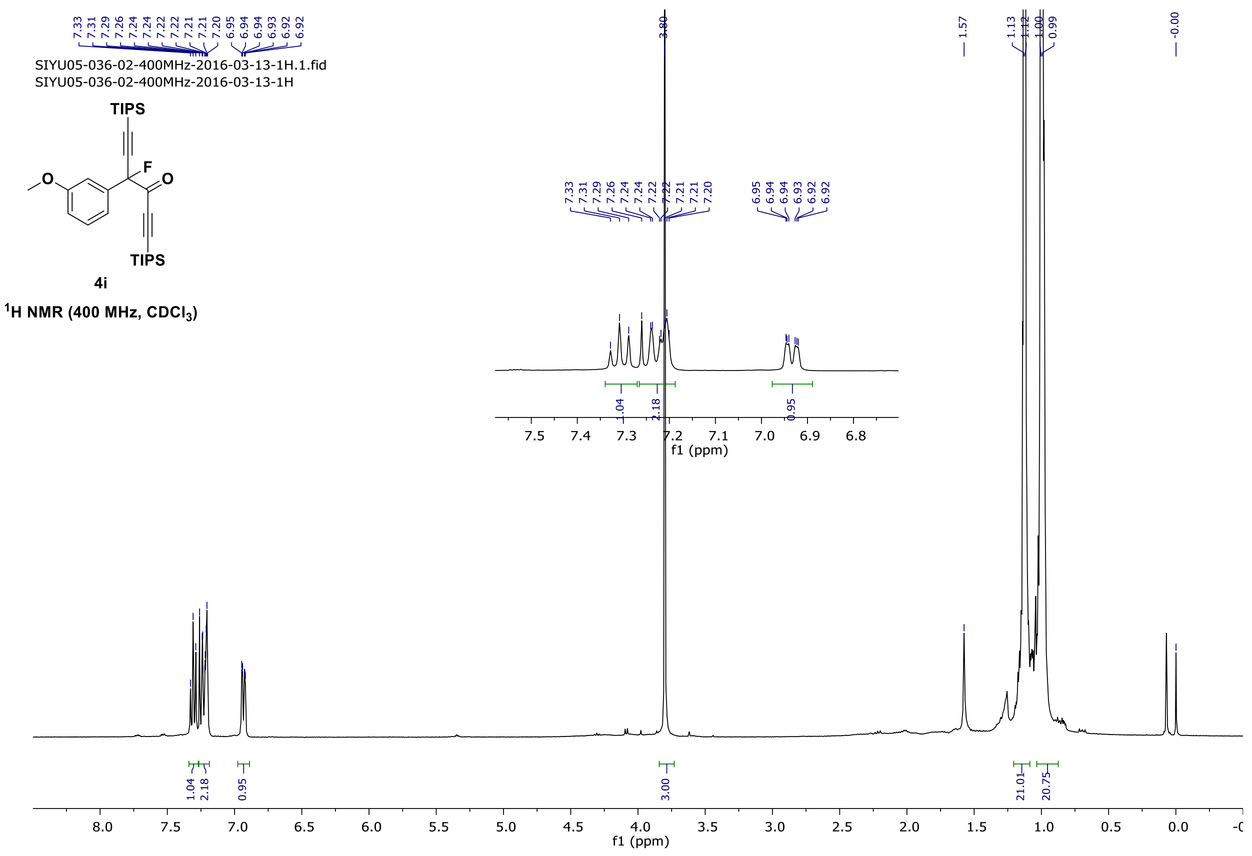

Supplementary Figure 88. ^{1}H NMR of the **4i** (400 MHz, $CDCl_3$)

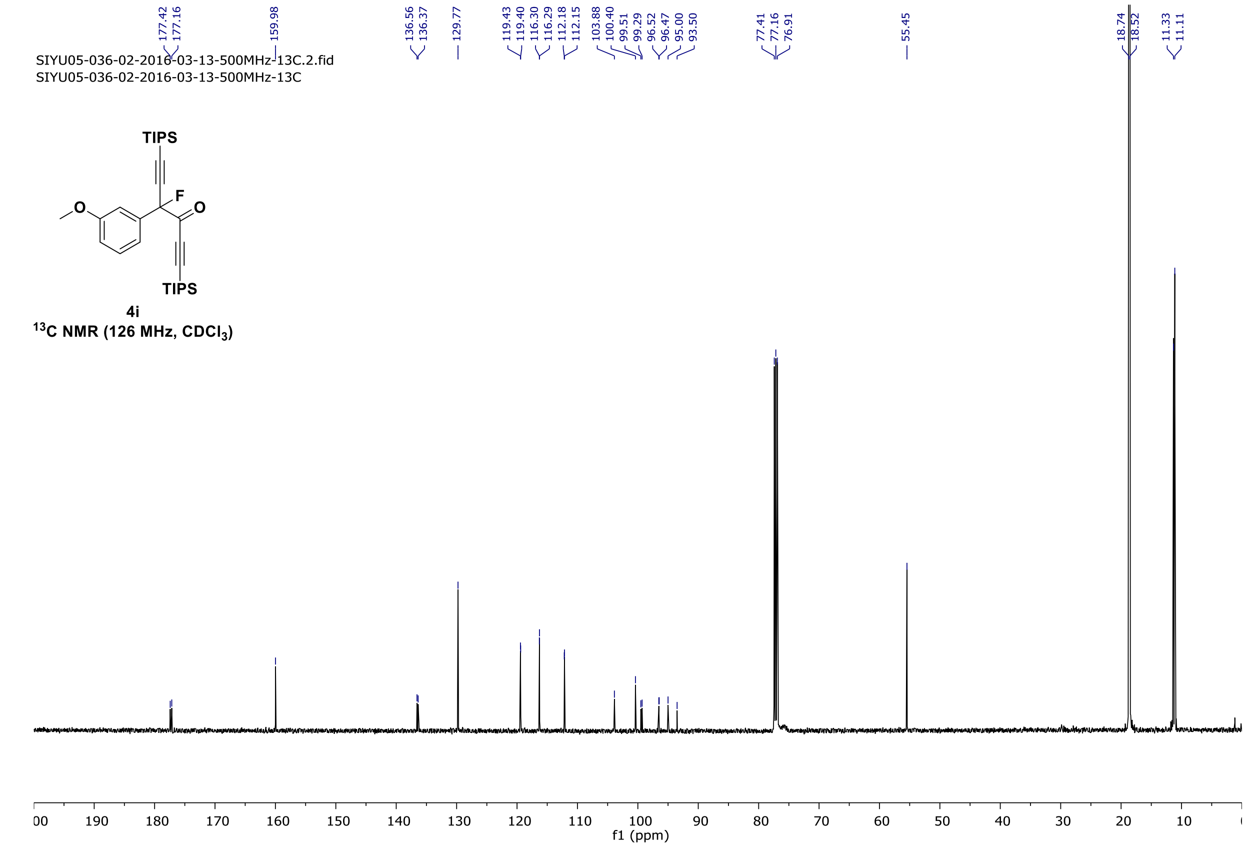

Supplementary Figure 89. ^{13}C NMR of the **4i** (126 MHz, $CDCl_3$)

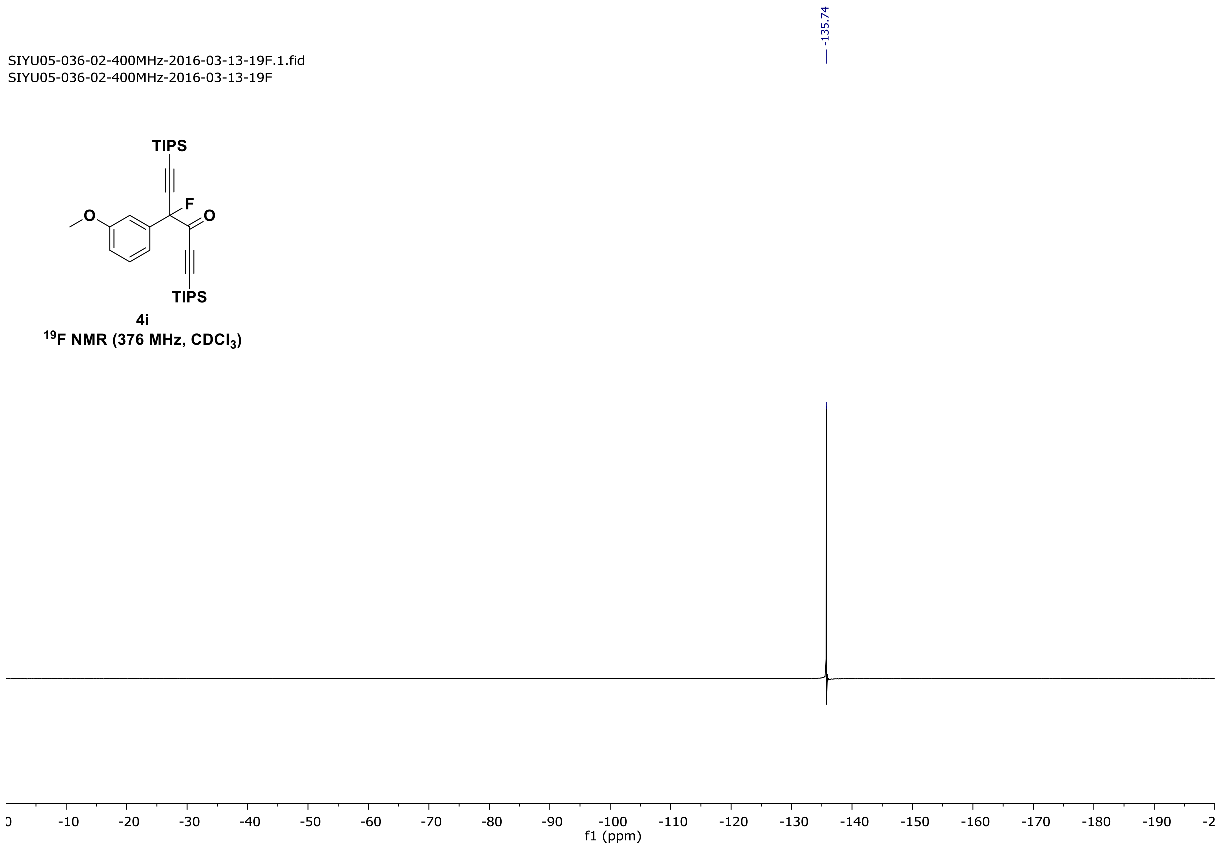

Supplementary Figure 90. ^{19}F NMR of the **4i** (376 MHz, $CDCl_3$)

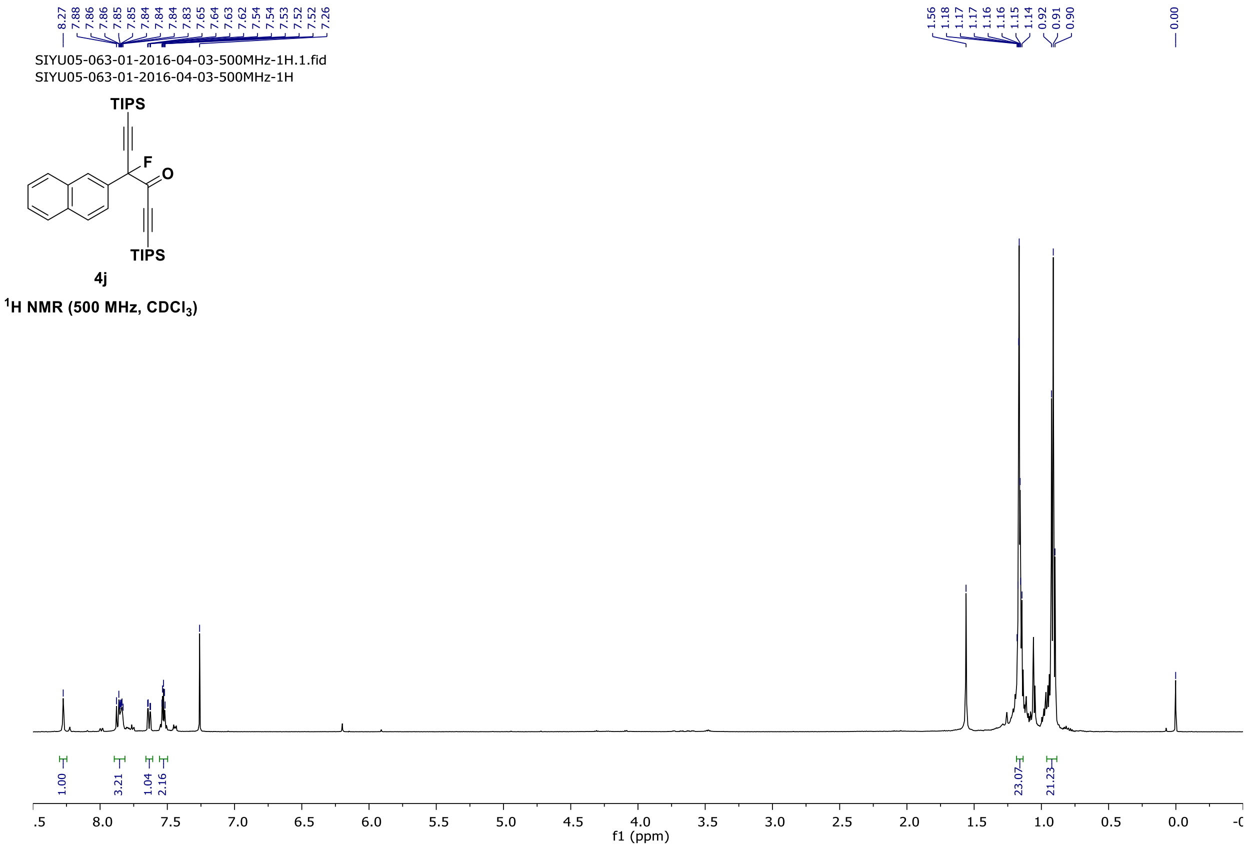

Supplementary Figure 91. ^1H NMR of the **4j** (500 MHz, $CDCl_3$)

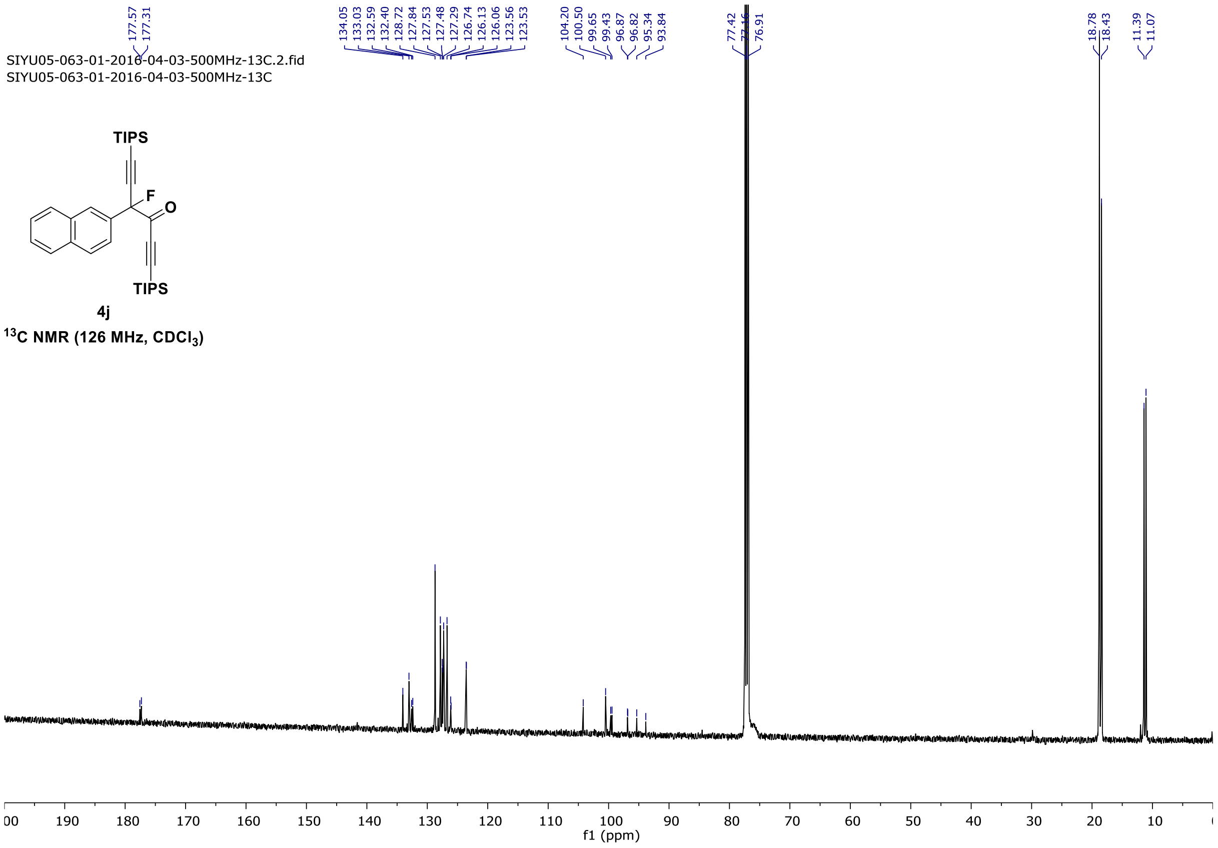

Supplementary Figure 92. ^{13}C NMR of the **4j** (126 MHz, $CDCl_3$)

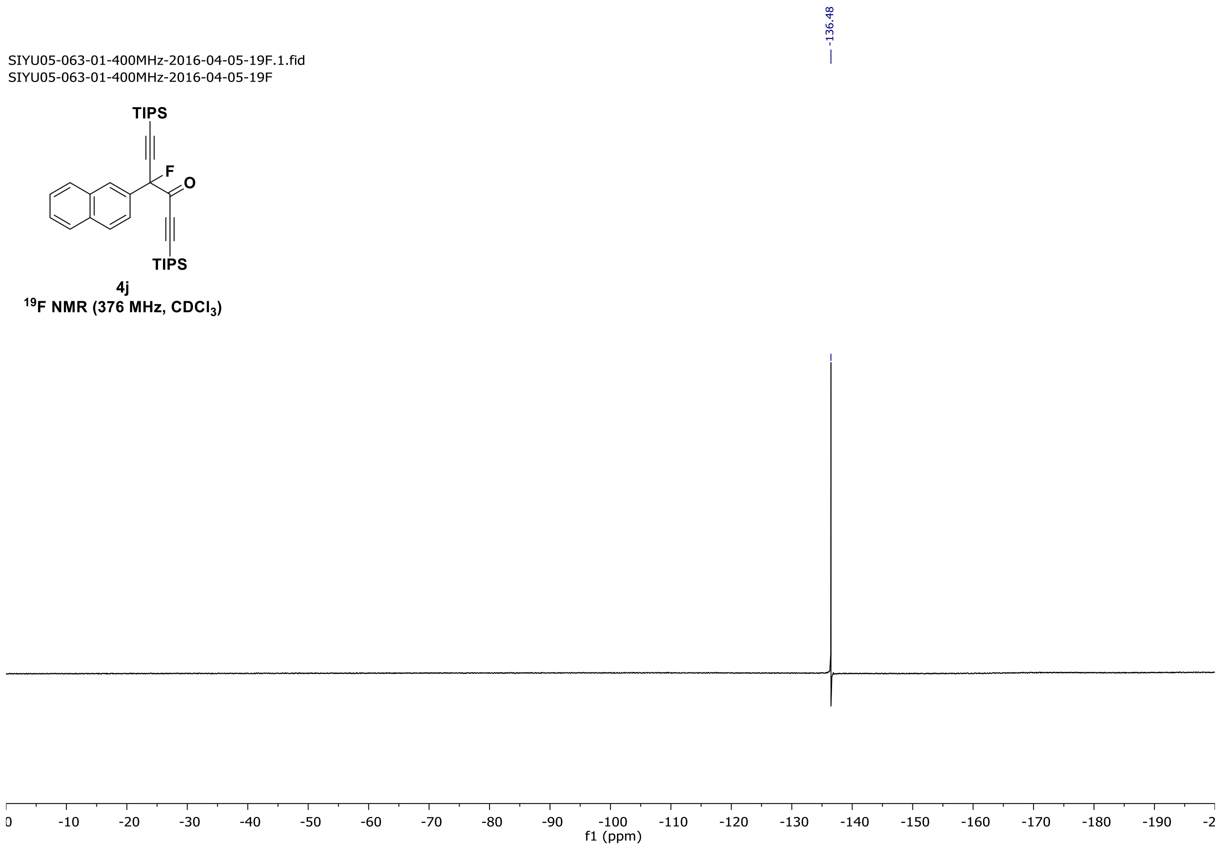

Supplementary Figure 93. ^{19}F NMR of the **4j** (376 MHz, $CDCl_3$)

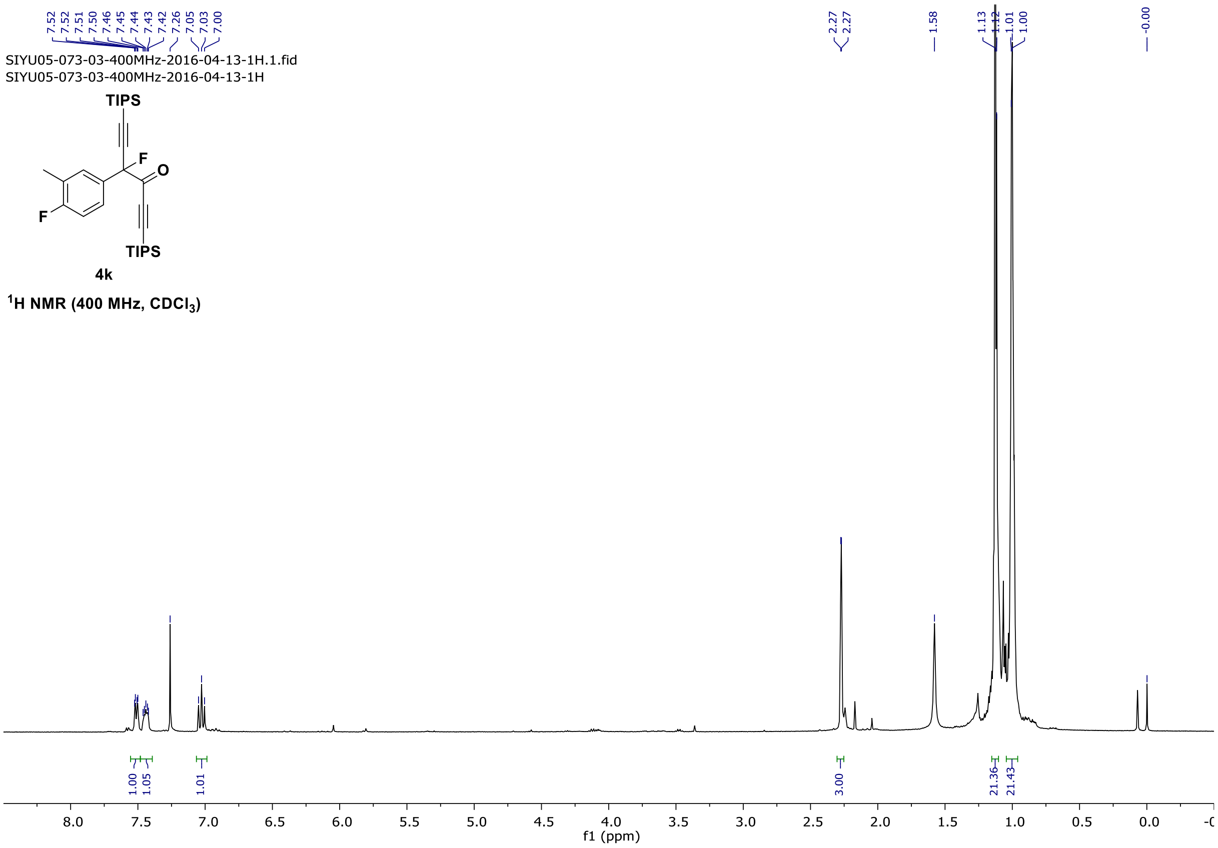

Supplementary Figure 94. ^{1}H NMR of the **4k** (400 MHz, $CDCl_3$)

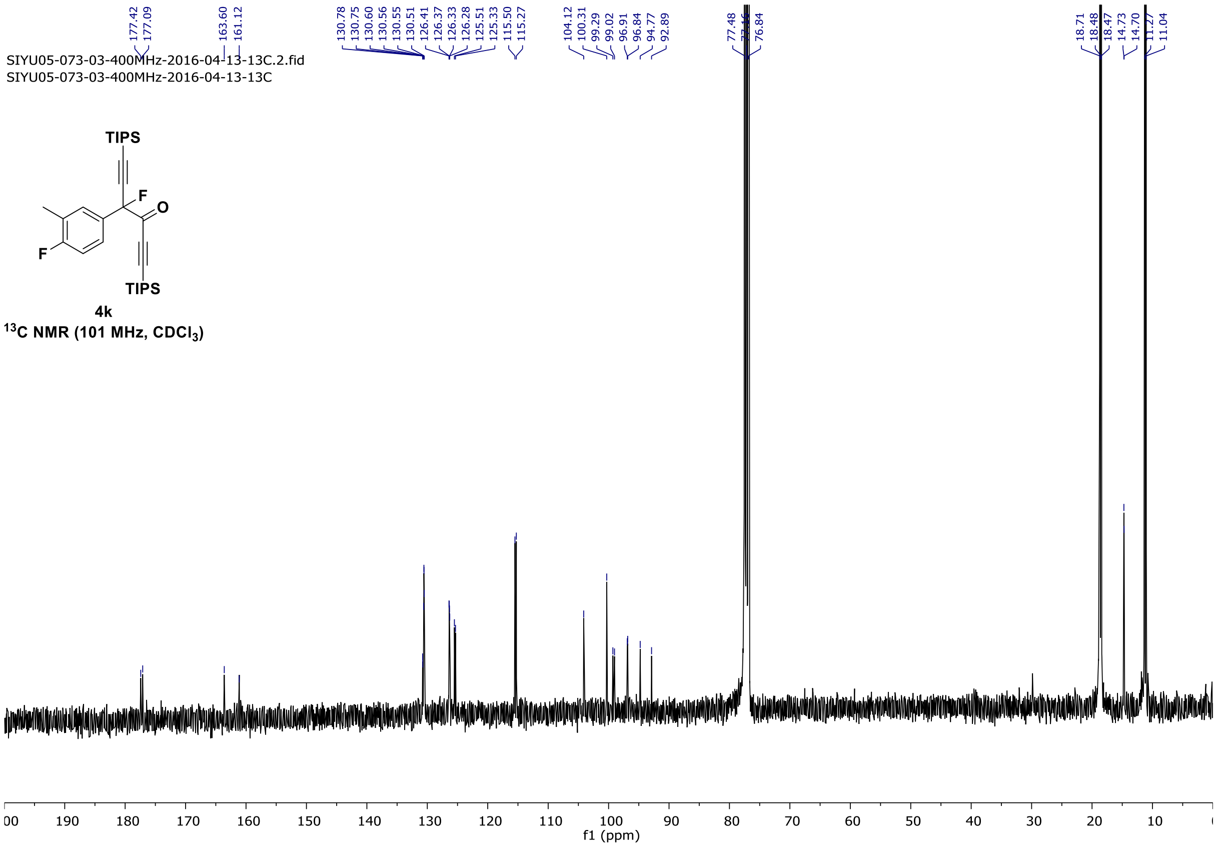

Supplementary Figure 95. ^{13}C NMR of the **4k** (101 MHz, $CDCl_3$)

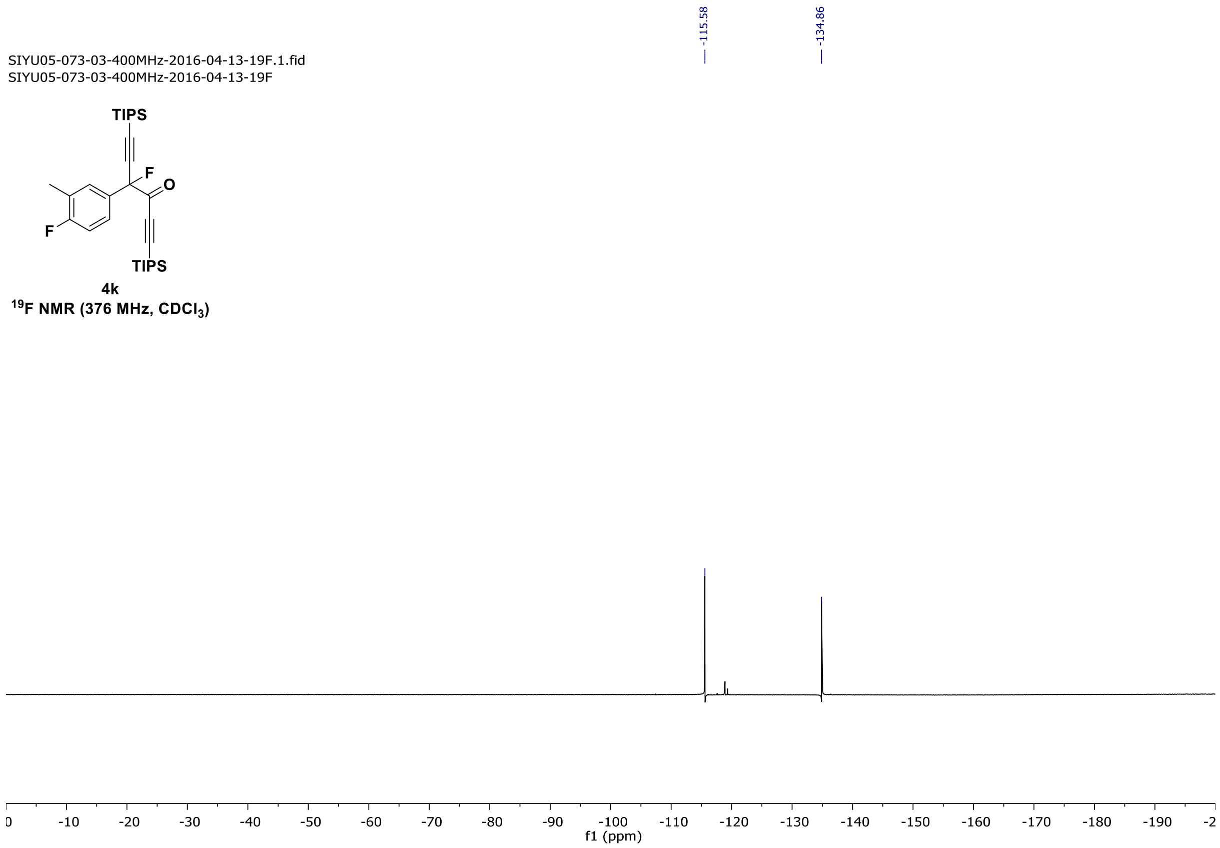

Supplementary Figure 93. ^{19}F NMR of the **4k** (376 MHz, $CDCl_3$)

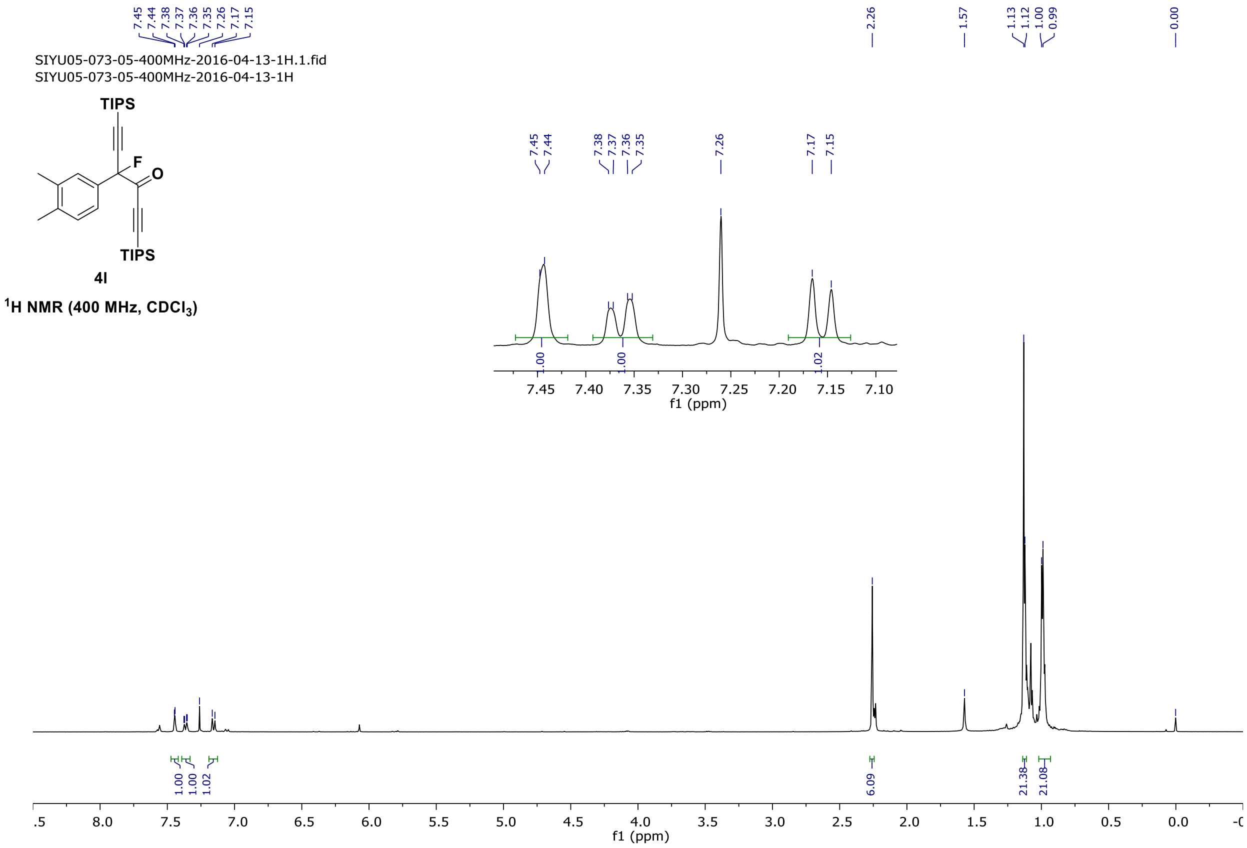

Supplementary Figure 97. ^{1}H NMR of the **4l** (400 MHz, $CDCl_3$)

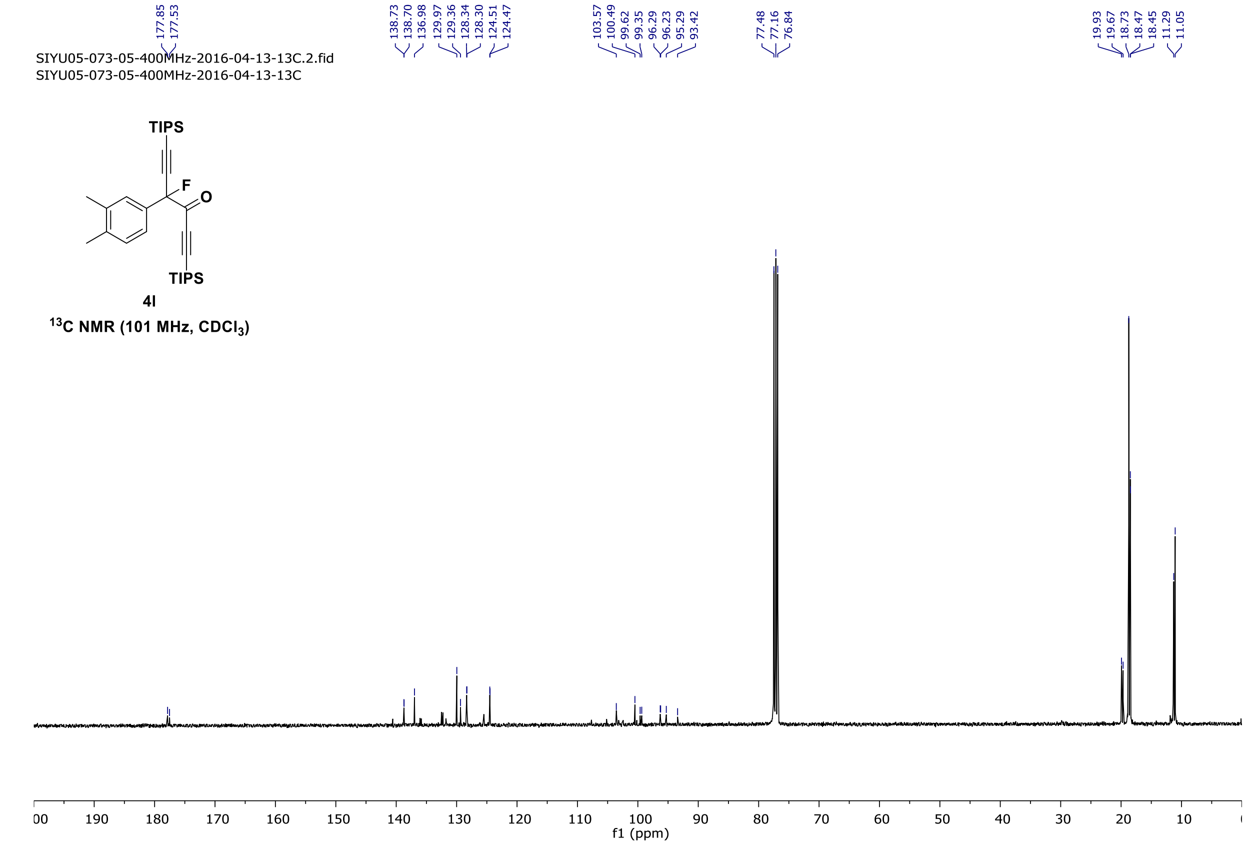

Supplementary Figure 98. ^{13}C NMR of the **4l** (101 MHz, $CDCl_3$)

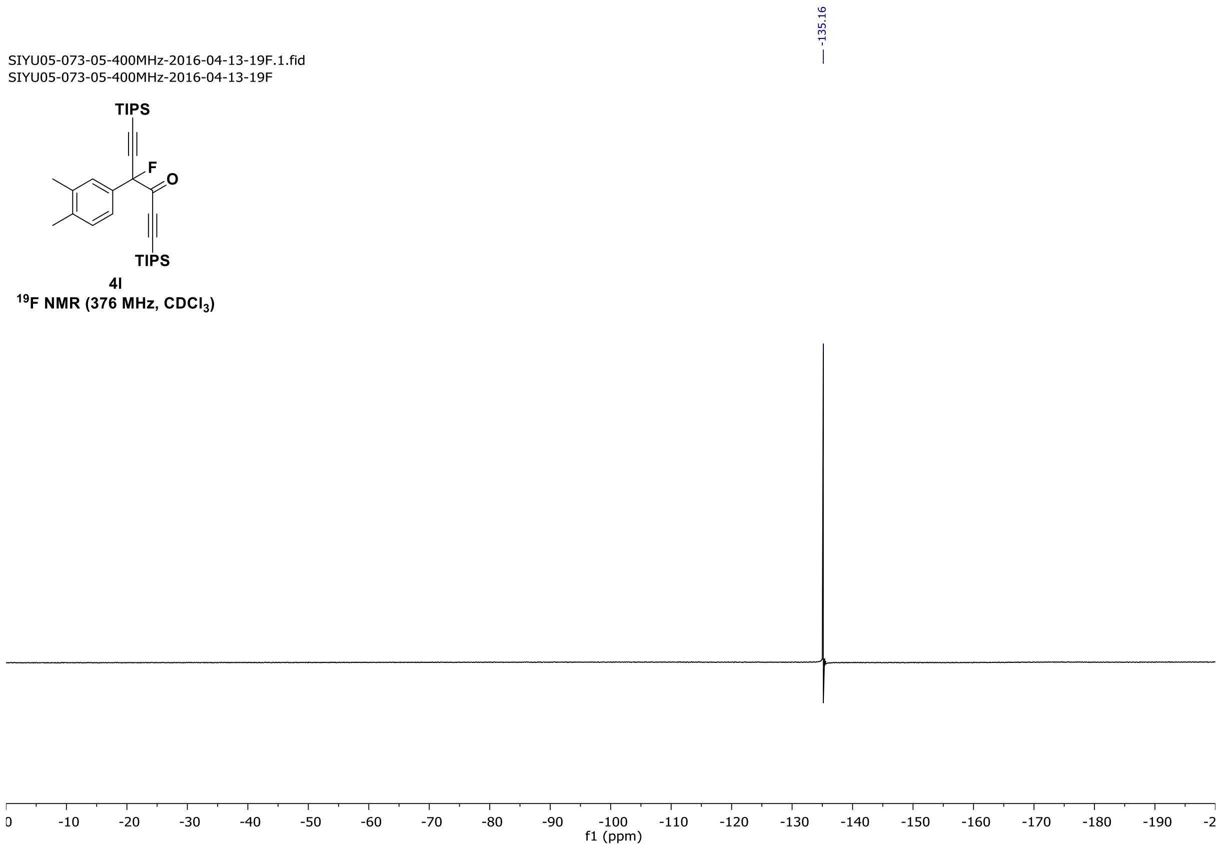

Supplementary Figure 99. ^{19}F NMR of the **4l** (376 MHz, $CDCl_3$)

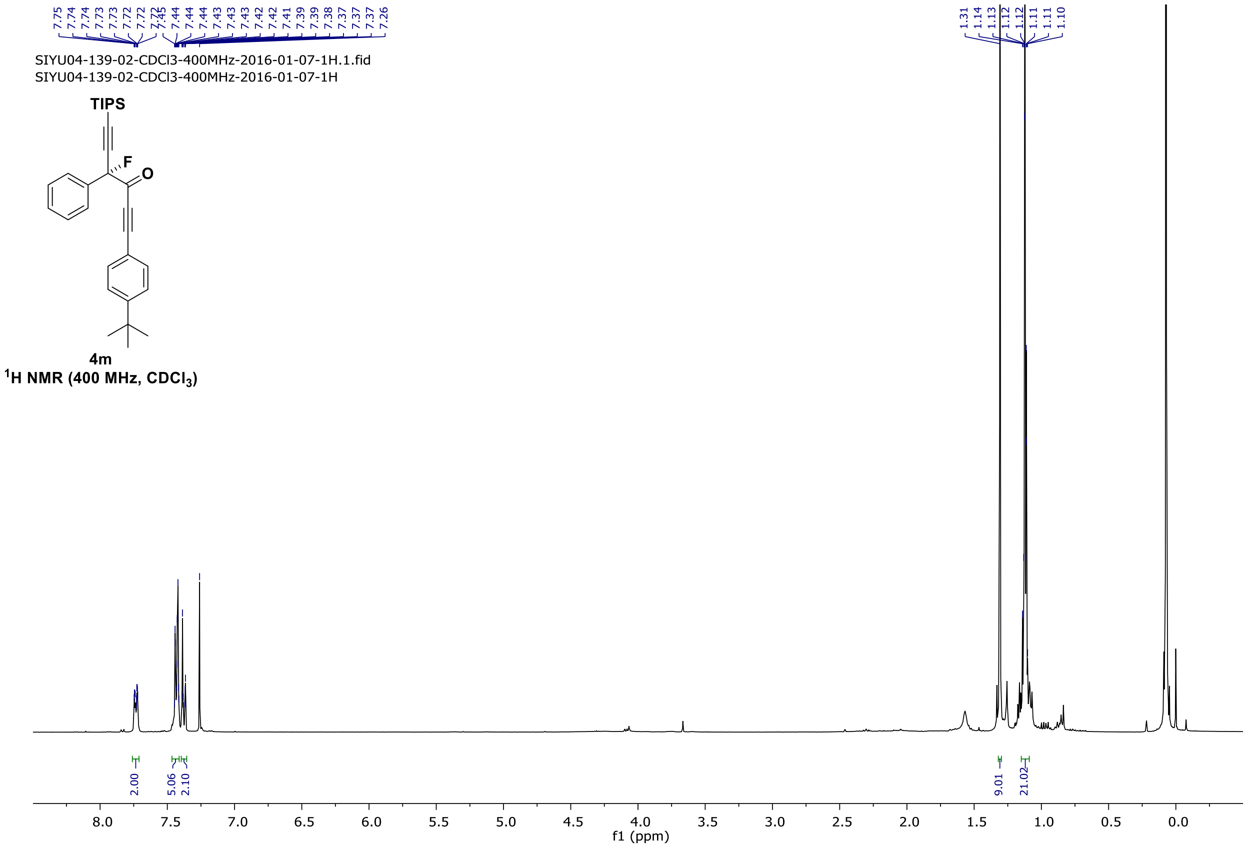

Supplementary Figure 100. ^{1}H NMR of the **4m** (400 MHz, $CDCl_3$)

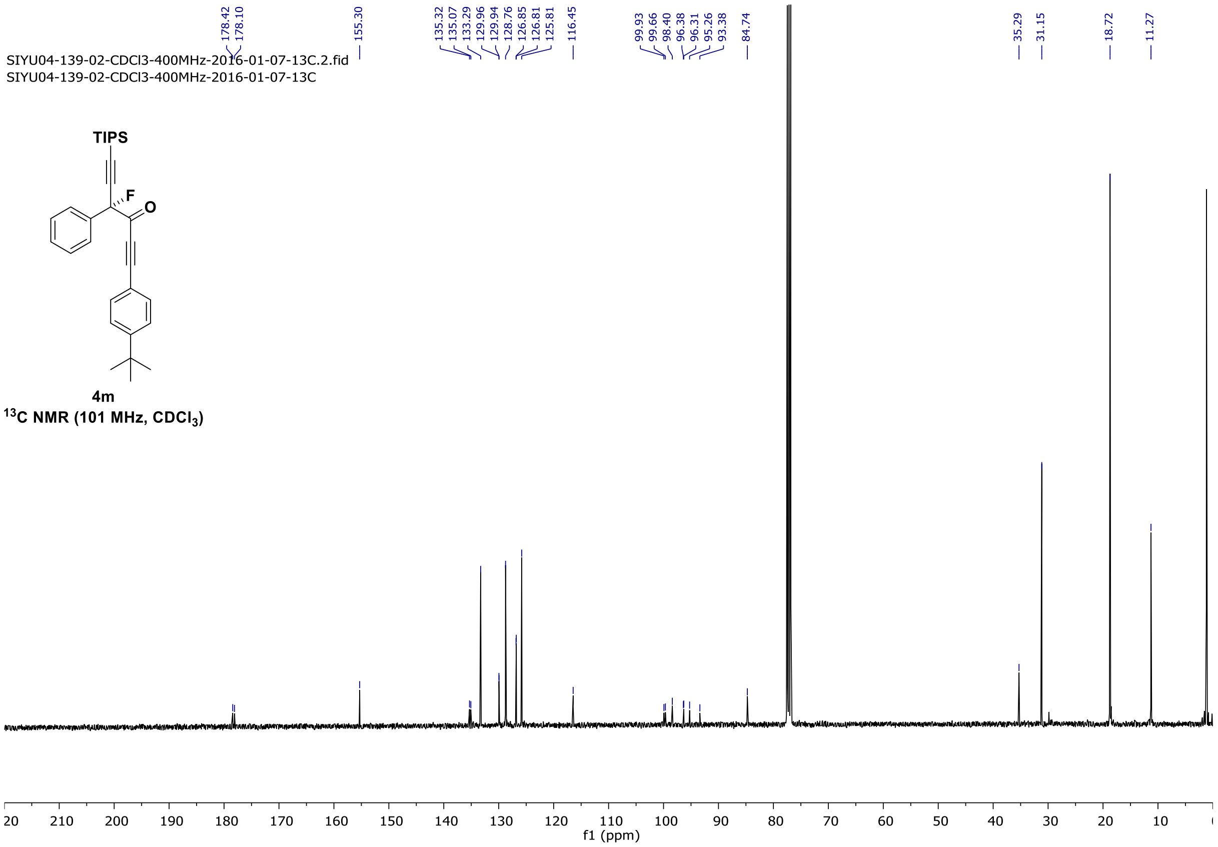

Supplementary Figure 101. ^{13}C NMR of the **4m** (101 MHz, $CDCl_3$)

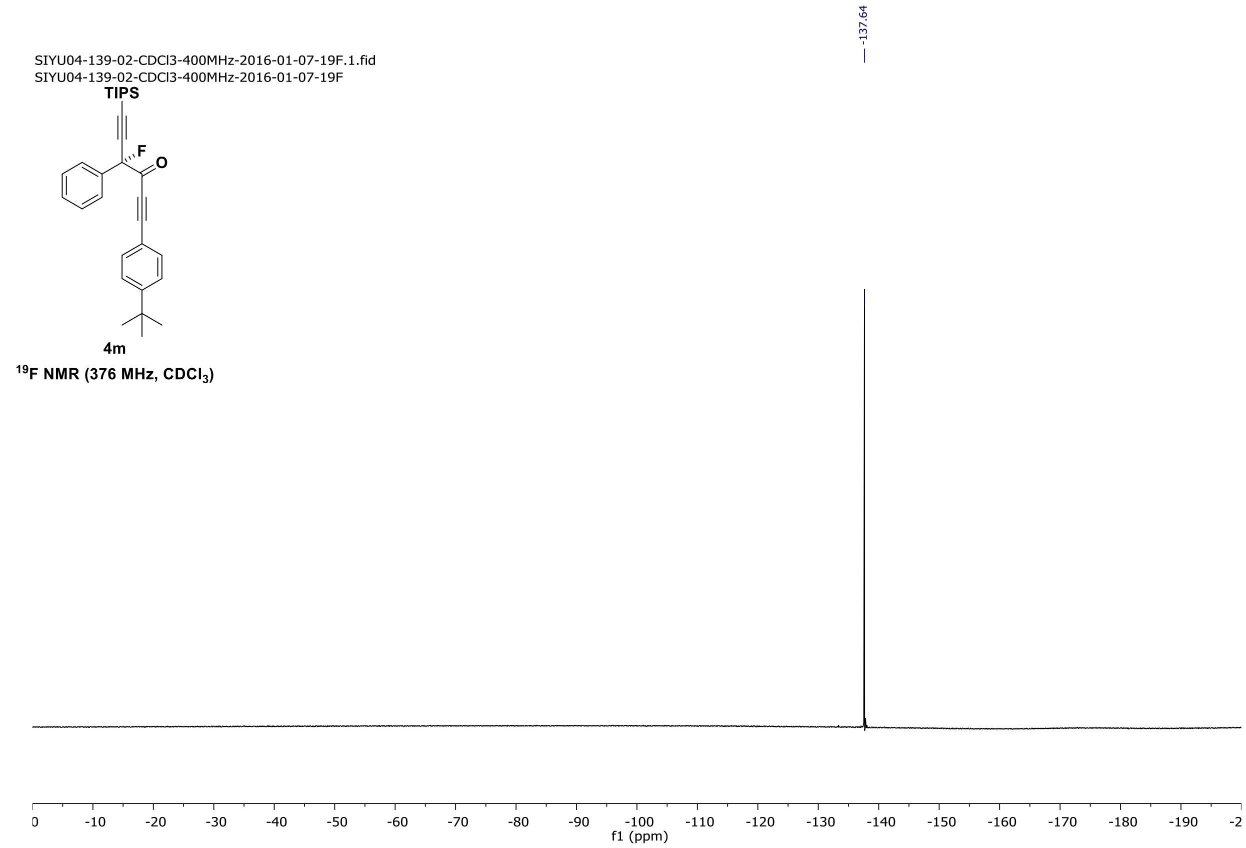

Supplementary Figure 102. ^{19}F NMR of the **4m** (376 MHz, $CDCl_3$)

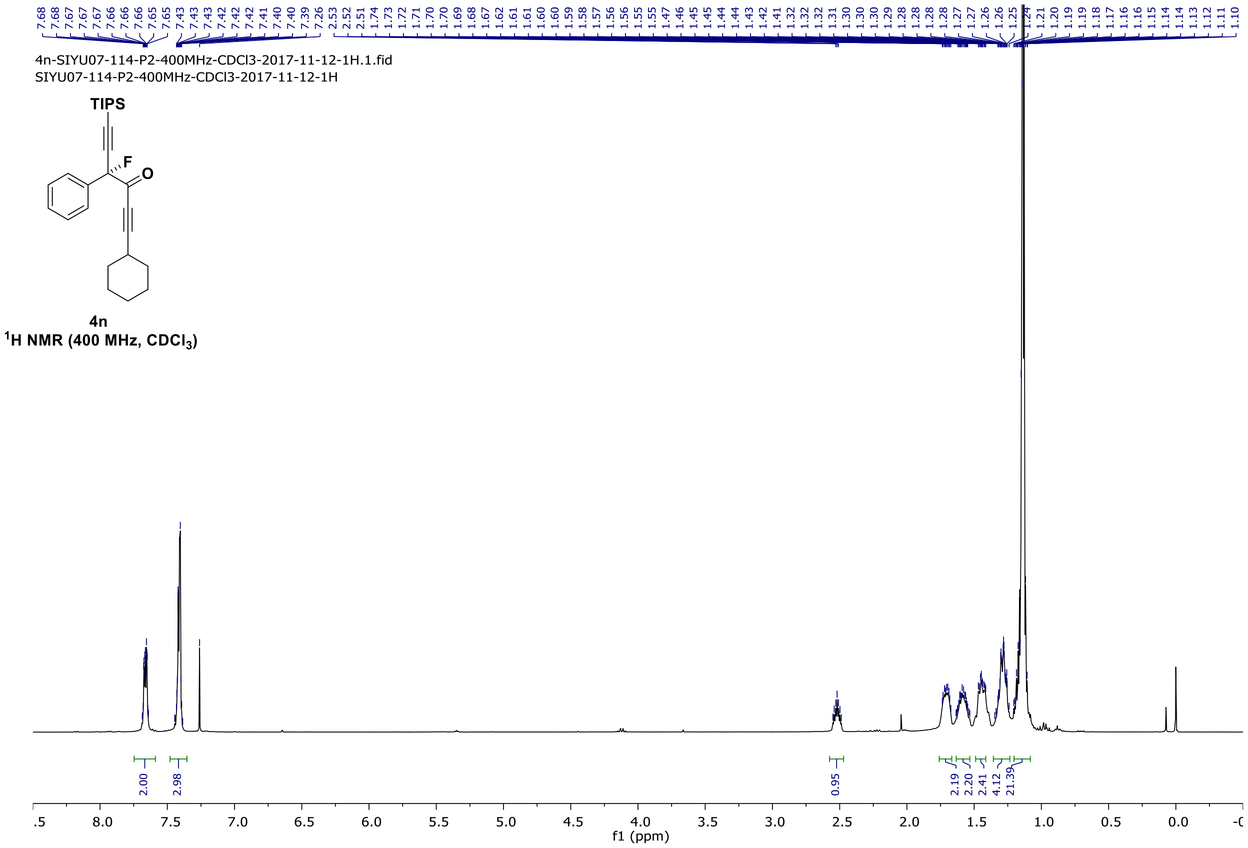

Supplementary Figure 103. ^{1}H NMR of the **4n** (400 MHz, $CDCl_3$)

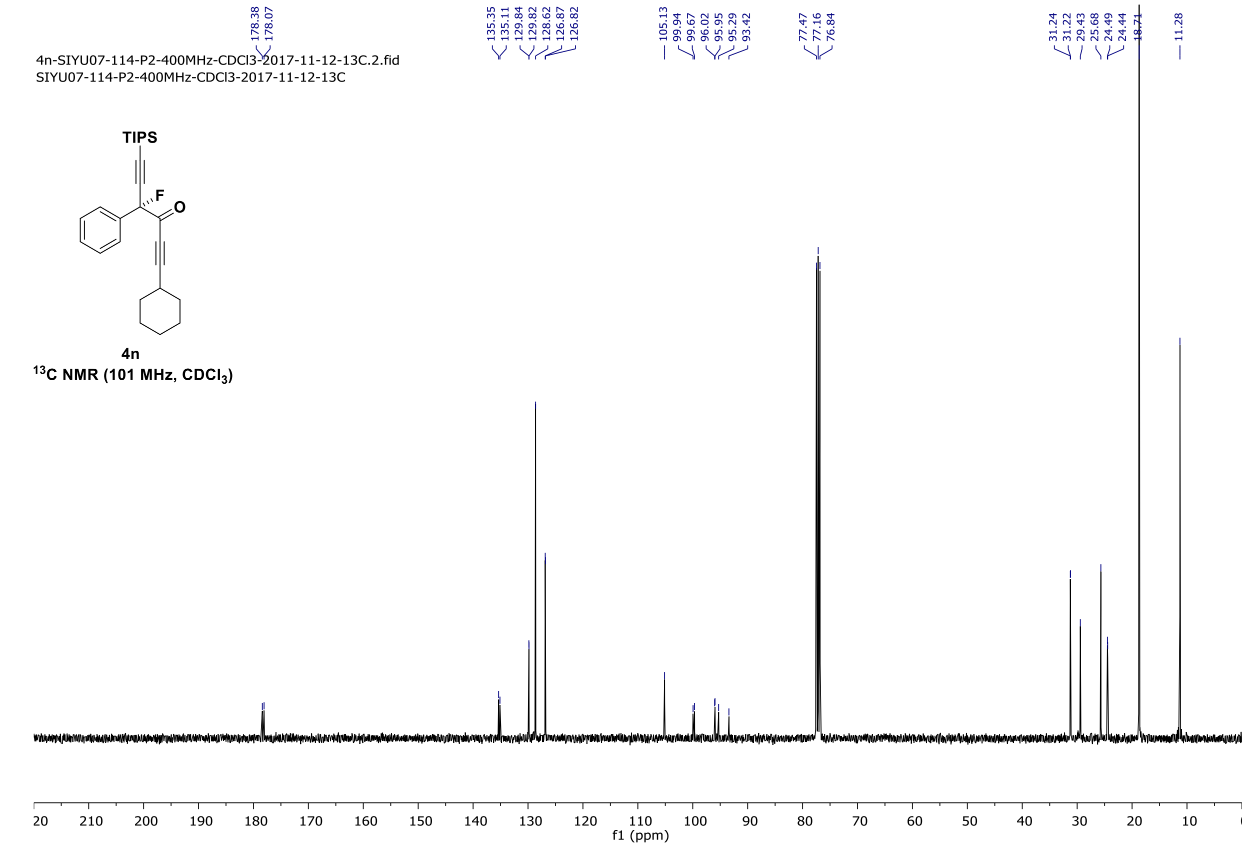

Supplementary Figure 104. ^{13}C NMR of the **4n** (101 MHz, $CDCl_3$)

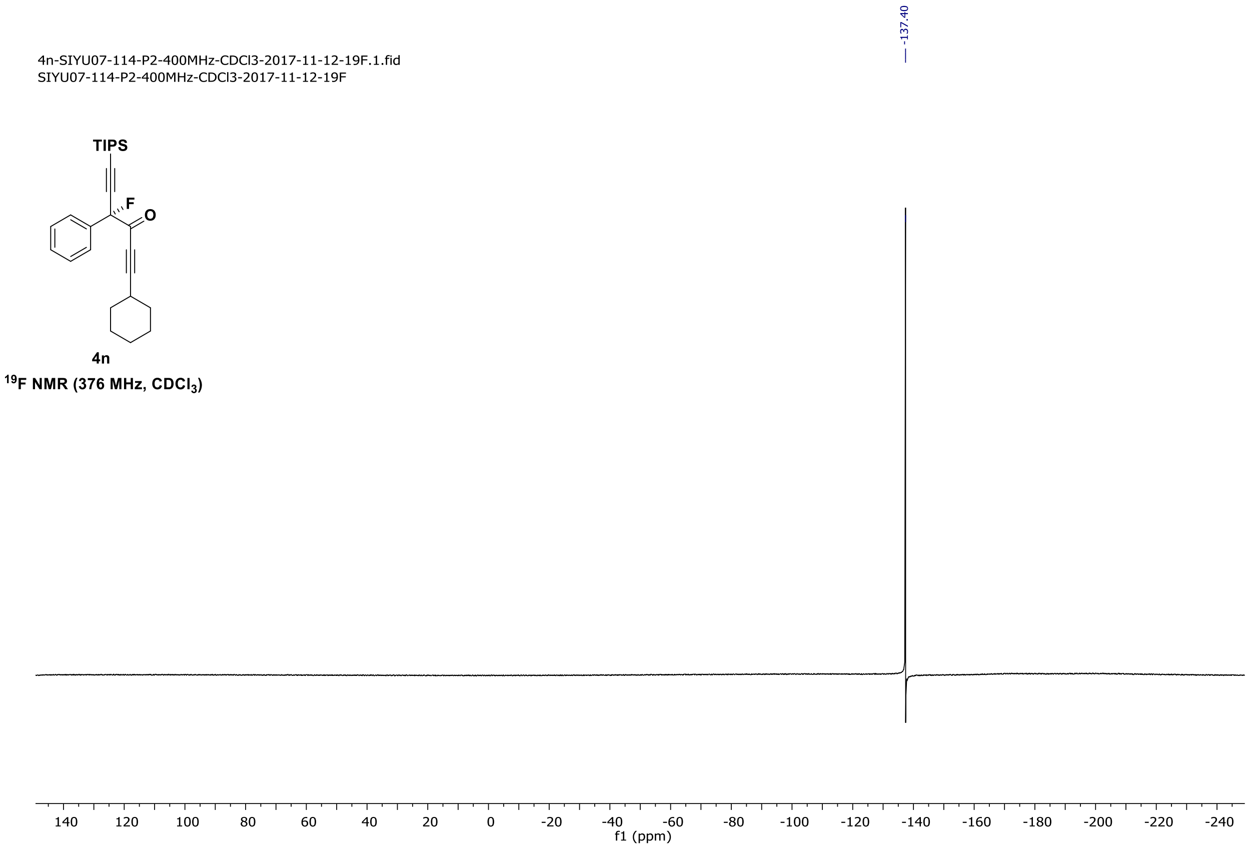

Supplementary Figure 105. ^{19}F NMR of the **4n** (376 MHz, $CDCl_3$)

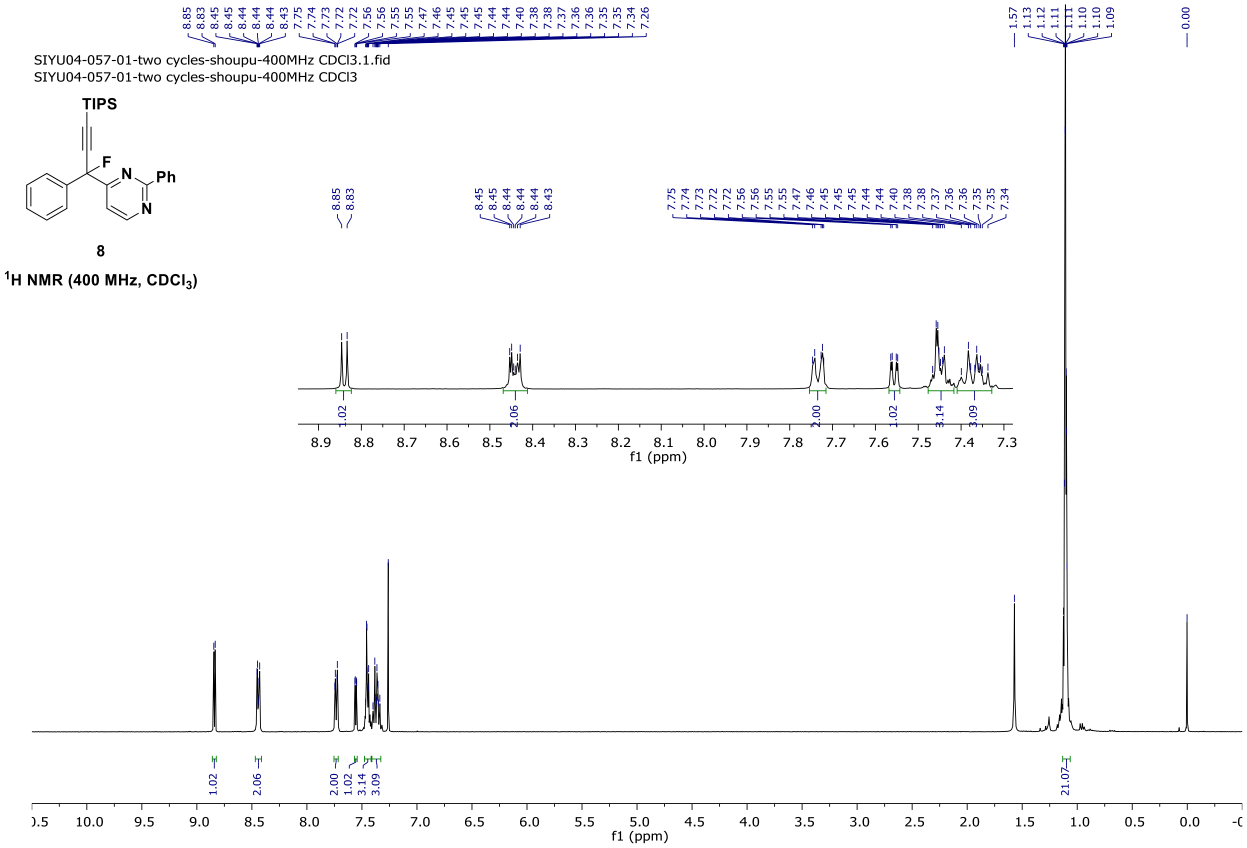

Supplementary Figure 106. ^{1}H NMR of the compound **8** (400 MHz, $CDCl_3$)

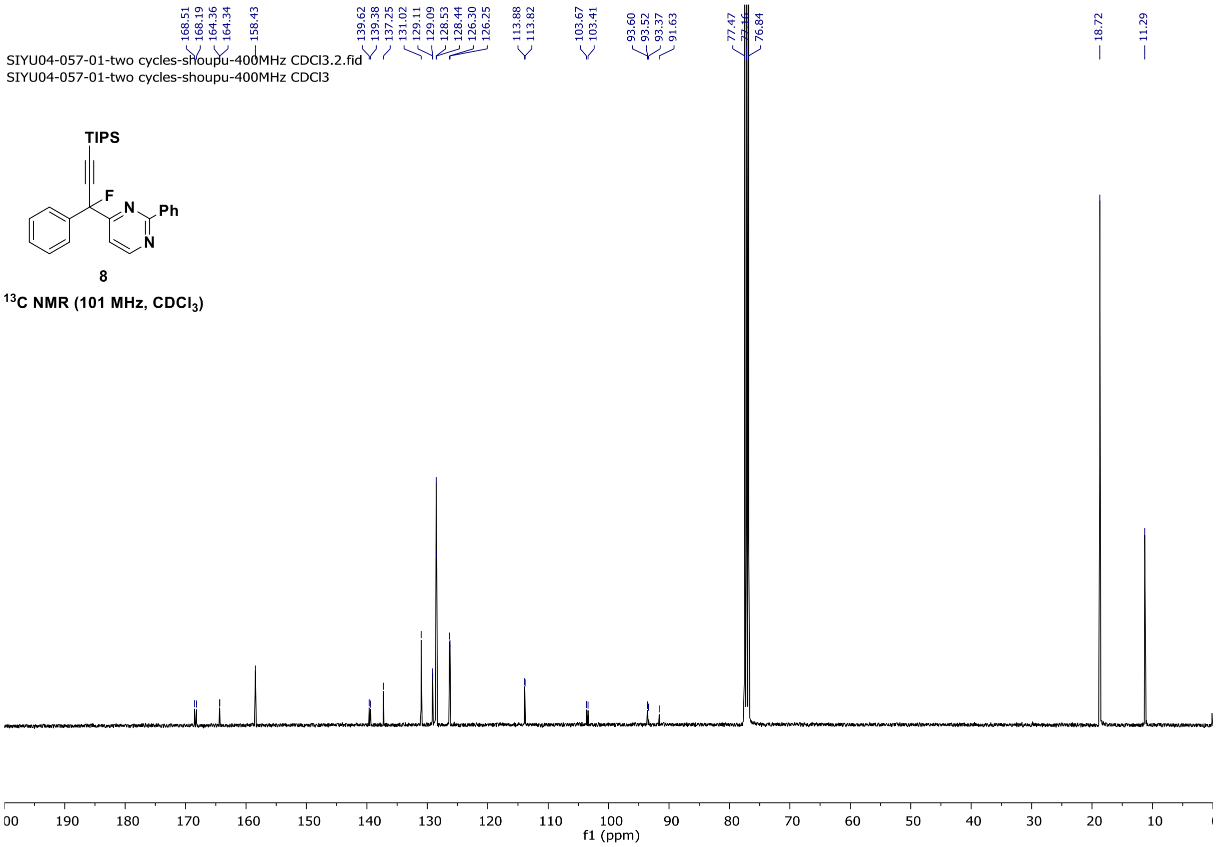

Supplementary Figure 107. ^{13}C NMR of the compound **8** (101 MHz, $CDCl_3$)

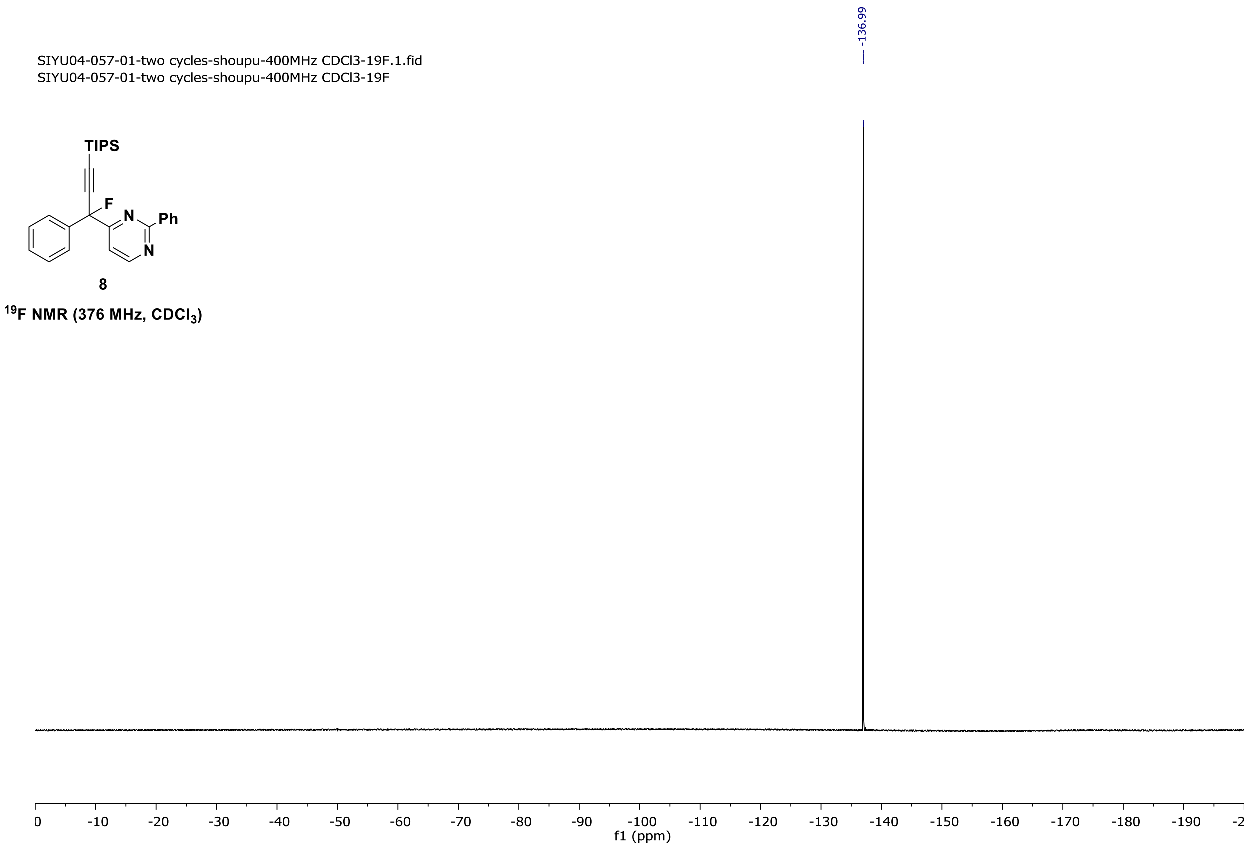

Supplementary Figure 108. ^{19}F NMR of the compound **8** (376 MHz, $CDCl_3$)

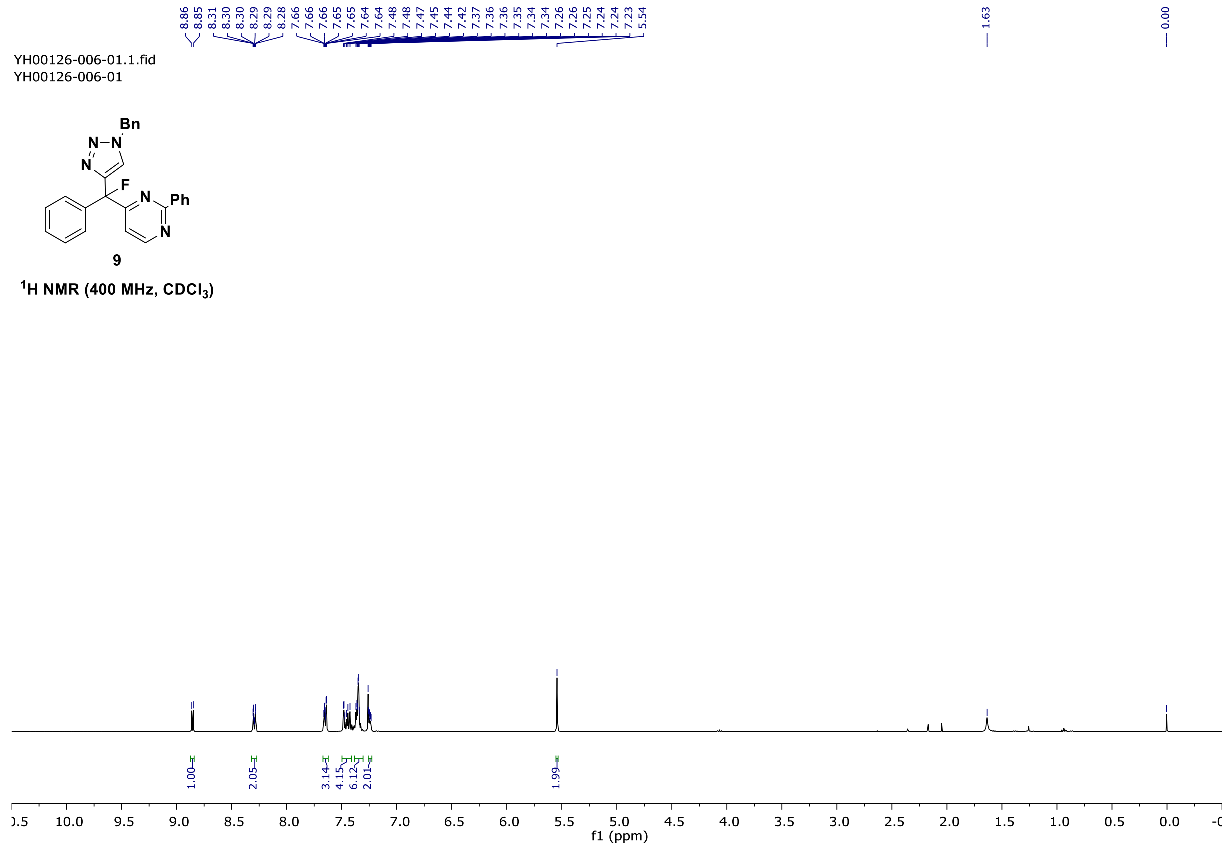

Supplementary Figure 109. ^{1}H NMR of the compound **9** (400 MHz, $CDCl_3$)

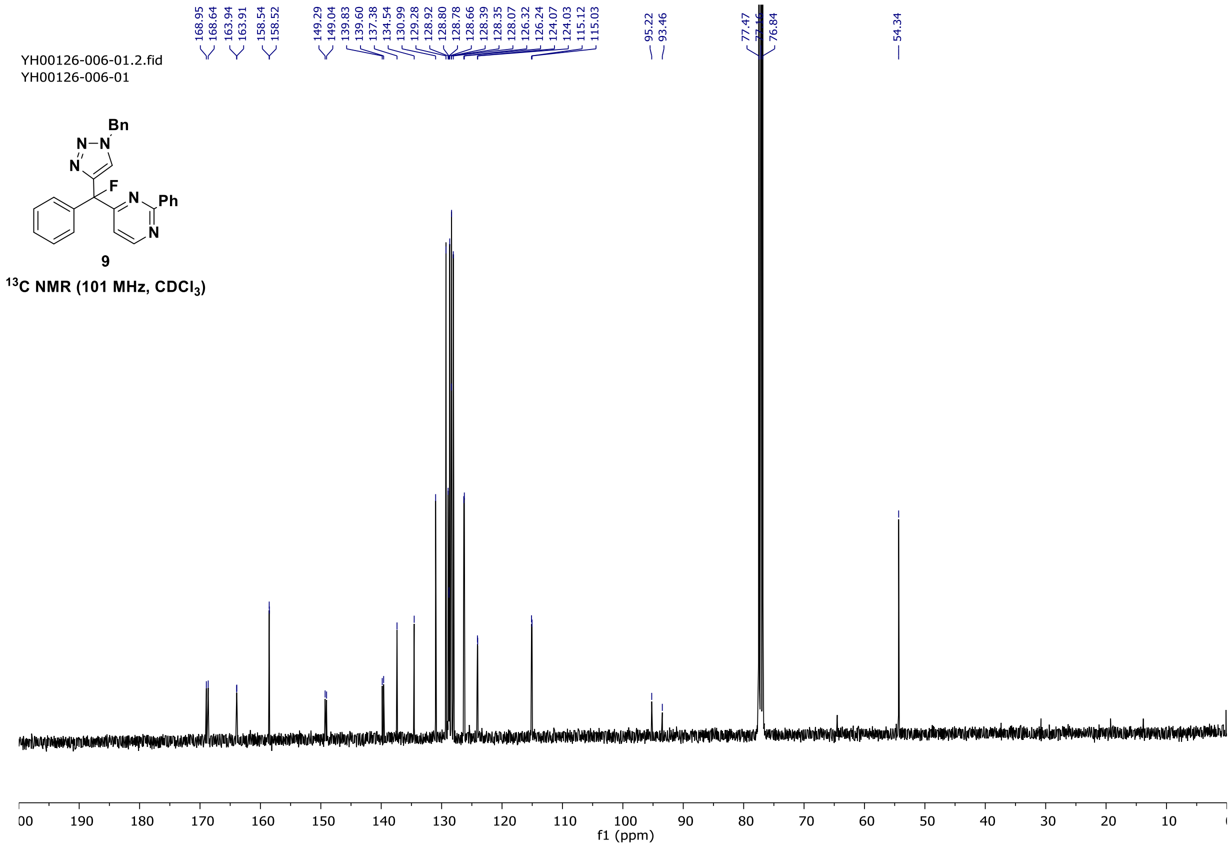

Supplementary Figure 110. ^{13}C NMR of the compound **9** (101 MHz, $CDCl_3$)

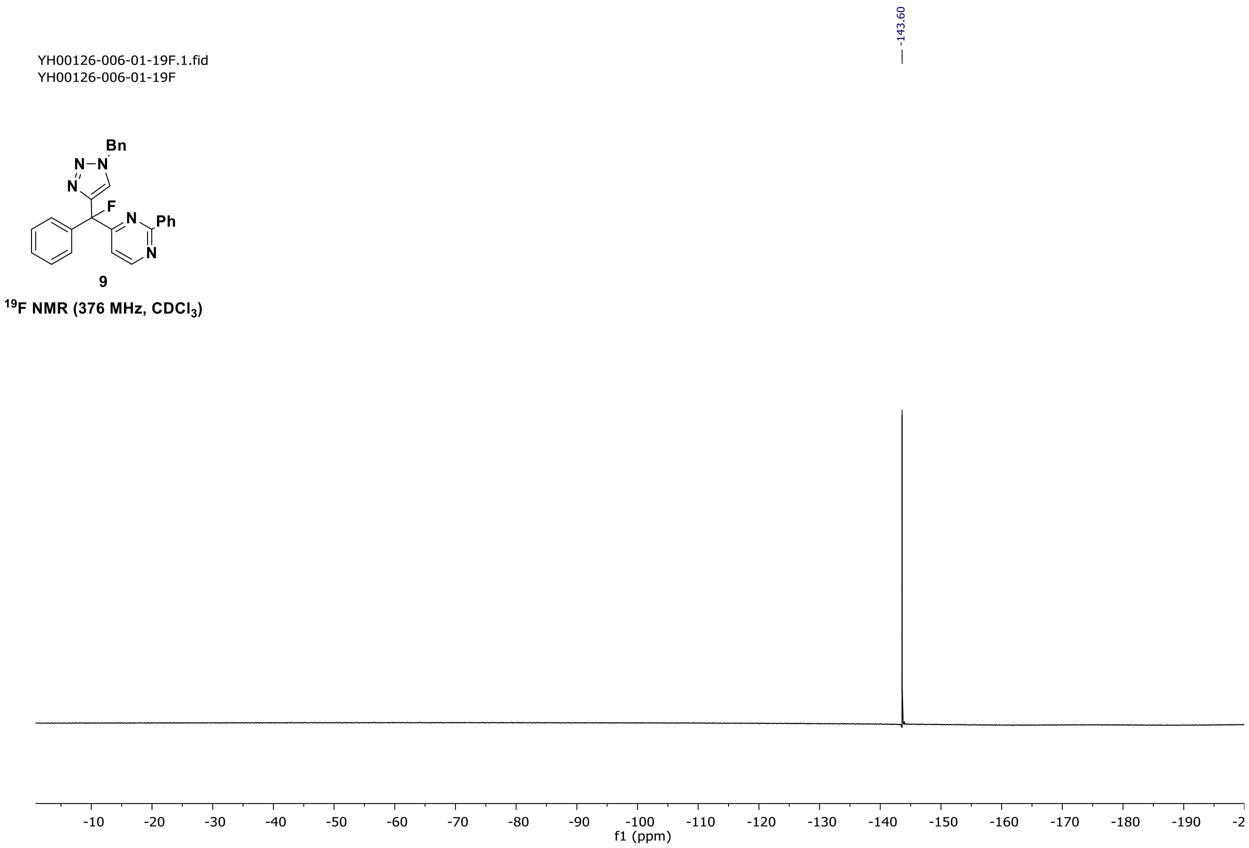

Supplementary Figure 111. ^{19}F NMR of the compound **9** (376 MHz, $CDCl_3$)

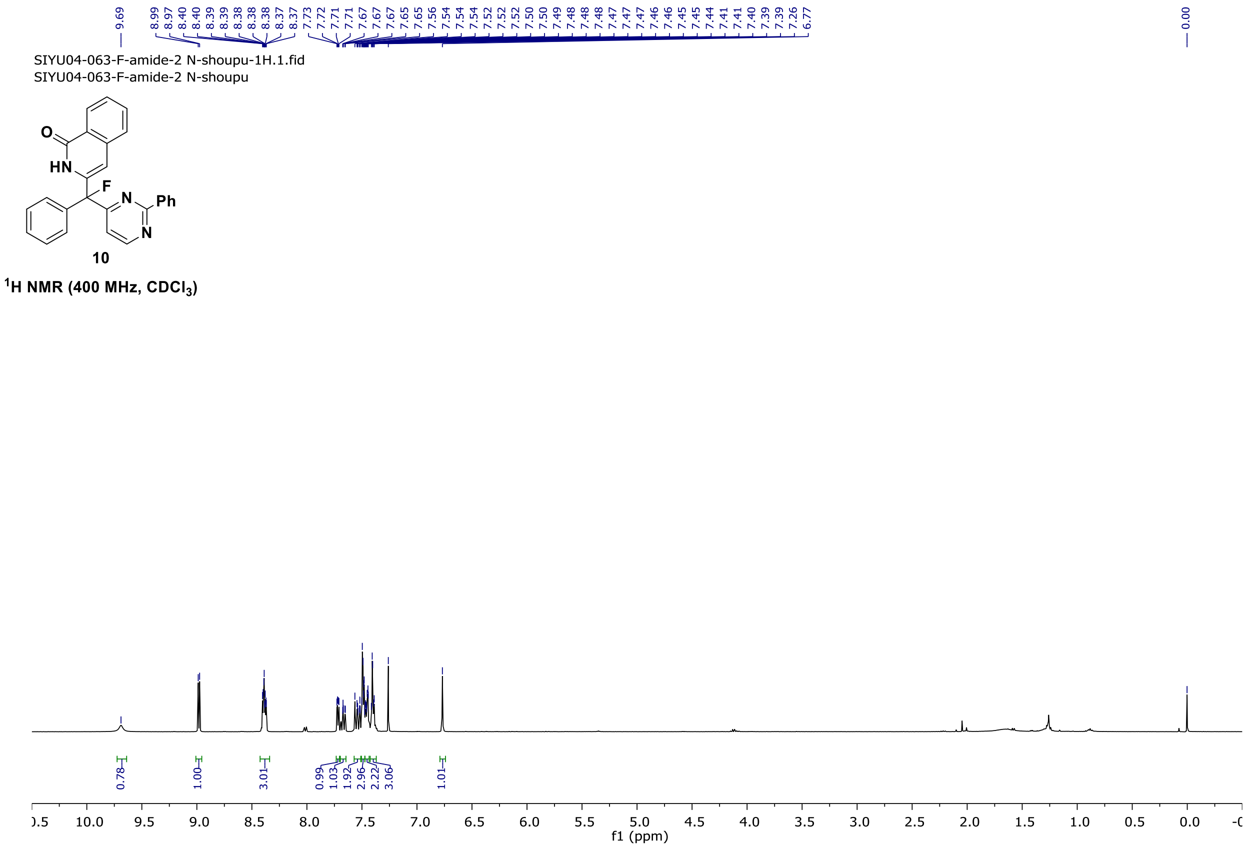

Supplementary Figure 112. ^{1}H NMR of the compound **10** (400 MHz, $CDCl_3$)

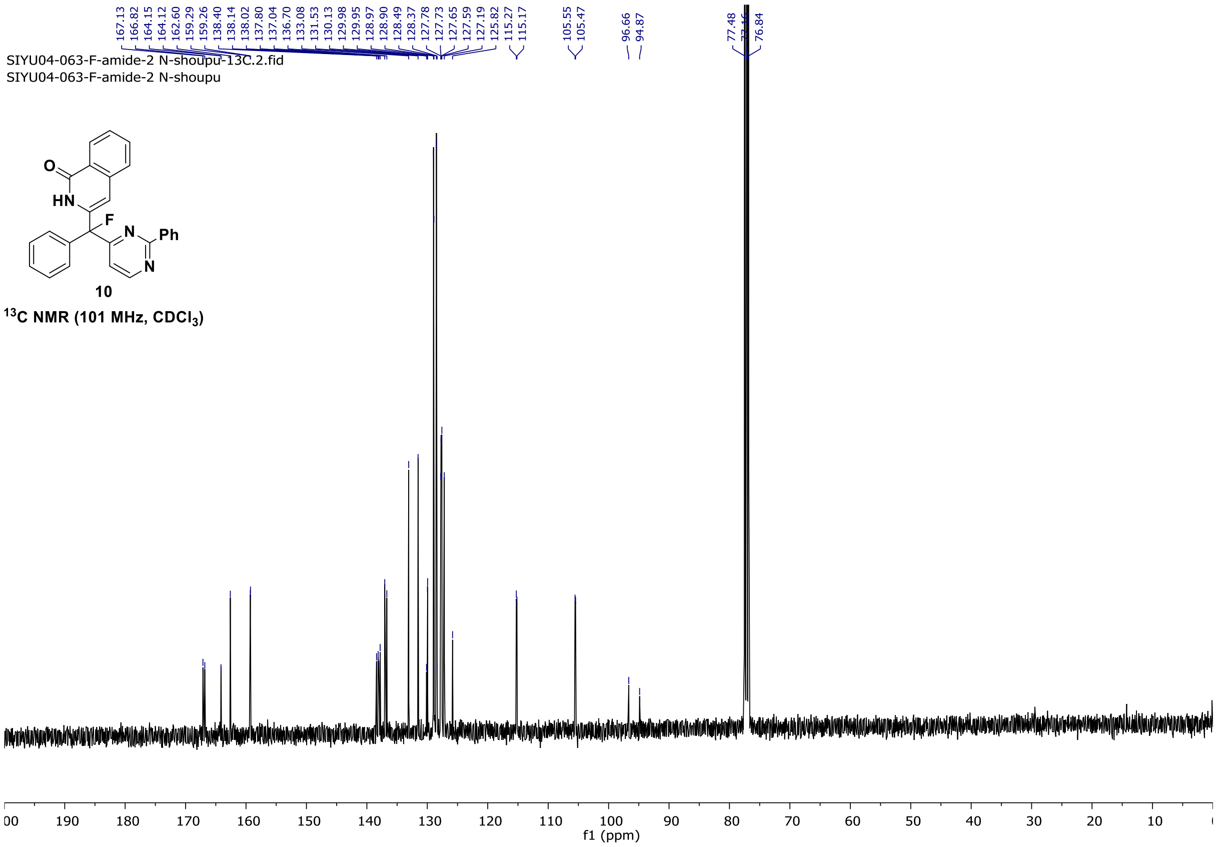

Supplementary Figure 113. ^{13}C NMR of the compound **10** (101 MHz, $CDCl_3$)

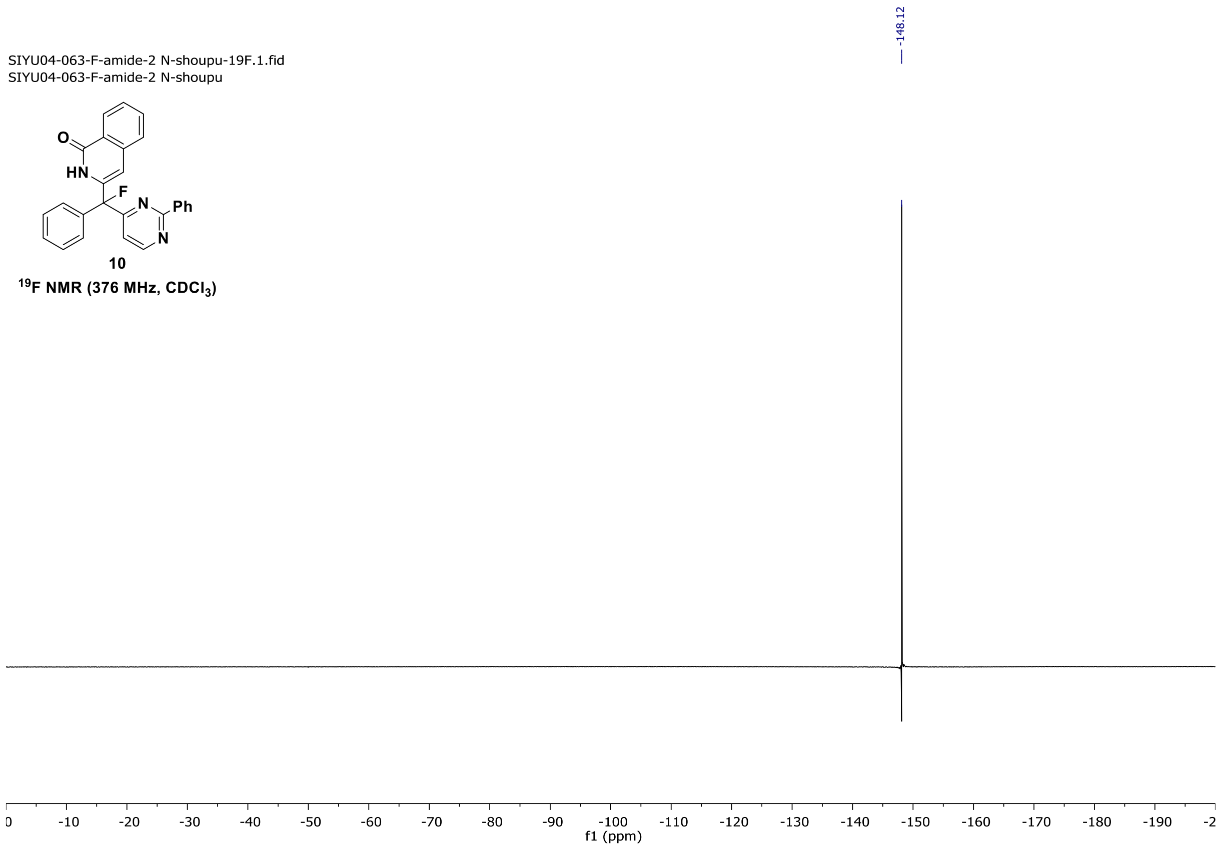

Supplementary Figure 114. ^{19}F NMR of the compound **10** (376 MHz, $CDCl_3$)

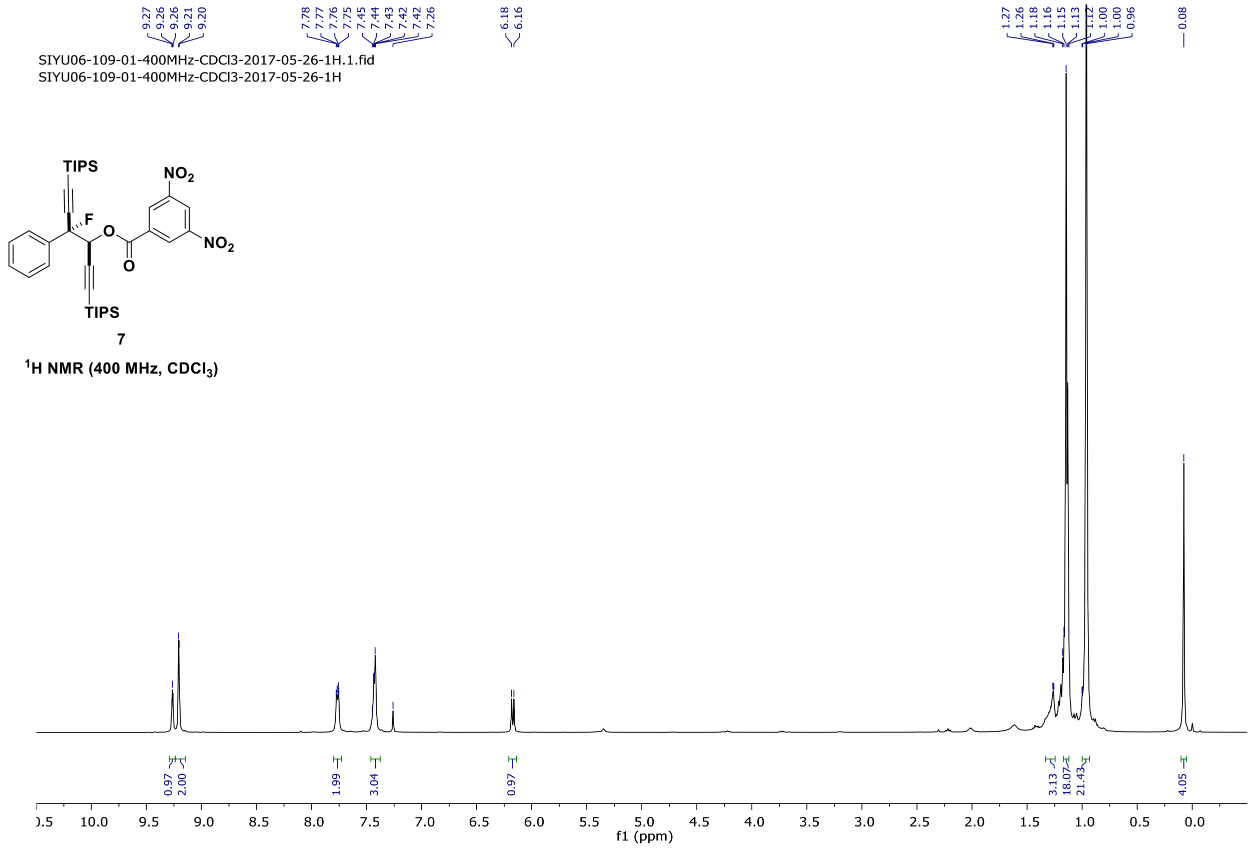

Supplementary Figure 115. ^{1}H NMR of the compound **7** (400 MHz, $CDCl_3$)

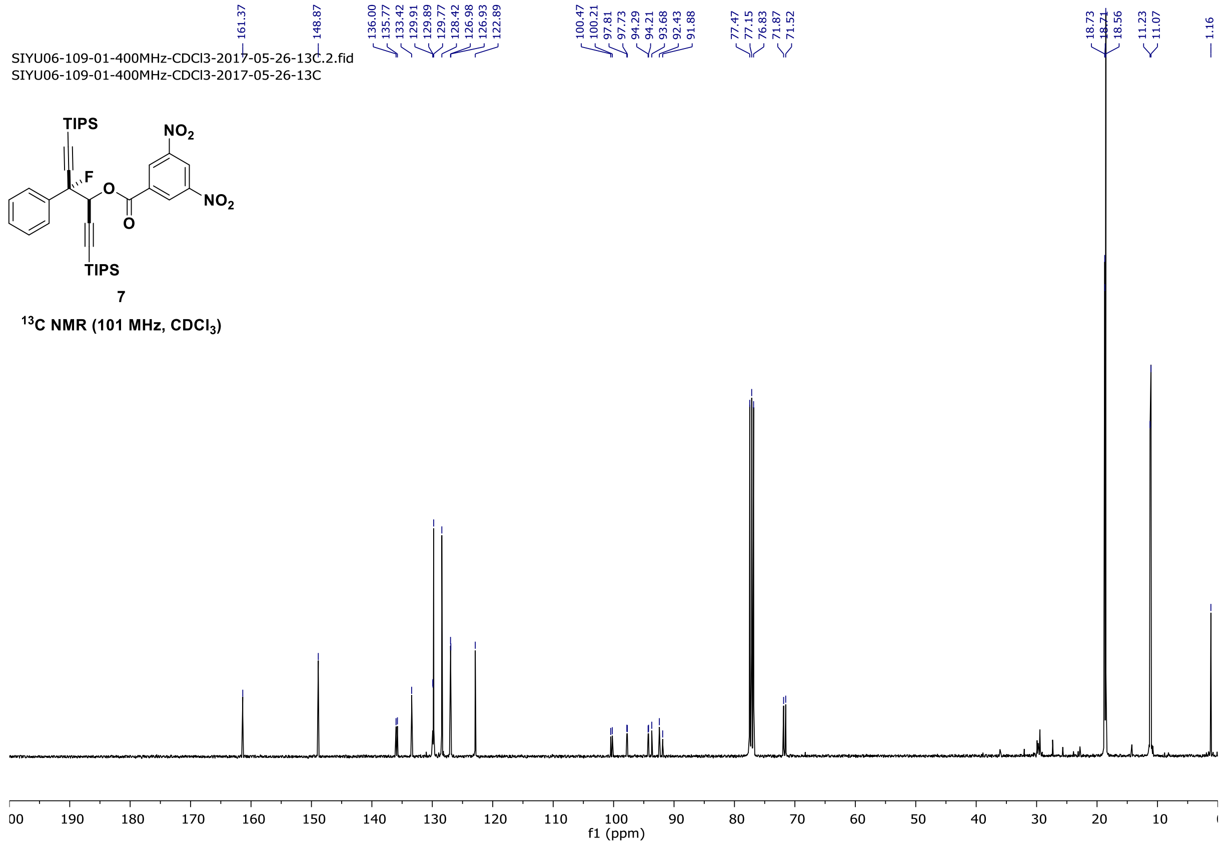

Supplementary Figure 116. ^{13}C NMR of the compound **7** (101 MHz, $CDCl_3$)

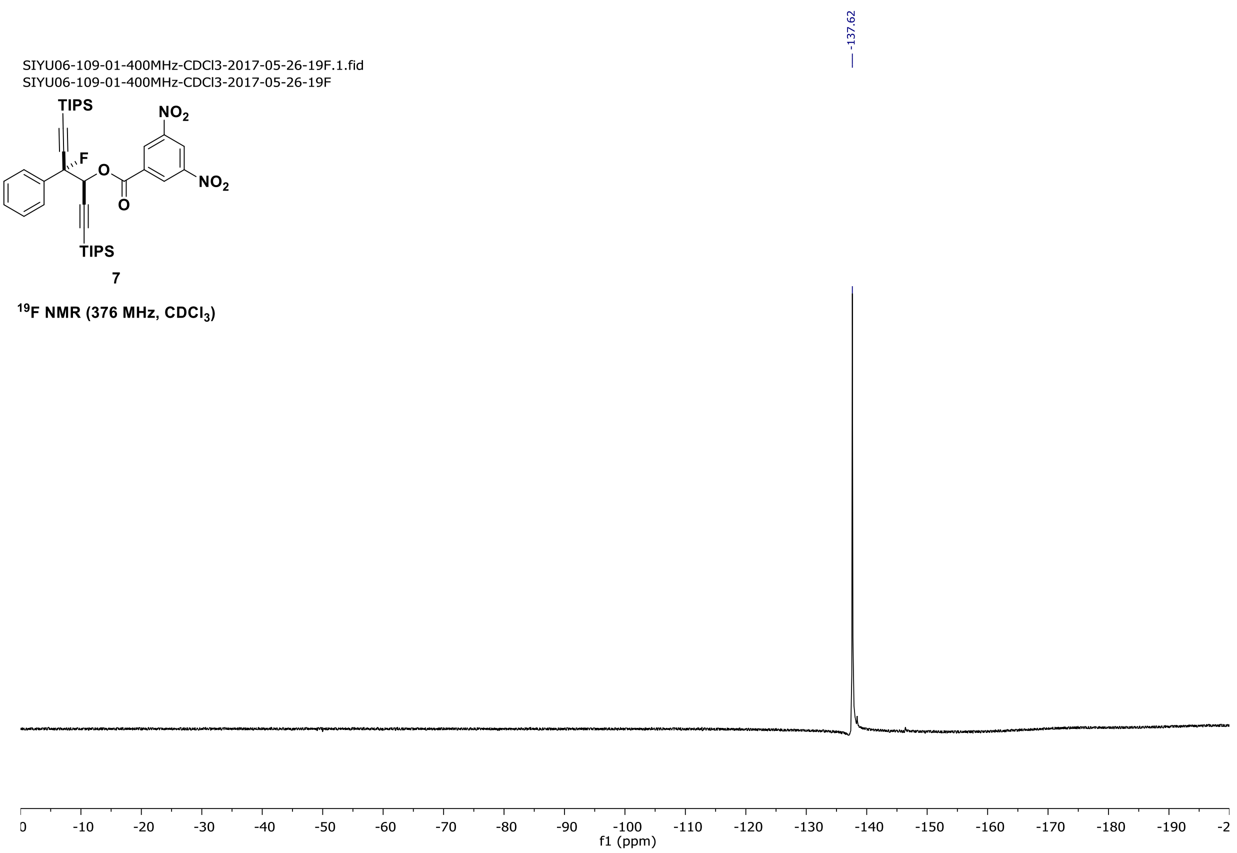

Supplementary Figure 117. ^{19}F NMR of the compound **7** (376 MHz, $CDCl_3$)

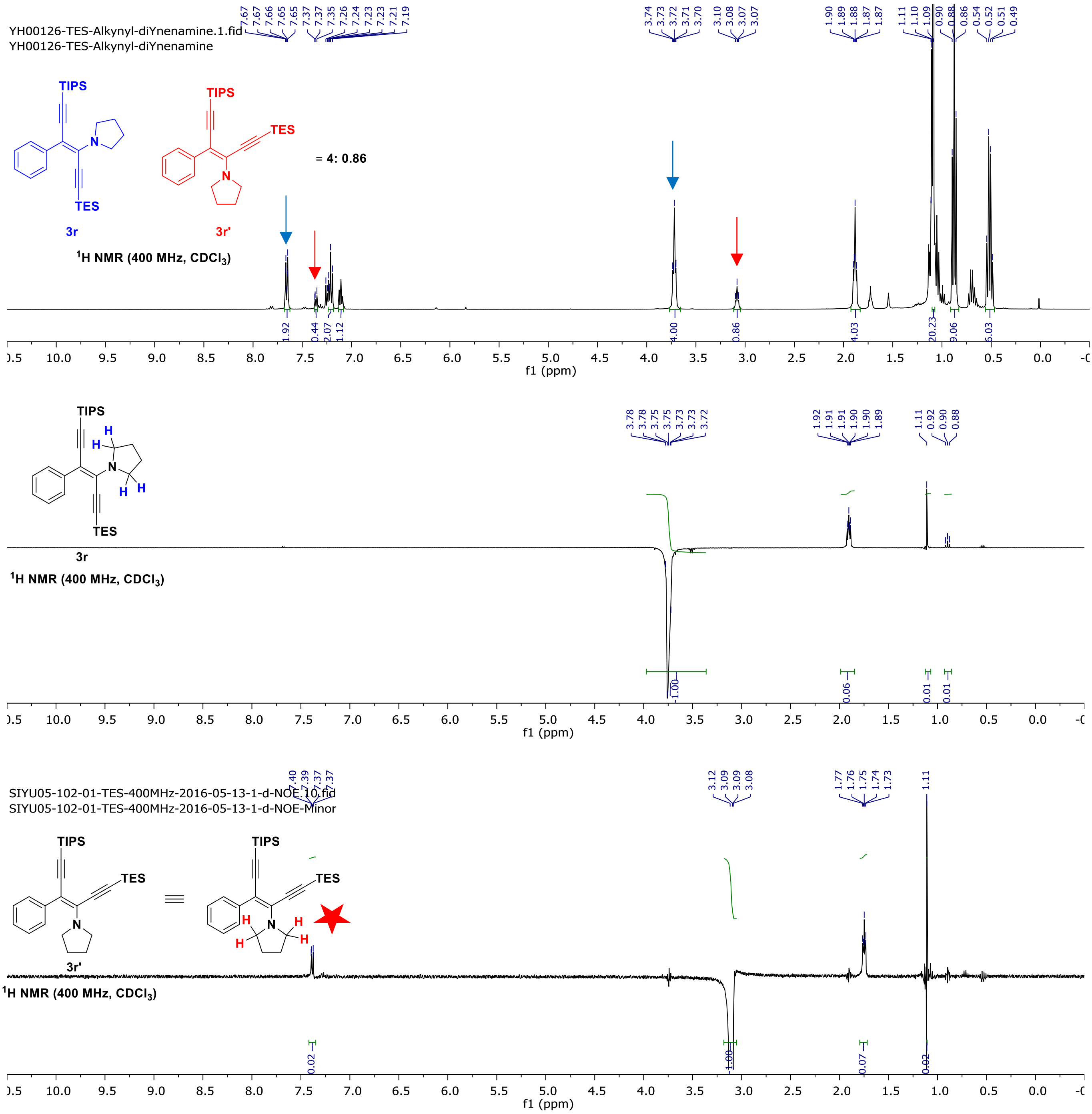

Supplementary Figure 118. NOE (400 MHz, $CDCl_3$): trans-enamine **3r** is the major product

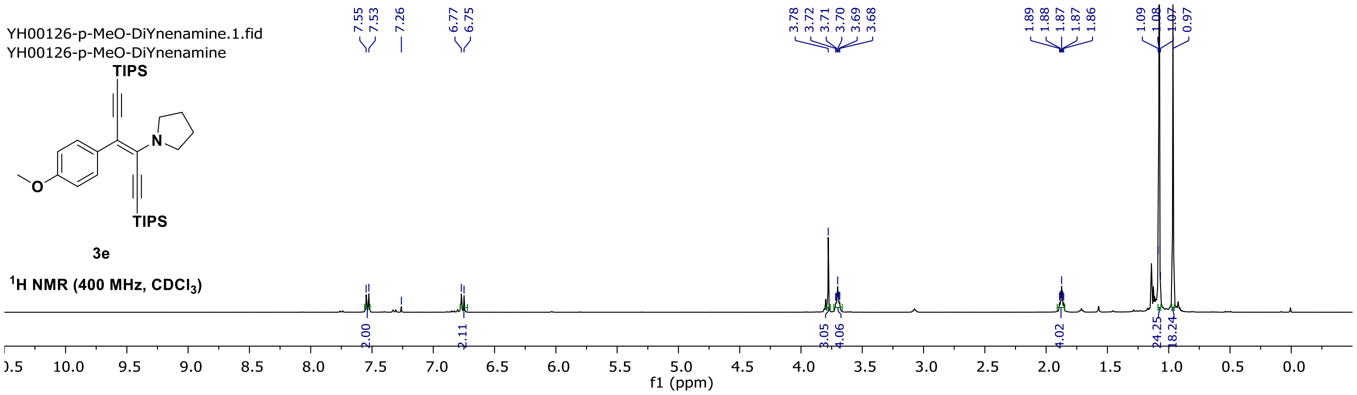

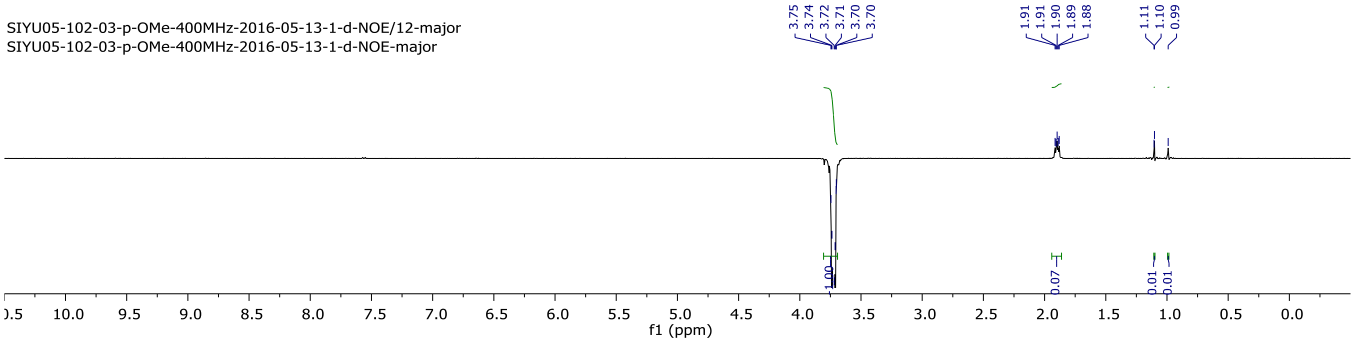

Supplementary Figure 119. NOE (400 MHz, $CDCl_3$): trans-enamine **3e** is the major product

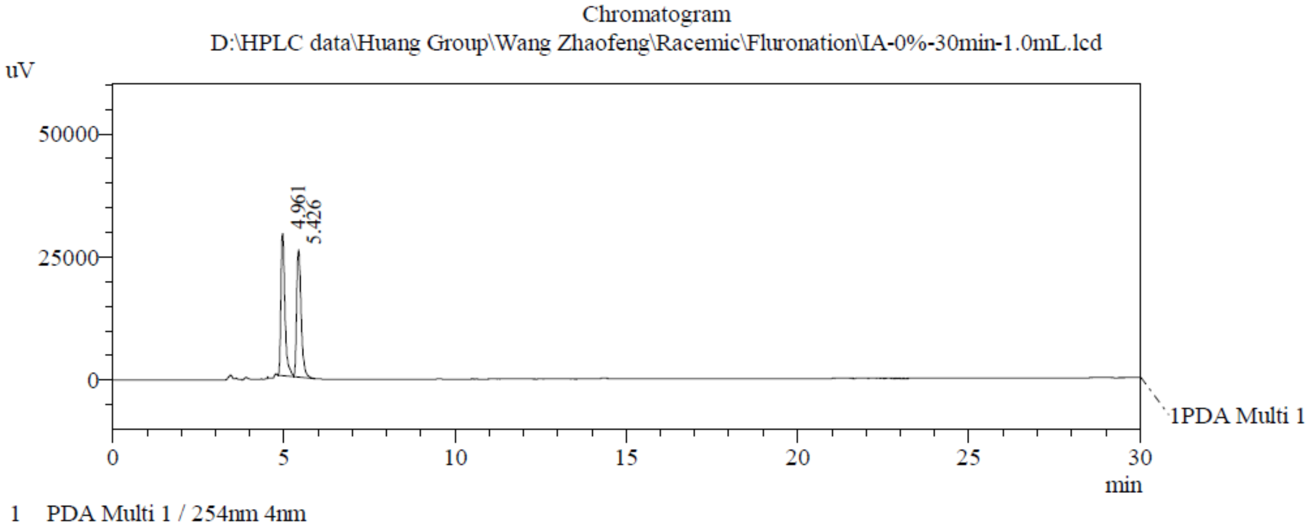

PeakTable

PDA Ch1 254nm 4nm

Peak#	Ret. Time	Area	Height	Area %	Height %
1	4.961	233073	28791	50.011	52.689
2	5.426	232967	25852	49.989	47.311
Total		466040	54643	100.000	100.000

Supplementary Figure 120. HPLC traces for racemic product **4a**

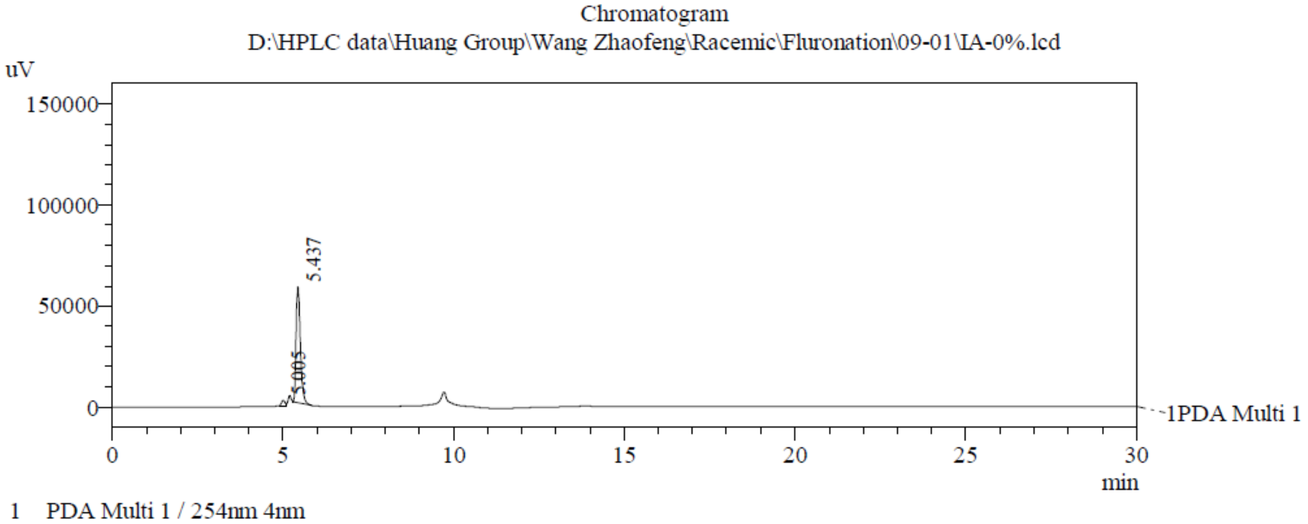

PeakTable

PDA Ch1 254nm 4nm

Peak#	Ret. Time	Area	Height	Area %	Height %
1	5.005	19912	2865	3.840	4.750
2	5.437	498633	57446	96.160	95.250
Total		518545	60311	100.000	100.000

Supplementary Figure 121. HPLC traces for chiral product **4a**

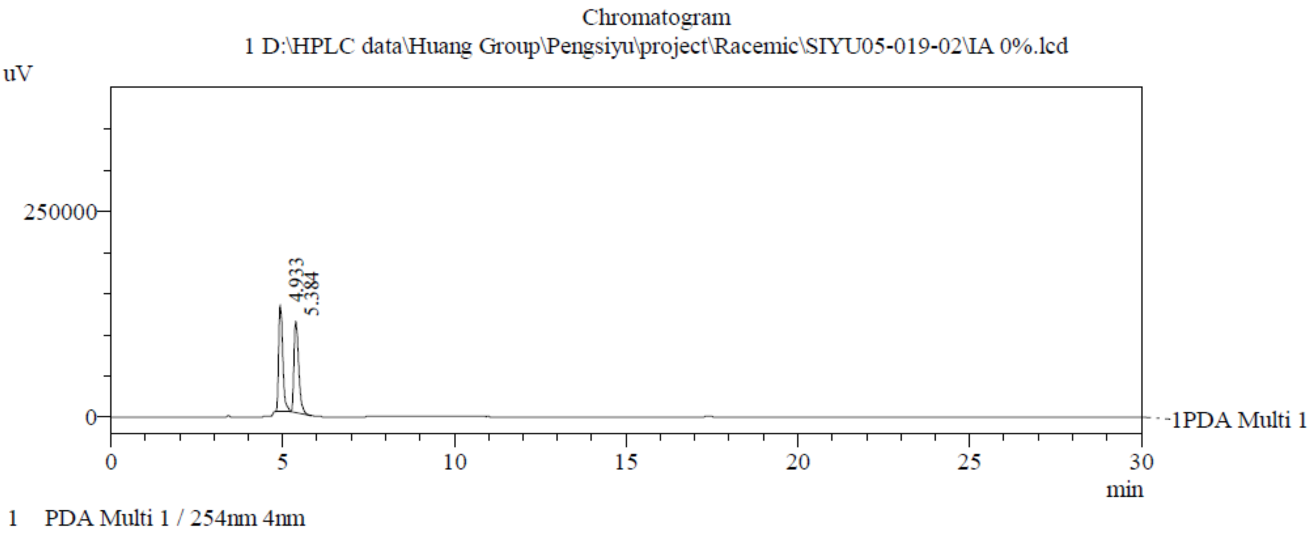

PeakTable

PDA Ch1 254nm 4nm

Peak#	Ret. Time	Area	Height	Area %	Height %
1	4.933	1062177	129227	50.204	53.879
2	5.384	1053564	110619	49.796	46.121
Total		2115741	239846	100.000	100.000

Supplementary Figure 122. HPLC traces for racemic product **4b**

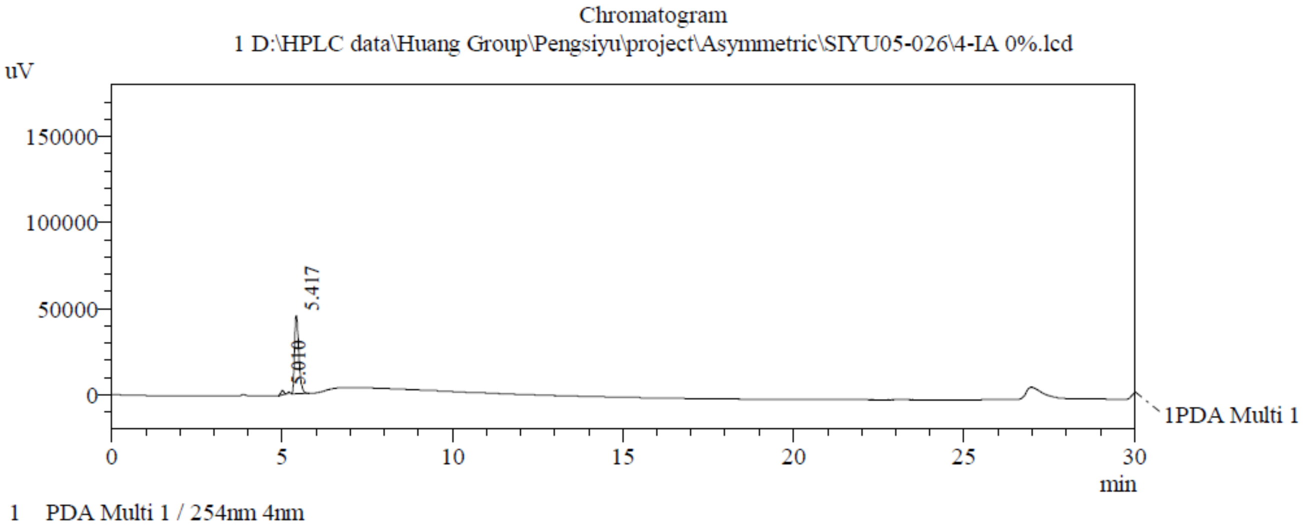

PeakTable

PDA Ch1 254nm 4nm

Peak#	Ret. Time	Area	Height	Area %	Height %
1	5.010	15589	2489	3.830	5.209
2	5.417	391476	45287	96.170	94.791
Total		407065	47775	100.000	100.000

Supplementary Figure 123. HPLC traces for chiral product **4b**

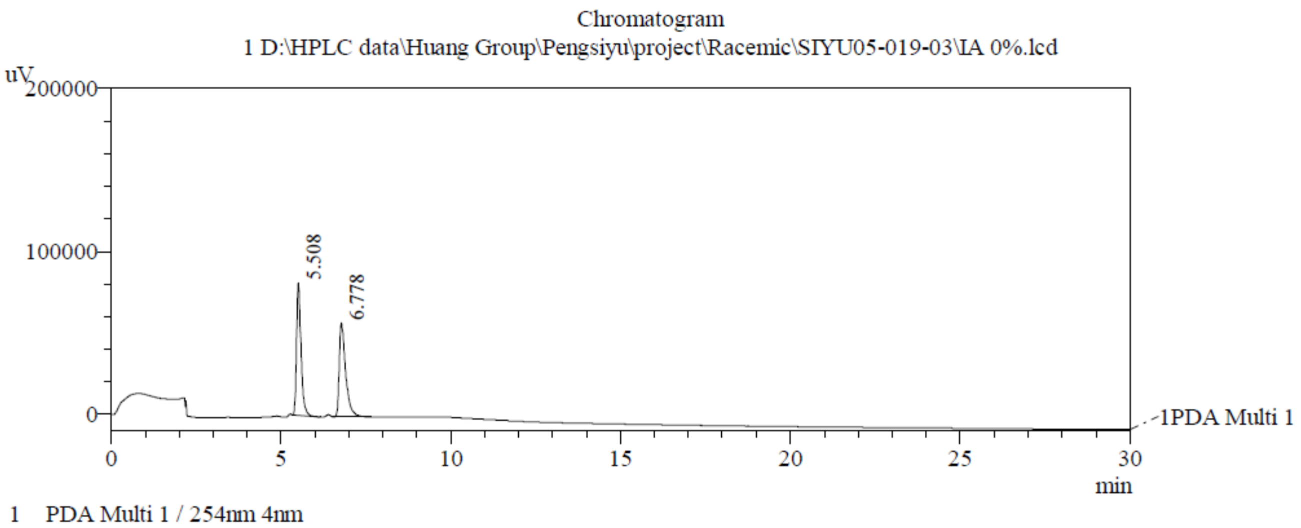

PeakTable

PDA Ch1 254nm 4nm

Peak#	Ret. Time	Area	Height	Area %	Height %
1	5.508	759856	81595	49.753	58.617
2	6.778	767399	57606	50.247	41.383
Total		1527255	139201	100.000	100.000

Supplementary Figure 124. HPLC traces for racemic product **4c**

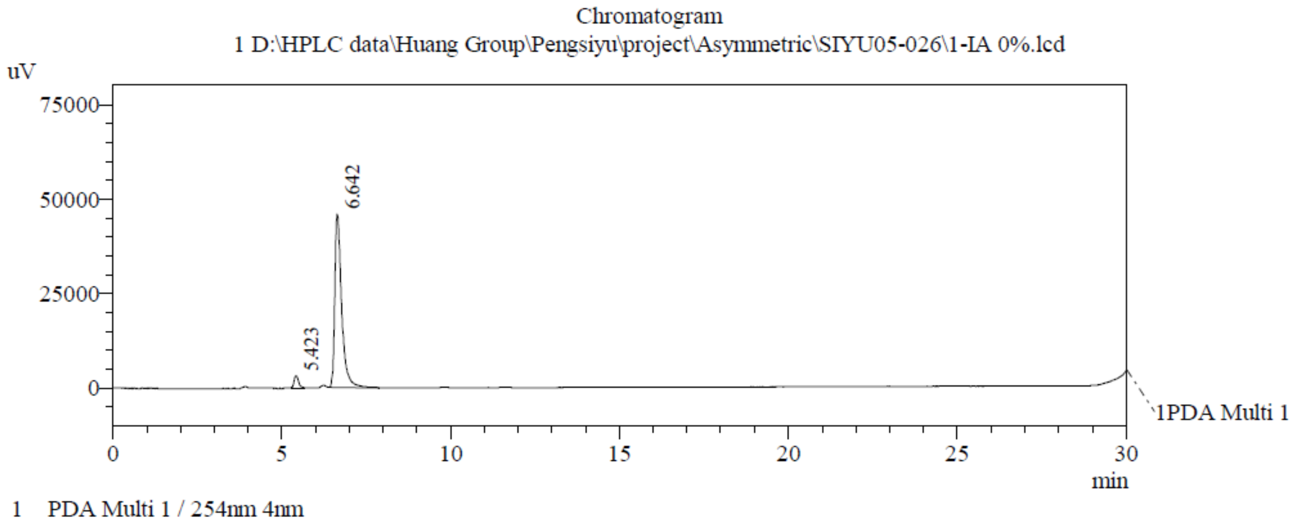

PeakTable

PDA Ch1 254nm 4nm

Peak#	Ret. Time	Area	Height	Area %	Height %
1	5.423	30401	3298	4.454	6.710
2	6.642	652125	45849	95.546	93.290
Total		682526	49147	100.000	100.000

Supplementary Figure 125. HPLC traces for chiral product **4c**

Chromatogram
1 D:\HPLC data\Huang Group\Pengsiyu\project\Racemic\SIYU05-036-01\IA 0%.lcd

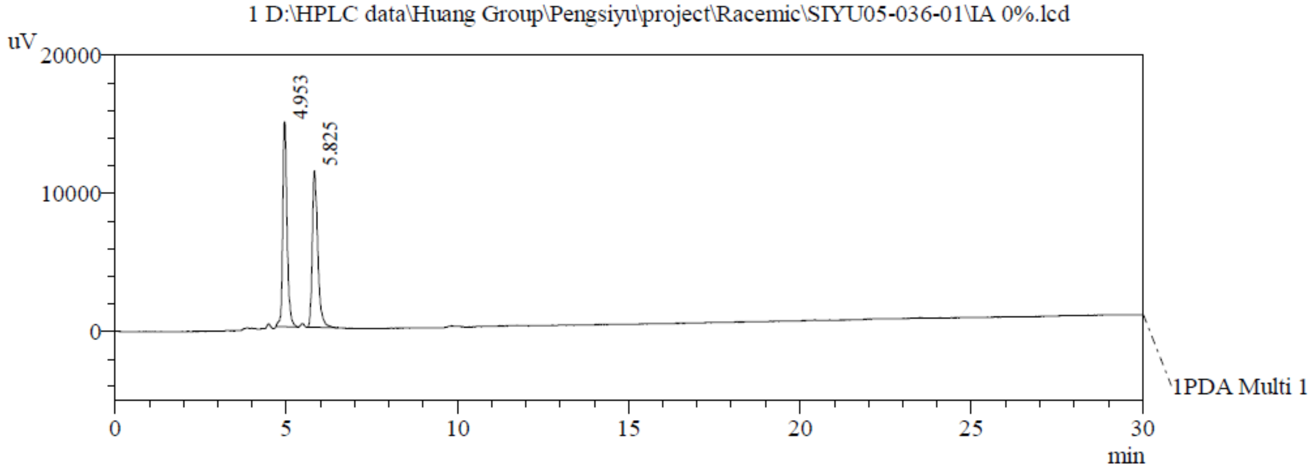

1 PDA Multi 1 / 254nm 4nm

PeakTable

PDA Ch1 254nm 4nm

Peak#	Ret. Time	Area	Height	Area %	Height %
1	4.953	131030	14883	50.711	56.730
2	5.825	127357	11352	49.289	43.270
Total		258387	26235	100.000	100.000

Supplementary Figure 126. HPLC traces for racemic product **4d**

Chromatogram
D:\HPLC data\Huang Group\Pengsiyu\project\Asymmetric\SIYU05-057\3 IA 0%.lcd

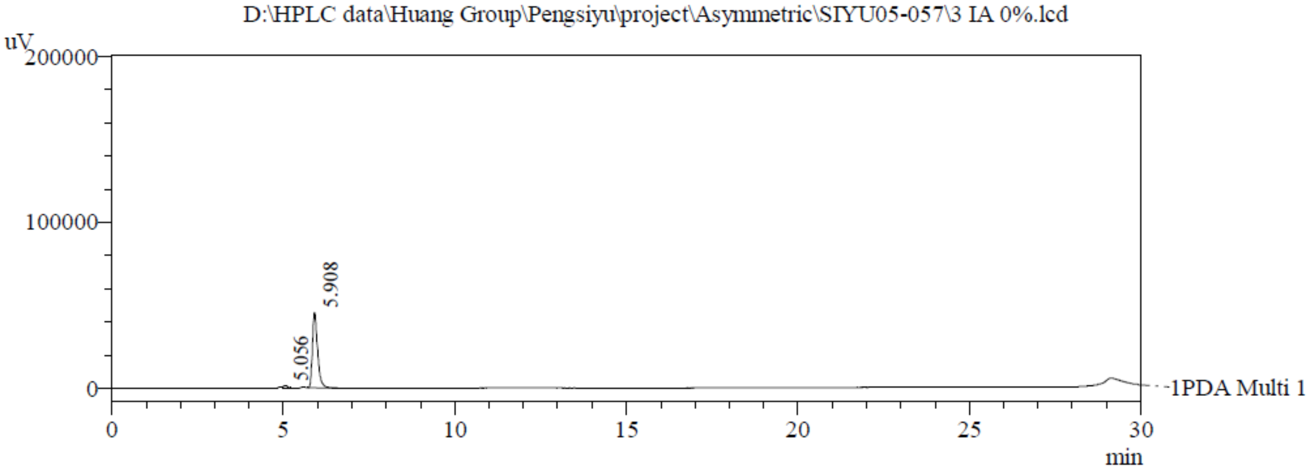

1 PDA Multi 1 / 254nm 4nm

PeakTable

PDA Ch1 254nm 4nm

Peak#	Ret. Time	Area	Height	Area %	Height %
1	5.056	12840	1710	2.611	3.611
2	5.908	478896	45634	97.389	96.389
Total		491736	47343	100.000	100.000

Supplementary Figure 127. HPLC traces for chiral product **4d**

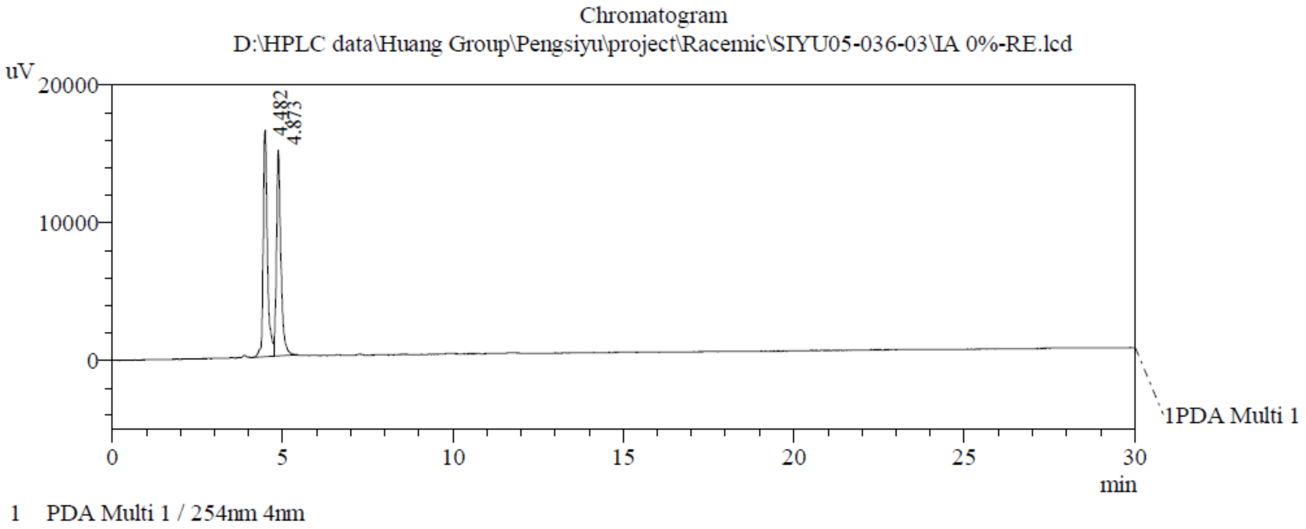

PeakTable

PDA Ch1 254nm 4nm

Peak#	Ret. Time	Area	Height	Area %	Height %
1	4.482	140431	16469	50.510	52.309
2	4.873	137594	15015	49.490	47.691
Total		278024	31484	100.000	100.000

Supplementary Figure 128. HPLC traces for racemic product **4e**

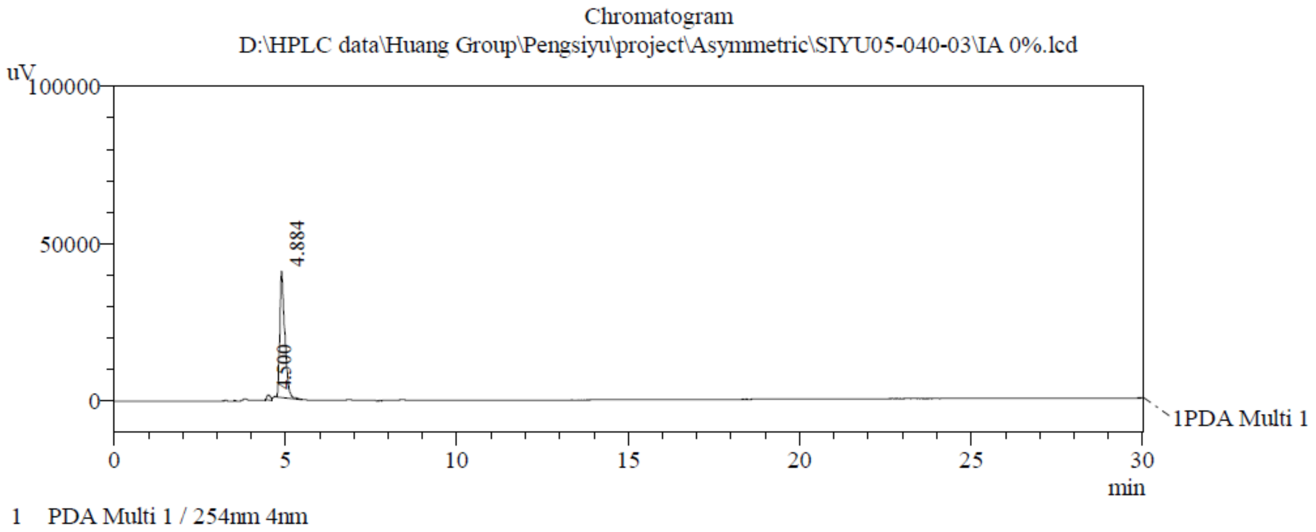

PeakTable

PDA Ch1 254nm 4nm

Peak#	Ret. Time	Area	Height	Area %	Height %
1	4.500	12402	1702	2.922	4.061
2	4.884	412019	40215	97.078	95.939
Total		424421	41917	100.000	100.000

Supplementary Figure 129. HPLC traces for chiral product **4e**

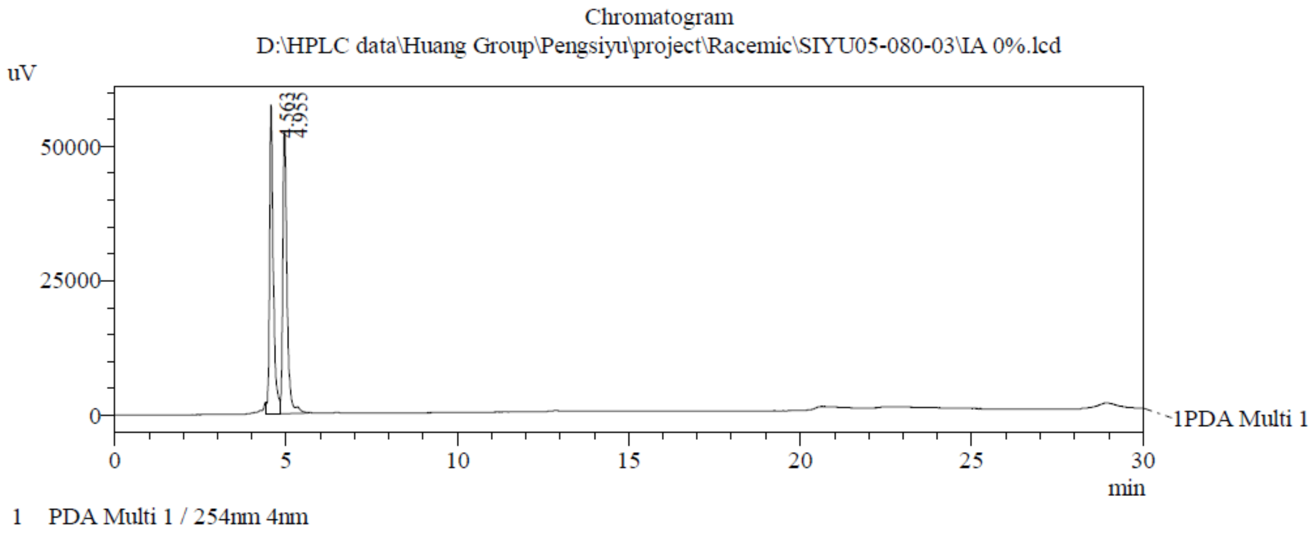

PeakTable

PDA Ch1 254nm 4nm

Peak#	Ret. Time	Area	Height	Area %	Height %
1	4.563	456780	57564	50.100	52.262
2	4.955	454951	52582	49.900	47.738
Total		911731	110145	100.000	100.000

Supplementary Figure 130. HPLC traces for racemic product **4f**

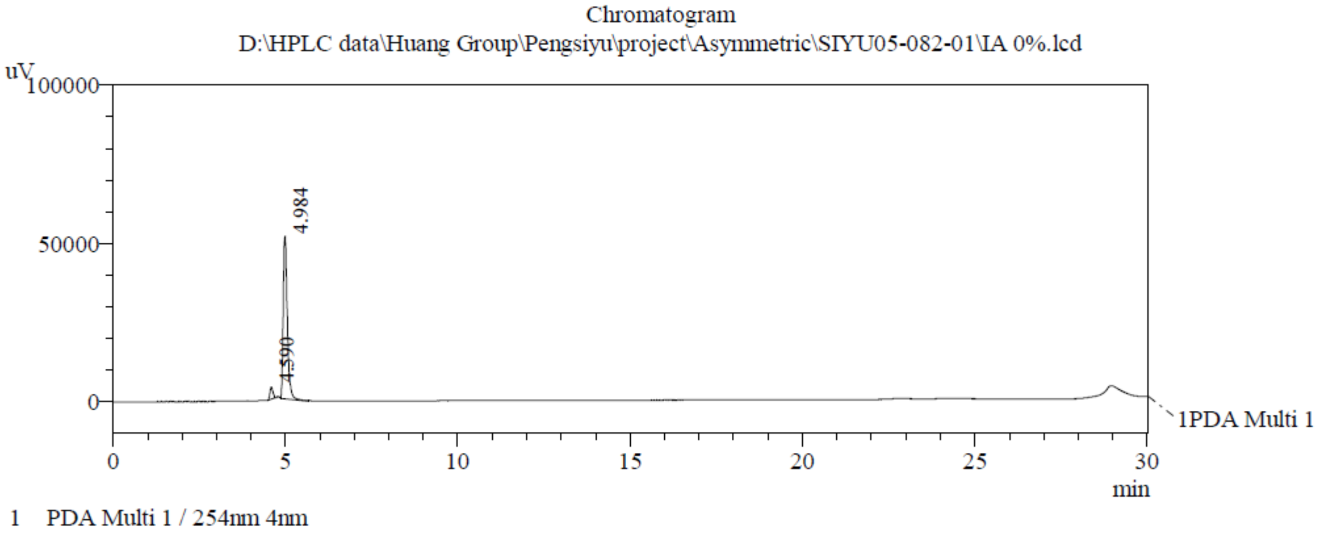

PeakTable

PDA Ch1 254nm 4nm

Peak#	Ret. Time	Area	Height	Area %	Height %
1	4.590	23303	3829	5.228	6.918
2	4.984	422416	51522	94.772	93.082
Total		445718	55351	100.000	100.000

Supplementary Figure 131. HPLC traces for chiral product **4f**

Chromatogram
1 D:\HPLC data\Huang Group\Pengsiyu\project\Racemic\SIYU05-030-01\IA 0%.lcd

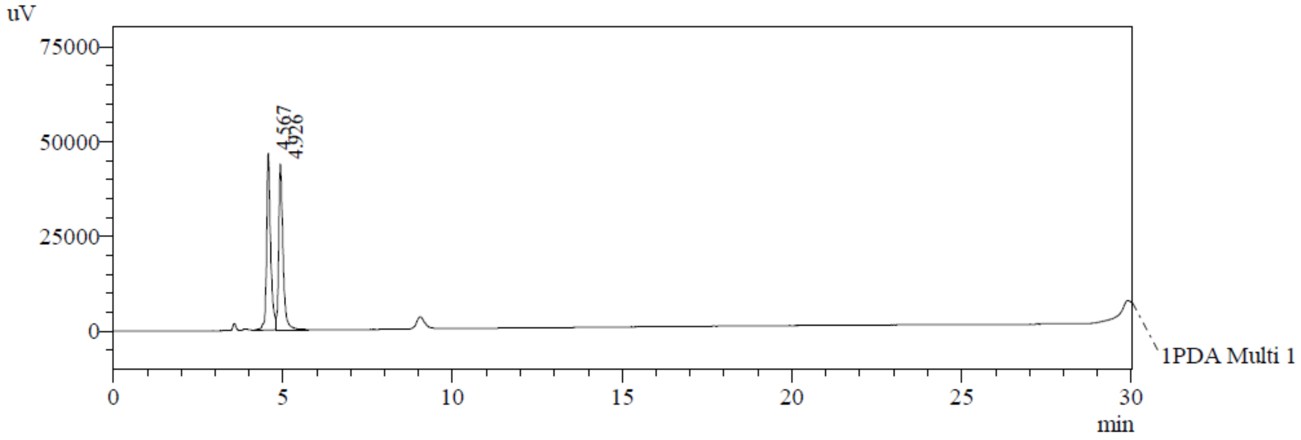

1 PDA Multi 1 / 254nm 4nm

PeakTable

PDA Ch1 254nm 4nm

Peak#	Ret. Time	Area	Height	Area %	Height %
1	4.567	395629	46573	50.262	51.623
2	4.926	391497	43645	49.738	48.377
Total		787126	90219	100.000	100.000

Supplementary Figure 132. HPLC traces for racemic product **4g**

Chromatogram
1 D:\HPLC data\Huang Group\Pengsiyu\project\Asymmetric\SIYU05-029-01\IA-0% inj1.lcd

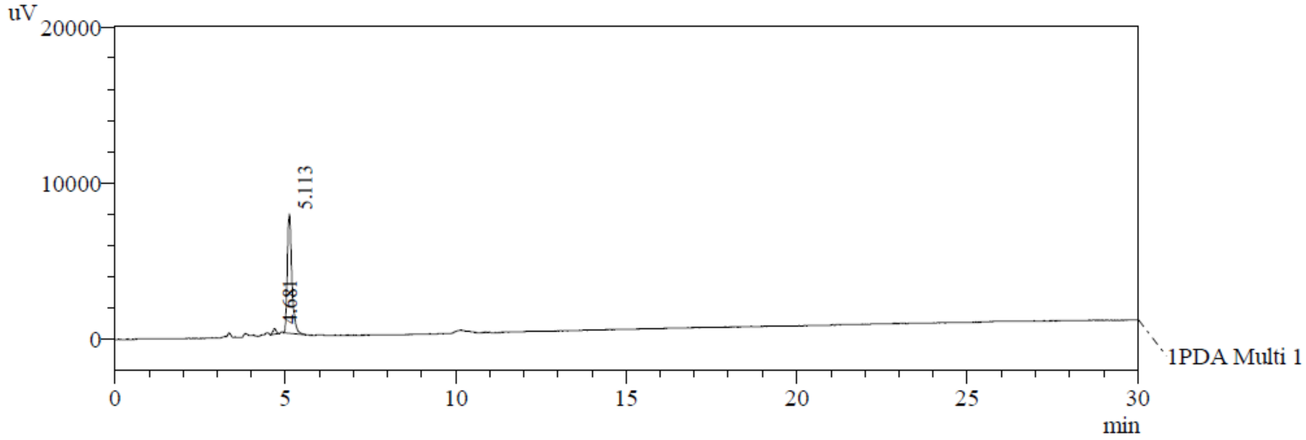

1 PDA Multi 1 / 254nm 4nm

PeakTable

PDA Ch1 254nm 4nm

Peak#	Ret. Time	Area	Height	Area %	Height %
1	4.681	2390	366	3.253	4.571
2	5.113	71085	7646	96.747	95.429
Total		73475	8012	100.000	100.000

Supplementary Figure 133. HPLC traces for chiral product **4g**

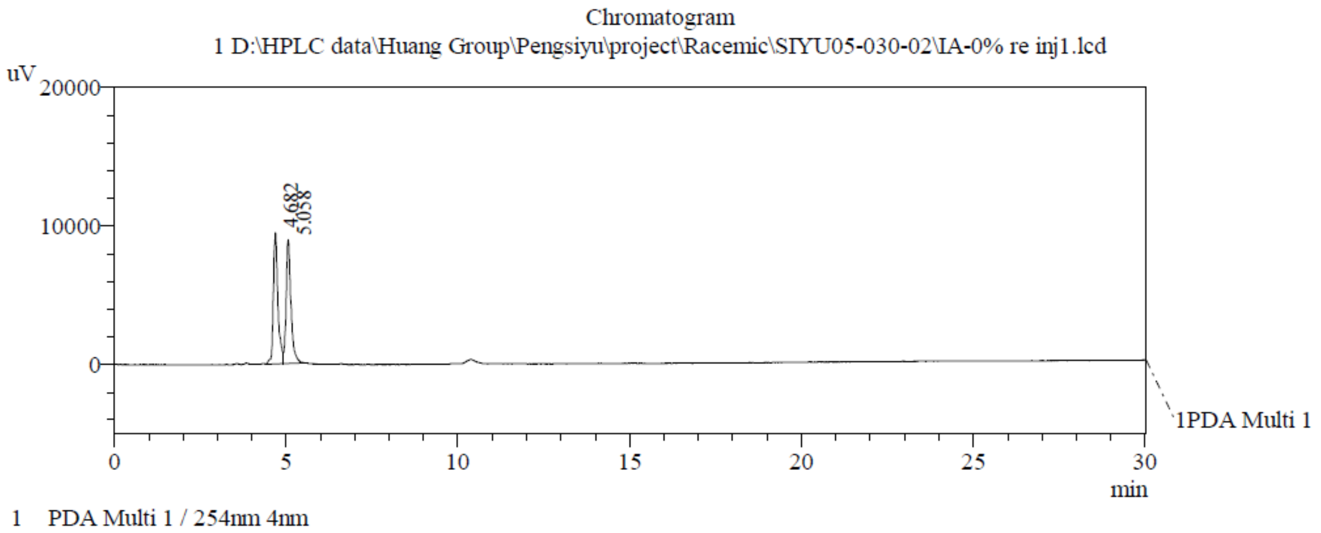

PeakTable

PDA Ch1 254nm 4nm

Peak#	Ret. Time	Area	Height	Area %	Height %
1	4.682	87301	9444	50.052	51.418
2	5.058	87118	8923	49.948	48.582
Total		174420	18368	100.000	100.000

Supplementary Figure 134. HPLC traces for racemic product **4h**

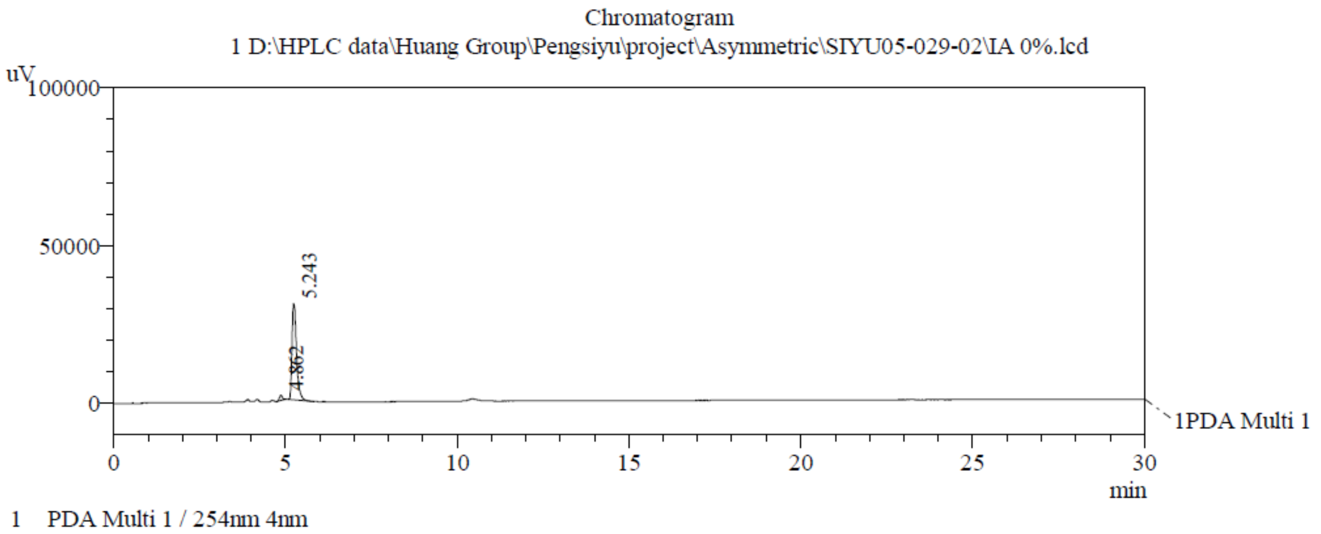

PeakTable

PDA Ch1 254nm 4nm

Peak#	Ret. Time	Area	Height	Area %	Height %
1	4.862	10703	1655	3.713	5.123
2	5.243	277534	30655	96.287	94.877
Total		288237	32311	100.000	100.000

Supplementary Figure 135. HPLC traces for chiral product **4h**

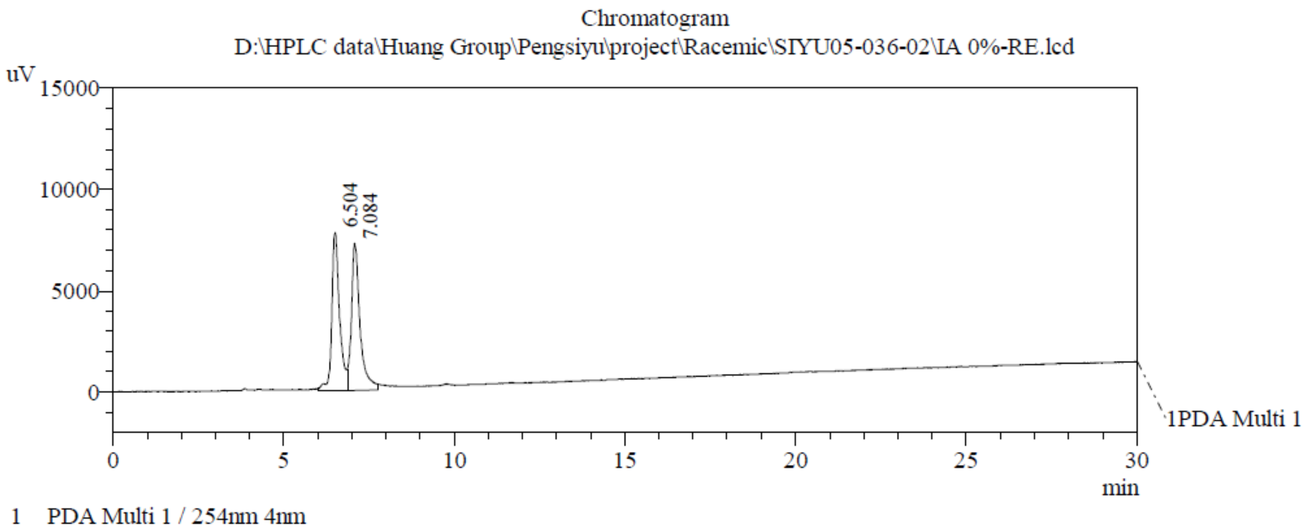

PeakTable

PDA Ch1 254nm 4nm

Peak#	Ret. Time	Area	Height	Area %	Height %
1	6.504	124615	7810	49.228	51.833
2	7.084	128526	7258	50.772	48.167
Total		253140	15068	100.000	100.000

Supplementary Figure 136. HPLC traces for racemic product **4i**

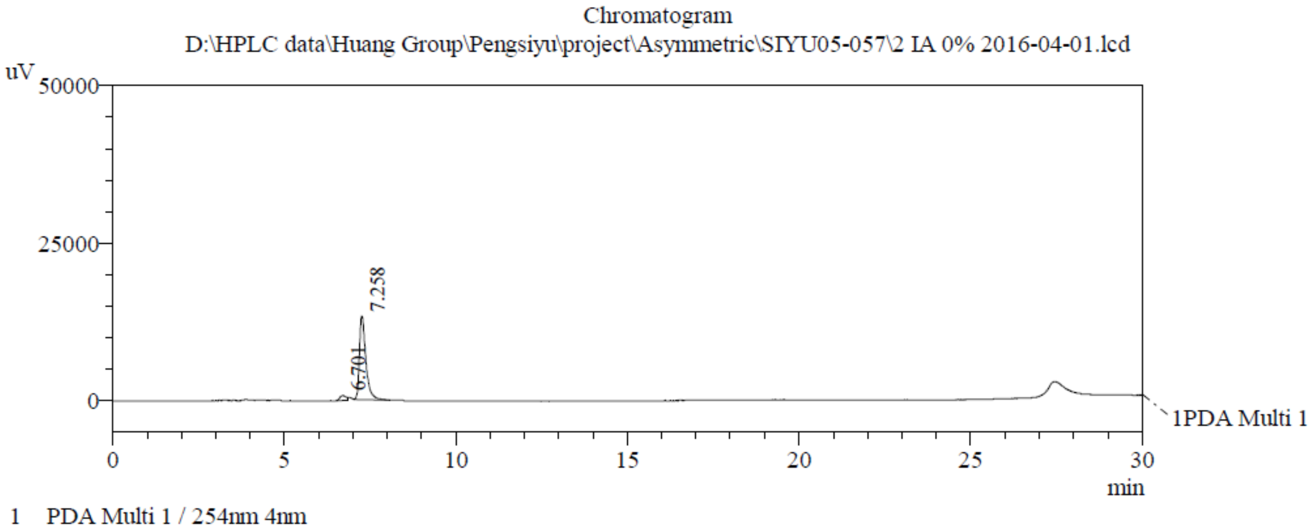

PeakTable

PDA Ch1 254nm 4nm

Peak#	Ret. Time	Area	Height	Area %	Height %
1	6.701	8779	781	4.812	5.551
2	7.258	173650	13283	95.188	94.449
Total		182429	14064	100.000	100.000

Supplementary Figure 137. HPLC traces for chiral product **4i**

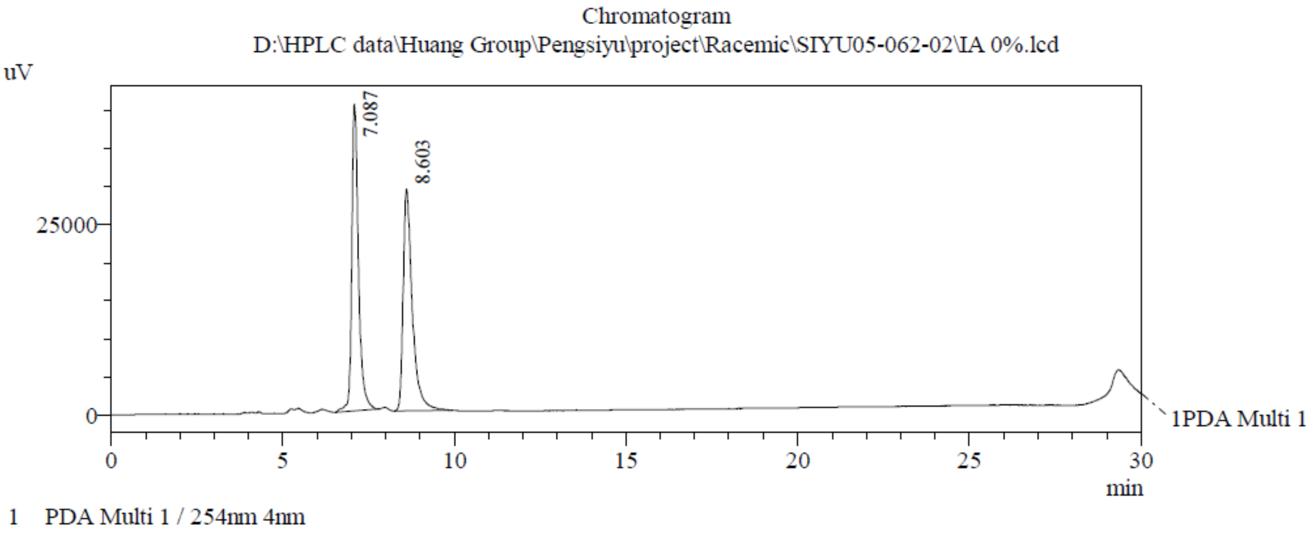

PeakTable

PDA Ch1 254nm 4nm

Peak#	Ret. Time	Area	Height	Area %	Height %
1	7.087	545951	40278	50.312	58.039
2	8.603	539177	29121	49.688	41.961
Total		1085128	69399	100.000	100.000

Supplementary Figure 138. HPLC traces for racemic product **4j**

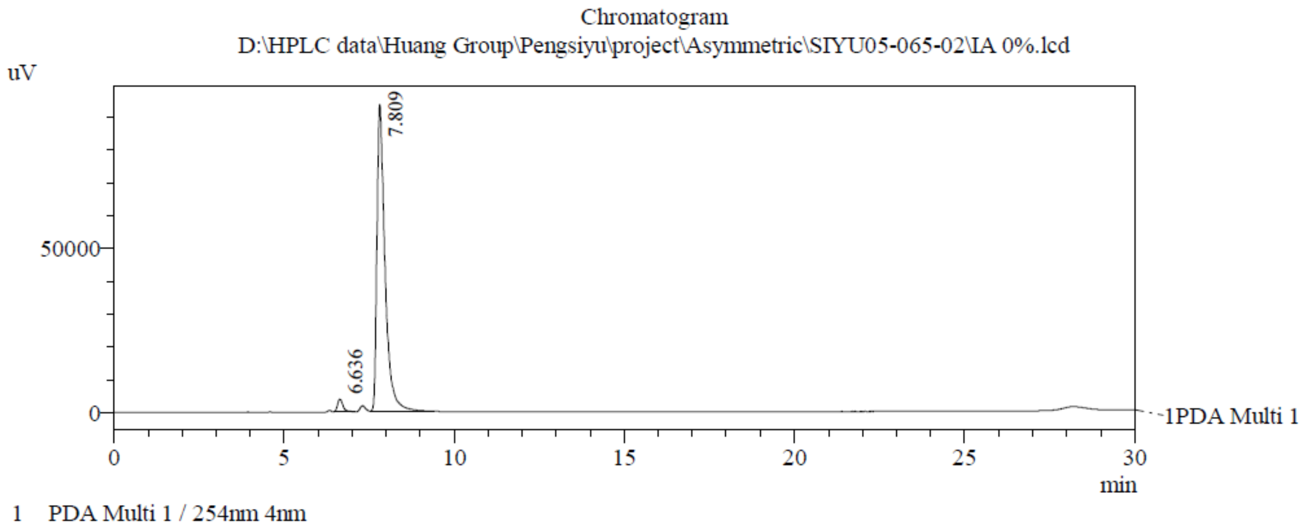

PeakTable

PDA Ch1 254nm 4nm

Peak#	Ret. Time	Area	Height	Area %	Height %
1	6.636	45519	3943	2.864	4.045
2	7.809	1544091	93541	97.136	95.955
Total		1589609	97485	100.000	100.000

Supplementary Figure 139. HPLC traces for chiral product **4j**

Chromatogram
D:\HPLC data\Huang Group\Pengsiyu\project\Racemic\SIYU05-073-03\IA 0%.lcd

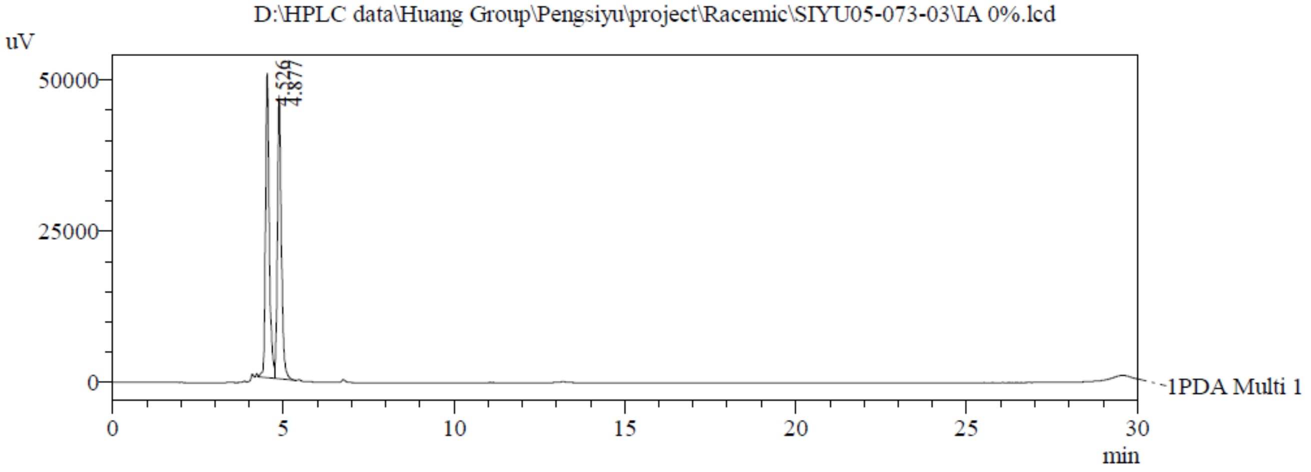

1 PDA Multi 1 / 254nm 4nm

PeakTable

PDA Ch1 254nm 4nm

Peak#	Ret. Time	Area	Height	Area %	Height %
1	4.526	390217	50369	50.516	51.818
2	4.877	382240	46834	49.484	48.182
Total		772457	97204	100.000	100.000

Supplementary Figure 140. HPLC traces for racemic product **4k**

Chromatogram
D:\HPLC data\Huang Group\Pengsiyu\project\Asymmetric\SIYU05-073-04\IA 0%-RE.lcd

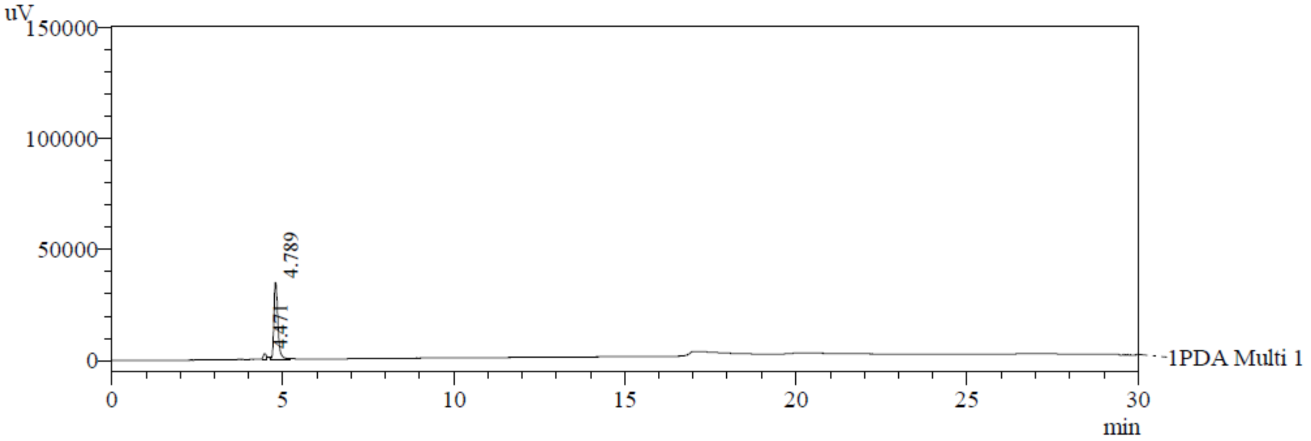

1 PDA Multi 1 / 254nm 4nm

PeakTable

PDA Ch1 254nm 4nm

Peak#	Ret. Time	Area	Height	Area %	Height %
1	4.471	14778	2437	5.133	6.616
2	4.789	273097	34394	94.867	93.384
Total		287875	36831	100.000	100.000

Supplementary Figure 141. HPLC traces for chiral product **4k**

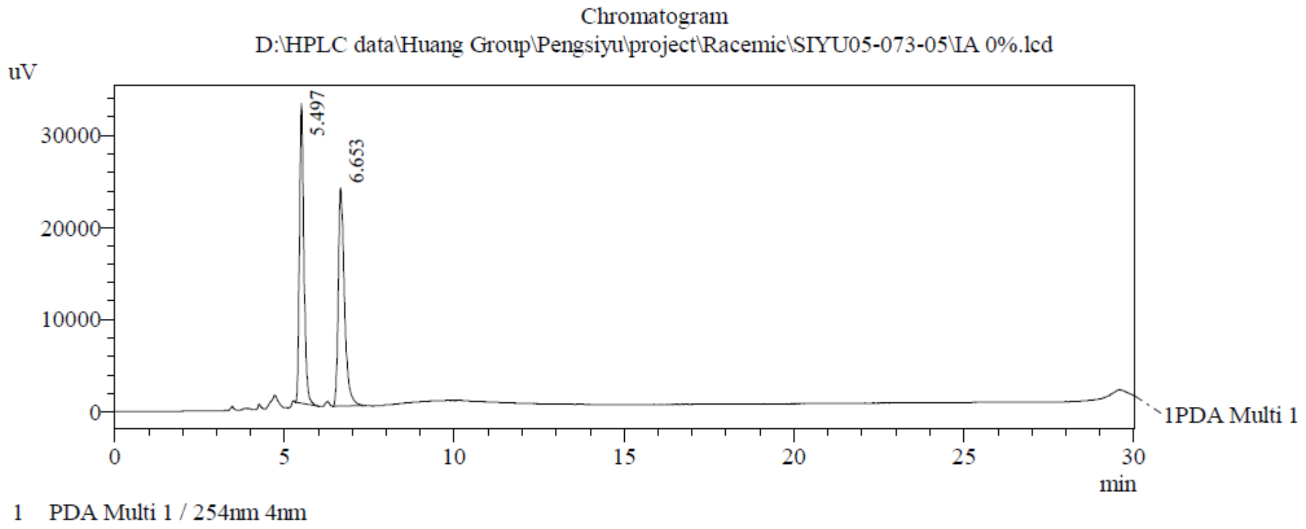

PeakTable

PDA Ch1 254nm 4nm

Peak#	Ret. Time	Area	Height	Area %	Height %
1	5.497	296594	32586	49.562	57.818
2	6.653	301835	23774	50.438	42.182
Total		598429	56360	100.000	100.000

Supplementary Figure 142. HPLC traces for racemic product **4l**

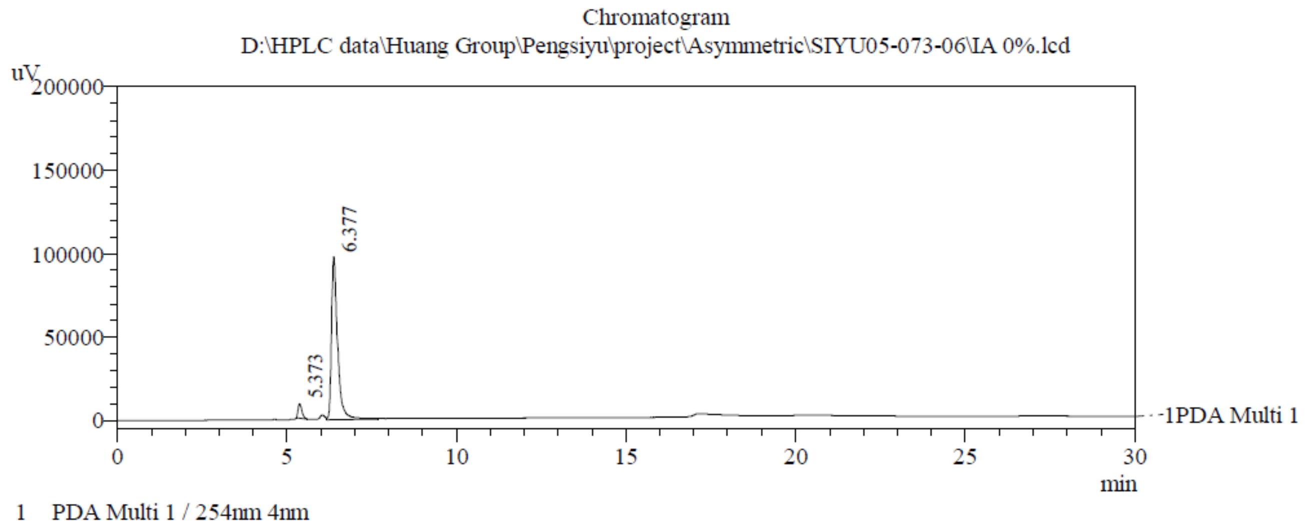

PeakTable

PDA Ch1 254nm 4nm

Peak#	Ret. Time	Area	Height	Area %	Height %
1	5.373	65533	8604	5.078	8.107
2	6.377	1224974	97527	94.922	91.893
Total		1290507	106131	100.000	100.000

Supplementary Figure 143. HPLC traces for chiral product **4l**

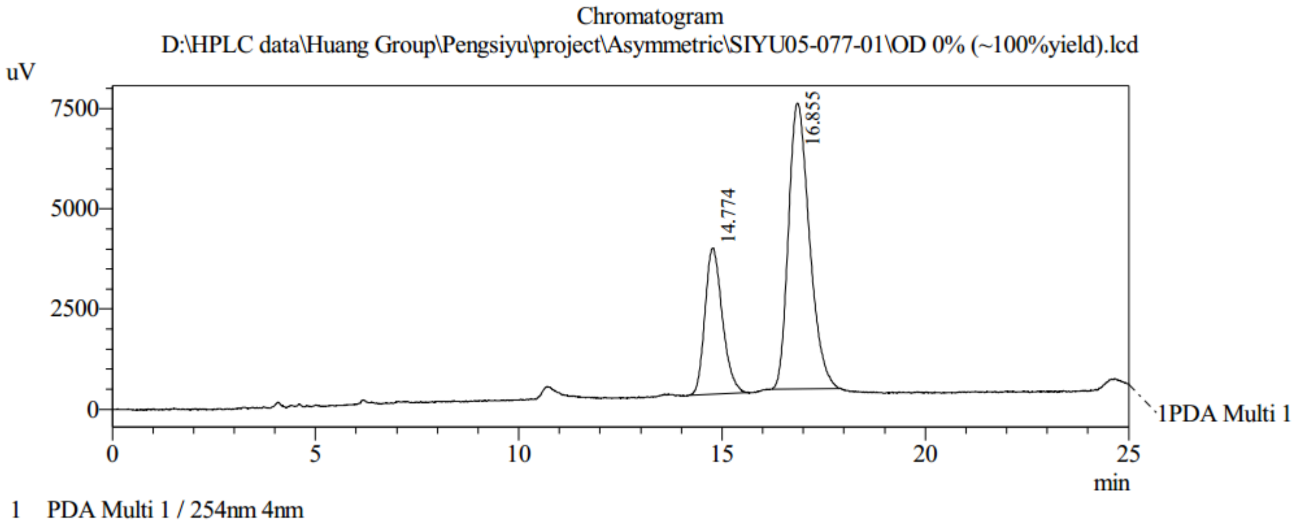

PeakTable

PDA Ch1 254nm 4nm

Peak#	Ret. Time	Area	Height	Area %	Height %
1	14.774	108492	3640	29.702	33.776
2	16.855	256774	7138	70.298	66.224
Total		365266	10778	100.000	100.000

Supplementary Figure 144. HPLC traces for racemic product **4m**

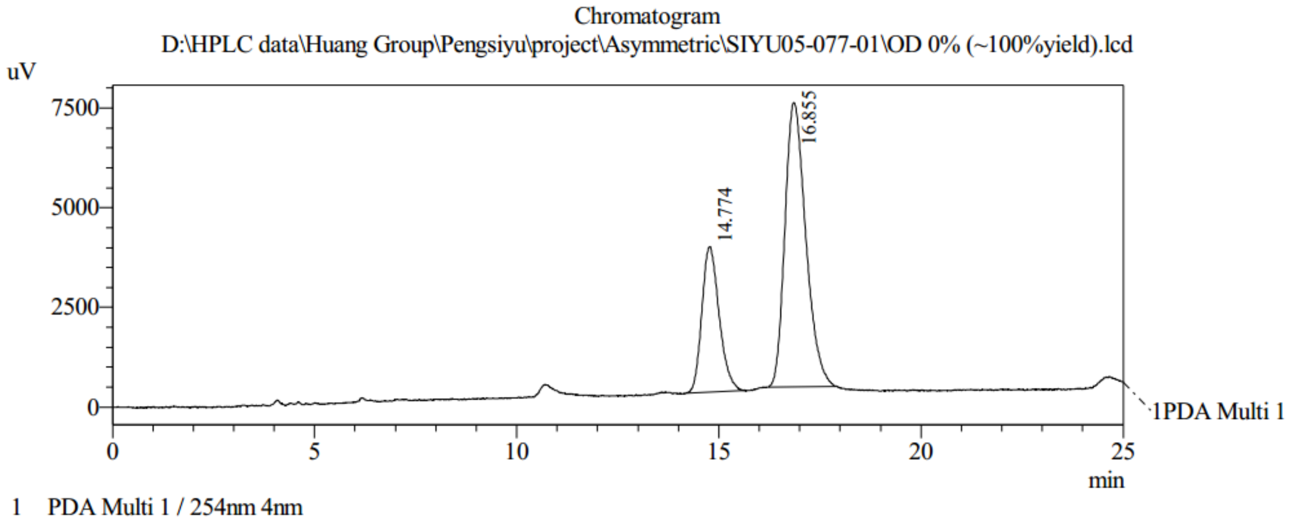

PeakTable

PDA Ch1 254nm 4nm

Peak#	Ret. Time	Area	Height	Area %	Height %
1	14.774	108492	3640	29.702	33.776
2	16.855	256774	7138	70.298	66.224
Total		365266	10778	100.000	100.000

Supplementary Figure 145. HPLC traces for chiral product **4m**

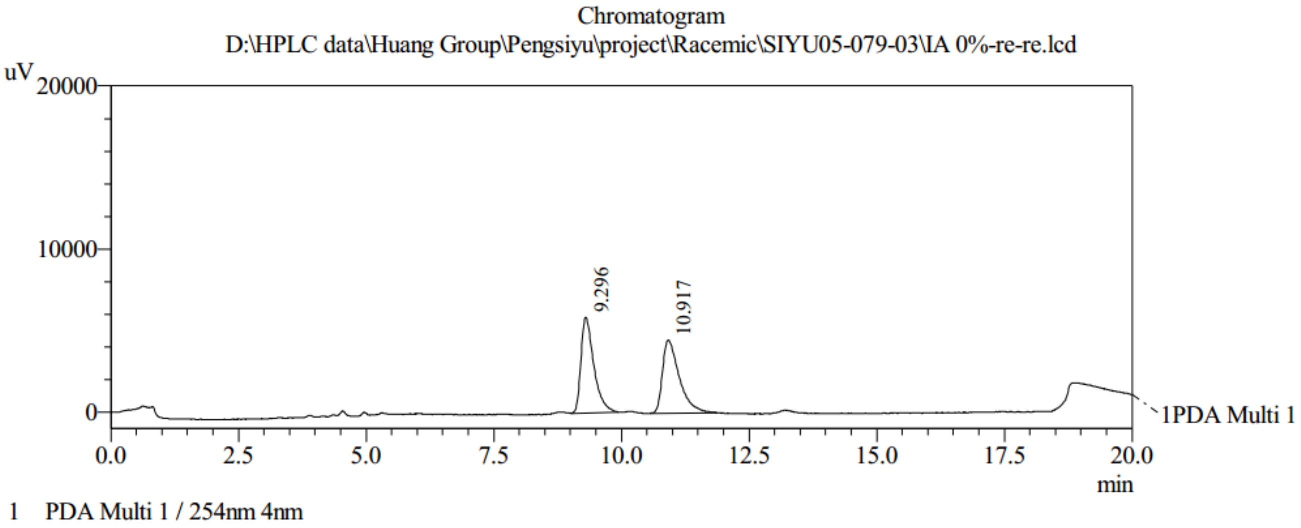

PeakTable

PDA Ch1 254nm 4nm

Peak#	Ret. Time	Area	Height	Area %	Height %
1	9.296	102716	5846	50.234	56.595
2	10.917	101760	4484	49.766	43.405
Total		204476	10330	100.000	100.000

Supplementary Figure 146. HPLC traces for racemic product **4n**

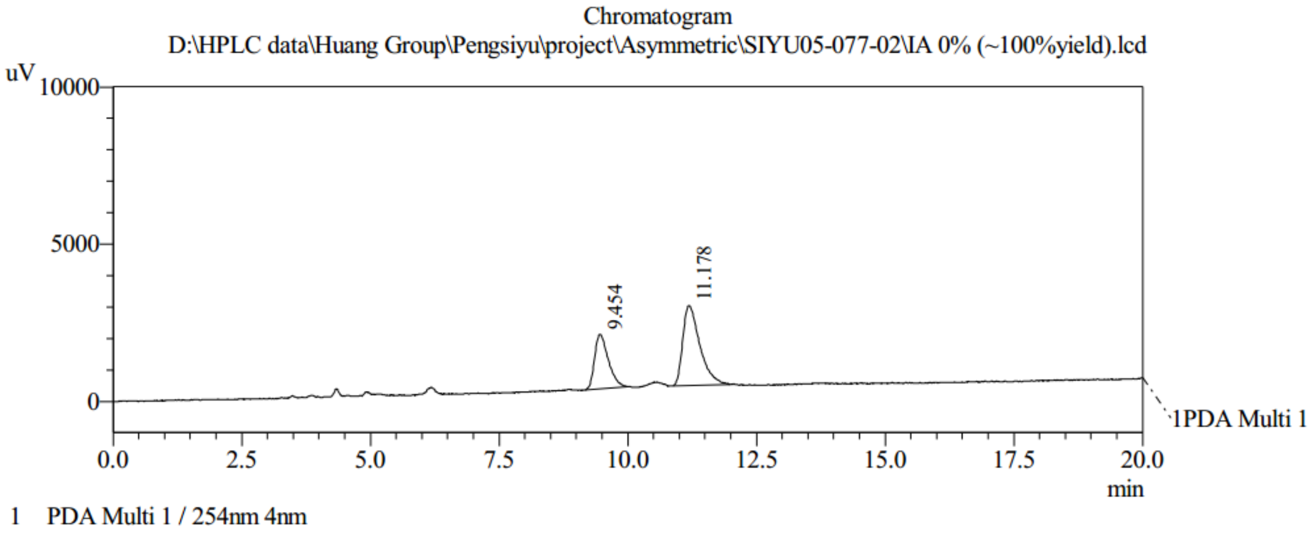

PeakTable

PDA Ch1 254nm 4nm

Peak#	Ret. Time	Area	Height	Area %	Height %
1	9.454	32532	1739	35.508	40.545
2	11.178	59087	2550	64.492	59.455
Total		91619	4290	100.000	100.000

Supplementary Figure 147. HPLC traces for chiral product **4n**

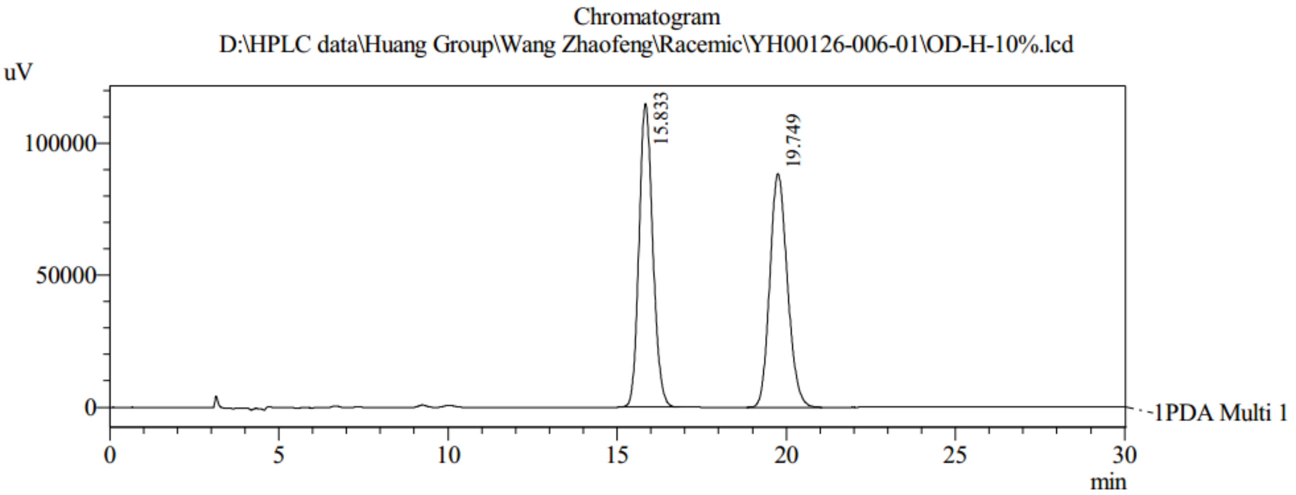

1 PDA Multi 1 / 254nm 4nm

PeakTable

PDA Ch1 254nm 4nm

Peak#	Ret. Time	Area	Height	Area %	Height %
1	15.833	3250358	115085	50.326	56.534
2	19.749	3208288	88482	49.674	43.466
Total		6458646	203567	100.000	100.000

Supplementary Figure 148. HPLC traces for racemic product **9**

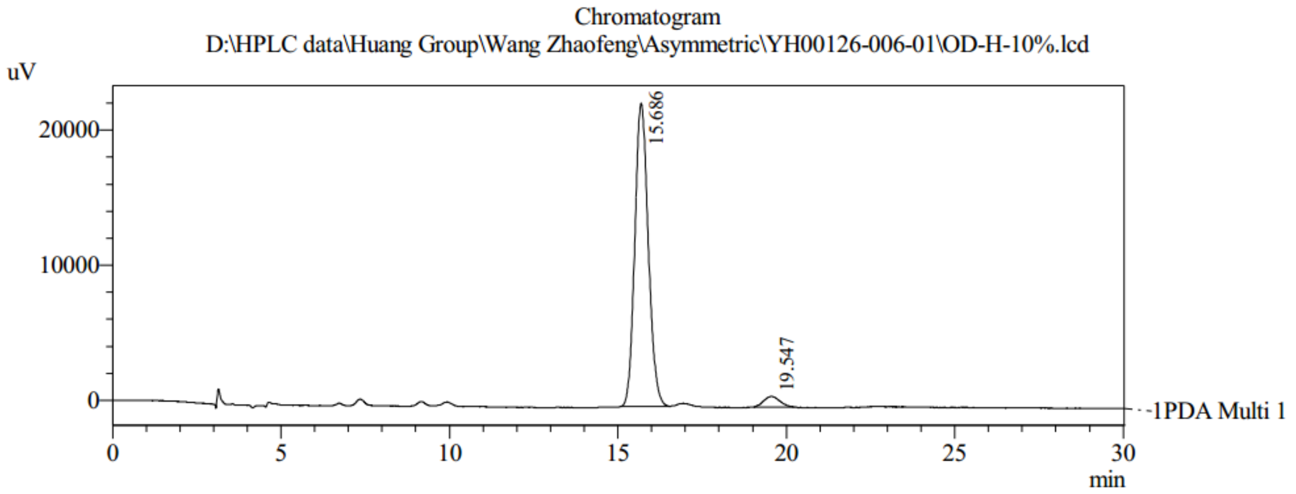

1 PDA Multi 1 / 254nm 4nm

PeakTable

PDA Ch1 254nm 4nm

Peak#	Ret. Time	Area	Height	Area %	Height %
1	15.686	625412	22483	95.933	96.576
2	19.547	26516	797	4.067	3.424
Total		651928	23280	100.000	100.000

Supplementary Figure 149. HPLC traces for chiral product **9**

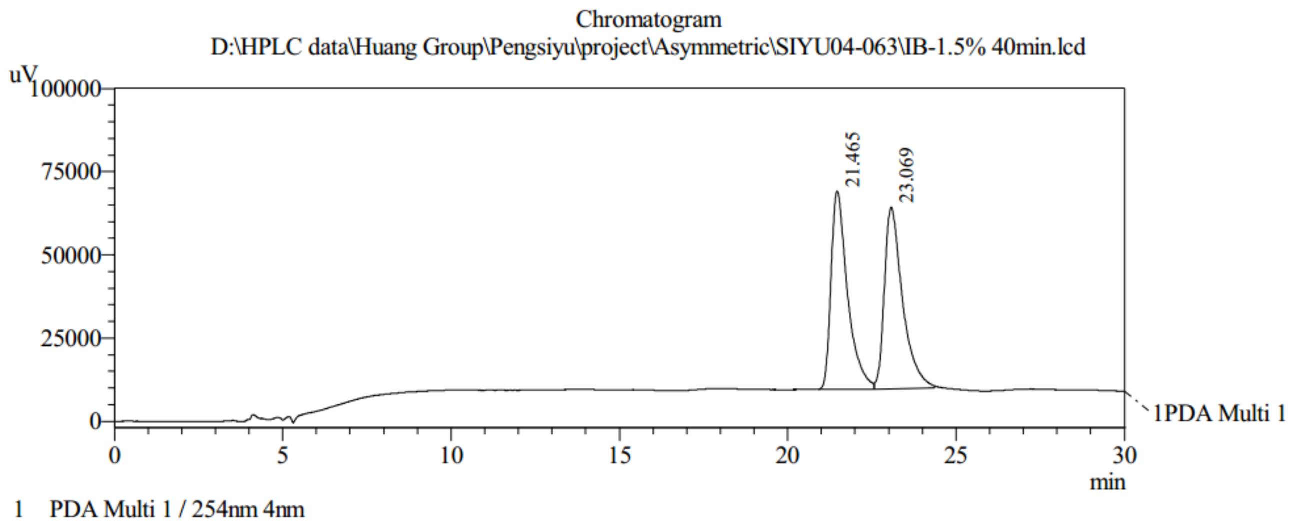

PeakTable

PDA Ch1 254nm 4nm

Peak#	Ret. Time	Area	Height	Area %	Height %
1	21.465	2044204	59542	49.862	52.179
2	23.069	2055483	54570	50.138	47.821
Total		4099687	114113	100.000	100.000

Supplementary Figure 150. HPLC traces for racemic product **10**

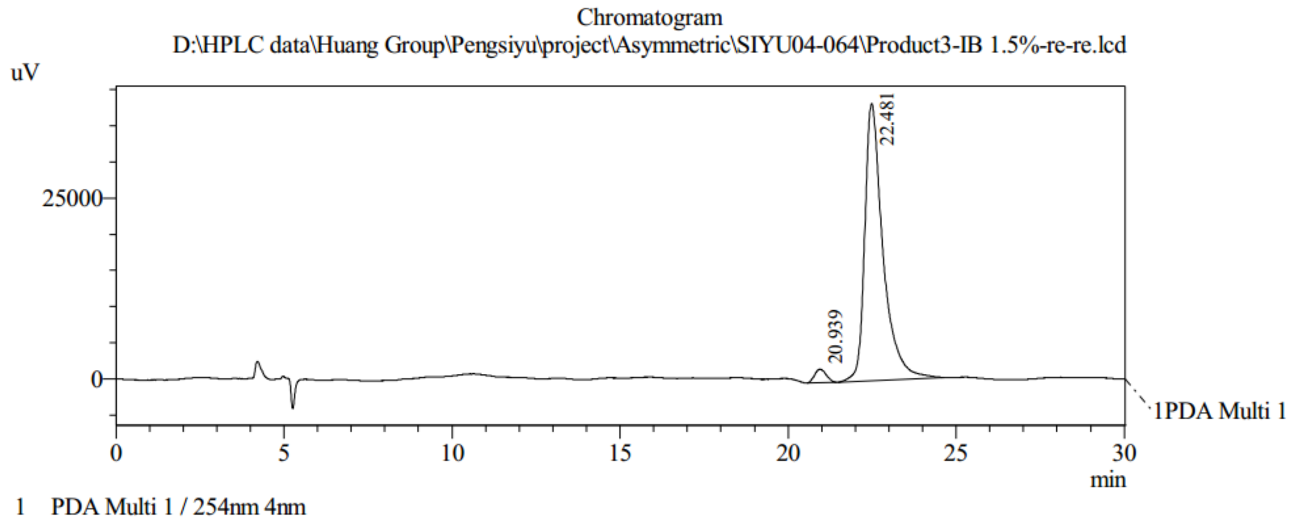

PeakTable

PDA Ch1 254nm 4nm

Peak#	Ret. Time	Area	Height	Area %	Height %
1	20.939	45252	1874	3.033	4.666
2	22.481	1446920	38293	96.967	95.334
Total		1492172	40167	100.000	100.000

Supplementary Figure 151. HPLC traces for chiral product **10**

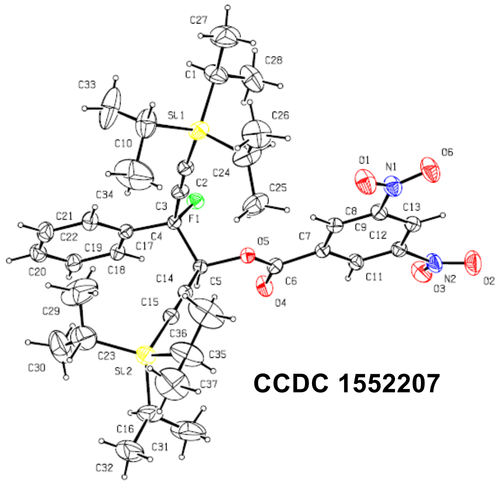

CCDC 1552207

≡

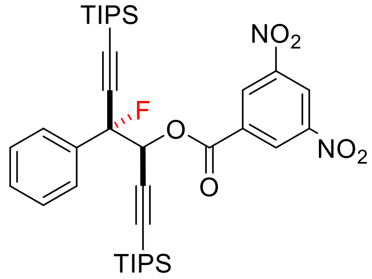

7
(Derivative of **4a**)

Supplementary Figure 152. ORTEP presentation of the compound **7**

Supplementary methods

Analytical data of diyneamines:

(1) **(*trans*)-1-(4-phenyl-1,6-bis(triisopropylsilyl)hexa-3-en-1,5-diyn-3-yl)pyrrolidine 3a** (DYE 1)

(47.0 mg, 0.088 mmol, 88% yield), yellow oil. *trans*:*cis* ratio > 20:1. **^{1}H NMR** (400 MHz, $CDCl_3$) δ 7.63 – 7.61 (m, 2H), 7.21 – 7.17 (m, 2H), 7.10 – 7.06 (m, 1H), 3.74 – 3.70 (m, 4H), 1.90 – 1.86 (m, 4H), 1.07 – 1.06 (m, 18H, 3H, 3H), 0.95 (s, 18H); **^{13}C NMR** (101 MHz, $CDCl_3$) δ 140.36, 136.38, 129.85, 127.41, 125.60, 109.39, 102.62, 101.09, 100.20, 97.96, 51.58, 25.82, 18.93, 18.66, 11.87, 11.33; **HRMS** (ESI-TOF) [M+H] calculated for $[C_{34}H_{56}NSi_2]^+$ 534.3951, found 534.3946.

(2) **(trans)-1-(4-(m-tolyl)-1,6-bis(triisopropylsilyl)hexa-3-en-1,5-diyn-3-yl)pyrrolidine 3b** (DYE 2)

(47.1 mg, 0.086 mmol, 86% yield), yellow oil. *trans*:*cis* ratio = 10.2:1. **^{1}H NMR** (*major isomer*) (400 MHz, $CDCl_3$) δ 7.45 – 7.43 (m, 2H), 7.09 (dd, *J* = 7.6, 7.6 Hz, 1H), 6.90 (d, *J* = 7.6 Hz, 1H), 3.72 – 3.69 (m, 4H), 2.28 (s, 3H), 1.89 – 1.86 (m, 4H), 1.14 – 1.08 (m, 18H, 3H, 3H), 0.96 (s, 18H); **^{13}C NMR** (*major isomer*) (101 MHz, $CDCl_3$) δ 140.18, 136.59, 136.21, 130.63, 127.34, 126.89, 126.40, 109.49, 102.65, 101.21, 100.07, 97.93, 51.58, 25.83, 21.55, 18.93, 18.65, 11.87, 11.32; **HRMS** (ESI-TOF) [M+H] calculated for $[C_{35}H_{58}NSi_2]^+$ 548.4108, found 548.4102.

(3) **(trans)-1-(4-(p-tolyl)-1,6-bis(triisopropylsilyl)hexa-3-en-1,5-diyn-3-yl)pyrrolidine 3c** (DYE 3)

(44.9 mg, 0.082 mmol, 82% yield), yellow oil. *trans*:*cis* ratio = 9.1:1. **^{1}H NMR** (*major isomer*) (400 MHz, $CDCl_3$) δ 7.50 (d, *J* = 8.0 Hz, 2H), 7.00 (d, *J* = 8.0 Hz, 2H), 3.72 – 3.68 (m, 4H), 2.28 (s, 3H), 1.89 – 1.85 (m, 4H), 1.12 – 1.07 (m, 18H, 3H, 3H), 0.95 (s, 18H); **^{13}C NMR** (*major isomer*) (101 MHz, $CDCl_3$) δ 137.39, 136.13, 135.11, 129.68, 128.14, 109.44, 102.75, 101.20, 100.06, 97.87, 51.53, 25.81, 21.22, 18.93, 18.63, 11.86, 11.33; **HRMS** (ESI-TOF) [M+H] calculated for $[C_{35}H_{58}NSi_2]^+$ 548.4108, found 548.4098.

(4) **(trans)-1-(4-(4-(tert-butyl)phenyl)-1,6-bis(triisopropylsilyl)hexa-3-en-1,5-diyn-3-yl)pyrrolidine 3d** (DYE 4)

(46.0 mg, 0.078 mmol, 78% yield), yellow oil. *trans*:*cis* ratio = 19.4:1. **^{1}H NMR** (*major isomer*) (500 MHz, $CDCl_3$) δ 7.54 (d, *J* = 8.4 Hz, 2H), 7.21 (d, *J* = 8.4 Hz, 2H), 3.71 – 3.69 (m, 4H), 1.89 – 1.86 (m, 4H), 1.29 (s, 9H), 1.12 – 1.07 (m, 18H, 3H, 3H), 0.95 (s, 18H); **^{13}C NMR** (*major isomer*) (126 MHz, $CDCl_3$) δ 148.34, 137.32, 136.31, 129.49, 124.35, 109.47, 102.96, 101.59, 99.90, 98.00, 51.54, 34.51, 31.54, 25.81, 18.96, 18.71, 11.95, 11.42; **HRMS** (ESI-TOF) [M+H] calculated for $[C_{38}H_{64}NSi_2]^+$ 590.4572, found 590.4571.

(5) **(trans)-1-(4-(4-methoxyphenyl)-1,6-bis(triisopropylsilyl)hexa-3-en-1,5-diyn-3-yl)pyrrolidine 3e** (DYE 5)

(42.3 mg, 0.075 mmol, 75% yield), yellow oil. *trans*:*cis* ratio > 20:1. **^{1}H NMR** (400 MHz, $CDCl_3$) δ 7.54 (d, *J* = 8.8 Hz, 2H), 6.76 (d, *J* = 8.8 Hz, 2H), 3.78 (s, 3H), 3.72 – 3.68 (m, 4H), 1.89 – 1.86 (m, 4H), 1.09 – 1.07 (m, 18H, 3H, 3H), 0.97 (s, 18H); **^{13}C NMR** (101 MHz, $CDCl_3$) δ 157.85, 135.82, 133.00, 130.91, 112.99, 109.56, 102.83, 100.98, 99.96, 97.97, 55.41, 51.50, 25.80, 18.92, 18.67, 11.86, 11.35; **HRMS** [M+H] calculated for $[C_{35}H_{58}NOSi_2]^+$ 564.4051, found 564.4056.

(6) **(trans)-1-(4-(3-methoxyphenyl)-1,6-bis(triisopropylsilyl)hexa-3-en-1,5-diyn-3-yl)pyrrolidine 3f** (DYE 6)

(40.0 mg, 0.071 mmol, 71% yield), yellow oil. *trans*:*cis* ratio = 9.8:1. **^{1}H NMR** (*major isomer*) (500 MHz, $CDCl_3$) δ 7.23 (d, *J* = 8.0 Hz, 1H), 7.16 (dd, *J* = 2.0, 2.0 Hz, 1H), 7.09 (dd, *J* = 8.0, 8.0 Hz, 1H), 6.66 (dd, *J* = 8.0, 2.0 Hz, 1H), 3.77 (s, 3H), 3.73 – 3.70 (m, 4H), 1.89 – 1.87 (m, 4H), 1.11 – 1.06 (m, 18H, 3H, 3H), 0.96 (s, 18H); **^{13}C NMR** (*major isomer*) (126 MHz, $CDCl_3$) δ 159.11, 141.85, 136.58, 128.23, 122.79, 114.91, 112.11, 109.38, 102.58, 101.09, 100.47, 98.06, 55.20, 51.58, 25.81, 18.95, 18.67, 11.92, 11.39; **HRMS** [M+H] calculated for $[C_{35}H_{58}NOSi_2]^+$ 564.4051, found 564.4050.

(7) **(trans)-1-(4-(4-nitrophenyl)-1,6-bis(triisopropylsilyl)hexa-3-en-1,5-diyn-3-yl)pyrrolidine 3g** (DYE 7)

(36.5 mg, 0.063 mmol, 63% yield), yellow oil. *trans*:*cis* ratio > 20:1. **^{1}H NMR** (400 MHz, $CDCl_3$) δ 8.04 (d, *J* = 8.9 Hz, 2H), 7.81 (d, *J* = 8.9 Hz, 2H), 3.79 – 3.75 (m, 4H), 1.94 – 1.90 (m, 4H), 1.09 – 1.07 (m, 18H, 3H), 0.99 – 0.93(m, 18H, 3H); **^{13}C NMR** (101 MHz, $CDCl_3$) δ 148.06, 144.81, 138.14, 129.56, 122.85, 107.62, 102.49, 101.59, 98.81, 97.96, 51.99, 25.80, 18.92, 18.64, 11.83, 11.29; **HRMS** (ESI-TOF) [M+H] calculated for $[C_{34}H_{55}N_2O_2Si_2]^+$ 578.3797, found 578.3797.

(8) **(trans)-1-(4-(3-fluorophenyl)-1,6-bis(triisopropylsilyl)hexa-3-en-1,5-diyn-3-yl)pyrrolidine 3h** (DYE 8)

(36.4 mg, 0.066 mmol, 66% yield), yellow oil. *trans*:*cis* ratio = 14.7:1. **^{1}H NMR** (*major isomer*) (500 MHz, $CDCl_3$) δ 7.41 (d, *J* = 8.3 Hz, 1H), 7.34 (ddd, *J* = 10.9, 2.4, 2.4 Hz, 1H), 7.16 – 7.12 (m, 1H), 6.78 (ddd, *J* = 8.5, 8.5, 2.4 Hz, 1H), 3.74 – 3.71 (m, 4H), 1.90 – 1.87 (m, 4H), 1.12 – 1.08 (m, 18H, 3H), 0.98 – 0.96 (m, 18H, 3H); **^{13}C NMR** (*major isomer*) (126 MHz, $CDCl_3$) δ 162.41(d, *J* = 243.2 Hz), 142.91(d, *J* = 8.1 Hz), 136.71, 128.59(d, *J* = 8.6 Hz), 125.50(d, *J* = 2.6 Hz), 116.62(d, *J* = 22.0 Hz), 112.26(d, *J* = 21.2 Hz), 108.93, 102.25, 101.07, 99.66, 98.27, 51.63, 25.81, 18.92, 18.65, 11.91, 11.36; **^{19}F NMR** (*major isomer*) (376 MHz, $CDCl_3$) δ -115.48; **HRMS** (ESI-TOF) [M+H] calculated for $[C_{34}H_{55}FNSi_2]^+$ 552.3852, found 552.3850.

(9) **(trans)-1-(4-(3-chlorophenyl)-1,6-bis(triisopropylsilyl)hexa-3-en-1,5-diyn-3-yl)pyrrolidine 3i** (DYE 9)

(47.7 mg, 0.084 mmol, 84% yield), yellow oil. *trans*:*cis* ratio = 18:1. **^{1}H NMR** (*major isomer*) (300 MHz, $CDCl_3$) δ 7.62 (dd, *J* = 2.0, 2.0 Hz, 1H), 7.52 (ddd, *J* = 8.0, 2.0, 2.0 Hz, 1H), 7.15 – 7.03 (m, 2H), 3.75 – 3.70 (m, 4H), 1.91 – 1.87 (m, 4H), 1.08 – 1.07 (m, 18H, 3H), 0.98 – 0.96 (m, 18H, 3H); **^{13}C NMR** (*major isomer*) (75 MHz, $CDCl_3$) δ 142.47, 136.65, 133.10, 129.86, 128.58, 127.84, 125.43, 108.78, 102.02, 100.97, 98.99, 98.11, 51.63, 25.81, 18.91, 18.65, 11.83, 11.25; **HRMS** (ESI-TOF) [M+H] calculated for $[C_{34}H_{55}ClNSi_2]^+$ 568.3556, found 568.3552.

(10) **(trans)-1-(4-(3-bromophenyl)-1,6-bis(triisopropylsilyl)hexa-3-en-1,5-diyn-3-yl)pyrrolidine 3j** (DYE 10)

(51.5 mg, 0.084 mmol, 84% yield), yellow oil. *trans*:*cis* ratio = 14.9:1. **^{1}H NMR** (*major isomer*) (500 MHz, $CDCl_3$) δ 7.76 (dd, *J* = 1.5, 1.5 Hz, 1H), 7.56 (ddd, *J* = 7.8, 1.5, 1.5 Hz, 1H), 7.22 – 7.19 (m, 1H), 7.06 (dd, *J* = 7.9, 7.9 Hz, 1H), 3.73 – 3.71 (m, 4H), 1.90 – 1.87 (m, 4H), 1.09 – 1.07 (m, 18H, 3H), 0.99 – 0.96 (m, 18H, 3H); **^{13}C NMR** (*major isomer*) (126 MHz, $CDCl_3$) δ 142.96, 136.66, 132.83, 128.90, 128.42, 128.39, 121.53, 108.91, 102.13, 101.08, 99.18, 98.26, 51.62, 25.81, 18.93, 18.70, 11.91, 11.36; **HRMS** (ESI-TOF) [M+H] calculated for $[C_{34}H_{55}BrNSi_2]^+$ 612.3051, found 612.3064.

(11) **(*trans*)-1-(4-(4-fluorophenyl)-1,6-bis(triisopropylsilyl)hexa-3-en-1,5-diyn-3-yl)pyrrolidine 3k** (DYE 11)

(42.0 mg, 0.076 mmol, 76% yield), yellow oil. *trans*:*cis* ratio = 12.0:1. **^{1}H NMR** (*major isomer*) (500 MHz, $CDCl_3$) δ 7.55 (dd, *J* = 8.8, 5.6 Hz, 1H), 6.88 (dd, *J* = 8.8, 8.8 Hz, 1H), 3.73 – 3.70 (m, 4H), 1.89 – 1.87 (m, 4H), 1.08 – 1.07 (m, 18H, 3H), 0.96 – 0.95 (m, 18H, 3H); **^{13}C NMR** (*major isomer*) (126 MHz, $CDCl_3$) δ 161.37(d, *J* = 244.2 Hz), 136.71(d, *J* = 3.2 Hz), 136.27 , 131.47(d, *J* = 7.9 Hz), 114.19(d, *J* = 21.3 Hz), 109.40, 102.56, 100.47, 99.93, 98.19, 51.53, 25.80, 18.92, 18.64, 11.91, 11.37; **^{19}F NMR** (*major isomer*) (376 MHz, $CDCl_3$) δ -117.93; **HRMS** (ESI-TOF) [M+H] calculated for $[C_{34}H_{55}FNSi_2]^+$ 552.3852, found 552.3851.

(12) **(*trans*)-1-(4-(furan-2-yl)-1,6-bis(triisopropylsilyl)hexa-3-en-1,5-diyn-3-yl)pyrrolidine 3l** (DYE 12)

(38.1mg, 0.073mmol, 73% yield), yellow oil. *trans*:*cis* ratio = 12.8:1. **^{1}H NMR** (*major isomer*) (500 MHz, $CDCl_3$) δ 7.25 (dd, *J* = 1.9, 0.9 Hz, 1H), 6.40 (dd, *J* = 3.3, 0.9 Hz, 1H), 6.32 (dd, *J* = 3.3, 1.9 Hz, 1H), 3.73 – 3.70 (m, 4H), 1.88 – 1.85 (m, 4H), 1.09 – 1.08 (m, 18H, 3H, 3H), 1.06 (s, 18H); **^{13}C NMR** (*major isomer*) (126 MHz, $CDCl_3$) δ 153.71, 140.00, 135.53, 110.90, 106.95, 106.74, 102.06, 101.65, 97.49, 91.61, 51.71, 25.79, 18.92, 18.75, 11.88, 11.50; **HRMS** (ESI-TOF) [M+H] calculated for $[C_{32}H_{54}NOSi_2]^+$ 524.3738, found 524.3738.

(13) **(*trans*)-1-(4-(naphthalen-1-yl)-1,6-bis(triisopropylsilyl)hexa-3-en-1,5-diyn-3-yl)pyrrolidine 3m** (DYE 13)

(35.6 mg, 0.061 mmol, 61% yield), yellow oil. *trans*:*cis* ratio = 15.6:1. **^{1}H NMR** (*major isomer*) (400 MHz, $CDCl_3$) δ 8.26 (dd, *J* = 7.7, 1.8 Hz, 1H), 7.76 (dd, *J* = 7.2, 2.1 Hz, 1H), 7.67 (d, *J* = 8.3 Hz, 1H), 7.52 (dd, *J* = 7.1, 1.3 Hz, 1H), 7.42 – 7.32 (m, 3H), 3.86 – 3.83 (m, 4H), 1.95 – 1.92 (m, 4H), 0.97 – 0.94 (m, 18H, 3H), 0.68 – 0.65 (m, 18H, 3H); **^{13}C NMR** (*major isomer*) (101

MHz, $CDCl_3$) δ 139.05, 137.51, 134.14, 132.79, 128.85, 127.94, 126.96, 126.88, 125.55, 125.22, 125.15, 109.79, 102.27, 100.58, 97.65, 97.57, 51.41, 25.82, 18.82, 18.38, 11.77, 10.98; **HRMS** (ESI-TOF) [M+H] calculated for $[C_{38}H_{58}NSi_2]^+$ 584.4102, found 584.4100.

(14) **(*trans*)-1-(4-(naphthalen-2-yl)-1,6-bis(triisopropylsilyl)hexa-3-en-1,5-diyn-3-yl)pyrrolidine 3n** (DYE 14)

(51.4 mg, 0.088 mmol, 88% yield), yellow oil. *trans*:*cis* ratio = 8.9:1. **^{1}H NMR** (*major isomer*) (500 MHz, $CDCl_3$) δ 8.05 (d, *J* = 1.8 Hz, 1H), 7.81 (dd, *J* = 8.6, 1.8 Hz, 1H), 7.75 – 7.65 (m, 3H), 7.40 – 7.33 (m, 2H), 3.78 – 3.76 (m, 4H), 1.92 – 1.90 (m, 4H), 1.10 –1.09 (m, 18H, 3H), 0.87 – 0.85 (m, 18H, 3H); **^{13}C NMR** (*major isomer*) (126 MHz, $CDCl_3$) δ 138.15, 136.64, 133.50, 132.27, 128.67, 128.39, 128.00, 127.45, 126.67, 125.37, 124.98, 109.48, 102.77, 101.02, 100.31, 98.18, 51.64, 25.85, 18.97, 18.58, 11.95, 11.31; **HRMS** (ESI-TOF) [M+H] calculated for $[C_{38}H_{58}NSi_2]^+$ 584.4102, found 584.4107.

(15) **(*trans*)-1-(4-(3,4-dimethylphenyl)-1,6-bis(triisopropylsilyl)hexa-3-en-1,5-diyn-3-yl)pyrrolidine 3o** (DYE 15)

(45.5 mg, 0.081 mmol, 81% yield), yellow oil. *trans*:*cis* ratio = 8.6:1. **^{1}H NMR** (*major isomer*) (500 MHz, $CDCl_3$) δ 7.39 (d, *J* = 1.9 Hz, 1H), 7.36 (dd, *J* = 7.8, 1.9 Hz, 1H), 6.96 (d, *J* = 7.8 Hz, 1H), 3.71 – 3.68 (m, 4H), 2.20 (s, 3H), 2.19 (s, 3H), 1.88 – 1.86 (m, 4H), 1.08 – 1.07 (m, 18H, 3H), 0.96 – 0.96 (m, 18H, 3H); **^{13}C NMR** (*major isomer*) (126 MHz, $CDCl_3$) δ 137.88, 136.01, 135.06, 133.87, 131.24, 128.84, 127.26, 109.69, 102.94, 101.76, 99.96, 97.96, 51.53, 25.81, 18.95, 18.85, 18.72, 18.64, 11.95, 11.41; **HRMS** (ESI-TOF) [M+H] calculated for $[C_{36}H_{60}NSi_2]^+$ 562.4259, found 562.4261.

(16) **(*trans*)-1-(4-(4-fluoro-3-methylphenyl)-1,6-bis(triisopropylsilyl)hexa-3-en-1,5-diyn-3-yl)pyrrolidine 3p** (DYE 16)

(43.0 mg, 0.076 mmol, 76% yield), yellow oil. *trans*:*cis* ratio = 13:1. **^{1}H NMR** (*major isomer*) (500 MHz, $CDCl_3$) δ 7.41 – 7.36 (m, 2H), 6.82 (dd, *J* = 9.7, 8.3 Hz, 1H), 3.72 – 3.69 (m, 4H), 2.20 (d, *J* = 1.9 Hz, 3H), 1.89 – 1.86 (m, 4H), 1.08 – 1.07 (m, 18H, 3H), 0.96 – 0.94 (m, 18H, 3H); **^{13}C NMR** (*major isomer*) (126 MHz, $CDCl_3$) δ 160.00(d, *J* = 243.0 Hz), 136.33(d, *J* = 3.6 Hz), 136.10, 133.10(d, *J* = 5.0 Hz), 128.78(d, *J* = 7.8 Hz), 123.27(d, *J* = 17.4 Hz), 113.90(d, *J* = 22.4 Hz), 109.56, 102.66, 100.28(two overlaping carbons), 98.16, 51.52, 25.80, 18.92, 18.63, 14.66(d, *J* = 3.7 Hz), 11.92, 11.38; **^{19}F NMR** (*major isomer*) (376 MHz, $CDCl_3$) δ -122.22; **HRMS** (ESI-TOF) [M+H] calculated for $[C_{35}H_{57}FNSi_2]^+$ 566.4008, found 566.4016.

(17) **(*trans*)-1-(4-phenyl-6-(triisopropylsilyl)-1-(trimethylsilyl)hexa-3-en-1,5-diyn-3-yl)pyrrolidine 3q** (DYE 17)

(36.8 mg, 0.082 mmol, 82% yield), yellow oil. *trans*:*cis* ratio = 2.0:1. **^{1}H NMR** (*major isomer*) (400 MHz, $CDCl_3$) δ 7.68 – 7.62 (m, 2H), 7.24 – 7.20 (m, 2H), 7.14 – 7.10 (m, 1H), 3.71 – 3.68 (m, 4H), 1.89 – 1.86 (m, 4H), 1.08 – 1.06 (m, 18H, 3H), 0.07 (s, 9H); **^{13}C NMR** (*major isomer*) (101 MHz, $CDCl_3$) δ 140.03, 136.11, 129.64, 127.21, 125.64, 109.18, 103.35, 101.34, 101.03, 98.04, 51.44, 25.76, 18.92, 11.85, -0.44; **HRMS** (ESI-TOF) [M+H] calculated for $[C_{28}H_{44}NSi_2]^+$ 450.3007, found 450.3005.

(18) **(*trans*)-1-(4-phenyl-1-(triethylsilyl)-6-(triisopropylsilyl)hexa-3-en-1,5-diyn-3-yl)pyrrolidine 3r** (DYE 18)

(41.7 mg, 0.085 mmol, 85% yield), yellow oil. *trans*:*cis* ratio = 4.7:1. **^{1}H NMR** (*major isomer*) (400 MHz, $CDCl_3$) δ 7.67 – 7.64 (m, 2H), 7.24 – 7.19 (m, 2H), 7.12 – 7.08 (m, 1H), 3.74 – 3.70 (m, 4H), 1.90 – 1.87 (m, 4H), 1.11 – 1.09 (m, 18H, 3H), 0.88 (t, *J* = 7.9 Hz, 9H), 0.51 (q, *J* = 7.9 Hz, 6H); **^{13}C NMR** (*major isomer*) (101 MHz, $CDCl_3$) δ 140.18, 136.24, 129.72, 127.26, 125.63, 109.28, 101.99, 101.25, 97.98, 90.96, 51.47, 25.78, 18.92, 11.86, 7.49, 4.26; **HRMS** (ESI-TOF) [M+H] calculated for $[C_{31}H_{50}NSi_2]^+$ 492.3476, found 492.3476.

(19) **(*trans*)-1-(7,7-dimethyl-3-phenyl-1-(triisopropylsilyl)octa-3-en-1,5-diyn-4-yl)pyrrolidine 3s** (DYE 19)

(32.0 mg, 0.074 mmol, 74% yield), yellow oil. *trans*:*cis* ratio = 3.2:1. **^{1}H NMR** (*major isomer*) (400 MHz, $CDCl_3$) δ 7.69 – 7.61 (m, 2H), 7.23 – 7.19 (m, 2H), 7.11 – 7.06 (m, 1H), 3.71 – 3.68 (m, 4H), 1.88 – 1.85 (m, 4H), 1.11 (s, 9H), 1.09 – 1.08 (m, 18H, 3H); **^{13}C NMR** (*major isomer*) (101 MHz, $CDCl_3$) δ 140.63, 137.08, 129.57, 127.11, 125.20, 109.61, 106.31, 98.76, 96.46, 76.18, 51.41, 30.31, 28.33, 25.77, 18.94, 11.89; **HRMS** (ESI-TOF) [M+H] calculated for $[C_{29}H_{44}NSi]^+$ 434.3238, found 434.3237.

(20) **(*trans*)-1-(1-cyclohexyl-4-phenyl-6-(triisopropylsilyl)hexa-3-en-1,5-diyn-3-yl)pyrrolidine 3t** (DYE 20)

(34.0 mg, 0.074 mmol, 74% yield), yellow oil. *trans:cis* ratio = 2.2:1. **^{1}H NMR** (*major isomer*) (500 MHz, $CDCl_3$) δ 7.67 – 7.65 (m, 2H), 7.23 – 7.20 (m, 2H), 7.11 – 7.07 (m, 1H), 3.72 – 3.69 (m, 4H), 2.46 – 2.42 (m, 1H), 1.88 – 1.85 (m, 4H), 1.61 – 1.55 (m, 4H), 1.37 – 1.35 (m, 2H), 1.28 – 1.23 (m, 4H), 1.08 – 1.07 (m, 18H, 3H); **^{13}C NMR** (*major isomer*) (126 MHz, $CDCl_3$) δ 139.78, 136.19, 128.67, 126.19, 124.25, 108.81, 101.57, 98.17, 95.61, 76.57, 50.47, 31.01, 28.88, 25.03, 24.77, 23.72, 17.95, 10.96; **HRMS** (ESI-TOF) [M+H] calculated for $[C_{31}H_{46}NSi]^+$ 460.3394, found 460.3397.

(21) **(*trans*)-3-(4-phenyl-3-(pyrrolidin-1-yl)-6-(triisopropylsilyl)hexa-3-en-1,5-diyn-1-yl)pyridine 3u** (DYE 21)

(34.9 mg, 0.077 mmol, 77% yield), yellow oil. *trans:cis* ratio = 2.2:1. **^{1}H NMR** (*major isomer*) (400 MHz, $CDCl_3$) δ 8.46 (dd, *J* = 4.9, 2.0 Hz, 1H), 8.42 (d, *J* = 2.0 Hz, 1H), 7.69 – 7.66 (m, 2H), 7.45 – 7.38 (m, 2H), 7.30 – 7.26 (m, 2H), 7.19 – 7.17 (m, 1H), 3.80 – 3.76 (m, 4H), 1.94 – 1.91 (m, 4H), 1.09 – 1.07 (m, 18H, 3H); **^{13}C NMR** (*major isomer*) (101 MHz, $CDCl_3$) δ 151.88, 148.72, 140.30, 138.05, 135.70, 129.77, 127.45, 126.05, 123.10, 120.07, 108.98, 101.93, 98.66, 93.31, 89.94, 51.49, 25.78, 18.91, 11.84; **HRMS** (ESI-TOF) [M+H] calculated for $[C_{30}H_{39}N_2Si]^+$ 455.2877, found 455.2876.

(22) **(*trans*)-1-(1,4-diphenyl-6-(triisopropylsilyl)hexa-3-en-1,5-diyn-3-yl)pyrrolidine 3v** (DYE 22)

(38.5 mg, 0.085 mmol, 85% yield), yellow oil. *trans:cis* ratio = 2.8:1. **^{1}H NMR** (*major isomer*) (400 MHz, $CDCl_3$) δ 7.74 – 7.71 (m, 2H), 7.31 – 7.26 (m, 6H), 7.22 – 7.21 (m, 2H), 3.81 – 3.78 (m, 4H), 1.94 – 1.91 (m, 4H), 1.10 – 1.09 (m, 18H, 3H); **^{13}C NMR** (*major isomer*) (101 MHz, $CDCl_3$) δ 140.44, 136.49, 131.89, 131.32, 129.71, 128.40, 127.36, 125.68, 122.88, 109.35, 100.84, 97.93, 96.94, 86.67, 51.52, 25.81, 18.94, 11.88; **HRMS** (ESI-TOF) [M+H] calculated for $[C_{31}H_{40}NSi]^+$ 454.2925, found 454.2926.

(23) **(*trans*)-1-(1-(4-methoxyphenyl)-4-phenyl-6-(triisopropylsilyl)hexa-3-en-1,5-diyn-3-yl)pyrrolidine 3w** (DYE 23)

(36.7 mg, 0.076 mmol, 76% yield), yellow oil. *trans:cis* ratio = 2.5:1. **^{1}H NMR** (*major isomer*) (500 MHz, $CDCl_3$) δ 7.71 (d, *J* = 7.2 Hz, 2H), 7.28 – 7.25 (m, 2H), 7.18 – 7.11 (m, 3H), 6.78 (d, *J* = 7.2 Hz, 2H), 3.83 – 3.77 (m, 3H, 4H), 1.92 – 1.90 (m, 4H), 1.10 – 1.09 (m, 18H, 3H); **^{13}C NMR** (*major isomer*) (126 MHz, $CDCl_3$) δ 160.10, 140.69, 136.93, 132.88, 129.75, 127.33, 125.55, 115.15, 114.19, 109.63, 100.41, 97.63, 97.29, 85.58, 55.46, 51.51, 25.81, 18.96, 11.96; **HRMS** (ESI-TOF) [M+H] calculated for $[C_{32}H_{42}NOSi]^+$ 484.3030, found 484.3030.

(24) **(*trans*)-1-(1-(4-(tert-butyl)phenyl)-4-phenyl-6-(triisopropylsilyl)hexa-3-en-1,5-diyn-3-yl)pyrrolidine 3x** (DYE 24)

(44.7 mg, 0.088 mmol, 88% yield), yellow oil. *trans:cis* ratio = 2.7:1. **^{1}H NMR** (*major isomer*) (400 MHz, $CDCl_3$) δ 7.72 (d, *J* = 8.2 Hz, 2H), 7.39 – 7.34 (m, 2H), 7.30 – 7.27 (m, 3H), 7.16 (d, *J* = 8.2 Hz, 2H), 3.81 – 3.78 (m, 4H), 1.94 – 1.90 (m, 4H), 1.29 (s, 9H), 1.12 – 1.09 (s, 18H, 3H); **^{13}C NMR** (*major isomer*) (101 MHz, $CDCl_3$) δ 151.96, 140.49, 136.73, 131.11, 129.67, 127.33, 125.43, 119.86, 109.45, 100.47, 97.71, 97.24, 90.55, 86.04, 51.52, 34.94, 31.26, 25.81, 18.95, 11.89; **HRMS** (ESI-TOF) [M+H] calculated for $[C_{35}H_{48}NSi]^+$ 510.3551, found 510.3551.

(25) **(*trans*)-1-(4-(4-phenyl-3-(pyrrolidin-1-yl)-6-(triisopropylsilyl)hexa-3-en-1,5-diyn-1-yl)phenyl)pentan-1-one 3y** (DYE 25)

(33.3 mg, 0.062 mmol, 62% yield), yellow oil. *trans:cis* ratio = 2.8:1. **^{1}H NMR** (*major isomer*) (400 MHz, $CDCl_3$) δ 7.83 (d, *J* = 8.2 Hz, 2H), 7.70 – 7.68 (m, 2H), 7.30 – 7.27 (m, 2H), 7.24 (d, *J* = 8.2 Hz, 2H), 7.20 – 7.16 (m, 1H), 3.80 – 3.77 (m, 4H), 2.91 (t, *J* = 7.4 Hz, 2H), 1.95 – 1.91 (m, 4H), 1.73 – 1.65 (m, 2H), 1.45 – 1.36 (m, 2H), 1.10 – 1.07 (m, 18H, 3H), 0.94 (t, *J* = 7.4 Hz, 3H); **^{13}C NMR** (*major isomer*) (101 MHz, $CDCl_3$) δ 199.79, 140.34, 136.32, 135.92, 131.92, 131.33, 129.79, 128.08, 127.44, 125.98, 109.07, 102.08, 98.82, 96.05, 89.92, 51.51, 38.49, 26.59, 25.81, 22.59, 18.93, 14.06, 11.86; **HRMS** (ESI-TOF) [M+H] calculated for $[C_{36}H_{48}NOSi]^+$ 538.3500, found 538.3503.

(26) **(*trans*)-1-(9-chloro-3-phenyl-1-(triisopropylsilyl)nona-3-en-1,5-diyn-4-yl)pyrrolidine 3z** (DYE 26)

(24.9 mg, 0.055 mmol, 55% yield), yellow oil. *trans:cis* ratio = 2.5:1. **^{1}H NMR** (*major isomer*) (400 MHz, $CDCl_3$) δ 7.63 – 7.56 (m, 2H), 7.25 – 7.21 (m, 2H), 7.14 – 7.09 (m, 1H), 3.71 – 3.68 (m, 4H), 3.36 (t, *J* = 6.5 Hz, 2H), 2.42 (t, *J* = 6.5 Hz, 2H), 1.89 – 1.85 (m, 4H), 1.80 (tt, *J* = 6.5, 6.5 Hz, 2H), 1.08 – 1.06 (m, 18H, 3H); **^{13}C NMR** (*major isomer*) (101 MHz, $CDCl_3$) δ 140.71, 136.60, 129.66, 127.36, 125.52, 109.38, 99.53, 96.93, 96.12, 78.42, 51.46, 43.61, 30.87, 25.76, 18.93, 17.03, 11.87; **HRMS** (ESI-TOF) [M+H] calculated for $[C_{28}H_{41}ClNSi]^+$ 454.2691, found 454.2699.

(27) **(*R*,*trans*)-tert-butyl (1-(4-phenyl-1,6-bis(triisopropylsilyl)hexa-3-en-1,5-diyn-3-yl)pyrrolidin-3-yl)carbamate 3aa** (DYE 27)

(49.3 mg, 0.076 mmol, 76% yield), yellow oil. *trans:cis* ratio = 8.0:1. **^{1}H NMR** (*major isomer*) (400 MHz, $CDCl_3$) δ 7.62 – 7.60 (m, 2H), 7.23 – 7.19 (m, 2H), 7.13 – 7.09 (m, 1H), 4.72 – 4.70 (m, 1H), 4.24 (br, 1H), 3.92 – 3.84 (m, 2H), 3.66 – 3.54 (m, 2H), 2.18 – 2.10 (m, 1H), 1.87 – 1.85 (m, 1H), 1.45 (s, 9H), 1.08 – 1.06 (m, 18H, 3H), 0.95 – 0.94 (m, 18H, 3H); **^{13}C NMR** (*major isomer*) (101 MHz, $CDCl_3$) δ 155.40, 139.70, 136.06, 129.74, 127.52, 126.03, 108.62, 103.72, 102.01, 101.26, 99.36, 79.67, 57.11, 50.45, 49.30, 32.19, 28.53, 18.92, 18.65, 11.79, 11.29; **HRMS** [M+H] calculated for $[C_{39}H_{65}N_2O_2Si_2]^+$ 649.4579, found 649.4583.

(28) **(*R*,*trans*)-3-((tert-butyldiphenylsilyl)oxy)-1-(4-phenyl-1,6-bis(triisopropylsilyl)hexa-3-en-1,5-diyn-3-yl)pyrrolidine 3ab** (DYE 28)

(64.6 mg, 0.082 mmol, 82% yield), yellow oil. *trans*:*cis* ratio > 20:1. **^{1}H NMR** (400 MHz, $CDCl_3$) δ 7.72 – 7.64 (m, 6H), 7.45 – 7.37 (m, 6H), 7.23 (dd, *J* = 7.7, 7.7 Hz, 2H), 7.16 – 7.08 (m, 1H), 4.42 (tt, *J* = 4.4, 4.4 Hz, 1H), 4.03 – 3.97 (m, 1H), 3.85 – 3.81 (m, 1H), 3.71 – 3.64 (m, 2H), 1.94 (ddt, *J* = 11.8, 7.7, 4.2 Hz, 1H), 1.83 (ddt, *J* = 11.8, 7.7, 4.2 Hz, 1H), 1.11 – 1.09 (m, 18H, 3H, 9H), 0.97 – 0.96 (m, 18H, 3H); **^{13}C NMR** (101 MHz, $CDCl_3$) δ 140.05, 136.63, 135.84, 135.77, 135.73, 134.18, 133.89, 129.85, 129.76, 127.82, 127.81, 127.43, 125.68, 109.01, 102.21, 101.73, 100.62, 98.18, 72.20, 60.08, 49.41, 34.56, 26.99, 19.25, 18.96, 18.66, 11.83, 11.30; **HRMS** [M+H] calculated for $[C_{50}H_{74}NOSi_3]^+$ 788.5073, found 788.5072.

(29) **(*3S,4S*)-3,4-bis((tert-butyldimethylsilyl)oxy)-1-((*trans*)-4-phenyl-1,6-bis(triisopropylsilyl)hexa-3-en-1,5-diyn-3-yl)pyrrolidine 3ac** (DYE 29)

(51.6 mg, 0.065 mmol, 65% yield), yellow oil. *trans*:*cis* ratio > 20:1. **^{1}H NMR** (400 MHz, $CDCl_3$) δ 7.63 – 7.61 (m, 2H), 7.21 – 7.17 (m, 2H), 7.10 – 7.06 (m, 1H), 4.02 (t, *J* = 3.6 Hz, 2H), 3.94 (dd, *J* = 11.3, 3.6 Hz, 2H), 3.53 (dd, *J* = 11.0, 3.6 Hz, 2H), 1.13 – 1.08 (m, 21H), 0.95 (s, 18H), 0.89 – 0.84 (m, 21H), 0.08 (s, 6H), 0.07 (s, 6H); **^{13}C NMR** (101 MHz, $CDCl_3$) δ 140.12, 137.06, 129.79, 127.41, 125.67, 109.02, 102.16, 101.45, 100.74, 98.00, 76.26, 57.50, 25.91, 19.00, 18.66, 18.10, 11.84, 11.31, -4.54, -4.63; **HRMS** [M+H] calculated for $[C_{46}H_{84}NO_2Si_4]^+$ 794.5574, found 794.5586.

(30) **(*trans*)-1-(4-phenyl-1,6-bis(triisopropylsilyl)hexa-3-en-1,5-diyn-3-yl)azetidine 3ad** (DYE 30)

(38.9 mg, 0.075 mmol, 75% yield), yellow oil. *trans:cis* ratio = 4.2:1. **^{1}H NMR** (*major isomer*) (400 MHz, $CDCl_3$) δ 7.70 – 7.68 (m, 2H), 7.23 – 7.19 (m, 2H), 7.13 – 7.09 (m, 1H), 4.23 (t, *J* = 7.5 Hz, 4H), 2.24 (p, *J* = 7.5 Hz, 2H), 1.10 – 1.07 (m, 18H, 3H), 1.01 – 1.00 (m, 18H, 3H); **^{13}C NMR** (*major isomer*) (126 MHz, $CDCl_3$) δ 139.19, 138.71, 129.07, 127.48, 125.83, 107.37, 102.05, 101.56, 100.43, 99.04, 54.54, 18.91, 18.67, 16.77, 11.86, 11.39; **HRMS** (ESI-TOF) [M+H] calculated for $[C_{33}H_{54}NSi_2]^+$ 520.3789, found 520.3786.

Analytical data of α,α-difunctionalized Ynones:

(31) **(*R*)-4-fluoro-4-phenyl-1,6-bis(triisopropylsilyl)hexa-1,5-diyn-3-one 4a** (DFY 1)

(38. 0 mg, 0.076 mmol, 76% yield, 92% ee), yellow oil, $[\alpha]_D^{25}$=8.1 (c=1.00, $CHCl_3$). **^{1}H NMR** (400 MHz, $CDCl_3$) δ 7.68 – 7.65 (m, 2H), 7.41 – 7.39 (m, 3H), 1.17 – 1.11 (m, 18H, 3H), 1.00 – 0.95 (m, 18H, 3H); **^{13}C NMR** (101 MHz, $CDCl_3$) δ 177.40 (d, *J* = 32.3 Hz), 135.00 (d, *J* = 24.2 Hz), 129.96 (d, *J* = 2.0 Hz), 128.70, 127.00 (d, *J* = 4.0 Hz), 103.97, 100.30, 99.27 (d, *J* = 27.3 Hz), 96.61 (d, *J* = 7.1 Hz), 94.34 (d, *J* = 189.9 Hz), 18.71, 18.49, 11.27, 11.03; **^{19}F NMR** (376 MHz, $CDCl_3$) δ -136.00; **HRMS** (ESI-TOF) [M+Na] calculated for $[C_{30}H_{47}FNaOSi_2]^+$ 521.3042, found 521.3041. **HPLC** analysis (Chiralpak IA column, Ethanol/hexane = 0/100, 1.0 mL/min, λ = 254nm): t_R(minor) = 5.01 minutes, t_R(major) = 5.44 minutes.

(32) **(*R*)-4-fluoro-4-(m-tolyl)-1,6-bis(triisopropylsilyl)hexa-1,5-diyn-3-one 4b** (DFY 2)

(39. 5 mg, 0.077 mmol, 77% yield, 92% ee), yellow oil, **^{1}H NMR** (400 MHz, $CDCl_3$) δ 7.50 (s, 1H), 7.44 (d, *J* = 7.6 Hz, 1H), 7.29 (dd, *J* = 7.6, 7.6 Hz, 1H), 7.20 (d, *J* = 7.6 Hz, 1H), 2.36 (s, 3H), 1.13 – 1.12 (m, 18H, 3H), 1.00 – 0.99 (m, 18H, 3H); **^{13}C NMR** (101 MHz, $CDCl_3$) δ 177.50(d, *J* = 32.0 Hz), 138.47, 134.85(d, *J* = 24.2 Hz), 130.71(d, *J* = 2.1 Hz), 128.64, 127.76(d, *J* = 4.1 Hz), 124.10(d, *J* = 4.1 Hz), 103.80, 100.35, 99.44, 96.51(d, *J* = 7.1 Hz), 94.42(d, *J* = 188.6 Hz), 21.50, 18.72, 18.49(d, *J* = 1.5 Hz), 11.27, 11.02; **^{19}F NMR** (376 MHz, $CDCl_3$) δ -135.96; **HRMS** (ESI-TOF) [M+Na] calculated for $[C_{31}H_{49}FNaOSi_2]^+$ 535.3198, found 535.3196. **HPLC** analysis (Chiralpak IA column, Ethanol/hexane = 0/100, 1.0 mL/min, λ = 254nm): t_R(minor) = 5.01 minutes, t_R(major) = 5.42 minutes.

(33) **(*R*)-4-fluoro-4-(p-tolyl)-1,6-bis(triisopropylsilyl)hexa-1,5-diyn-3-one 4c** (DFY 3)

(41. 0 mg, 0.080 mmol, 80% yield, 91% ee), yellow oil, **^{1}H NMR** (400 MHz, $CDCl_3$) δ 7.53 (d, *J* = 7.5 Hz, 2H), 7.21 (d, *J* = 7.9 Hz, 2H), 2.36 (s, 3H), 1.12 – 1.11 (m, 18H, 3H), 1.00 – 0.98 (m, 18H, 3H); **^{13}C NMR** (101 MHz, $CDCl_3$) δ 177.62(d, *J* = 32.5 Hz), 140.07(d, *J* = 2.3 Hz), 132.11(d, *J* = 24.7 Hz), 129.39, 127.00(d, *J* = 4.0 Hz), 103.75, 100.39, 99.38(d, *J* = 27.2 Hz), 96.36(d, *J* = 7.0 Hz), 94.30(d, *J* = 188.3 Hz), 21.35, 18.73, 18.49(d, *J* = 1.2 Hz), 11.27, 11.04; **^{19}F NMR** (376 MHz, $CDCl_3$) δ -135.08; **HRMS** (ESI-TOF) [M+Na] calculated for $[C_{31}H_{49}FNaOSi_2]^+$ 535.3198, found 535.3200. **HPLC** analysis (Chiralpak IA column, Ethanol/hexane = 0/100, 1.0 mL/min, λ = 254nm): t_R(minor) = 5.42 minutes, t_R(major) = 6.64 minutes.

(34) **(*R*)-4-(4-(tert-butyl)phenyl)-4-fluoro-1,6-bis(triisopropylsilyl)hexa-1,5-diyn-3-one 4d** (DFY 4)

(39. 9 mg, 0.072 mmol, 72% yield, 95% ee), yellow oil, **^{1}H NMR** (400 MHz, $CDCl_3$) δ 7.58 (d, *J* = 7.7 Hz, 2H), 7.42 (d, *J* = 8.2 Hz, 2H), 1.31 (s, 9H), 1.14 – 1.06 (m, 18H, 3H), 0.99 – 0.95 (m, 18H, 3H); **^{13}C NMR** (101 MHz, $CDCl_3$) δ 177.44 (d, *J* = 32.3 Hz), 153.23, 131.91(d, *J* = 23.9 Hz), 126.93(d, *J* = 3.7 Hz), 125.74, 103.70, 100.43, 99.17(d, *J* = 27.3 Hz), 96.59(d, *J* = 6.8 Hz), 94.32(d, *J* = 188.6 Hz), 34.88, 31.36, 18.74, 18.51, 11.28, 11.03; **^{19}F NMR** (376 MHz, $CDCl_3$) δ -134.71; **HRMS** (ESI-TOF) [M+Na] calculated for $[C_{34}H_{55}FNaOSi_2]^+$ 577.3668, found 577.3664. **HPLC** analysis (Chiralpak IA column, Ethanol/hexane = 0/100, 1.0 mL/min, λ = 254nm): t_R(minor) = 5.06 minutes, t_R(major) = 5.91 minutes.

(35) **(*R*)-4-fluoro-4-(3-fluorophenyl)-1,6-bis(triisopropylsilyl)hexa-1,5-diyn-3-one 4e** (DFY 5)

(31. 5 mg, 0.061 mmol, 61% yield, 94% ee), yellow oil, **^{1}H NMR** (400 MHz, $CDCl_3$) δ 7.43 – 7.37 (m, 3H), 7.13 – 7.08 (m, 1H), 1.12 – 1.11 (m, 18H, 3H), 1.01 – 1.00 (m, 18H, 3H); **^{13}C NMR** (126 MHz, $CDCl_3$) δ 176.79(d, *J* = 32.7 Hz), 162.84(d, *J* = 247.3 Hz), 137.48(dd, *J*=24.0 Hz, 7.6 Hz), 130.35(d, *J* = 8.0 Hz), 122.57, 116.93(d, *J* = 21.1 Hz), 114.48(dd, *J* = 24.0, 4.7 Hz), 104.60, 100.12, 98.96(d, *J* = 26.9 Hz), 97.02(d, *J*=6.3 Hz), 93.62(d, *J* = 190.2 Hz), 18.70, 18.50, 11.30, 11.10; **^{19}F NMR** (376 MHz, $CDCl_3$) δ -111.89, -137.17; **HRMS** (ESI-TOF) [M+Na] calculated for $[C_{30}H_{46}F_2NaOSi_2]^+$ 539.2947, found 539.2949. **HPLC** analysis (Chiralpak IA column, Ethanol/hexane = 0/100, 1.0 mL/min, λ = 254nm): t_R(minor) = 4.50 minutes, t_R(major) = 4.88 minutes.

(36) **(*R*)-4-fluoro-4-(4-fluorophenyl)-1,6-bis(triisopropylsilyl)hexa-1,5-diyn-3-one 4f** (DFY 6)

(32. 0 mg, 0.062 mmol, 62% yield, 90% ee), yellow oil, ^{1}**H NMR** (400 MHz, $CDCl_3$) δ 7.64 (dd, *J* = 8.5, 5.2, 2H), 7.10 (dd, *J* = 8.5, 8.5 Hz, 2H), 1.12 – 1.11 (m, 18H, 3H), 1.01 – 1.00 (m, 18H, 3H); ^{13}**C NMR** (101 MHz, $CDCl_3$) δ 177.15(d, *J* = 32.7 Hz), 163.79(dd, *J* = 249.7, 2.4 Hz), 131.05(dd, *J* = 24.9, 3.2 Hz), 129.11(dd, *J* = 8.7, 4.1 Hz), 115.77(d, *J* = 22.1 Hz), 104.37(d, *J* = 1.5 Hz), 100.18, 99.01(d, *J* = 27.0 Hz), 97.02(d, *J* = 7.0 Hz), 93.72(d, *J* = 189.1 Hz), 18.71, 18.49, 11.24, 11.03; ^{19}**F NMR** (376 MHz, $CDCl_3$) δ -111.27, -134.87; **HRMS** (ESI-TOF) [M+Na] calculated for $[C_{30}H_{46}F_2NaOSi_2]^+$ 539.2947, found 539.2943. **HPLC** analysis (Chiralpak IA column, Ethanol/hexane = 0/100, 1.0 mL/min, λ = 254nm): t_R(minor) = 4.59 minutes, t_R(major) = 4.98 minutes.

(37) **(*R*)-4-(3-chlorophenyl)-4-fluoro-1,6-bis(triisopropylsilyl)hexa-1,5-diyn-3-one 4g** (DFY 7)

(32. 0 mg, 0.060 mmol, 60% yield, 93% ee), yellow oil, ^{1}**H NMR** (400 MHz, $CDCl_3$) δ 7.72 (dd, *J* = 2.0, 2.0 Hz, 1H), 7.50 (ddt, *J* = 7.5, 2.0, 2.0 Hz, 1H), 7.40 – 7.32 (m, 2H), 1.13 – 1.12 (m, 18H, 3H), 1.02 – 1.01 (m, 18H, 3H); ^{13}**C NMR** (101 MHz, $CDCl_3$) δ 176.69(d, *J* = 32.1 Hz), 137.01(d, *J* = 24.7 Hz), 134.83, 130.10(d, *J* = 1.7 Hz), 130.00, 127.50(d, *J* = 4.6 Hz), 124.93(d, *J* = 4.2 Hz), 104.84, 99.99, 98.65(d, *J* = 26.8 Hz), 97.34(d, *J* = 6.9 Hz), 93.63(d, *J* = 190.2 Hz), 18.69, 18.49, 11.23, 11.02; ^{19}**F NMR** (376 MHz, $CDCl_3$) δ -137.67; **HRMS** (ESI-TOF) [M+Na] calculated for $[C_{30}H_{46}ClFNaOSi_2]^+$ 555.2652, found 555.2652. **HPLC** analysis (Chiralpak IA column, Ethanol/hexane = 0/100, 1.0 mL/min, λ = 254nm): t_R(minor) = 4.68 minutes, t_R(major) = 5.11 minutes.

(38) **(*R*)-4-(3-bromophenyl)-4-fluoro-1,6-bis(triisopropylsilyl)hexa-1,5-diyn-3-one 4h** (DFY 8)

(37. 0 mg, 0.064 mmol, 64% yield, 92% ee), yellow oil, ^{1}**H NMR** (400 MHz, $CDCl_3$) δ 7.89 (d, *J* = 1.6 Hz, 1H), 7.55 (d, *J* = 1.6 Hz, 1H), 7.53 (d, *J* = 1.6 Hz, 1H), 7.29 (dd, *J* = 7.9, 7.9 Hz, 1H), 1.13 – 1.12 (m, 18H, 3H), 1.02 – 1.01 (m, 18H, 3H); ^{13}**C NMR** (126 MHz, $CDCl_3$) δ 176.73(d, *J*=32.8 Hz), 133.05, 130.42(d, *J* = 4.7 Hz), 130.25, 129.02, 125.42(d, *J* = 4.2 Hz), 122.87, 104.84, 100.14, 98.86(d, *J* = 27.2 Hz), 97.40(d, *J* = 7.1 Hz), 93.56(d, *J* = 190.3 Hz), 18.72, 18.54, 11.32, 11.12; ^{19}**F NMR** (376 MHz, $CDCl_3$) δ -137.61; **HRMS** (ESI-TOF) [M+Na] calculated for $[C_{30}H_{46}BrFNaOSi_2]^+$ 599.2147, found 599.2148. **HPLC** analysis (Chiralpak IA column, Ethanol/hexane = 0/100, 1.0 mL/min, λ = 254nm): t_R(minor) = 4.86 minutes, t_R(major) = 5.24 minutes.

(39) **(*R*)-4-fluoro-4-(3-methoxyphenyl)-1,6-bis(triisopropylsilyl)hexa-1,5-diyn-3-one 4i** (DFY 9)

(31.7 mg, 0.060 mmol, 60% yield, 90% ee), yellow oil, ^{1}**H NMR** (400 MHz, $CDCl_3$) δ 7.31 (dd, *J* = 8.0, 8.0 Hz, 1H), 7.27 – 7.19 (m, 2H), 6.93 (ddd, *J* = 8.0, 1.5, 1.5 Hz, 1H), 3.80 (s, 3H), 1.13 – 1.12 (m, 18H, 3H), 1.01– 0.99 (m, 18H, 3H); ^{13}**C NMR** (126 MHz, $CDCl_3$) δ 177.28(d, *J* = 32.4 Hz), 159.97, 136.45(d, *J* = 24.3 Hz), 129.76, 119.40(d, *J* = 4.4 Hz), 116.28(d, *J* = 2.3 Hz), 112.15(d, *J* = 4.7 Hz), 103.87, 100.39, 99.39(d, *J* = 27.1 Hz), 96.48(d, *J* = 7.0 Hz), 94.24(d, *J* = 188.9 Hz), 55.44, 18.73, 18.51, 11.32, 11.10; ^{19}**F NMR** (376 MHz, $CDCl_3$) δ -135.74; **HRMS** (ESI-TOF) [M+Na] calculated for $[C_{31}H_{49}FNaO_2Si_2]^+$ 551.3147, found 551.3146. **HPLC** analysis (Chiralpak IA column, Ethanol/hexane = 0/100, 1.0 mL/min, λ = 254nm): t_R(minor) = 6.70 minutes, t_R(major) = 7.26 minutes.

(40) **(*R*)-4-fluoro-4-(naphthalen-2-yl)-1,6-bis(triisopropylsilyl)hexa-1,5-diyn-3-one 4j** (DFY 10)

(41.2 mg, 0.075 mmol, 75% yield, 94% ee), yellow oil, ^{1}**H NMR** (500 MHz, $CDCl_3$) δ 8.27 (s, 1H), 7.88 – 7.83 (m, 3H), 7.64 (dd, *J* = 8.6, 1.9 Hz, 1H), 7.54 – 7.52 (m, 2H), 1.18 – 1.14 (m, 18H, 3H), 0.92 – 0.90 (m, 18H, 3H); ^{13}**C NMR** (126 MHz, $CDCl_3$) δ 177.44(d, *J* = 32.4 Hz), 134.05, 133.03, 132.50(d, *J* = 24.5 Hz), 128.72, 127.84, 127.50(d, *J* = 5.4 Hz), 127.29, 126.74, 126.10(d, *J* = 8.0 Hz), 123.54(d, *J* = 3.6 Hz), 104.20, 100.50, 99.54(d, *J* = 27.0 Hz), 96.85(d, *J* = 7.2 Hz), 94.59(d, *J* = 188.8 Hz), 18.78, 18.43, 11.39, 11.07; ^{19}**F NMR** (376 MHz, $CDCl_3$) δ -136.48; **HRMS** (ESI-TOF) [M+Na] calculated for $[C_{34}H_{49}FNaOSi_2]^+$ 571.3198, found 571.3195. **HPLC** analysis (Chiralpak IA column, Ethanol/hexane = 0/100, 1.0 mL/min, λ = 254nm): t_R(minor) = 6.64 minutes, t_R(major) = 7.81 minutes.

(41) **(*R*)-4-fluoro-4-(4-fluoro-3-methylphenyl)-1,6-bis(triisopropylsilyl)hexa-1,5-diyn-3-one 4k** (DFY 11)

(30.8 mg, 0.058 mmol, 58% yield, 90% ee), yellow oil, **^{1}H NMR** (400 MHz, $CDCl_3$) δ 7.52 – 7.50 (m, 1H), 7.46 – 7.42 (m, 1H), 7.05 – 7.00 (m, 1H), 2.27 (d, *J* = 2.5 Hz, 3H), 1.13 – 1.12 (m, 18H, 3H), 1.01 – 1.00 (m, 18H, 3H); **^{13}C NMR** (101 MHz, $CDCl_3$) δ 177.26(d, *J* = 32.8 Hz), 162.36(d, *J* = 249.1 Hz), 130.76(d, *J* = 3.2 Hz), 130.63 – 130.46 (m), 126.35(dd, *J* = 8.8, 4.2 Hz), 125.42(d, *J* = 18.0 Hz), 115.38(d, *J* = 23.2 Hz), 104.12, 100.31, 99.16(d, *J* = 27.1 Hz), 96.87(d, *J* = 7.0 Hz), 93.83(d, *J* = 189.1 Hz), 18.71, 18.48(d, *J* = 1.7 Hz), 14.71(d, *J* = 3.4 Hz), 11.27, 11.04; **^{19}F NMR** (376 MHz, $CDCl_3$) δ -115.58, -134.86; **HRMS** (ESI-TOF) [M+Na] calculated for $[C_{31}H_{48}F_2NaOSi_2]^+$ 553.3104, found 553.3100. **HPLC** analysis (Chiralpak IA column, Ethanol/hexane = 0/100, 1.0 mL/min, λ = 254nm): t_R(minor) = 4.47 minutes, t_R(major) = 4.79 minutes.

(42) **(*R*)-4-(3,4-dimethylphenyl)-4-fluoro-1,6-bis(triisopropylsilyl)hexa-1,5-diyn-3-one 4l** (DFY 12)

(35.8 mg, 0.068 mmol, 68% yield, 90% ee), yellow oil, **^{1}H NMR** (400 MHz, $CDCl_3$) δ 7.45 (d, *J* = 2.0 Hz, 1H), 7.36 (dd, *J* = 7.9, 2.0 Hz, 1H), 7.16 (d, *J* = 7.9 Hz, 1H), 2.26 (s, 6H), 1.13 – 1.12 (m, 18H, 3H), 1.00 – 0.99 (m, 18H, 3H); **^{13}C NMR** (101 MHz, $CDCl_3$) δ 177.69(d, *J* = 32.5 Hz), 138.71(d, *J* = 2.4 Hz), 136.98, 129.97, 129.36, 128.32(d, *J* = 3.9 Hz), 124.49(d, *J* = 3.9 Hz), 103.57, 100.49, 99.49(d, *J* = 26.9 Hz), 96.26(d, *J* = 6.8 Hz), 94.35 (d, *J* = 188.2 Hz), 19.93, 19.67, 18.73, 18.46(d, *J* = 1.6 Hz), 11.29, 11.05; **^{19}F NMR** (376 MHz, $CDCl_3$) δ -135.16; **HRMS** (ESI-TOF) [M+Na] calculated for $[C_{32}H_{51}FNaOSi_2]^+$ 549.3355, found 549.3355. **HPLC** analysis (Chiralpak IA column, Ethanol/hexane = 0/100, 1.0 mL/min, λ = 254nm): t_R(minor) = 5.37 minutes, t_R(major) = 6.38 minutes.

(43) **(*R*)-1-(4-(tert-butyl)phenyl)-4-fluoro-4-phenyl-6-(triisopropylsilyl)hexa-1,5-diyn-3-one 4m** (DFY 13)

(33.6 mg, 0.071 mmol, 71% yield, 41% ee), yellow oil, **^{1}H NMR** (400 MHz, $CDCl_3$) δ 7.75 – 7.72 (m, 2H), 7.45 – 7.41 (m, 5H), 7.39 – 7.37 (m, 2H), 1.31 (s, 9H), 1.14 – 1.10 (m, 18H, 3H); **^{13}C NMR** (101 MHz, $CDCl_3$) δ 178.26(d, *J* = 32.2 Hz), 155.30, 135.20 (d, *J* = 24.6 Hz), 133.29, 129.95(d, *J* = 2.0 Hz), 128.76, 126.83(d, *J* = 4.5 Hz), 125.81, 116.45, 99.79(d, *J* = 27.0 Hz), 98.40, 96.35(d, *J* = 6.9 Hz), 94.32(d, *J* = 188.5 Hz), 84.74, 35.29, 31.15, 18.72, 11.27; **^{19}F NMR** (376 MHz, $CDCl_3$) δ -137.64; **HRMS** (ESI-TOF) [M+Na] calculated for $[C31H39FNaOSi]^+$ 497.2646, found 497.2642. **HPLC** analysis (Chiralpak OD column, Ethanol/hexane = 0/100, 1.0 mL/min, λ = 254nm): t_R(minor) = 14.77 minutes, t_R(major) = 16.86 minutes.

(44) **(*R*)-1-cyclohexyl-4-fluoro-4-phenyl-6-(triisopropylsilyl)hexa-1,5-diyn-3-one 4n** (DFY 14)

(27.1 mg, 0.064 mmol, 64% yield, 29% ee), yellow oil, **^{1}H NMR** (400 MHz, $CDCl_3$) δ 7.71 – 7.62 (m, 2H), 7.45 – 7.38 (m, 3H), 2.55 – 2.49 (m, 1H), 1.75 – 1.68 (m, 2H), 1.63 – 1.54 (m, 2H), 1.47 – 1.40 (m, 2H), 1.35 – 1.24 (m, 4H), 1.19 – 1.11 (m, 18H, 3H); **^{13}C NMR** (101 MHz, $CDCl_3$) δ 178.22(d, *J* = 31.8 Hz), 135.23(d, *J* = 24.5 Hz), 129.83(d, *J* = 2.1 Hz), 128.62 , 126.84(d, *J* = 4.4 Hz), 105.13(two overlaping carbons), 99.81(d, *J* = 27.0 Hz), 95.98(d, *J* = 7.0 Hz), 94.35(d, *J* = 188.1 Hz), 31.23(d, *J* = 2.8 Hz), 29.43, 25.68, 24.47(d, *J* = 4.3 Hz), 18.71, 11.28; **^{19}F NMR** (376 MHz, $CDCl_3$) δ -137.40; **HRMS** (ESI-TOF) [M+Na] calculated for $[C27H37FNaOSi]^+$ 447.2490, found 447.2491. **HPLC** analysis (Chiralpak IA column, Ethanol/hexane = 0/100, 1.0 mL/min, λ = 254nm): t_R(minor) = 9.45 minutes, t_R(major) = 11.18 minutes.

Analytical data of compounds 8-10:

(45) **(*R*)-4-(1-fluoro-1-phenyl-3-(triisopropylsilyl)prop-2-yn-1-yl)-2-phenylpyrimidine 8** (DFY 15)

(40.0 mg, 0.090 mmol, 90% yield), yellow oil, **^{1}H NMR** (400 MHz, $CDCl_3$) δ 8.84 (d, *J* = 5.1 Hz, 1H), 8.45 – 8.43 (m, 2H), 7.75 – 7.72 (m, 2H), 7.56 (dd, *J* = 5.1, 1.3 Hz, 1H), 7.47 – 7.44 (m, 3H), 7.40 – 7.34 (m, 3H), 1.13 – 1.09 (m, 18H, 3H); **^{13}C NMR** (101 MHz, $CDCl_3$) δ 168.35(d, *J* = 32.1 Hz), 164.35(d, *J* = 2.4 Hz), 158.43, 139.50(d, *J* = 24.5 Hz), 137.25, 131.02, 129.11, 129.09, 128.53, 128.44, 126.27(d, *J* = 5.4 Hz), 113.85(d, *J* = 6.3 Hz), 103.54(d, *J* = 26.2 Hz), 93.56(d, *J* = 7.5 Hz), 92.50(d, *J* = 175.2 Hz), 18.72, 11.29; **^{19}F NMR** (376 MHz, $CDCl_3$) δ -136.99; **HRMS** (ESI-TOF) [M+H] calculated for $[C_{28}H_{34}FN_2Si]^+$ 445.2470, found 445.2480.

(46) **(*R*)-4-((1-benzyl-1H-1,2,3-triazol-4-yl)fluoro(phenyl)methyl)-2-phenylpyrimidine 9** (DFY 16)

(31.1 mg, 0.070 mmol, 78% yield, 92% ee), yellow oil, **^{1}H NMR** (400 MHz, $CDCl_3$) δ 8.86 (d, *J* = 5.2 Hz, 1H), 8.31 – 8.28 (m, 2H), 7.66 – 7.64 (m, 3H), 7.48 – 7.42 (m, 4H), 7.37 – 7.34 (m, 6H), 7.26 – 7.23 (m, 2H), 5.54 (s, 2H); **^{13}C NMR** (101 MHz, $CDCl_3$) δ 168.80(d, *J* = 31.4 Hz), 163.92(d, *J* = 2.6 Hz), 158.53(d, *J* = 1.9 Hz), 149.17(d, *J* = 24.6 Hz), 139.72(d, *J* = 23.9 Hz), 137.38, 134.54, 130.99, 129.28, 128.92, 128.79(d, *J* = 1.5 Hz), 128.66, 128.39, 128.35, 128.07, 126.28(d, *J* = 8.1 Hz), 124.05(d, *J* = 4.7 Hz), 115.08(d, *J* = 8.4 Hz), 94.34(d, *J* = 176.8 Hz), 54.34; **^{19}F NMR** (376 MHz, $CDCl_3$) δ -143.60; **HRMS** (ESI-TOF) [M+Na] calculated for $[C_{26}H_{20}FN_5Na]^+$ 444.1595, found 444.1596. **HPLC** analysis (Chiralpak OD-H column, Ethanol/hexane = 10/90, 1.0 mL/min, λ = 254nm): t_R(major) = 15.69 minutes, t_R(minor) = 19.55 minutes.

(47) **(*R*)-3-(fluoro(phenyl)(2-phenylpyrimidin-4-yl)methyl)isoquinolin-1(2H)-one 10** (DFY 17)

(26.5 mg, 0.065 mmol, 72% yield, 94% ee), yellow oil, **^{1}H NMR** (400 MHz, $CDCl_3$) δ 9.69 (br, 1H), 8.98 (d, *J* = 5.1 Hz, 1H), 8.40 – 8.37 (m, 3H), 7.72 (dd, *J* = 5.1, 2.3 Hz, 1H), 7.69 – 7.65 (m, 1H), 7.56 – 7.52 (m, 2H), 7.50 – 7.47 (m, 3H), 7.47 – 7.44 (m, 2H), 7.41 – 7.39 (m, 3H), 6.77 (s, 1H); **^{13}C NMR** (101 MHz, $CDCl_3$) δ 166.97(d, *J* = 31.1 Hz), 164.13(d, *J* = 2.9 Hz), 162.60, 159.27 (d, *J* = 2.3 Hz), 138.27(d, *J* = 26.5 Hz), 137.91(d, *J* = 21.8 Hz), 136.87(d, *J* = 34.6 Hz), 133.08, 131.53, 130.13, 129.96(d, *J* = 2.7 Hz), 128.97, 128.90, 128.49, 128.37, 127.75(d, *J* = 4.6 Hz), 127.62(d, *J* = 6.3 Hz), 127.19, 125.82, 115.22(d, *J* = 9.9 Hz), 105.51(d, *J* = 9.0 Hz), 95.77(d, *J* = 180.6 Hz); **^{19}F NMR** (376 MHz, $CDCl_3$) δ -148.12; **HRMS** (ESI-TOF) [M+H] calculated for $[C_{26}H_{19}FN_3O]^+$ 408.1507, found 408.1495. **HPLC** analysis (Chiralpak IB column, Ethanol/hexane = 1.5/98.5, 1.0 mL/min, λ = 254nm): t_R(minor) = 20.94 minutes, t_R(major) = 22.48 minutes.

Analytical data of compounds 7:

(48) **(3*S*,4*R*)-4-fluoro-4-phenyl-1,6-bis(triisopropylsilyl)hexa-1,5-diyn-3-yl 3,5-dinitrobenzoate 7** (DFY 18)

(32.0 mg, 0.046 mmol, 23% yield), white solid, **^{1}H NMR** (400 MHz, $CDCl_3$) δ 9.26 (dd, *J* = 2.2, 2.2 Hz, 1H), 9.20 (d, *J* = 2.2 Hz, 2H), 7.78 – 7.75 (m, 2H), 7.45 – 7.42 (m, 3H), 6.17 (d, *J* = 8.2 Hz, 1H), 1.27 – 1.13 (m, 18H, 3H), 1.12 – 0.96 (m, 18H, 3H); **^{13}C NMR** (101 MHz, $CDCl_3$) δ 161.37 , 148.87 , 135.89 (d, *J* = 23.3 Hz), 133.42 , 129.90 (d, *J* = 1.9 Hz), 129.77 , 128.42 , 126.96 (d, *J* = 5.1 Hz), 122.89 , 100.34 (d, *J* = 26.1 Hz), 97.77 (d, *J* = 8.1 Hz), 94.25 (d, *J* = 7.4 Hz), 93.05 (d, *J* = 126.4 Hz), 91.88 , 71.69 (d, *J* = 35.0 Hz), 18.72 (d, *J* = 2.1 Hz), 18.56 , 11.23 , 11.07; **^{19}F NMR** (376 MHz, $CDCl_3$) δ -137.62; **HRMS** (ESI-TOF) [M+Na] calculated for $[C_{37}H_{51}FN_2NaO_6Si_2]^+$ 717.3162, found 717.3164.

Supplementary Notes

Supplementary Note 1

All DFT calculations were carried out with Gaussian 09. Geometry optimizations and frequency calculations were performed at B3LYP/6-31G(d) level. Solvent effects were taken into account by using the SMD in *m*-xylene. Single-point calculations with solvent effects were performed with B3LYP-D3(BJ)/6-311+G(d,p). All of the energies shown in this article were calculated at 1 atm and 243.15 K.

Supplementary Note 2

Cartesian coordinates of ***R*-3ab-R1**

C	-2.98356800	3.05096700	-3.06006300
C	-2.36353600	2.09987700	-2.24916300
C	-3.03758800	1.53651000	-1.15267700
C	-4.36192100	1.94071000	-0.90851300
C	-4.98249900	2.89054400	-1.71911200
C	-4.29575200	3.45213900	-2.79837300
H	-2.44522800	3.46591900	-3.90858100
H	-1.35185900	1.77546200	-2.47300100
H	-4.89963700	1.50577600	-0.07108400
H	-6.00452100	3.19358200	-1.50620500
H	-4.78108400	4.18807000	-3.43393100
C	-2.39317800	0.49526000	-0.29838400
C	-1.12253900	0.68594600	0.24517700
C	-0.57815300	2.01211800	0.27421700
C	-3.18925900	-0.64193000	-0.01923200
C	-0.13210300	3.14679200	0.37103500
C	-3.96332800	-1.56669400	0.21230800
Si	0.45701200	4.89325100	0.49136400
Si	-5.14436100	-2.92271800	0.57792400
C	1.34194900	5.12004400	2.18463300
H	1.83391500	6.10394100	2.11407200
C	1.78451900	5.15010200	-0.87512600
H	2.61794100	4.51124900	-0.54332200
C	-1.06658800	6.05934700	0.35836900
H	-0.75869000	6.98564700	0.87089200
C	-5.02154200	-3.38354700	2.44779700
H	-6.00023500	-3.83017900	2.69000600
C	-4.71315500	-4.46838700	-0.49603200
H	-5.12152000	-5.32414600	0.06653100
C	-6.92860900	-2.31932800	0.16747900
H	-7.50375600	-3.24452400	-0.00303400
C	-1.45957600	6.43275300	-1.08482300
H	-2.32131000	7.11447200	-1.07931800
H	-0.65086400	6.93370200	-1.62684000
H	-1.75445700	5.54606400	-1.65820200
C	-2.30568200	5.50257500	1.09193900

H	-2.10603700	5.25371500	2.13863400
H	-3.11963700	6.24044000	1.07645500
H	-2.67360500	4.59445200	0.60238500
C	2.31850600	6.59574000	-0.93007800
H	1.54749800	7.30323100	-1.25834000
H	2.68813500	6.93987500	0.04327900
H	3.15169200	6.67531200	-1.64164700
C	1.37954000	4.64999500	-2.27471200
H	0.57217900	5.25128300	-2.70599200
H	2.23192700	4.70586600	-2.96594500
H	1.03951500	3.60901200	-2.24782600
C	2.44339200	4.06653500	2.41360000
H	3.18539300	4.05171500	1.60731600
H	2.98179000	4.26379300	3.35085100
H	2.01482300	3.05967700	2.48830100
C	0.39584700	5.16864400	3.39903000
H	0.96674000	5.30501100	4.32801800
H	-0.32110600	5.99371300	3.33205700
H	-0.17476500	4.23716300	3.50170600
C	-3.19369400	-4.68395500	-0.64422400
H	-2.98446300	-5.61160900	-1.19529000
H	-2.73807900	-3.85693200	-1.20142500
H	-2.68137600	-4.75232600	0.32074500
C	-5.37094600	-4.48569100	-1.88879300
H	-5.01148600	-3.65715200	-2.51117400
H	-5.12524400	-5.41695300	-2.41817200
H	-6.46283100	-4.41678400	-1.83800600
C	-6.99648400	-1.47872600	-1.12396500
H	-6.58850700	-2.00411100	-1.99241200
H	-8.03753200	-1.21414300	-1.35668800
H	-6.43303300	-0.54497700	-1.01636000
C	-7.62530200	-1.55884800	1.31197500
H	-7.10627200	-0.61997200	1.54162600
H	-8.65404300	-1.29704300	1.02787900
H	-7.68009800	-2.14524700	2.23550300
C	-4.82756200	-2.15166900	3.35502300
H	-5.61571500	-1.40385700	3.22672900
H	-4.82143800	-2.44794100	4.41345300
H	-3.87304700	-1.65677900	3.14162500
C	-3.94978600	-4.43829900	2.78409800
H	-3.98379900	-4.69332300	3.85249500
H	-4.08088700	-5.36875600	2.22134900
H	-2.93973400	-4.06206400	2.57763500
C	-0.45671100	-1.72489200	0.44765400
C	0.89992600	-0.03047200	1.53419000
C	0.74299200	-2.37847300	1.14859900

H	-0.39580200	-1.81520900	-0.64615900
H	-1.40568700	-2.16286900	0.75938100
C	1.77851800	-1.25020100	1.24018500
H	0.72431500	0.06402500	2.61660000
H	1.36366400	0.89073200	1.18028000
H	0.46401200	-2.69284500	2.16192300
H	1.12118600	-3.25218800	0.61264800
H	2.51276000	-1.41506400	2.03625000
N	-0.35649600	-0.30361400	0.82239100
O	2.43404500	-1.05511300	-0.01068600
Si	3.99597400	-1.47202800	-0.46449200
C	5.10511300	-1.58980000	1.06977600
C	5.16390600	-2.79937600	1.79306400
C	5.84709600	-0.50467200	1.57629900
C	5.92350400	-2.92039400	2.95846800
H	4.61543600	-3.66728500	1.43557400
C	6.61357500	-0.61966000	2.73748800
H	5.83402700	0.45029000	1.06053300
C	6.65338600	-1.82937300	3.43275600
H	5.94815800	-3.86707400	3.49230600
H	7.17935500	0.23546000	3.09855200
H	7.24934900	-1.92105600	4.33715000
C	3.95782800	-3.17573900	-1.28908500
C	2.76868700	-3.67358300	-1.85545300
C	5.11754100	-3.96450900	-1.41701600
C	2.73470400	-4.90469500	-2.51368900
H	1.85649800	-3.08653600	-1.78311600
C	5.09169800	-5.19476400	-2.07761300
H	6.05479300	-3.62162300	-0.98437800
C	3.89827600	-5.66837400	-2.62628200
H	1.80198100	-5.26656800	-2.93911800
H	6.00119000	-5.78463900	-2.16034400
H	3.87519500	-6.62704300	-3.13817100
C	4.49824400	-0.13885200	-1.75150100
C	5.98529200	-0.29880000	-2.13650000
H	6.19648400	-1.29757700	-2.53809200
H	6.25488700	0.42880700	-2.91549600
H	6.65542900	-0.13271500	-1.28488200
C	4.24249800	1.29228700	-1.22837000
H	4.86573100	1.54881100	-0.36437400
H	4.47302500	2.02294000	-2.01738500
H	3.19520900	1.43240100	-0.94054800
C	3.63157700	-0.33670700	-3.01829700
H	3.89571100	0.42043100	-3.77064400
H	3.78242500	-1.32177100	-3.47229100
H	2.56302900	-0.22487300	-2.79985600

Supplementary Note 3

Cartesian coordinates of ***R*-3ab-R2**

C	-5.56683700	2.33561400	1.11206800
C	-4.27828100	1.80938200	1.21361700
C	-3.33311700	2.01584600	0.19606300
C	-3.71248900	2.78327700	-0.91802600
C	-5.00047900	3.30920000	-1.02130100
C	-5.93452400	3.08643300	-0.00669400
H	-6.27913100	2.17189000	1.91702000
H	-3.99313800	1.24304100	2.09512900
H	-2.98609200	2.96475700	-1.70510500
H	-5.27456700	3.89353300	-1.89603000
H	-6.93624900	3.50089600	-0.08331100
C	-1.93041100	1.50021200	0.31982600
C	-1.68792400	0.14366900	0.52188200
C	-2.78804200	-0.76952000	0.39689400
C	-0.94167300	2.50935900	0.19814300
C	-3.68236700	-1.58747700	0.23778000
C	-0.20517500	3.48172200	0.05519000
Si	-5.06230200	-2.78344500	-0.04030200
Si	0.84934200	4.96905900	-0.14495800
C	-4.39847400	-4.23444200	-1.11507300
H	-5.19167400	-4.99881000	-1.07585400
C	-5.52653300	-3.52093300	1.67288700
H	-4.63525000	-4.10646800	1.94724700
C	-6.49796800	-1.87490900	-0.94050600
H	-7.05537500	-2.67482800	-1.45554200
C	2.18315300	4.64315600	-1.50206300
H	2.44661700	5.64508500	-1.87947400
C	1.72326500	5.36861000	1.52904400
H	2.62806900	5.93302900	1.24948300
C	-0.26779700	6.45017900	-0.67189600
H	0.29167400	7.34654400	-0.35605300
C	-7.48254000	-1.15335200	0.00109900
H	-8.28513500	-0.67803200	-0.57961700
H	-7.95609900	-1.83197800	0.71800000
H	-6.98022200	-0.35894000	0.56545400
C	-5.99828700	-0.88164900	-2.01109900
H	-5.32947300	-1.34563000	-2.74211400
H	-6.84950900	-0.45945800	-2.56262500
H	-5.45655000	-0.04786400	-1.55203400
C	-6.71046400	-4.50720200	1.60335900
H	-7.64340200	-4.00394700	1.32166700
H	-6.53988200	-5.31371200	0.88012300
H	-6.88171300	-4.97822100	2.58098600

C	-5.73248500	-2.48352800	2.79330600
H	-6.63305600	-1.88138100	2.63240900
H	-5.84603900	-2.98344500	3.76521000
H	-4.88306000	-1.79584000	2.86789500
C	-3.12007700	-4.86211000	-0.52500600
H	-3.25824000	-5.21058400	0.50496500
H	-2.80128600	-5.72718500	-1.12259800
H	-2.29349000	-4.14109600	-0.52403900
C	-4.17659100	-3.88178900	-2.59790200
H	-3.79341000	-4.75230600	-3.14826700
H	-5.10202600	-3.56597300	-3.09100600
H	-3.44279800	-3.07454600	-2.71406300
C	2.17191300	4.09840200	2.27930200
H	2.72797300	4.36019500	3.19053700
H	1.30254200	3.50241600	2.58198400
H	2.81742800	3.45461800	1.67367000
C	0.90677900	6.26266600	2.48120400
H	-0.01295200	5.76208600	2.80790600
H	1.48777600	6.49268700	3.38536600
H	0.62389300	7.21617000	2.02253300
C	-1.62803000	6.46172600	0.05468700
H	-1.52427300	6.45184800	1.14376100
H	-2.20168400	7.35985000	-0.21472000
H	-2.22915300	5.58831400	-0.22208500
C	-0.49424800	6.57093600	-2.19103800
H	-1.05242000	5.71055200	-2.58043600
H	-1.08478100	7.46866700	-2.42089200
H	0.44404100	6.64242300	-2.75189500
C	1.63603200	3.82239100	-2.68697500
H	0.75553600	4.28043400	-3.14761200
H	2.40002100	3.71304400	-3.46960600
H	1.34612000	2.81590500	-2.36347000
C	3.48305000	3.99764700	-0.98640200
H	4.21044900	3.89357200	-1.80392800
H	3.96113600	4.58576300	-0.19563000
H	3.29999200	2.99133400	-0.58967200
C	0.79989100	0.25133600	0.94549200
C	-0.32780400	-1.89803300	1.02373600
C	1.83453900	-0.86397500	1.16325400
H	0.76325700	0.92996300	1.80748700
H	1.04897800	0.84135900	0.06229100
C	0.99673400	-1.98894800	1.78629600
H	-0.27611700	-2.44664700	0.07223100
H	-1.17434700	-2.28938300	1.59147800
H	2.64156200	-0.52481200	1.82159000
H	1.47758500	-2.96597700	1.70013900

H	0.83293200	-1.77294300	2.84937900
N	-0.47842700	-0.45371000	0.79070600
O	2.36436100	-1.26273400	-0.10012000
Si	3.92576800	-1.76809100	-0.46674700
C	5.15805000	-0.93902300	0.71291200
C	5.36011200	-1.48919400	1.99620400
C	5.85430700	0.24688800	0.40890400
C	6.20937500	-0.88761600	2.92701800
H	4.85430000	-2.41144900	2.27219700
C	6.71103300	0.85040900	1.33173000
H	5.73521800	0.71334100	-0.56362100
C	6.88950200	0.28525200	2.59528500
H	6.34262900	-1.33704100	3.90787500
H	7.23747700	1.76269700	1.06313200
H	7.55454100	0.75469300	3.31555100
C	4.05647800	-3.64050500	-0.21126200
C	2.92066900	-4.46629200	-0.32010700
C	5.29386500	-4.26970800	0.02567900
C	3.01209200	-5.85362400	-0.19052100
H	1.94948600	-4.01705400	-0.51291600
C	5.39356100	-5.65720000	0.15200000
H	6.19499200	-3.66897400	0.12502300
C	4.25114800	-6.45255000	0.04612200
H	2.11814300	-6.46645100	-0.27609000
H	6.36170700	-6.11602200	0.33674200
H	4.32572300	-7.53228300	0.14724100
C	4.13475300	-1.35106600	-2.32839700
C	5.58382600	-1.63481800	-2.78109400
H	5.86740700	-2.67969300	-2.60449500
H	5.68674500	-1.44696700	-3.85946200
H	6.31328400	-0.99907100	-2.26570400
C	3.76517800	0.11768500	-2.62916700
H	4.43771300	0.83526500	-2.14718100
H	3.82072200	0.30279200	-3.71167100
H	2.74546500	0.34787100	-2.30366500
C	3.17488300	-2.25380100	-3.13982900
H	3.27353300	-2.03070100	-4.21198000
H	3.39315600	-3.31820200	-3.00193400
H	2.12812300	-2.08260800	-2.86291800
