# Supplementary material for: Streamlined asymmetric α-difunctionalization of ynones
Source: Nat Commun. 2018 Jan 25;9:375. doi: 10.1038/s41467-017-02801-9 (PMC5785506; doi:10.1038/s41467-017-02801-9)
Supplement: Supplementary file 3 — Description of Additional Supplementary Files [file 41467_2017_2801_MOESM3_ESM.pdf]

Description of Additional Supplementary Files

File Name: Supplementary Data 1
Description: data shelx
